# Supplementary material for: Global multi-ancestry genome-wide analyses identify genes and biological pathways associated with thyroid cancer and benign thyroid diseases
Source: Nat Genet. 2026 Feb 5;58(2):307–16. doi: 10.1038/s41588-025-02483-w (PMC12900643; doi:10.1038/s41588-025-02483-w)

nature genetics

Supplementary information

https://doi.org/10.1038/s41588-025-02483-w

Global multi-ancestry genome-wide analyses identify genes and biological pathways associated with thyroid cancer and benign thyroid diseases

In the format provided by the authors and unedited

Supplementary Figure 1. Manhattan and quartile-quartile (QQ) plots for ancestry stratified inverse-variance weighted meta-analysis results. Manhattan plots show the -log10(p-value) (y-axis) of each meta-analyzed SNP vs their genomic coordinate (x-axis). Genome wide significance, -log10(5e-8), is marked by a red horizontal line. QQ plots are displayed in the lower half of the figure.

Supplementary Figure 1

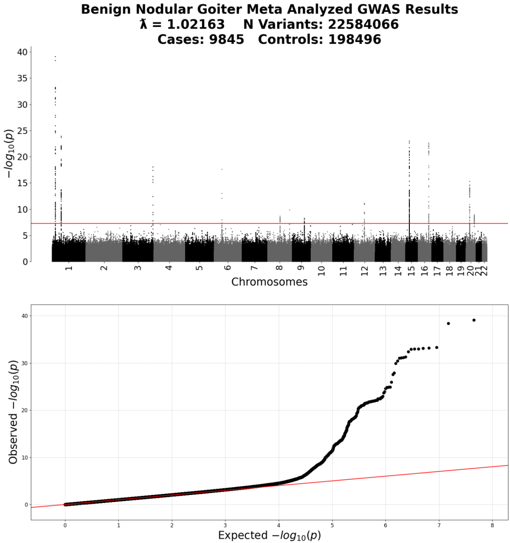

African Ancestry

Supplementary Figure 1

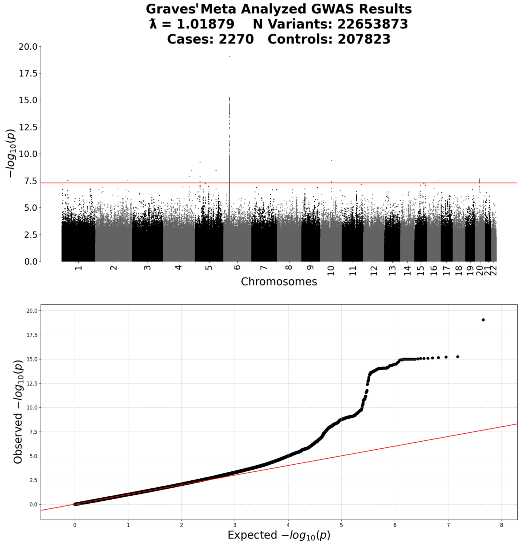

African Ancestry

Supplementary Figure 1

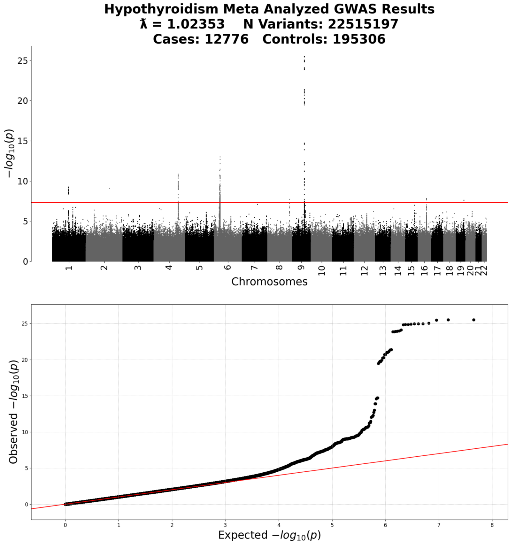

African Ancestry

Supplementary Figure 1

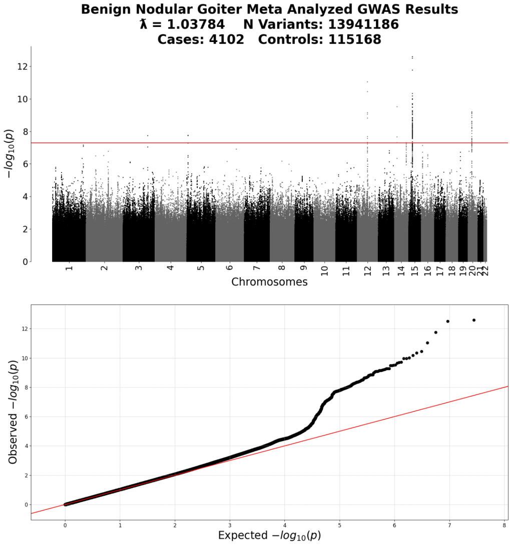

Admixed American Ancestry

Supplementary Figure 1

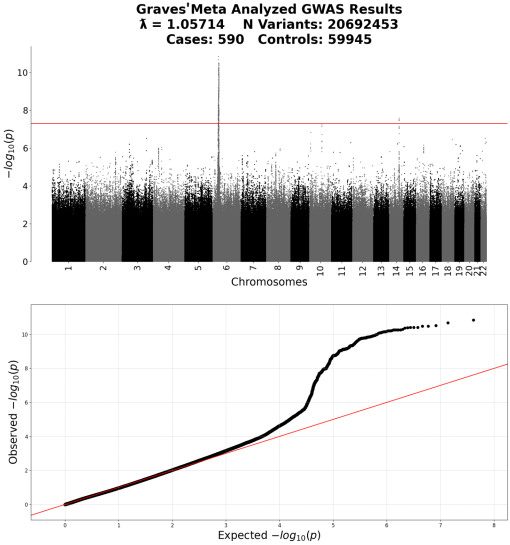

Admixed American Ancestry

Supplementary Figure 1

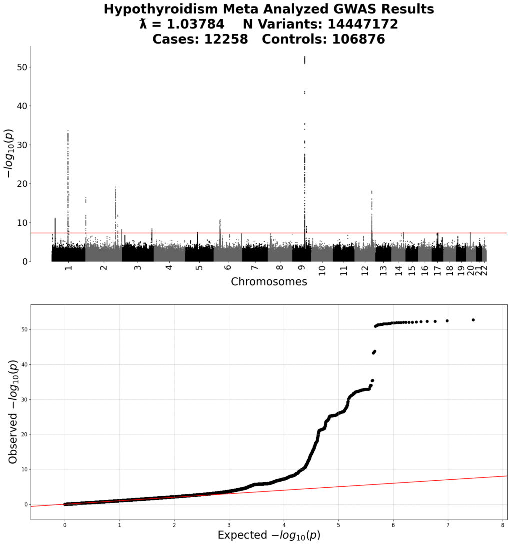

Admixed American Ancestry

Supplementary Figure 1

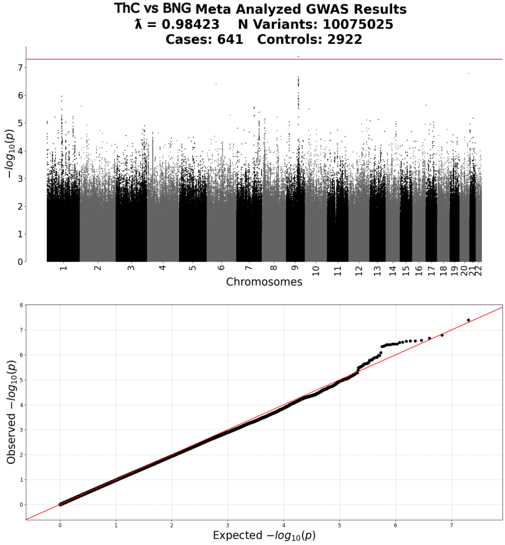

Admixed American Ancestry

Supplementary Figure 1

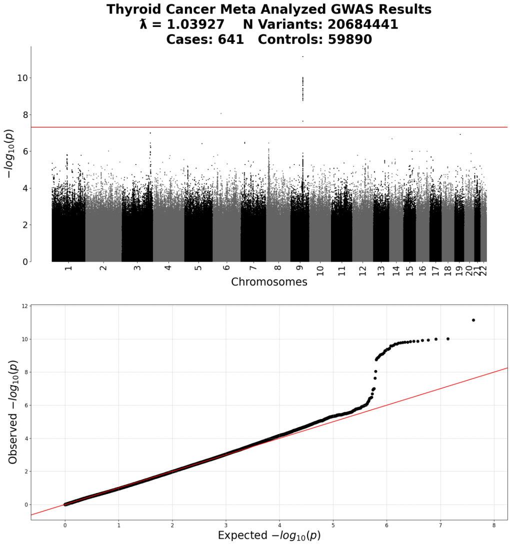

Admixed American Ancestry

Supplementary Figure 1

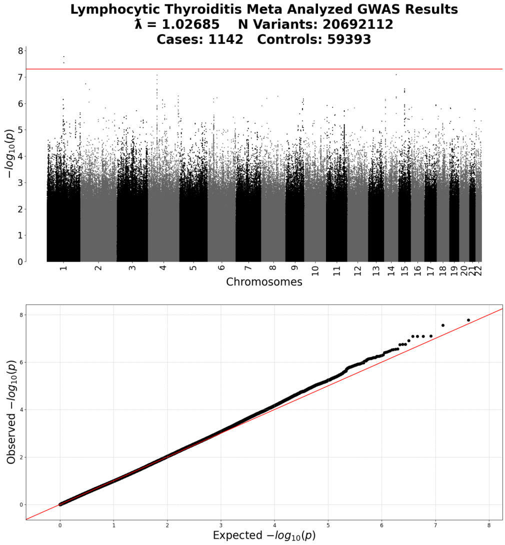

Admixed American Ancestry

Supplementary Figure 1

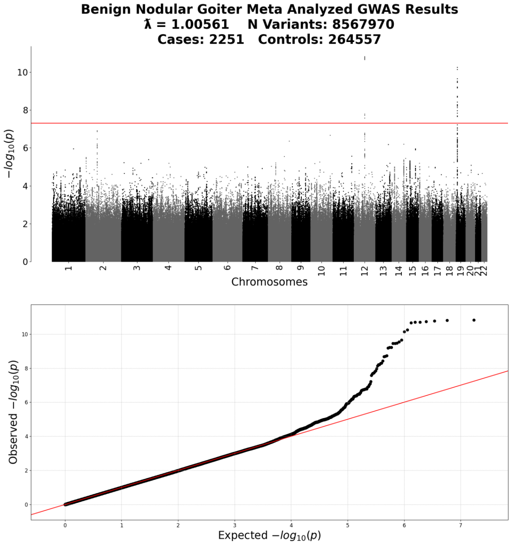

East Asian Ancestry

Supplementary Figure 1

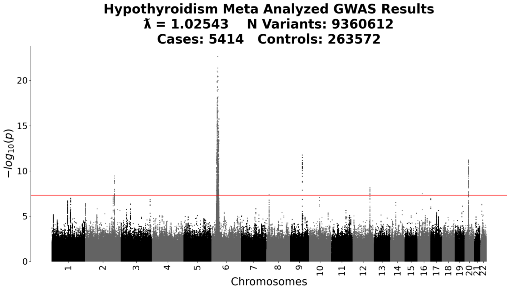

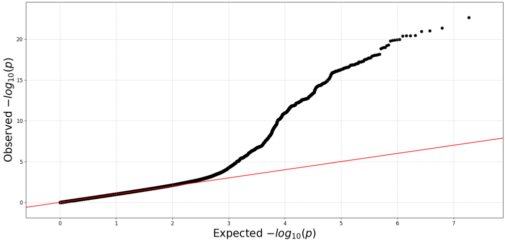

East Asian Ancestry

Supplementary Figure 1

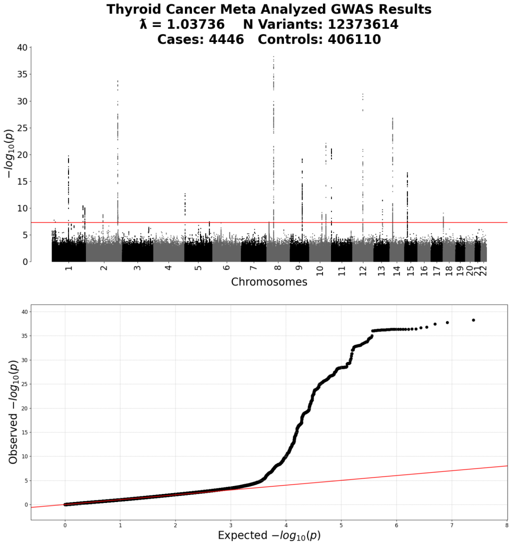

East Asian Ancestry

Supplementary Figure 1

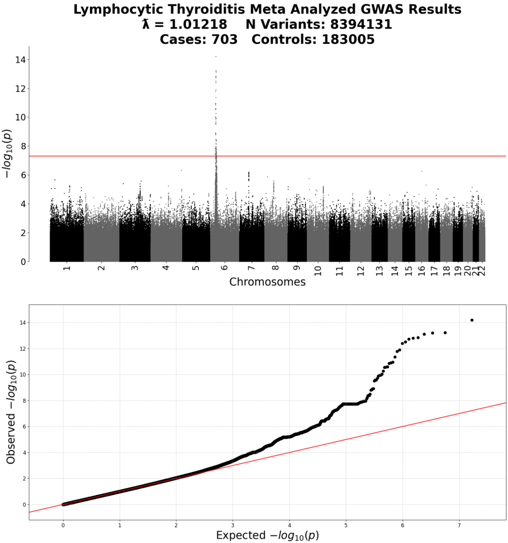

East Asian Ancestry

Supplementary Figure 1

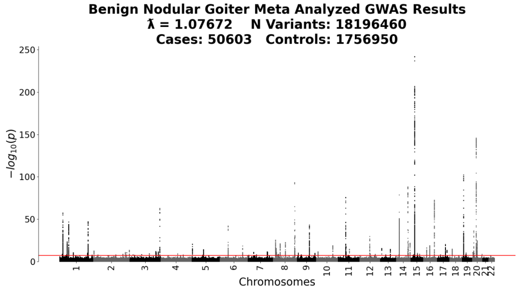

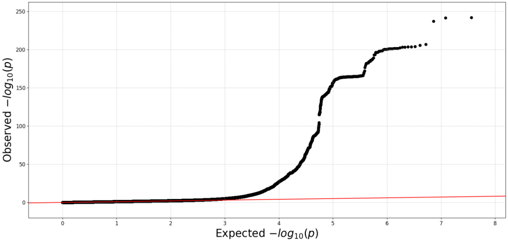

European Ancestry

Supplementary Figure 1

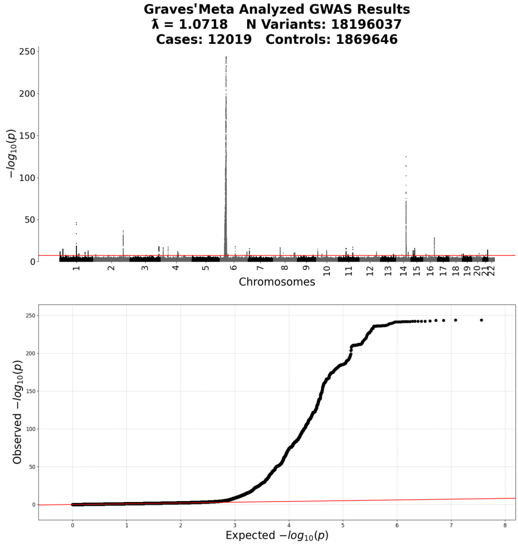

European Ancestry

Supplementary Figure 1

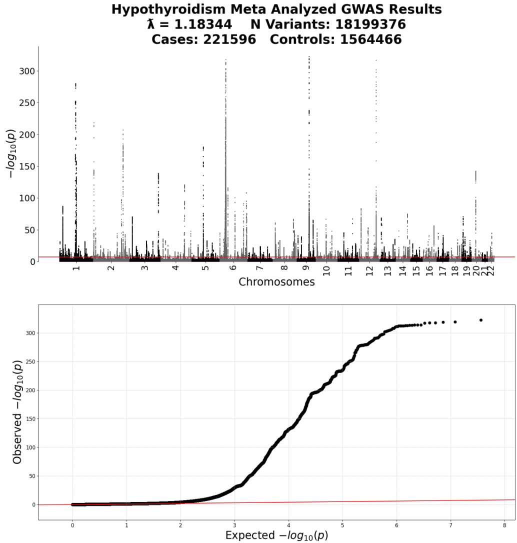

European Ancestry

Supplementary Figure 1

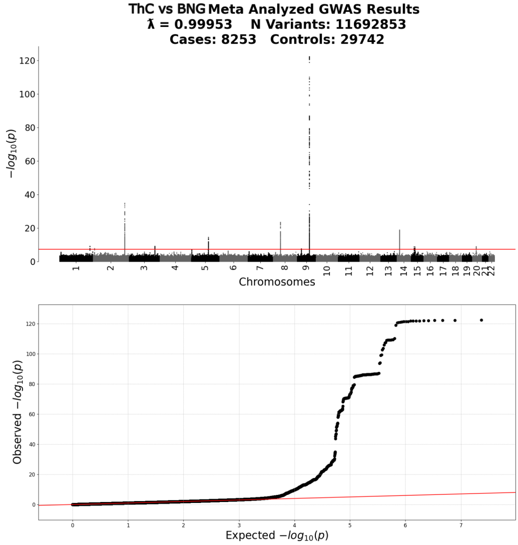

European Ancestry

Supplementary Figure 1

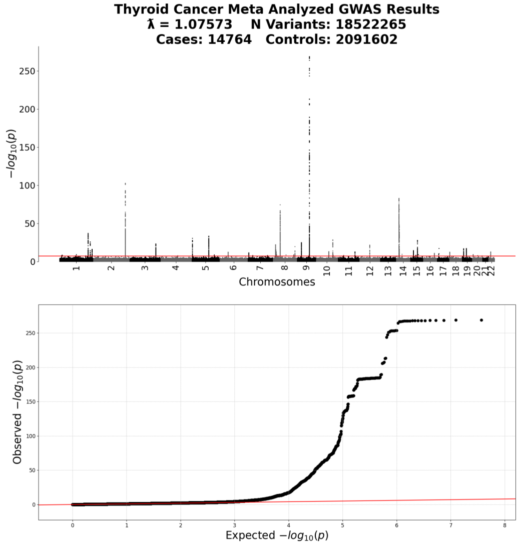

European Ancestry

Supplementary Figure 1

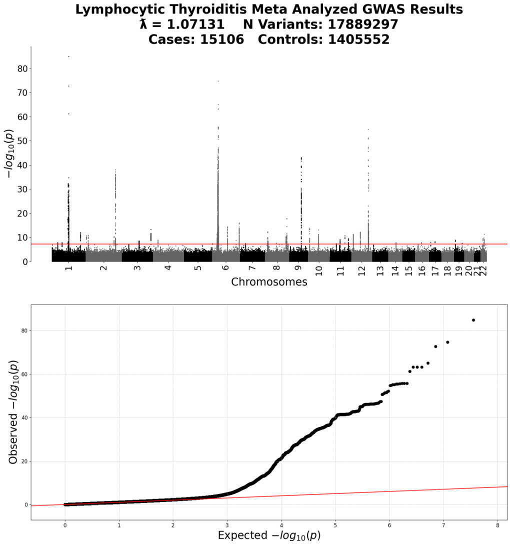

European Ancestry

Supplementary Figure 1

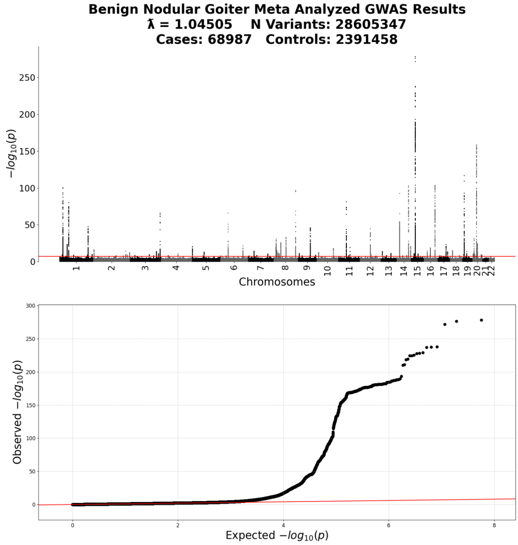

Mixed Ancestry

Supplementary Figure 1

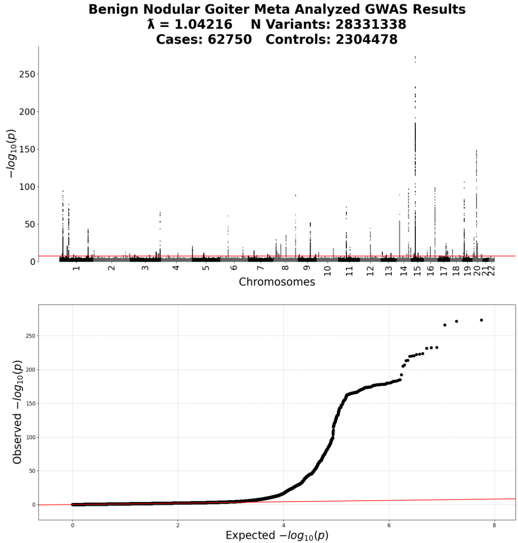

Mixed Ancestry (Leave CCPM Out)

Supplementary Figure 1

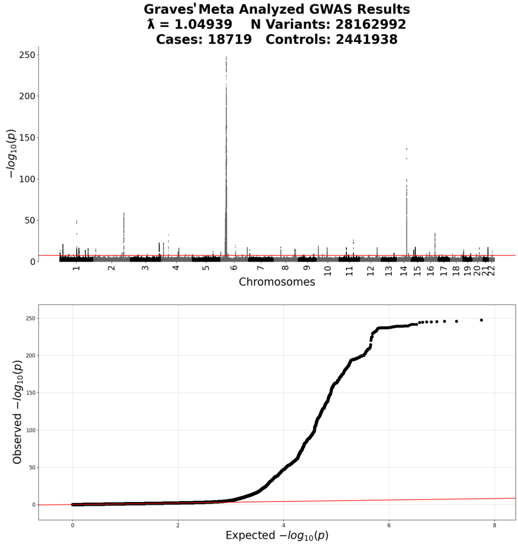

Mixed Ancestry

Supplementary Figure 1

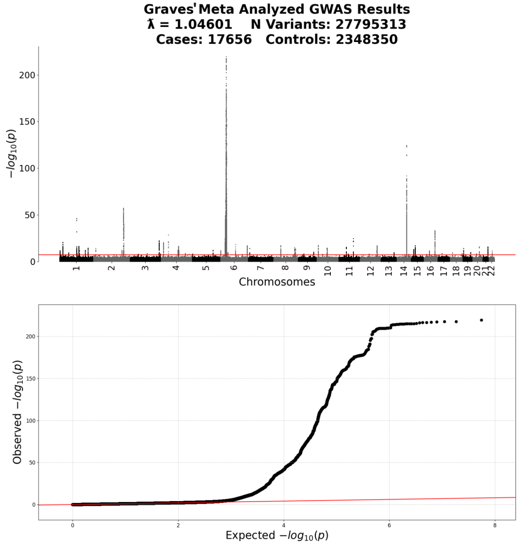

Mixed Ancestry (Leave CCPM Out)

Supplementary Figure 1

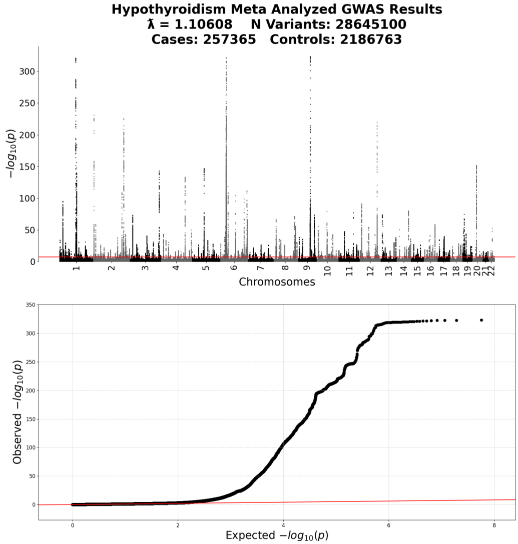

Mixed Ancestry

Supplementary Figure 1

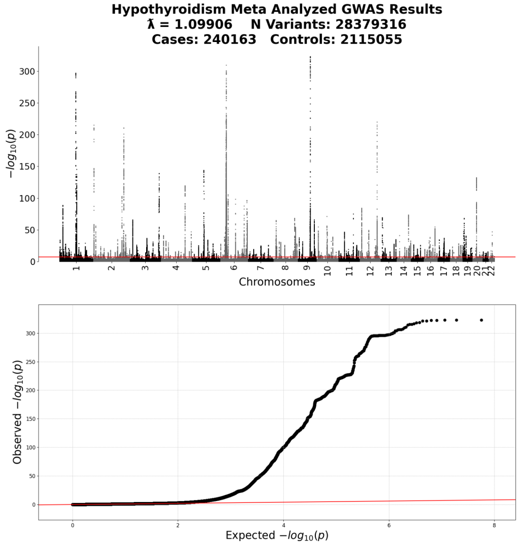

Mixed Ancestry (Leave CCPM Out)

Supplementary Figure 1

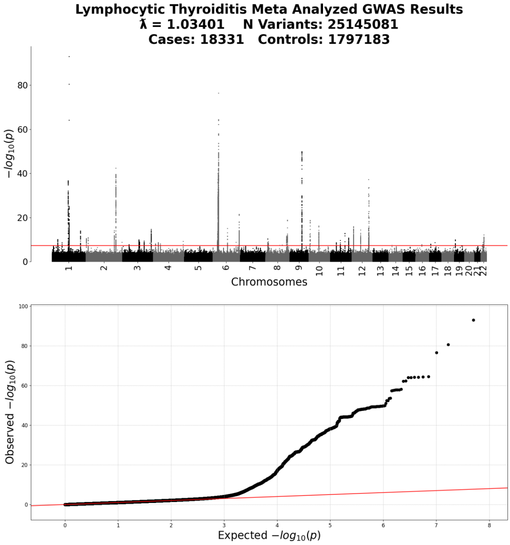

Mixed Ancestry

Supplementary Figure 1

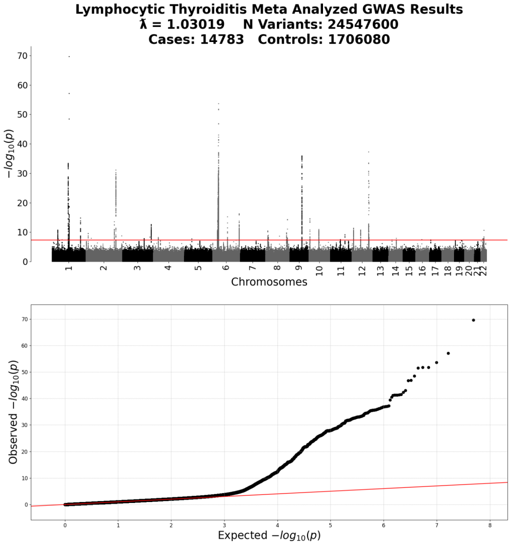

Mixed Ancestry (Leave CCPM Out)

Supplementary Figure 1

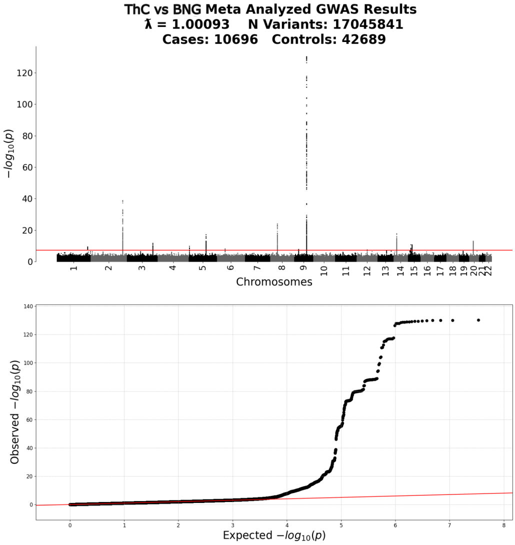

Mixed Ancestry

Supplementary Figure 1

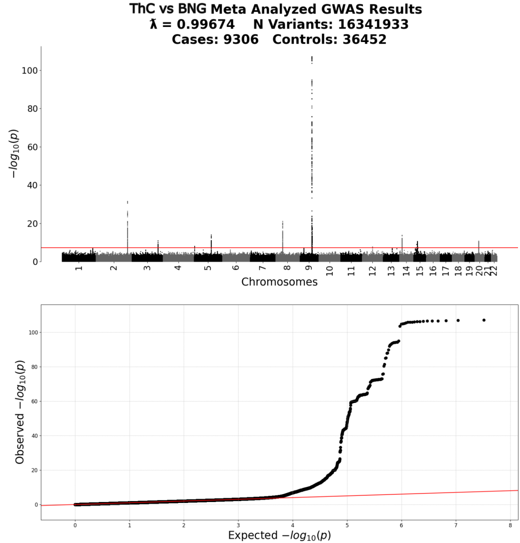

Mixed Ancestry (Leave CCPM Out)

Supplementary Figure 1

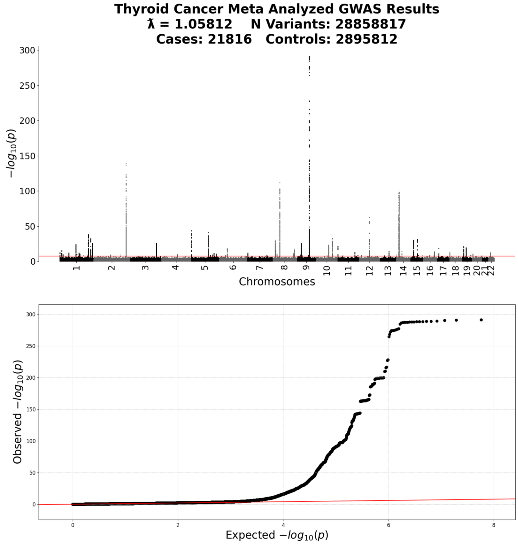

Mixed Ancestry

Supplementary Figure 1

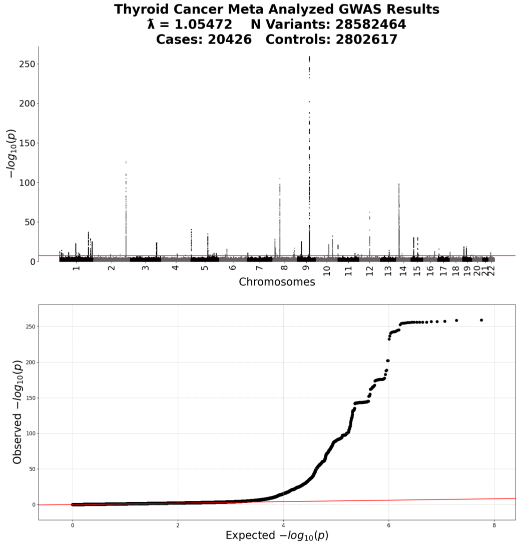

Mixed Ancestry (Leave CCPM Out)

Supplementary Figure 2.1. Locus plots for loci significantly associated with thyroid cancer but not benign nodular goiter. The left plot displays -log10(p-values) for variants from thyroid cancer meta-analysis, the right plot displays -log10(p-values) for variants within the same genomic region from the benign nodular goiter meta-analysis. The lead variant is shown with a purple diamond. Genome wide significance is indicated by the dashed horizontal line at -log10(5e-8).

Supplementary Figure 2.1

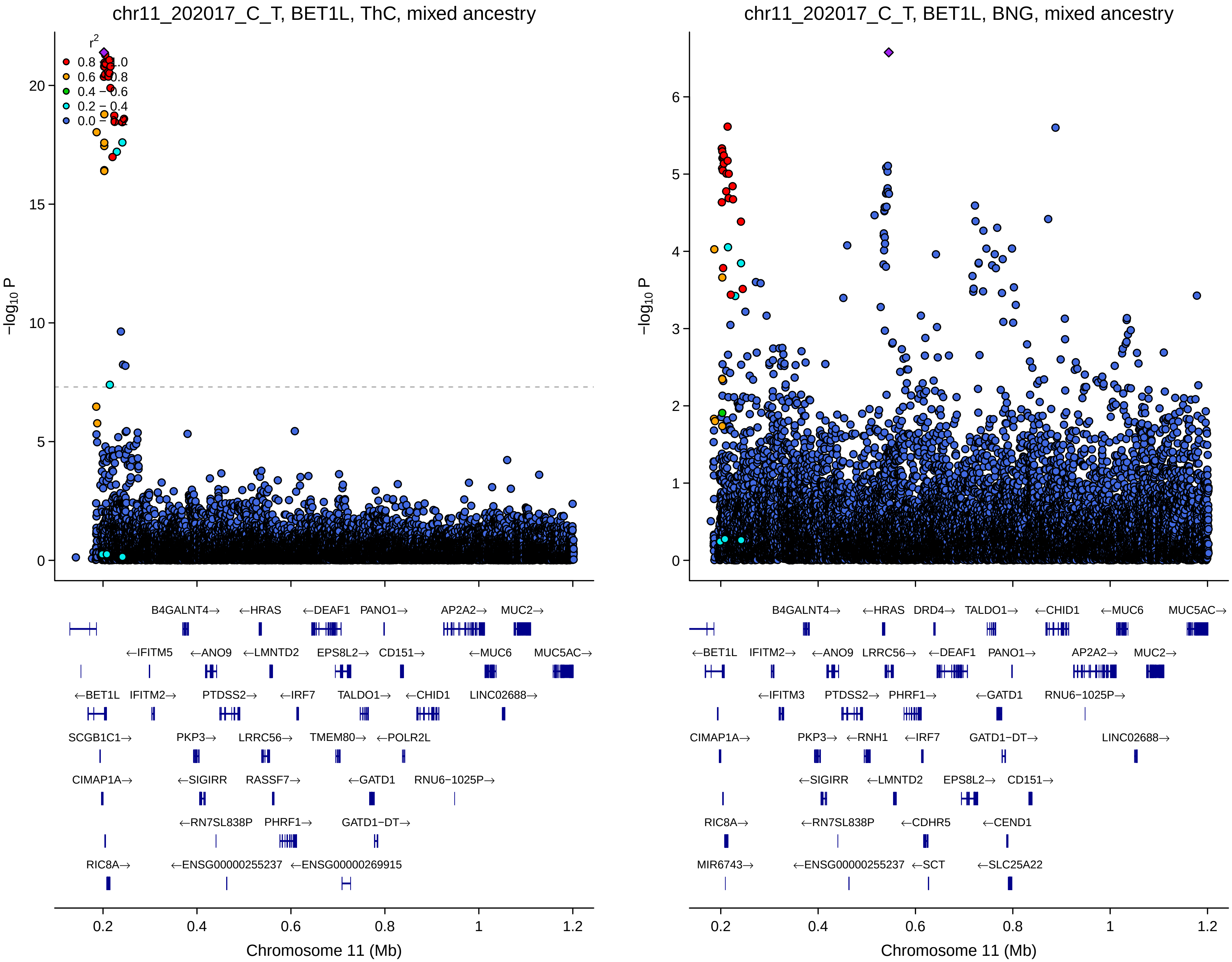

Supplementary Figure 2.1

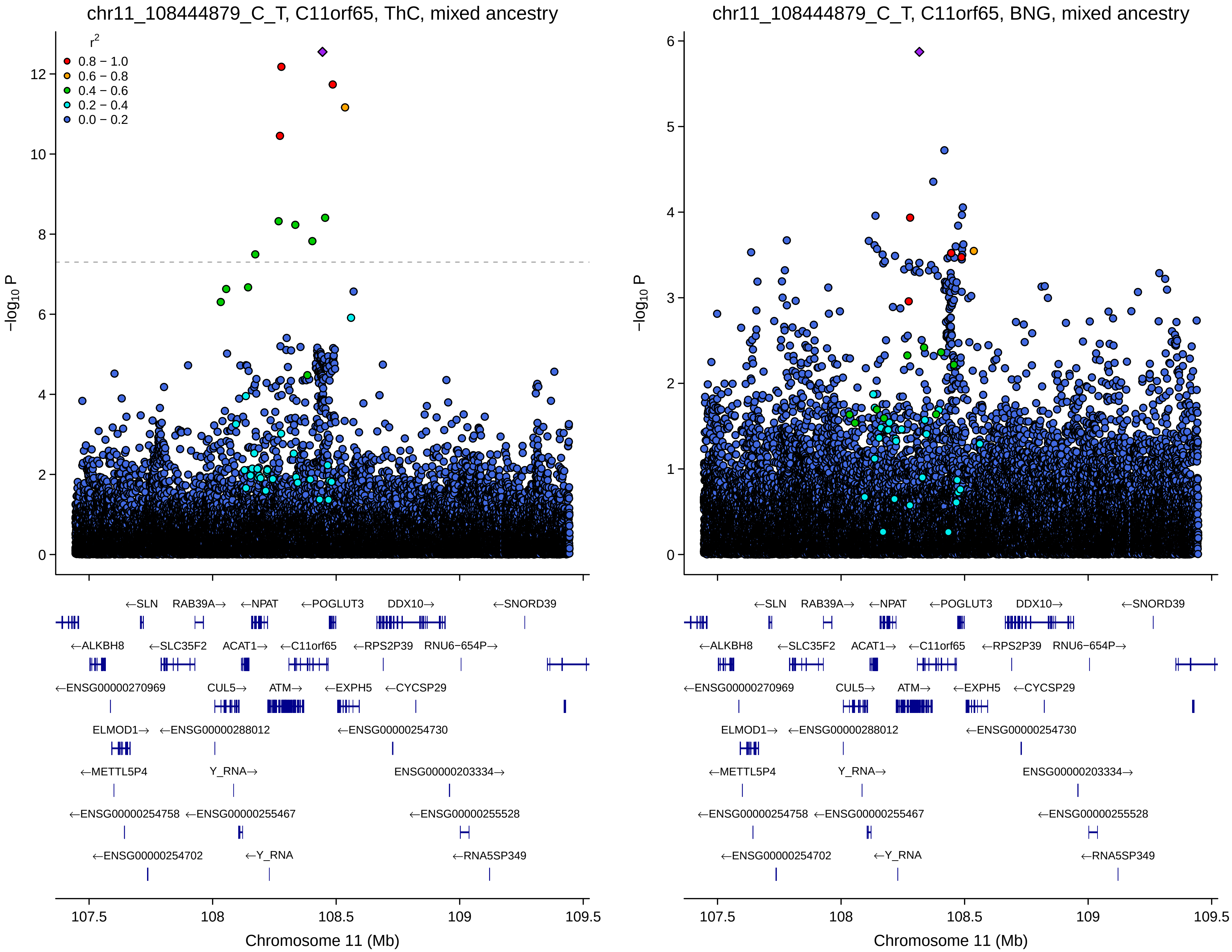

Supplementary Figure 2.1

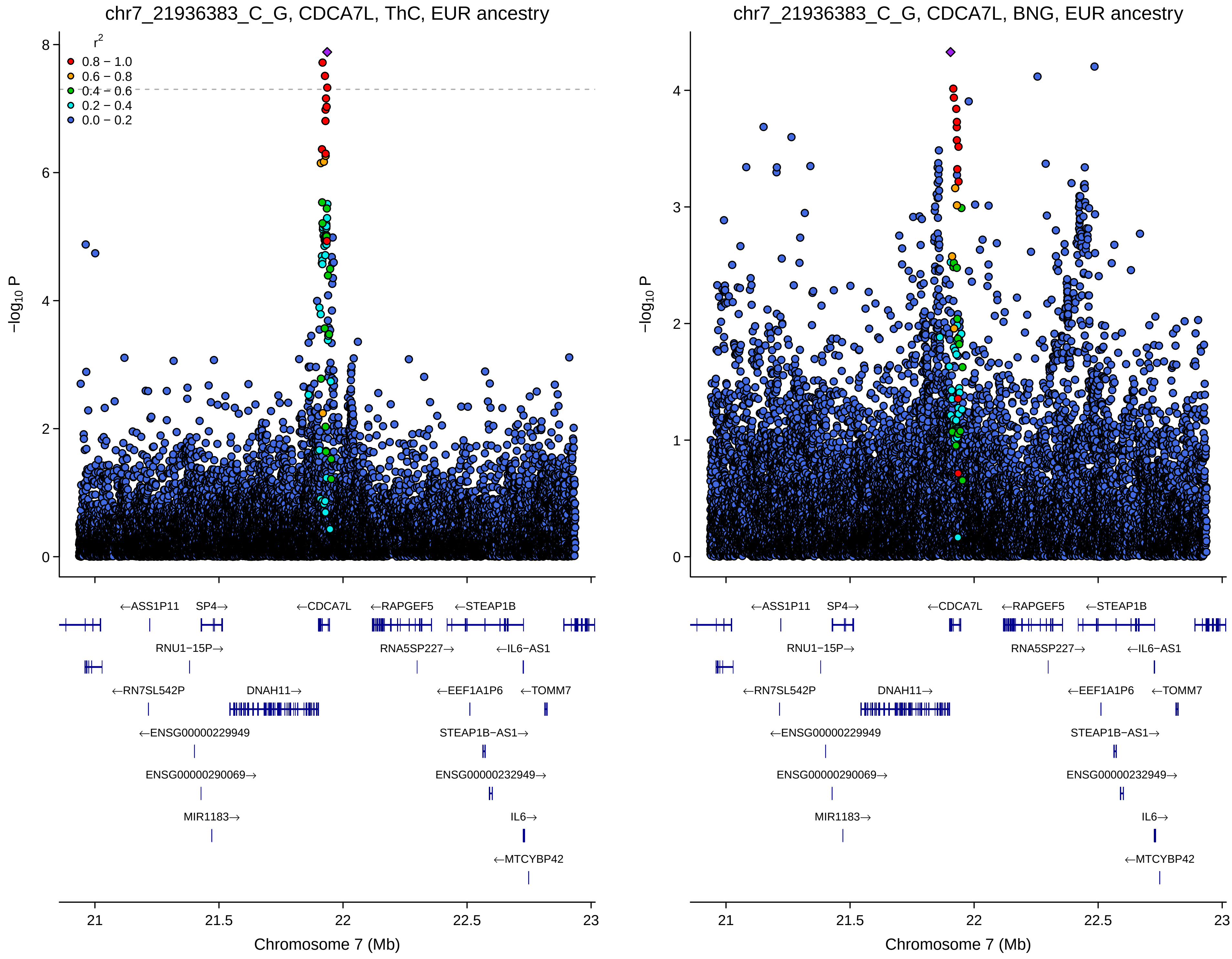

Supplementary Figure 2.1

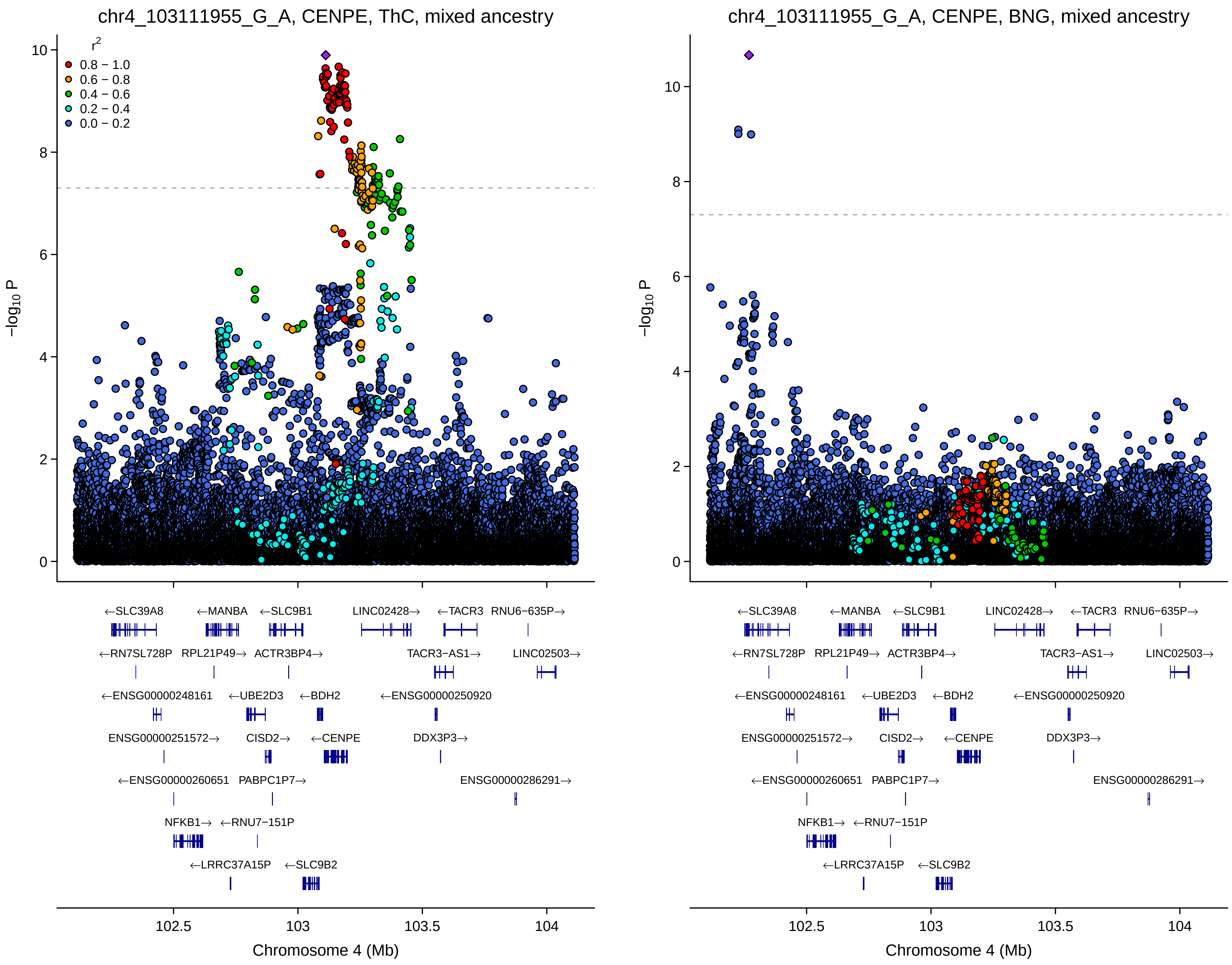

Supplementary Figure 2.1

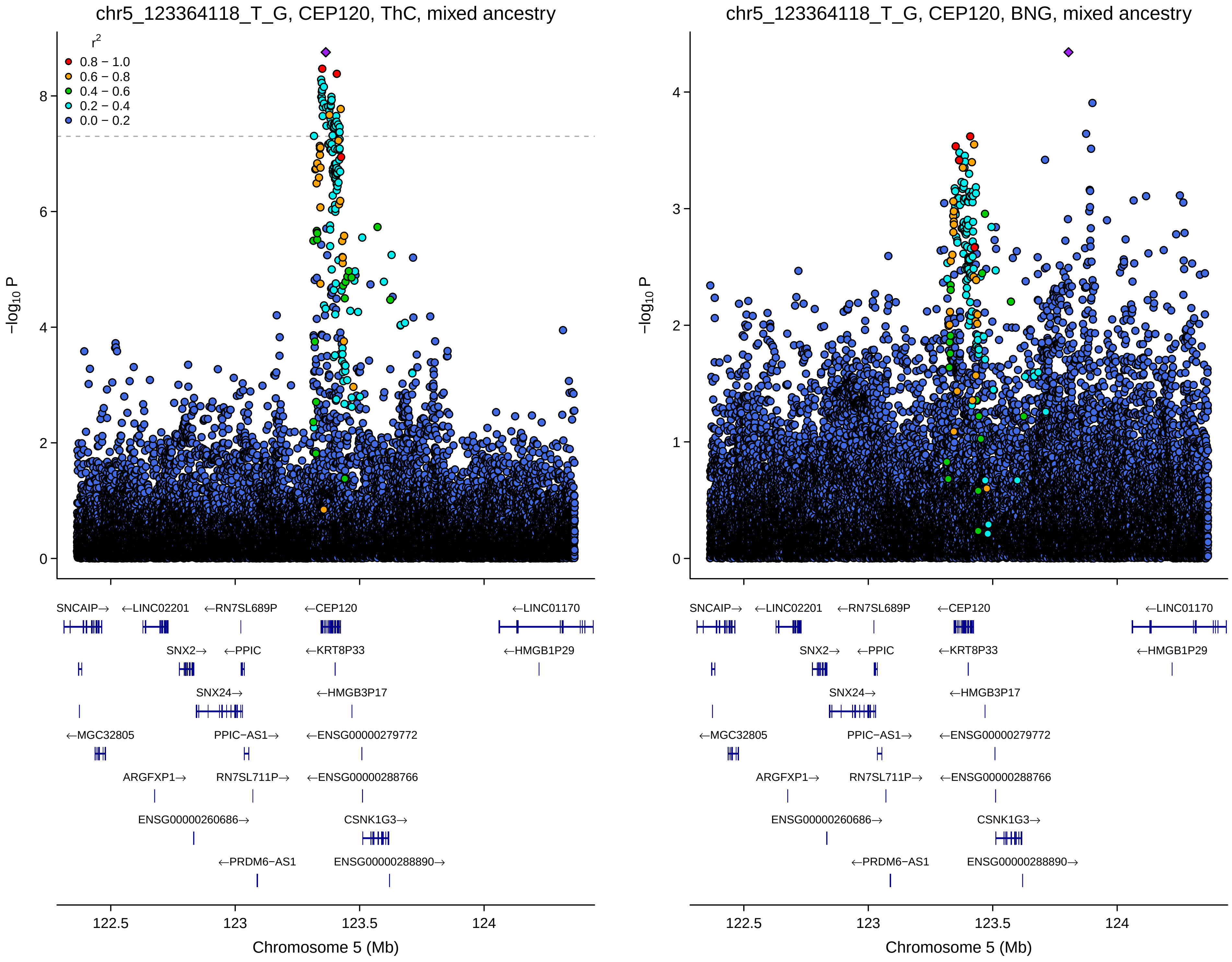

Supplementary Figure 2.1

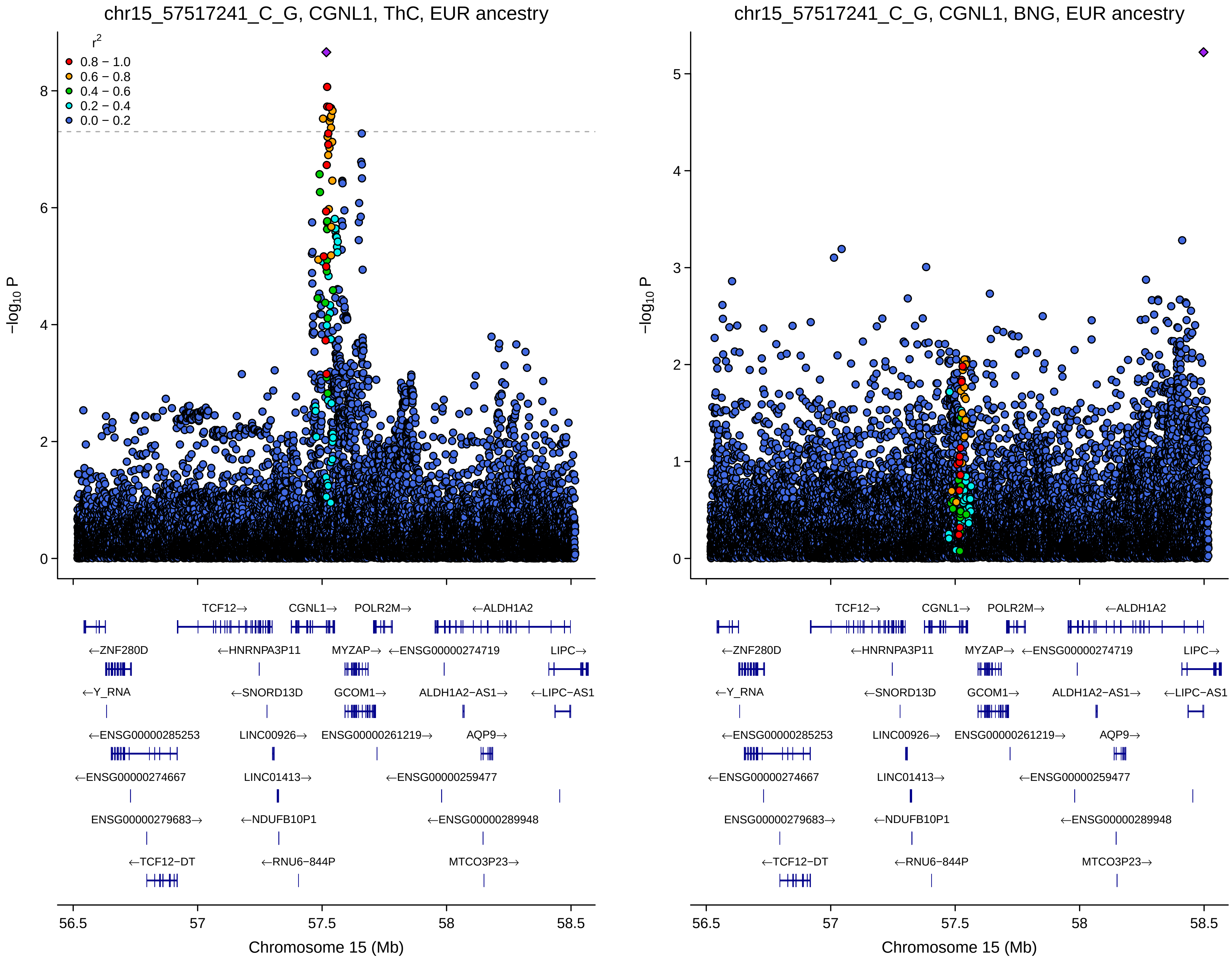

Supplementary Figure 2.1

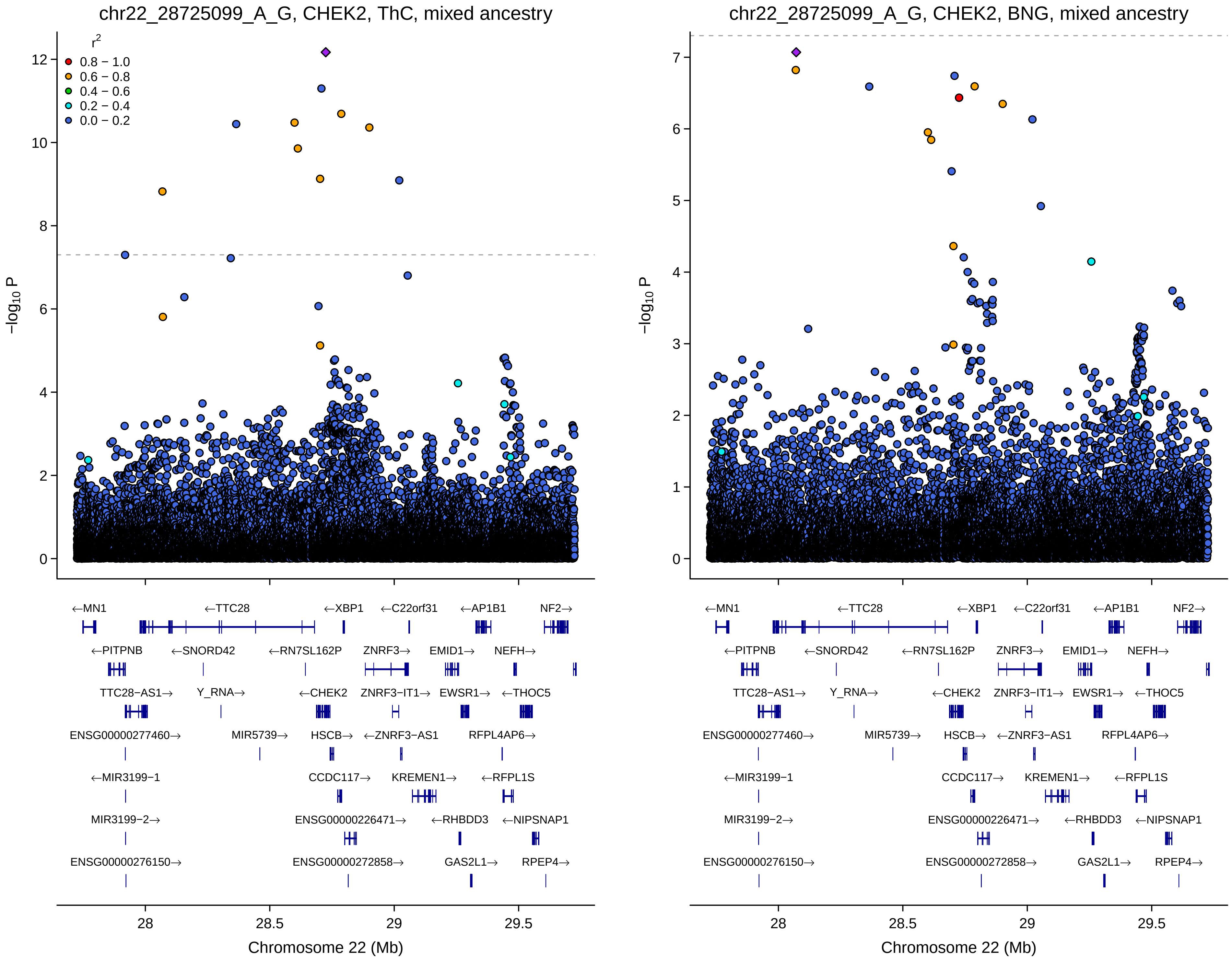

Supplementary Figure 2.1

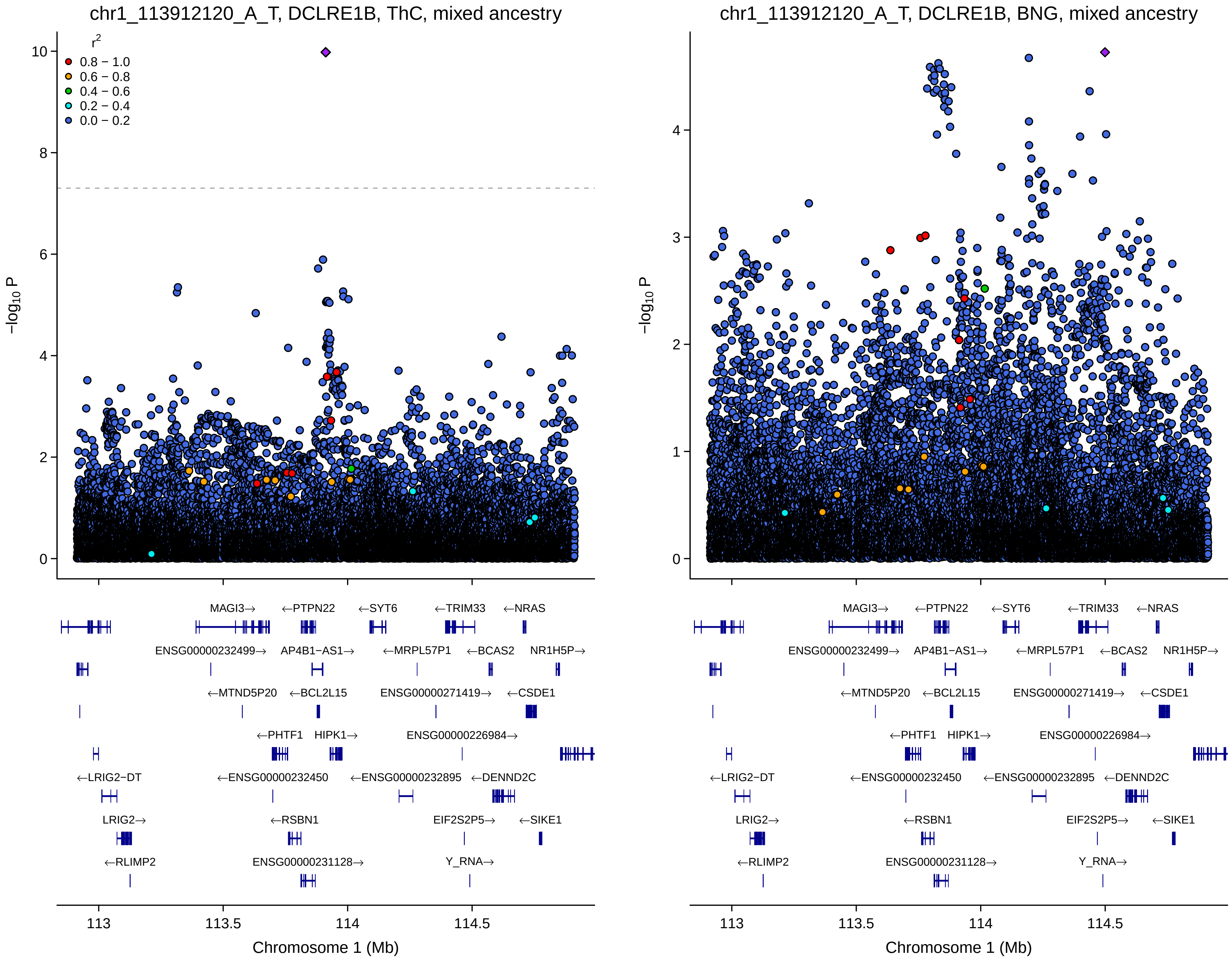

Supplementary Figure 2.1

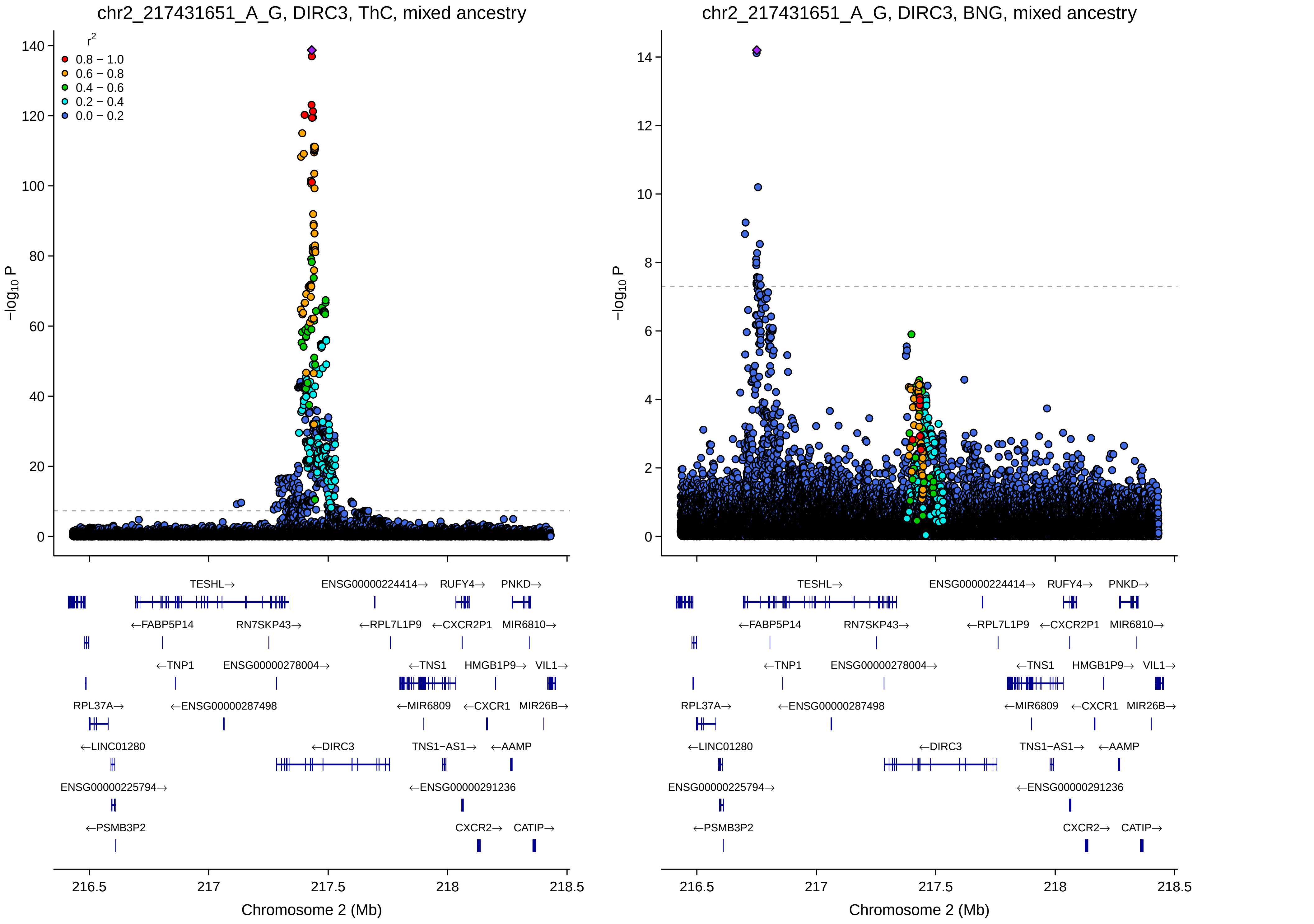

Supplementary Figure 2.1

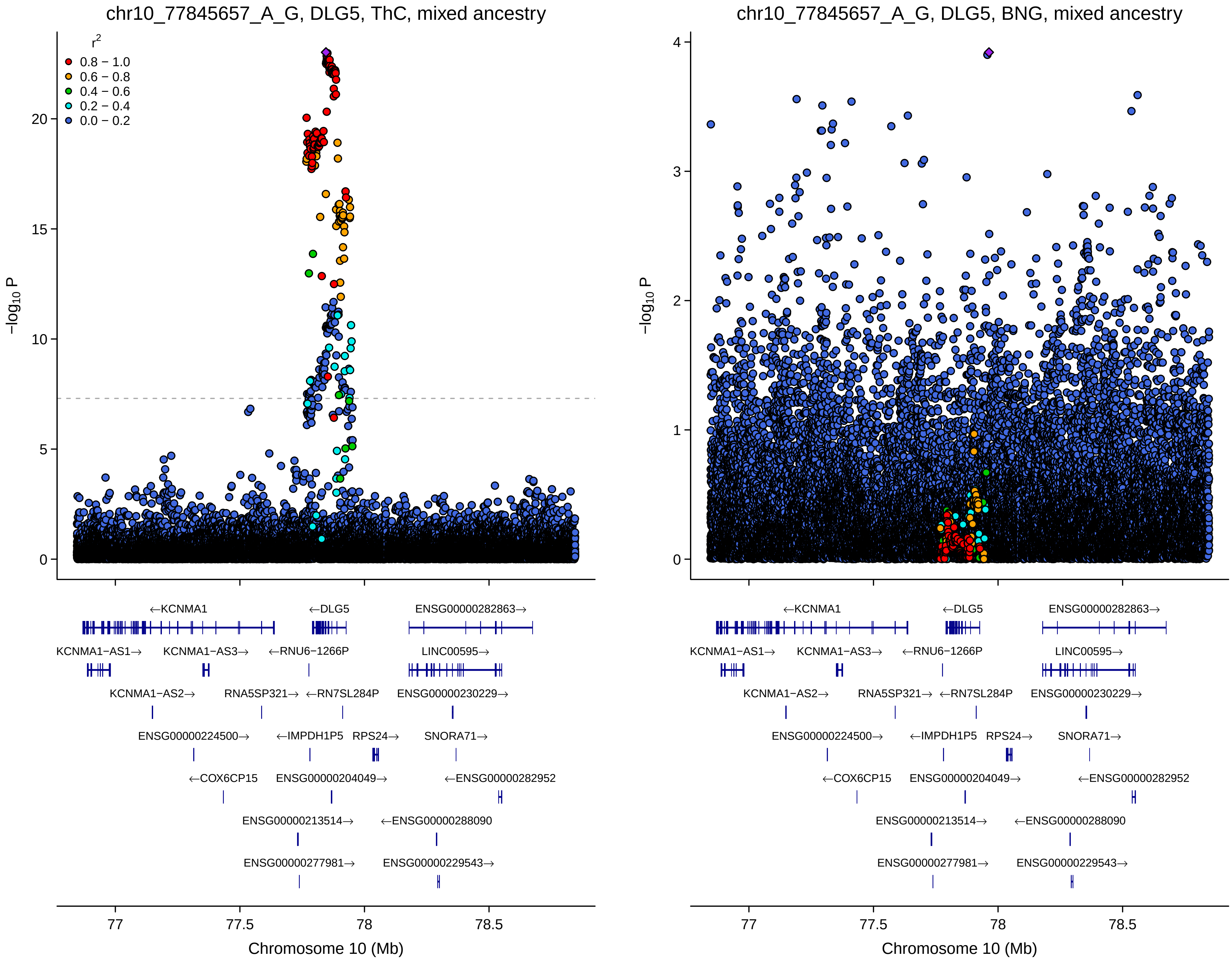

Supplementary Figure 2.1

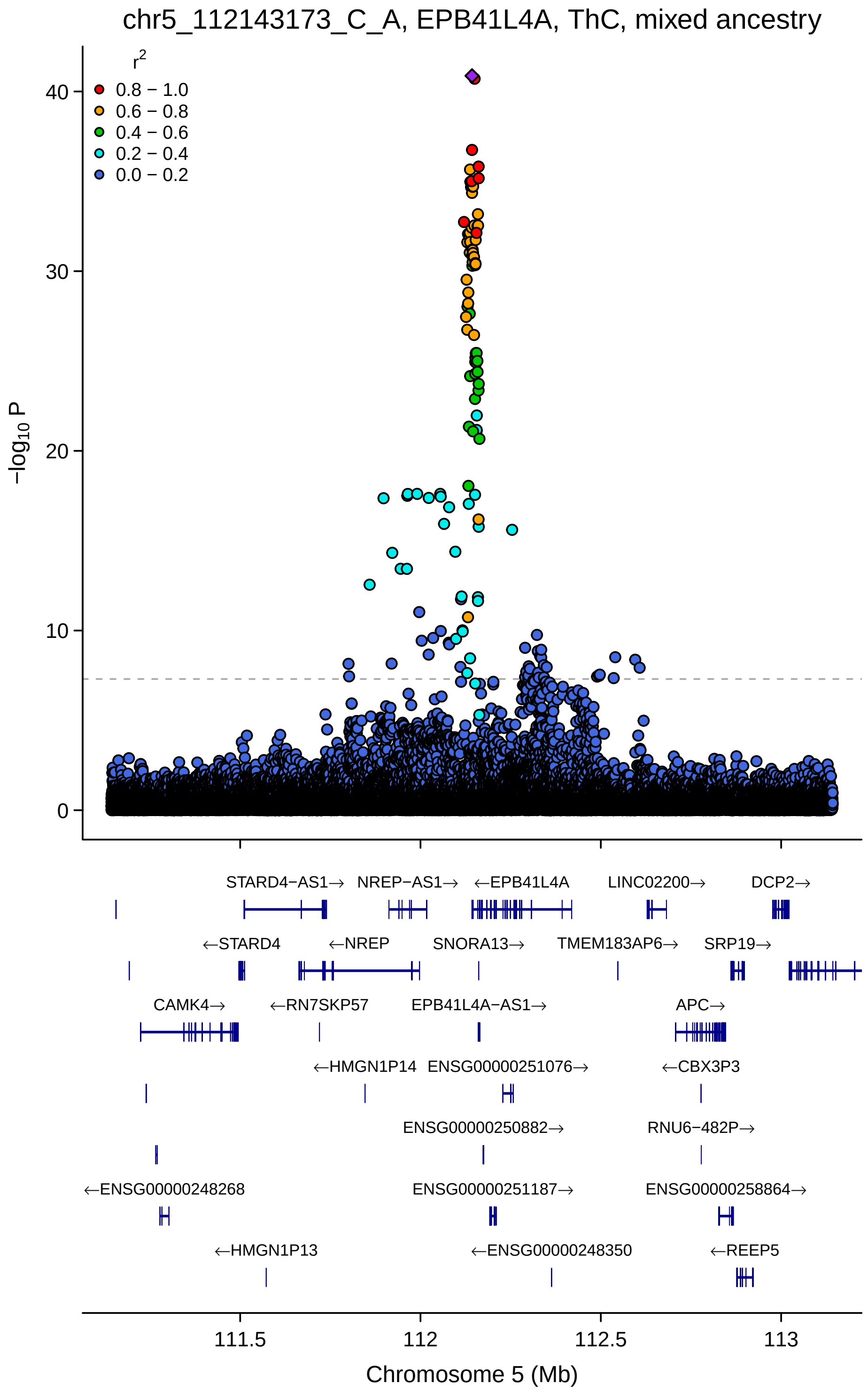

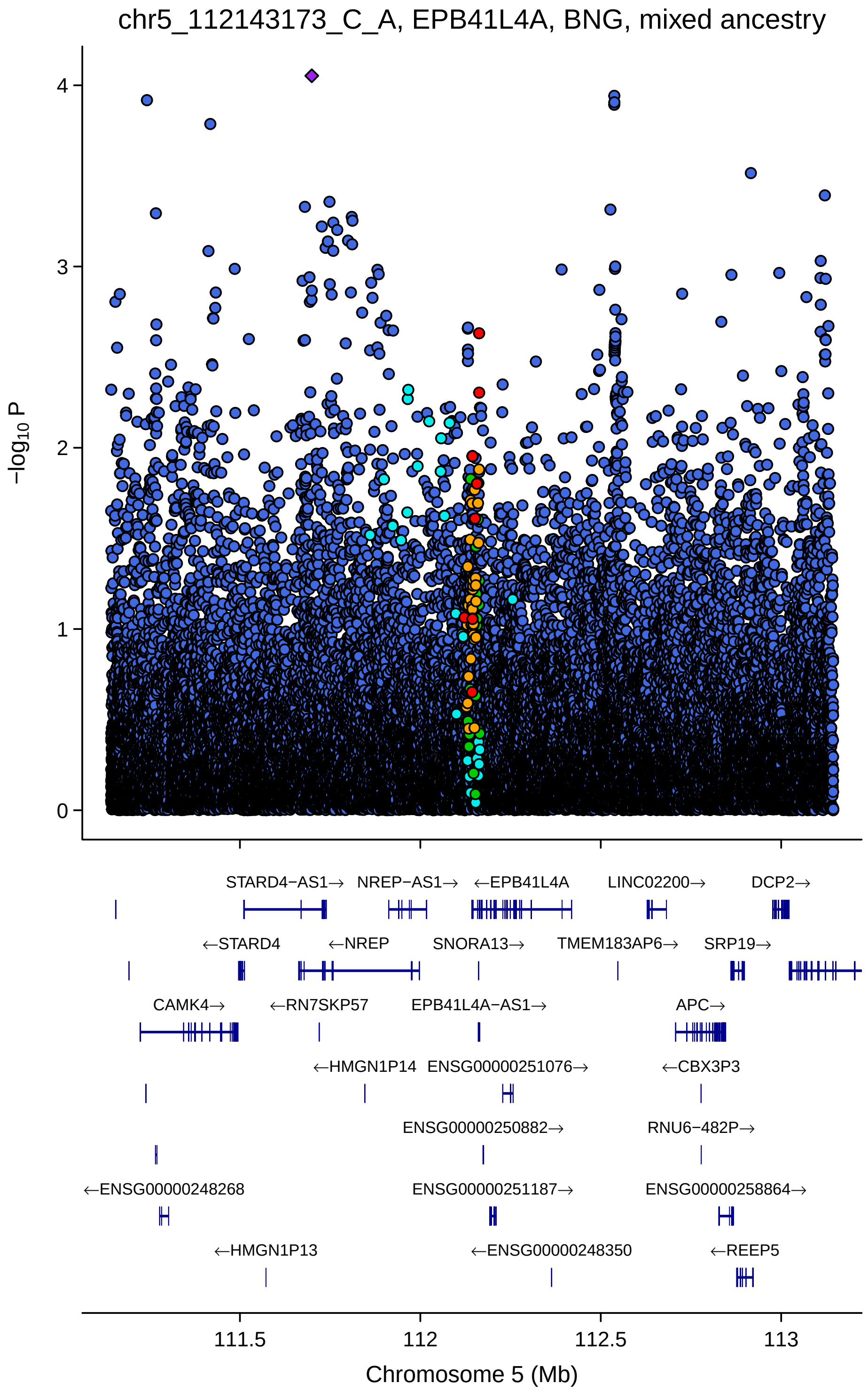

Supplementary Figure 2.1

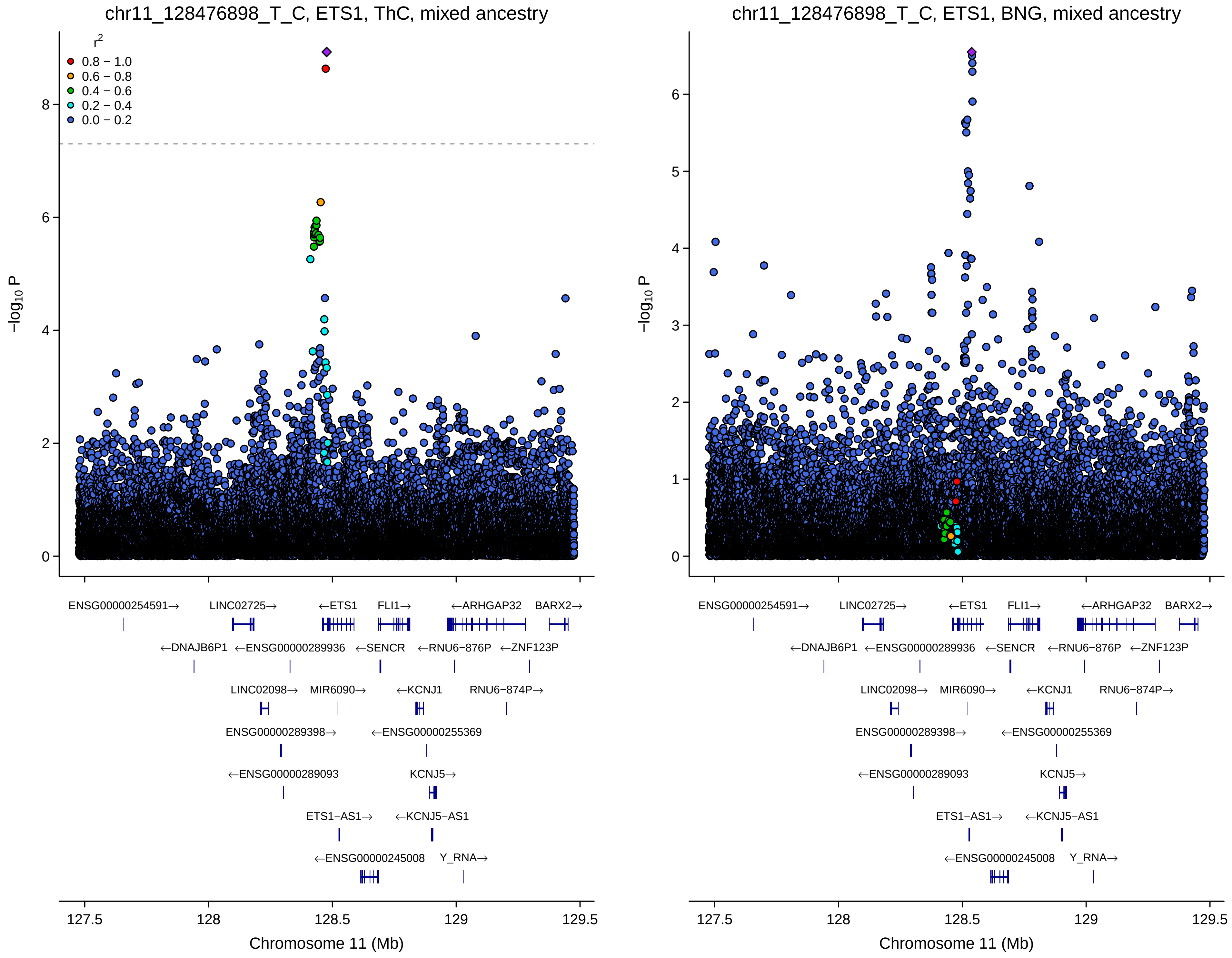

Supplementary Figure 2.1

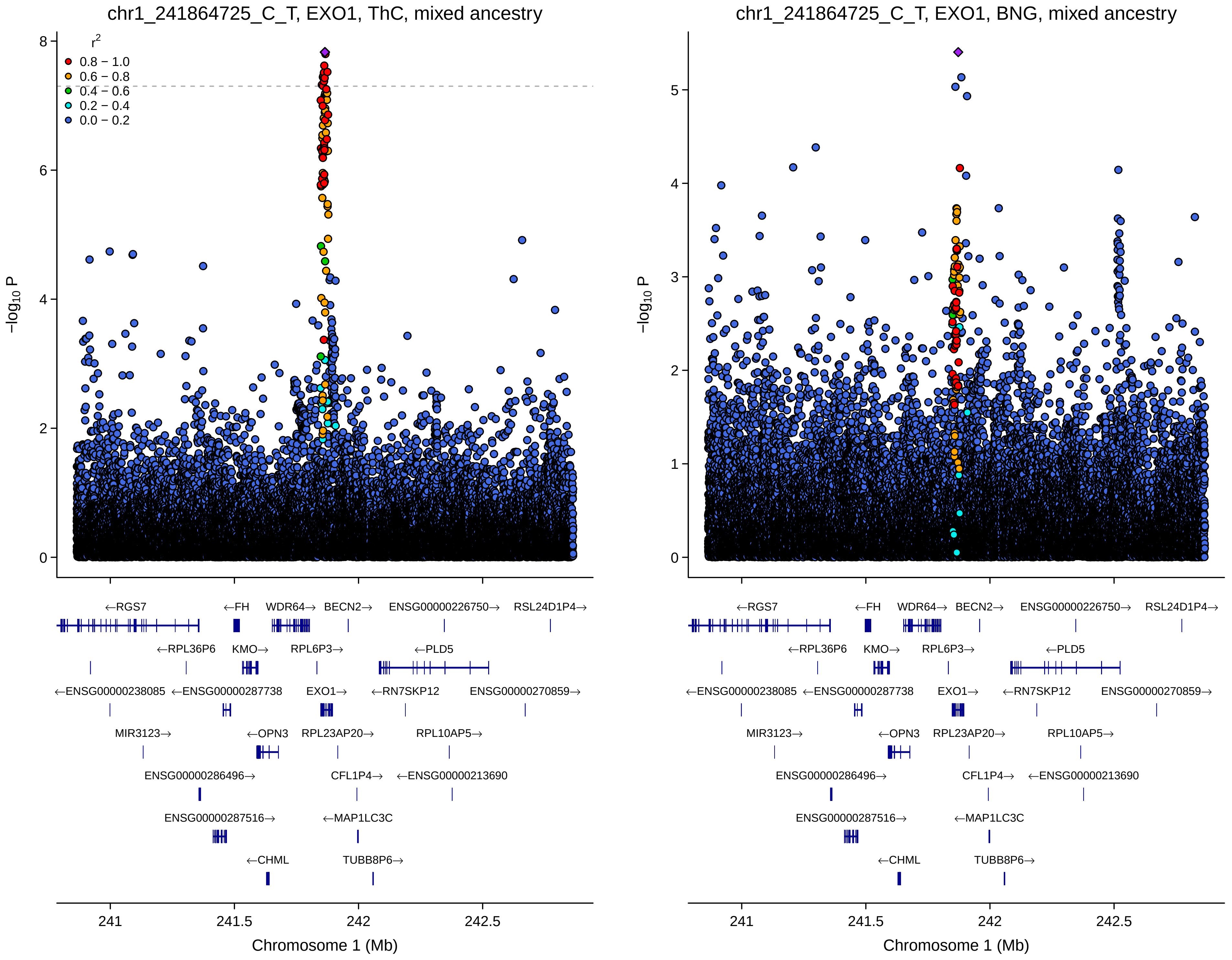

Supplementary Figure 2.1

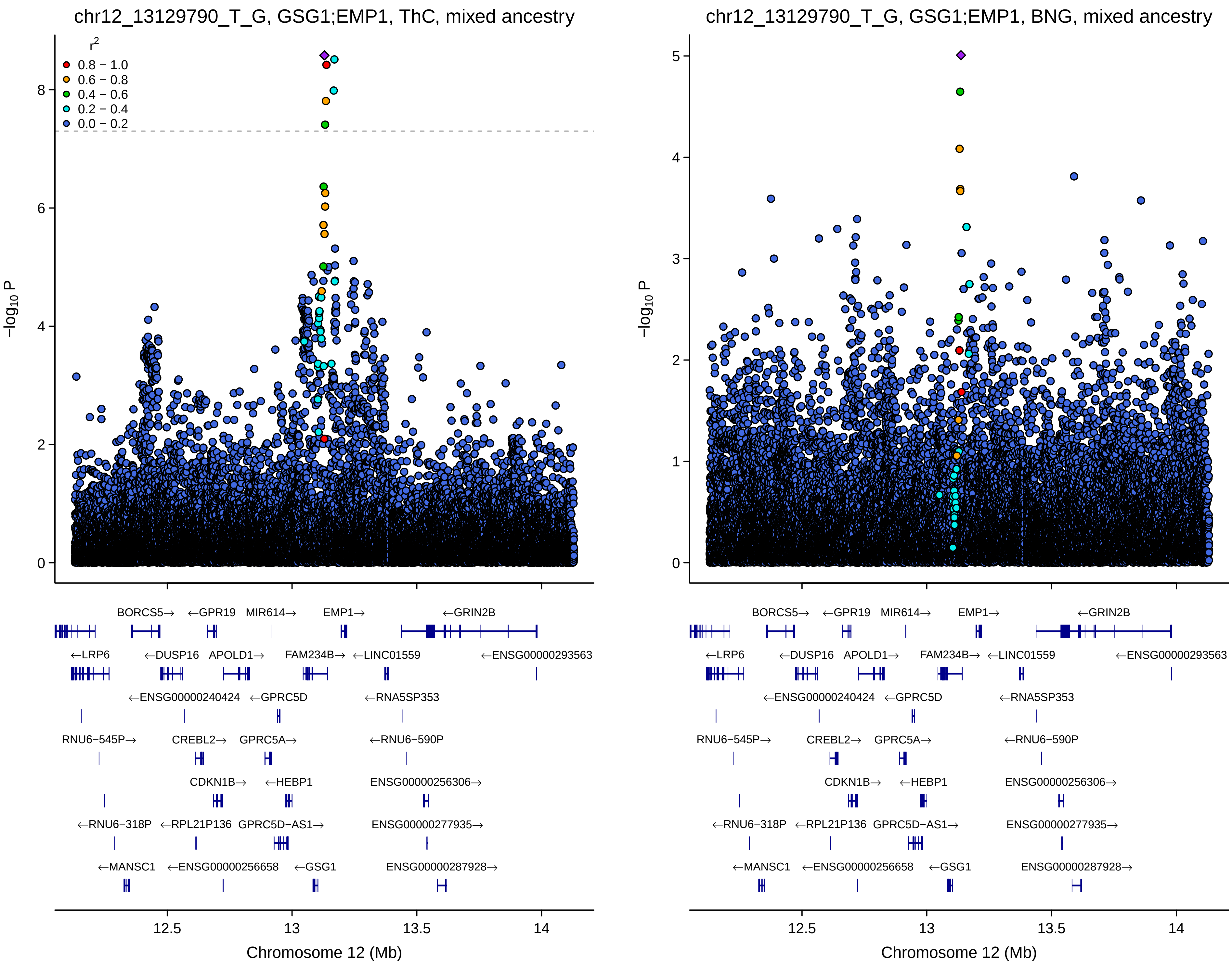

Supplementary Figure 2.1

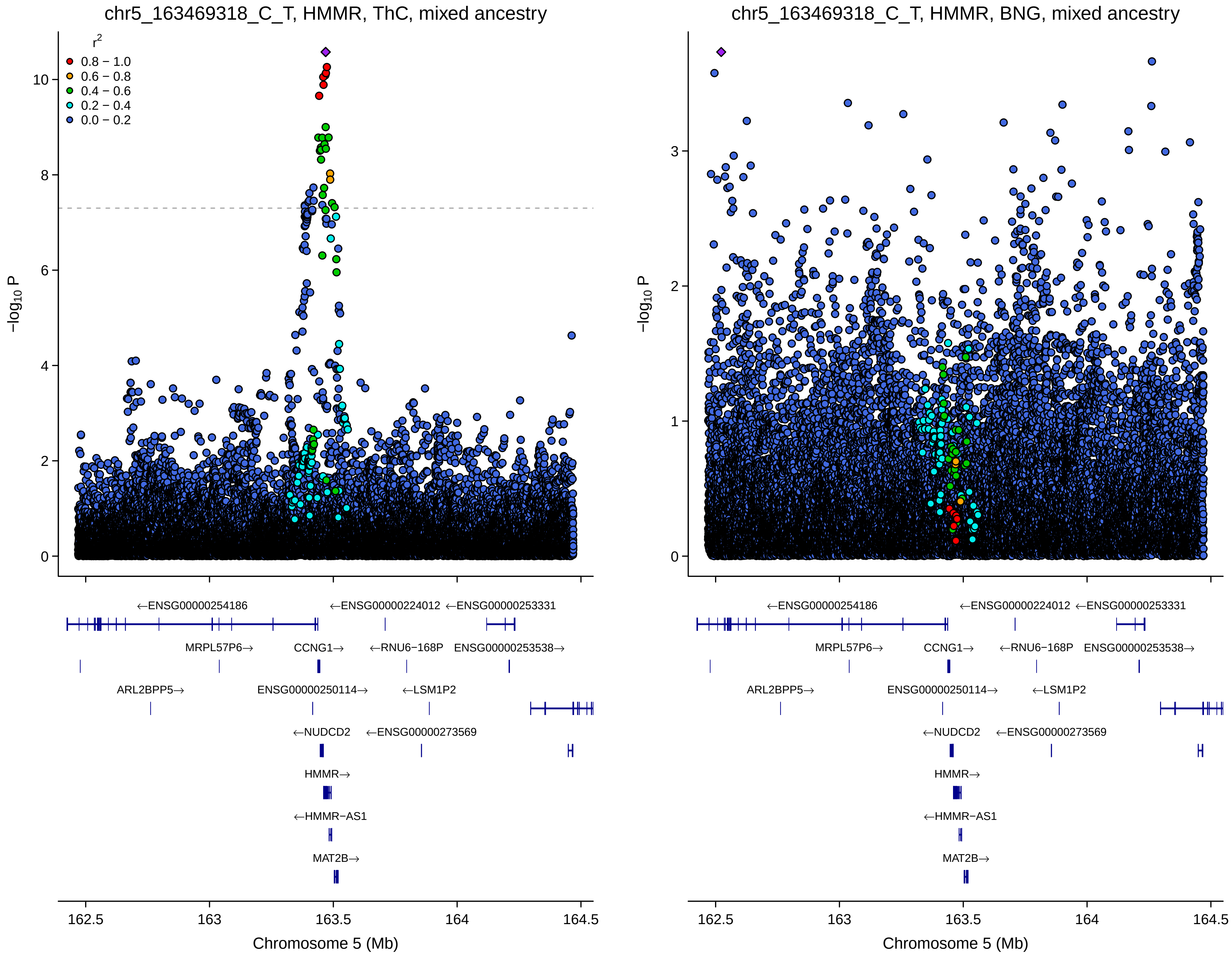

Supplementary Figure 2.1

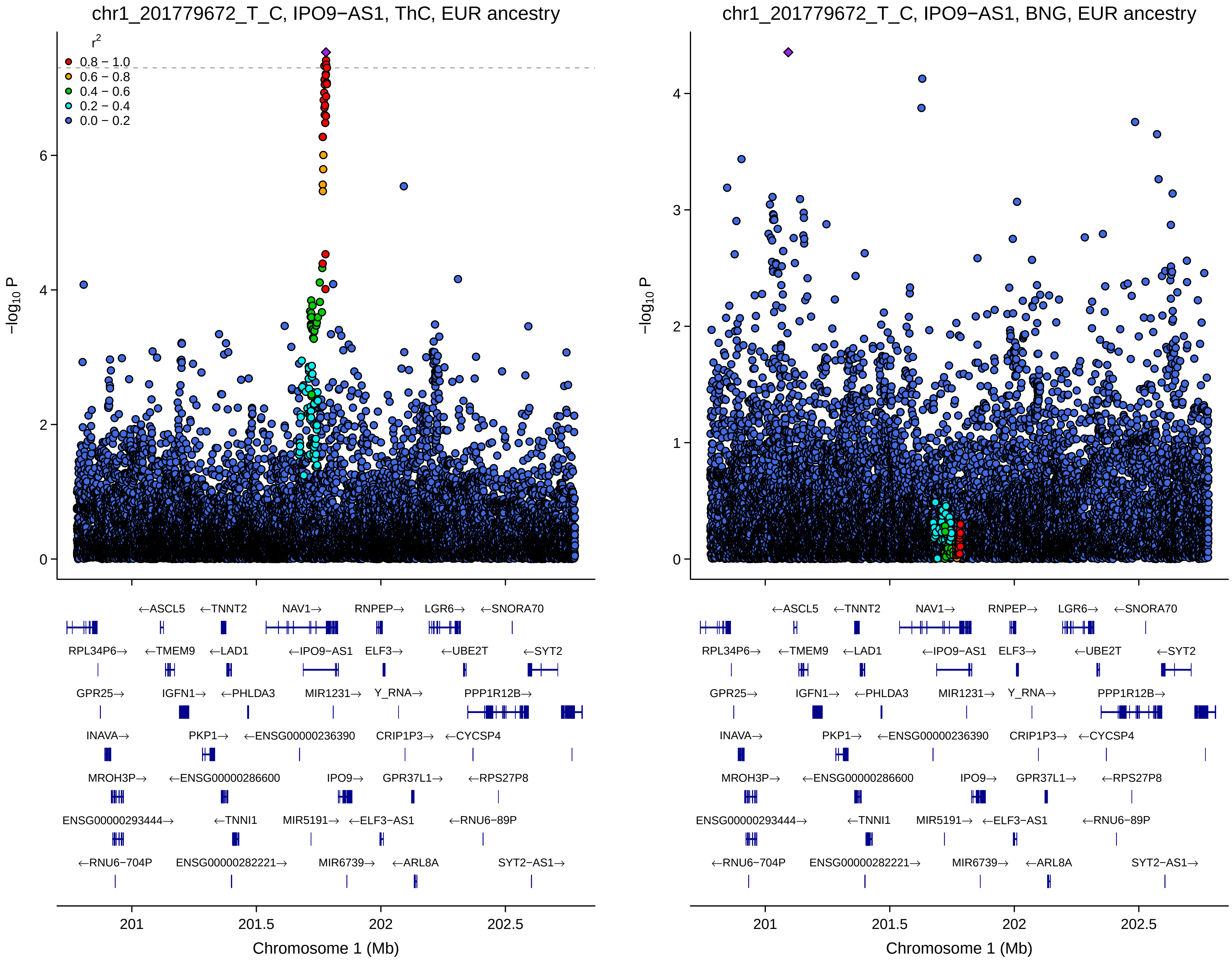

Supplementary Figure 2.1

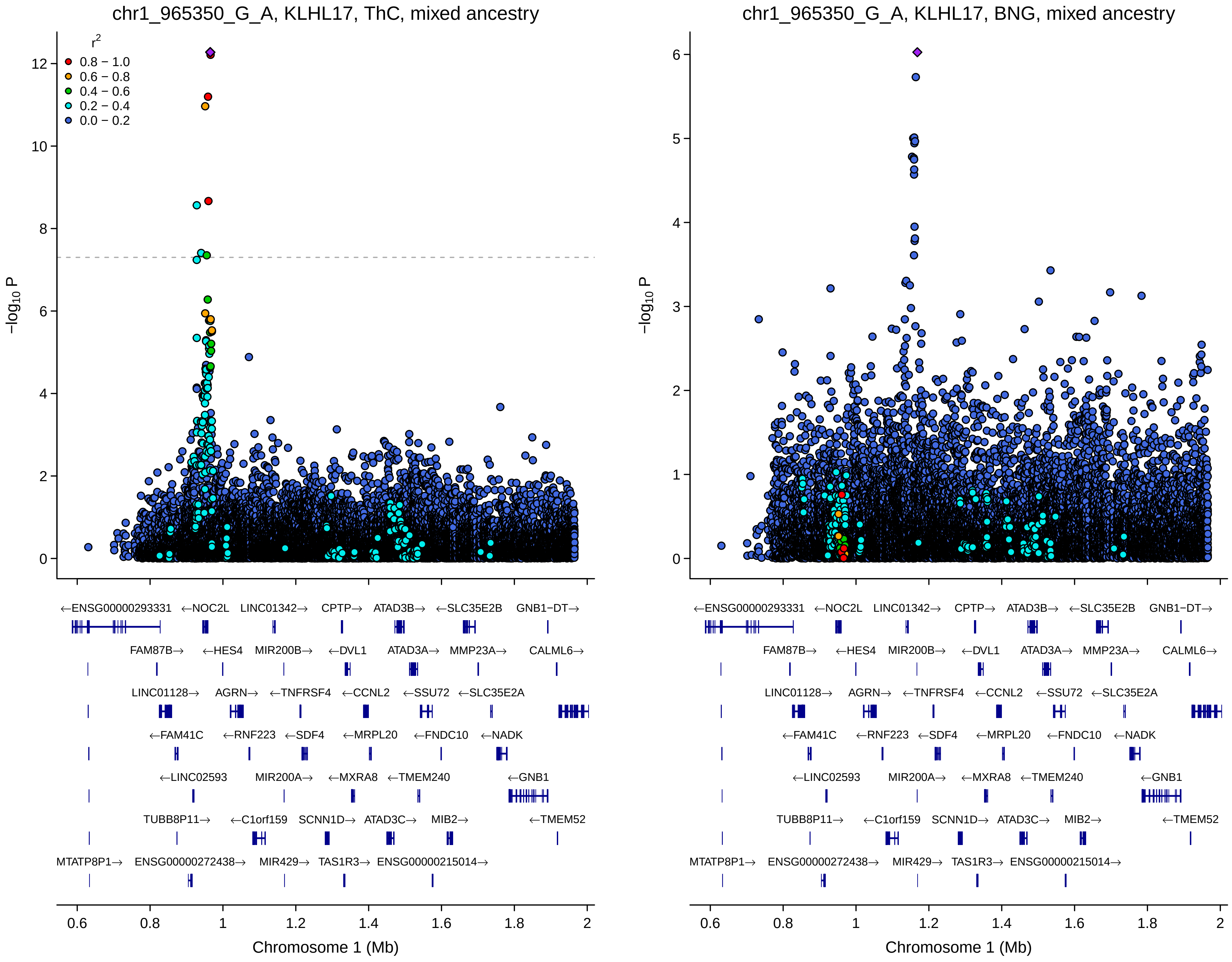

Supplementary Figure 2.1

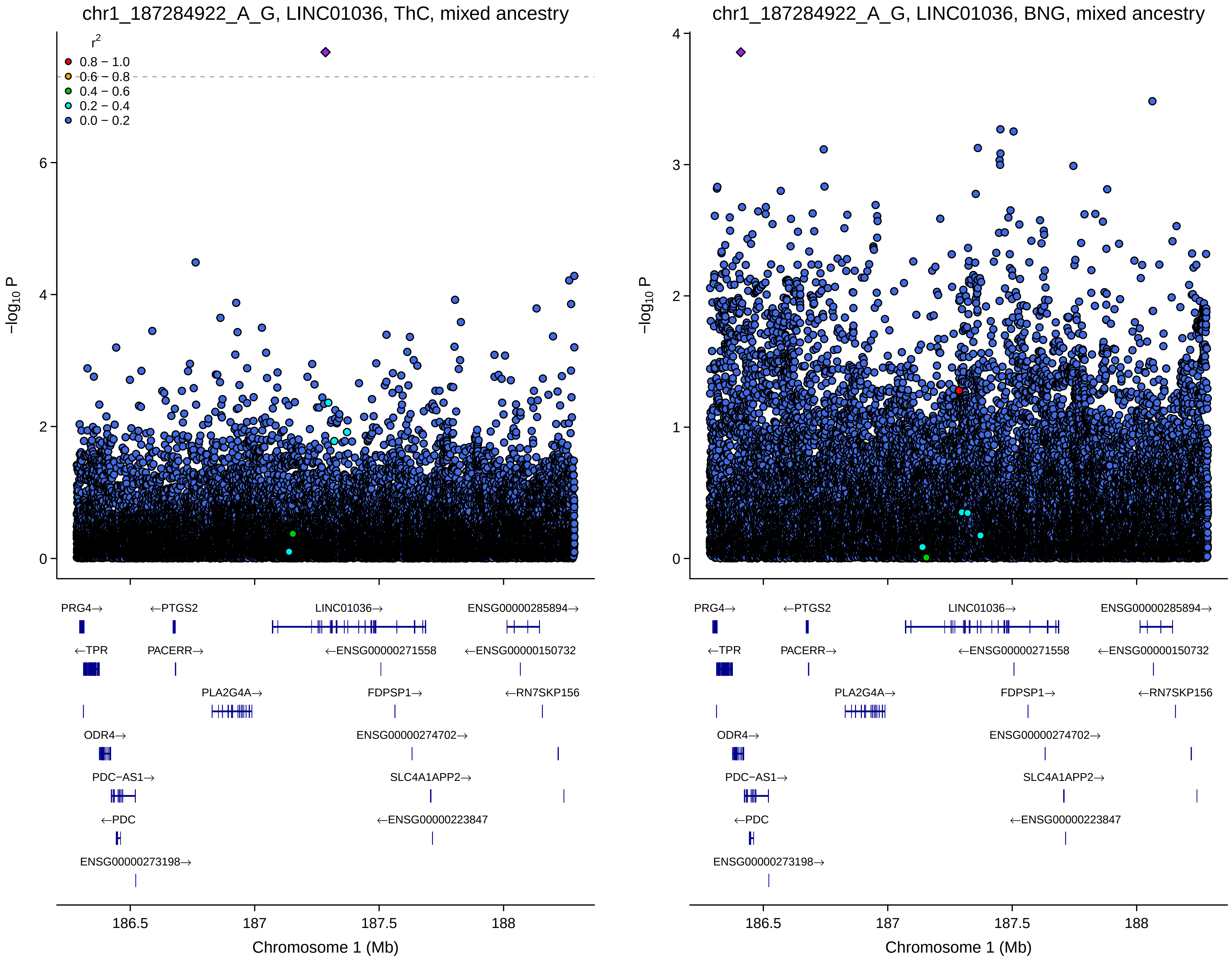

Supplementary Figure 2.1

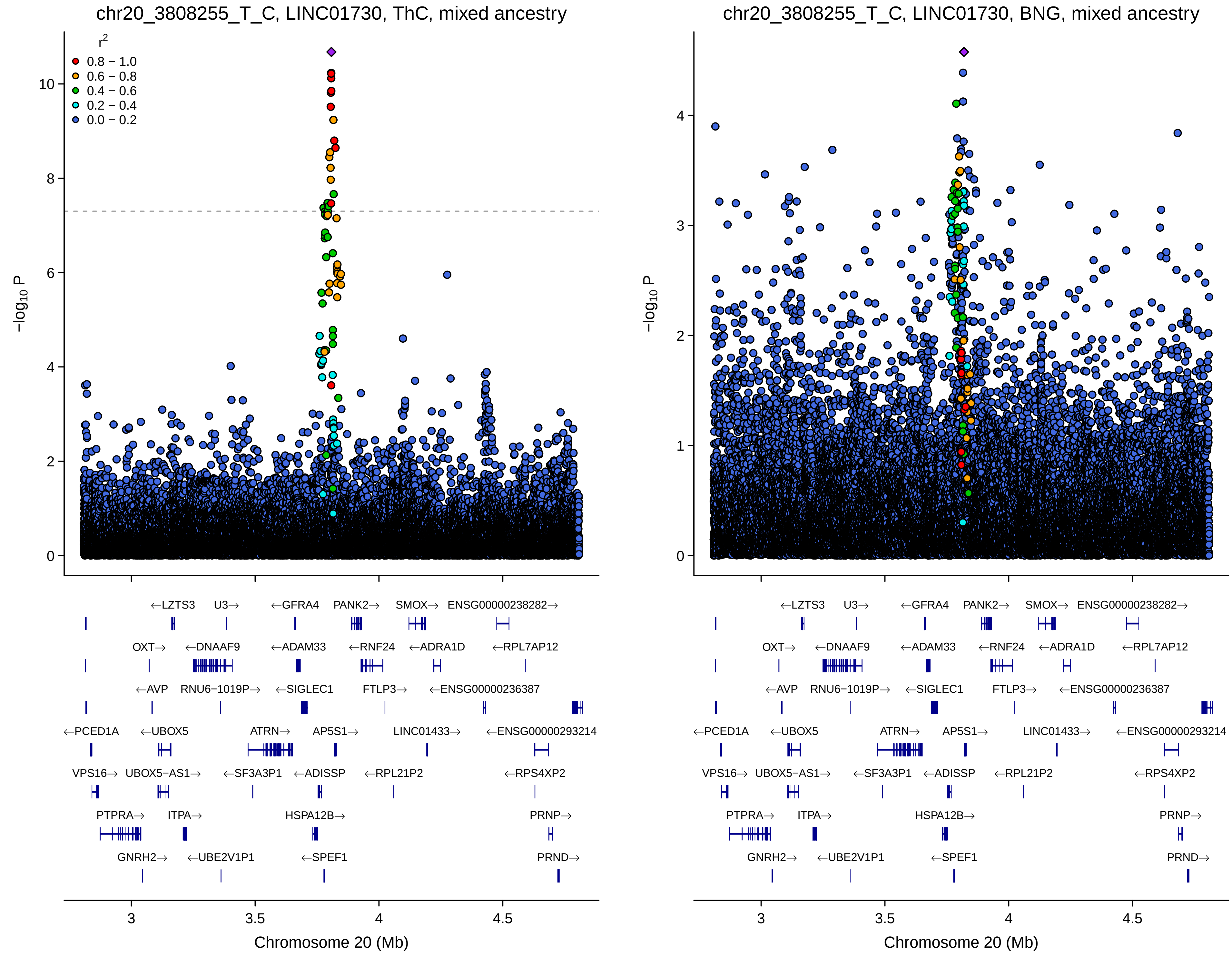

Supplementary Figure 2.1

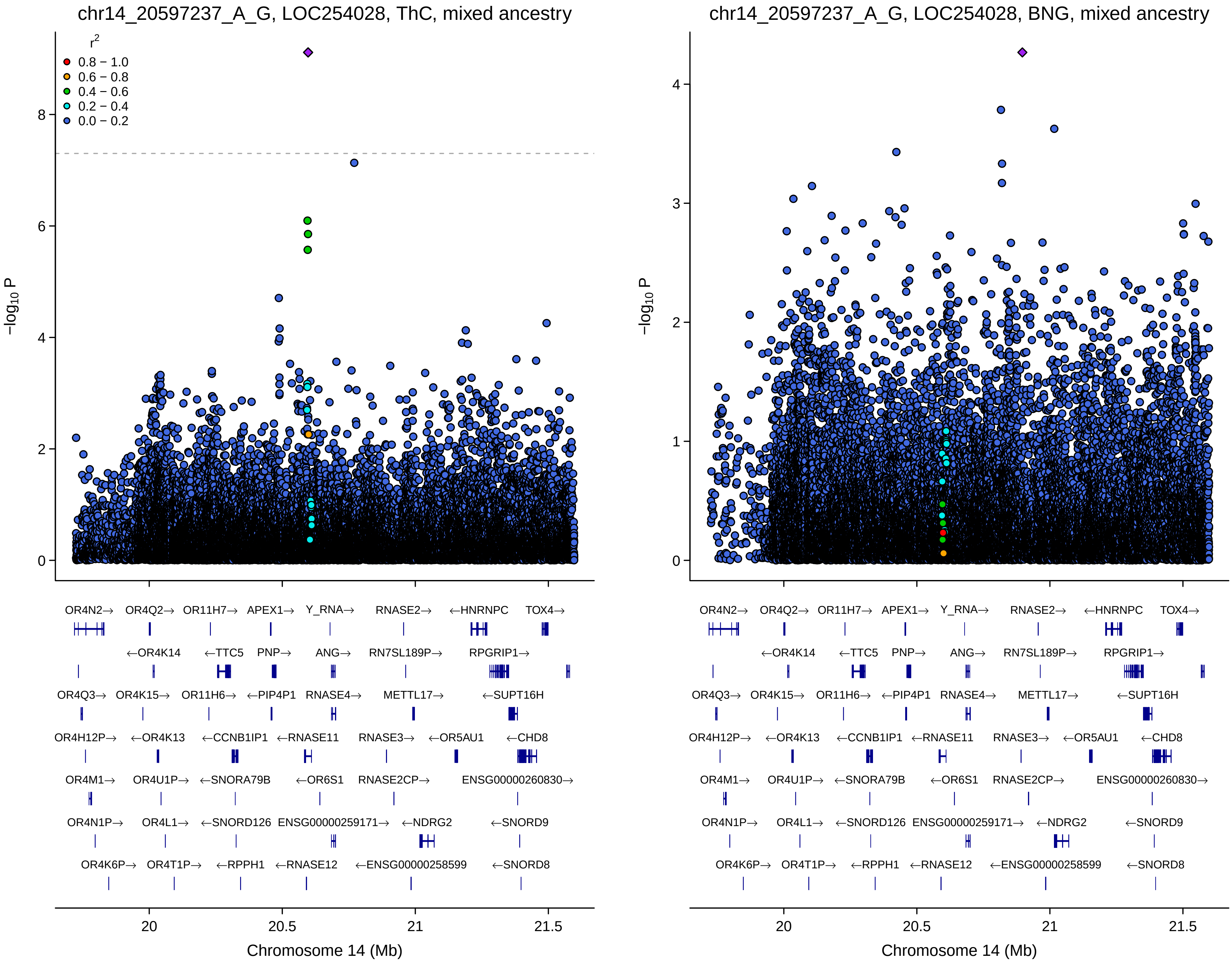

Supplementary Figure 2.1

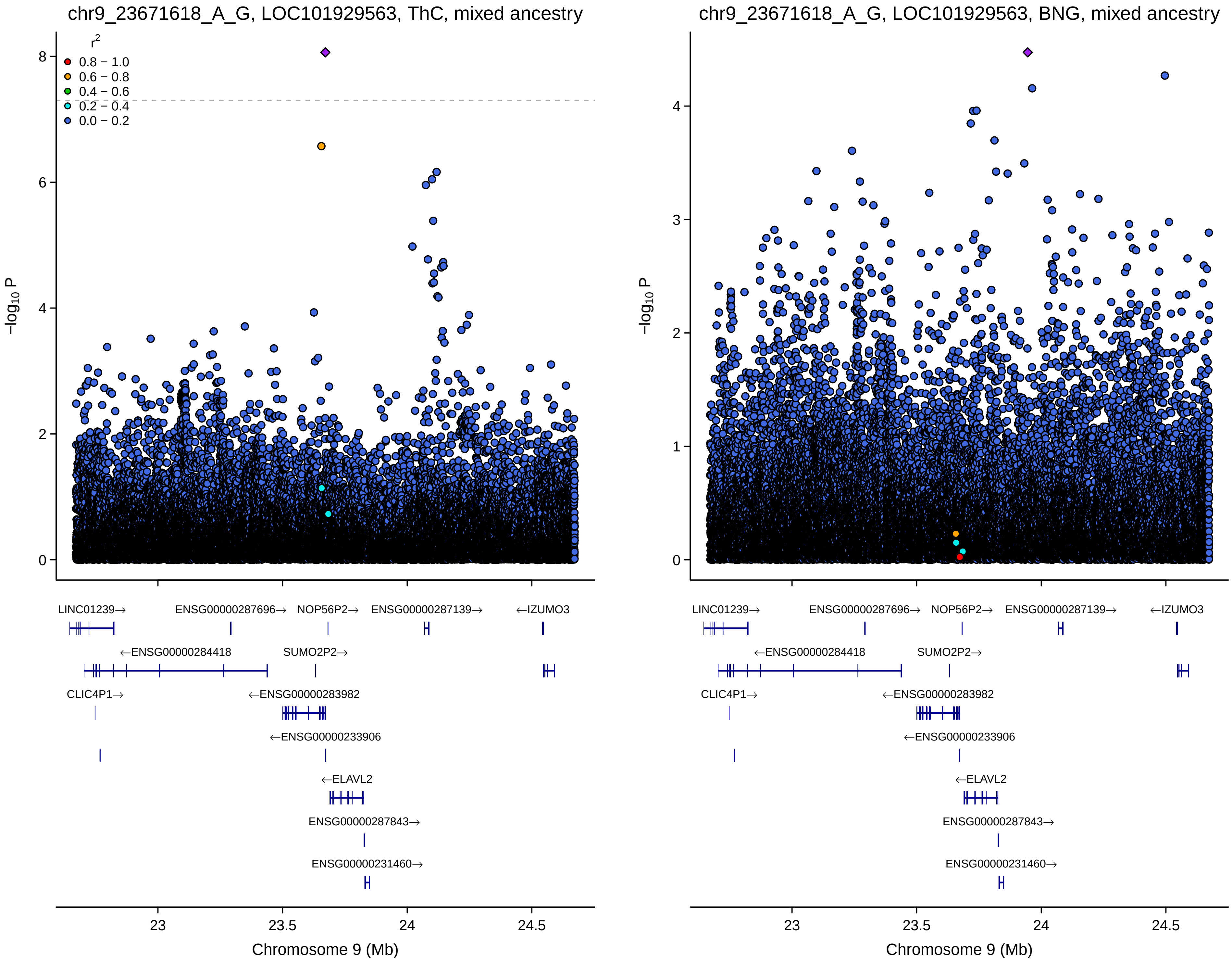

Supplementary Figure 2.1

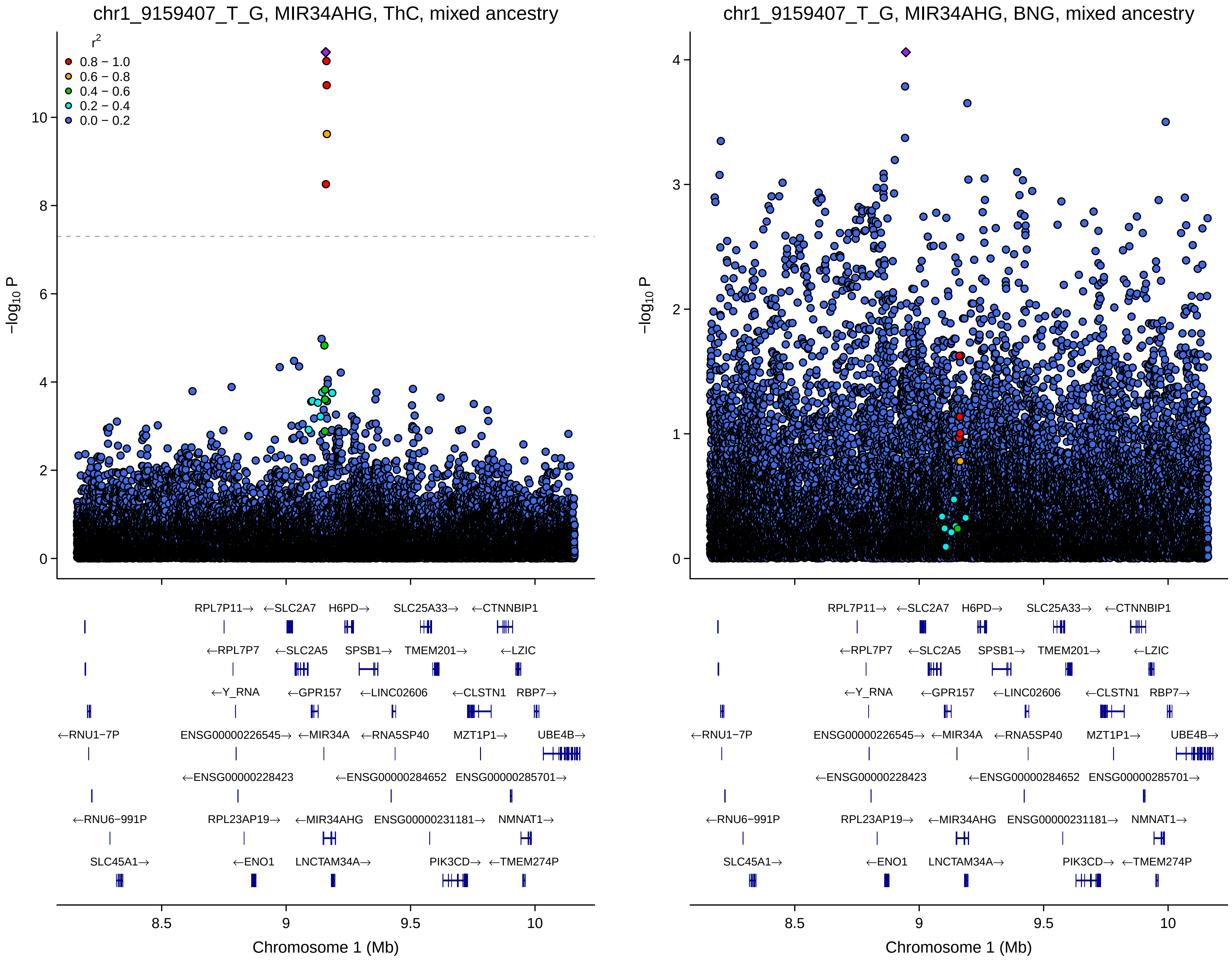

Supplementary Figure 2.1

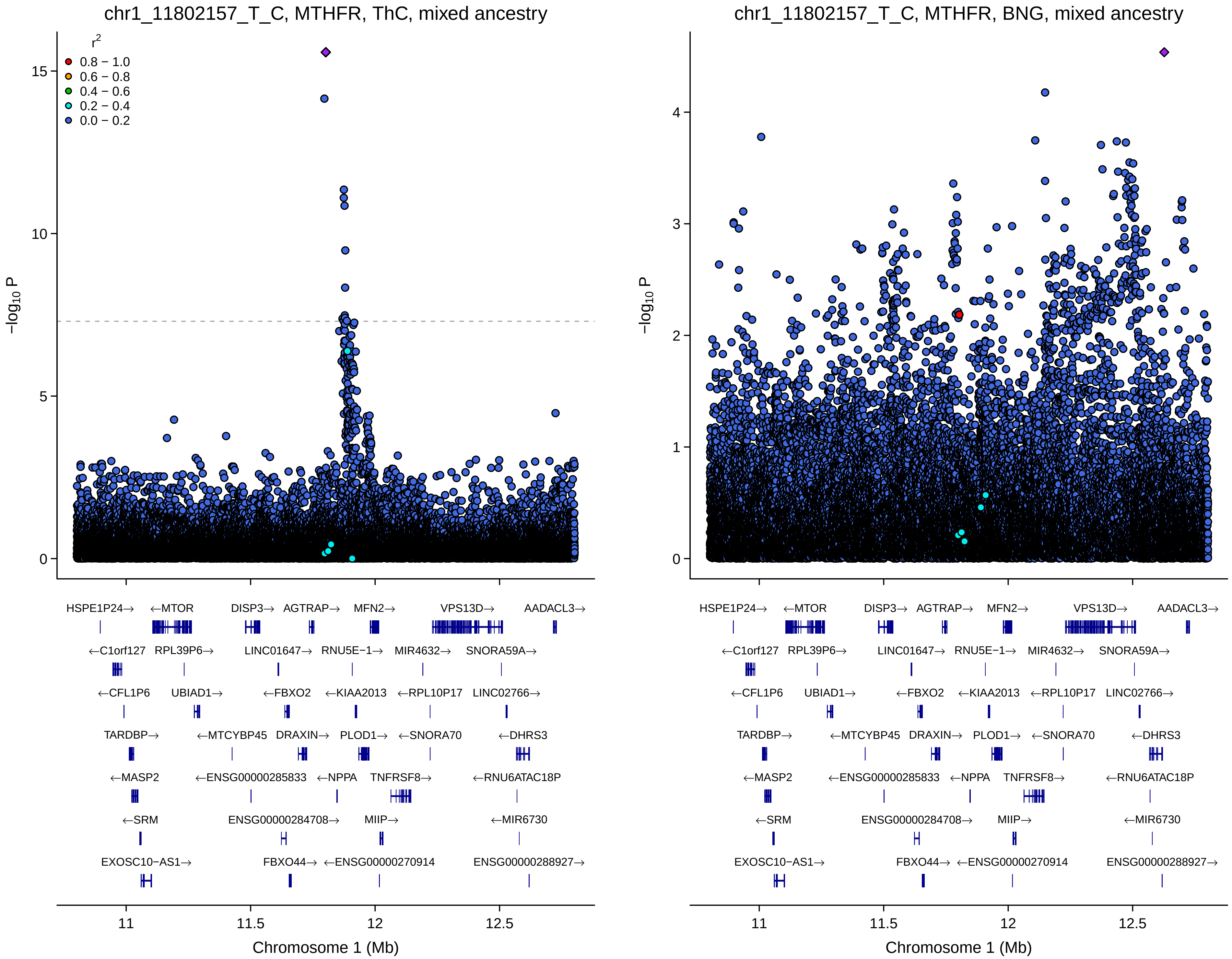

Supplementary Figure 2.1

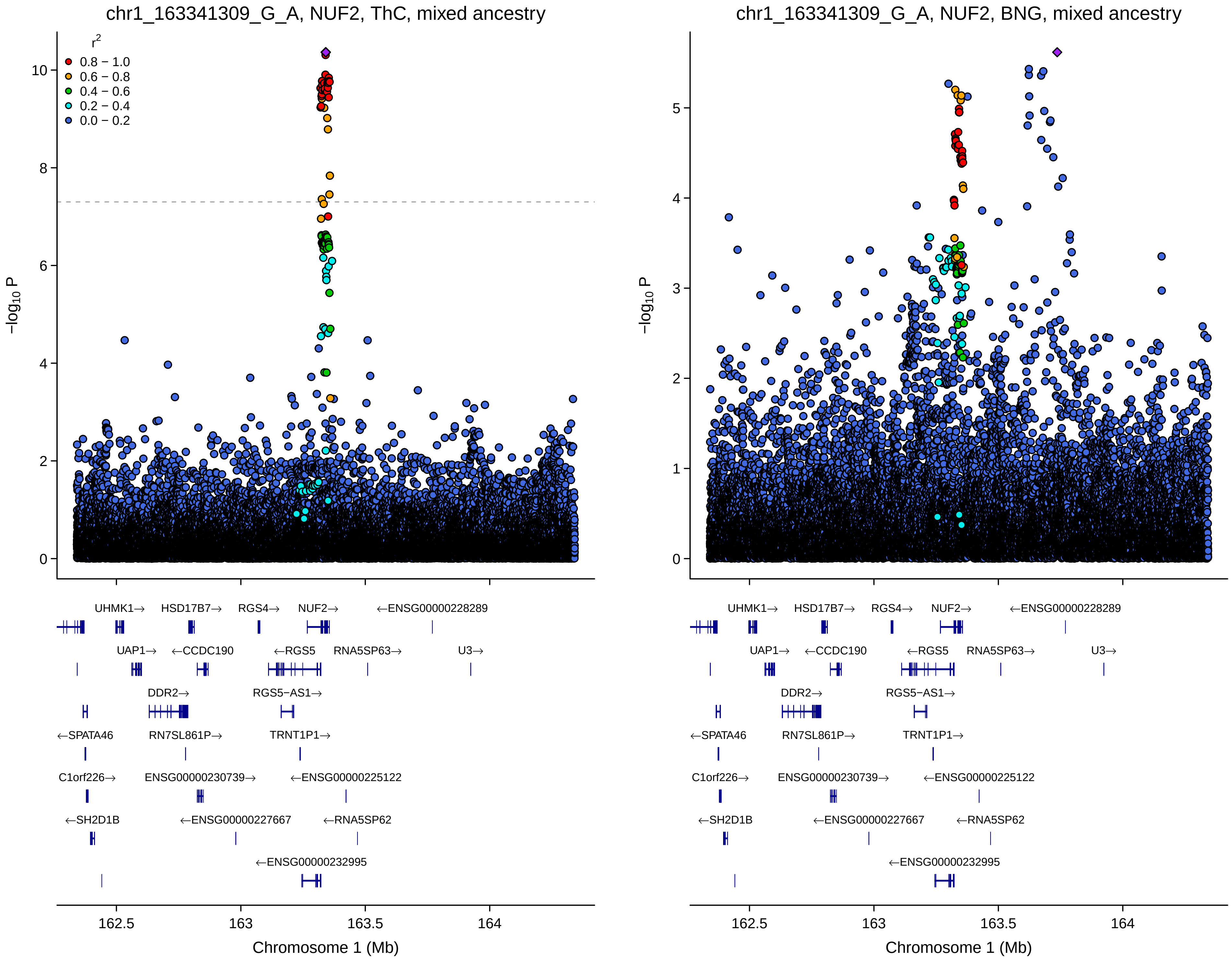

Supplementary Figure 2.1

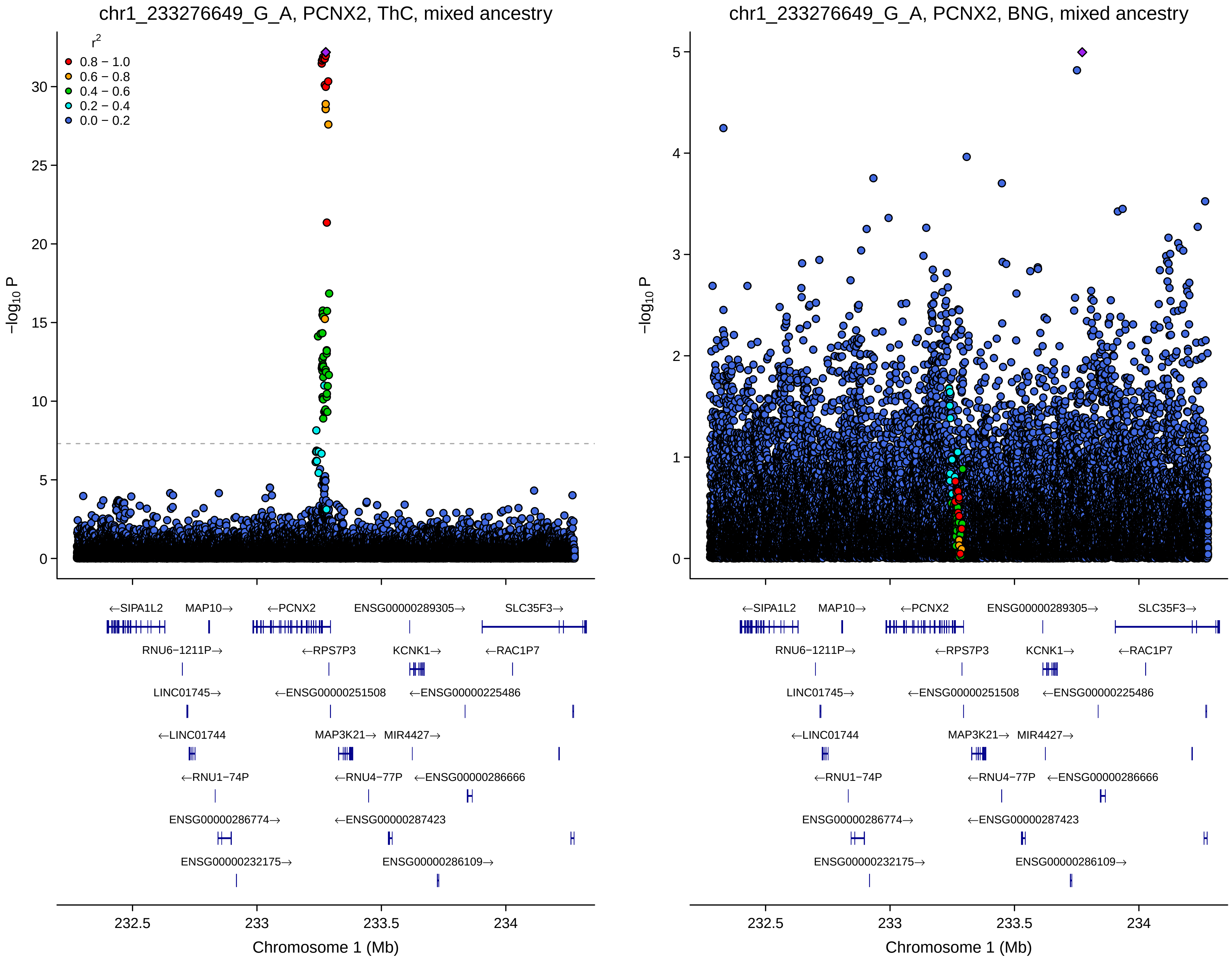

Supplementary Figure 2.1

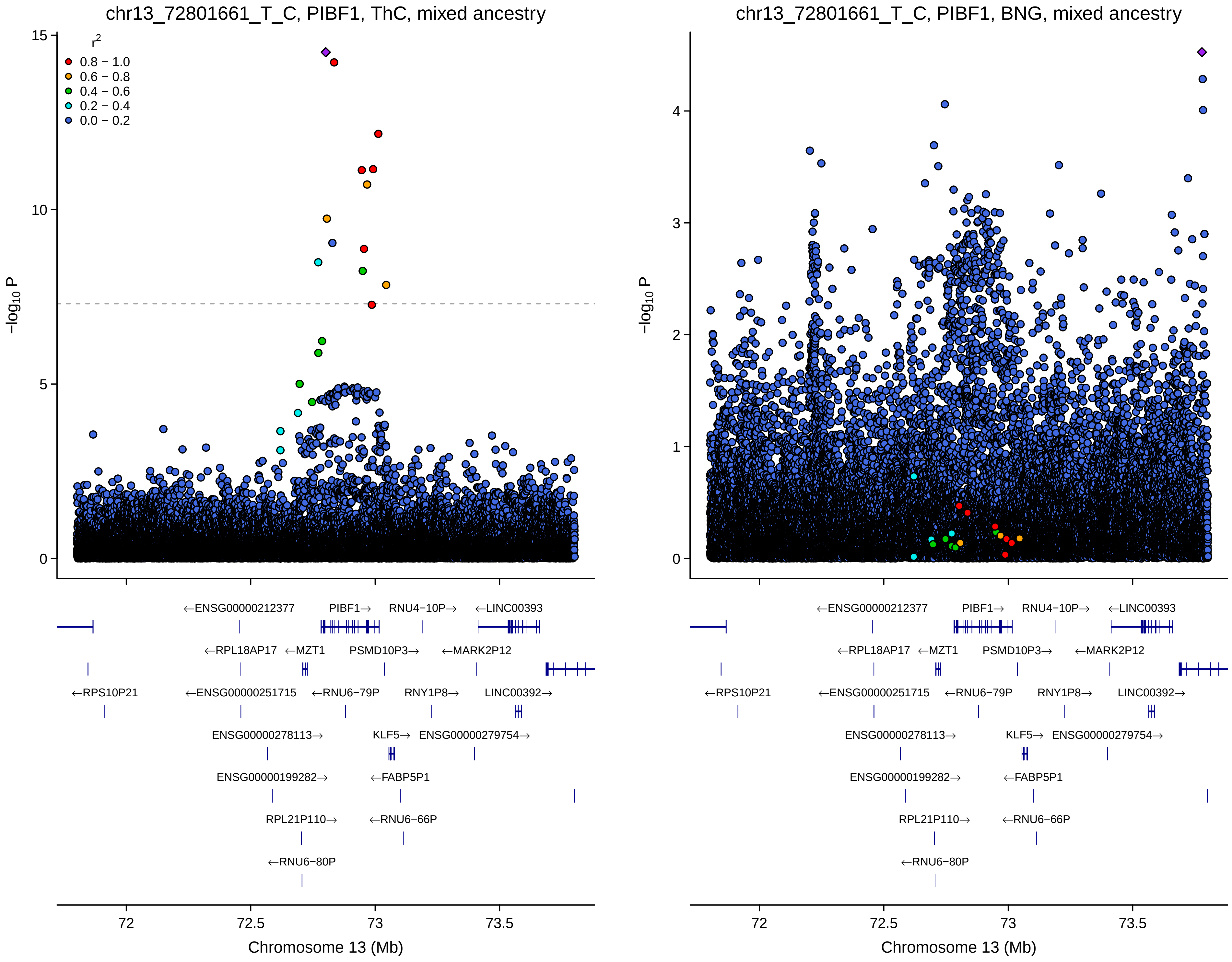

Supplementary Figure 2.1

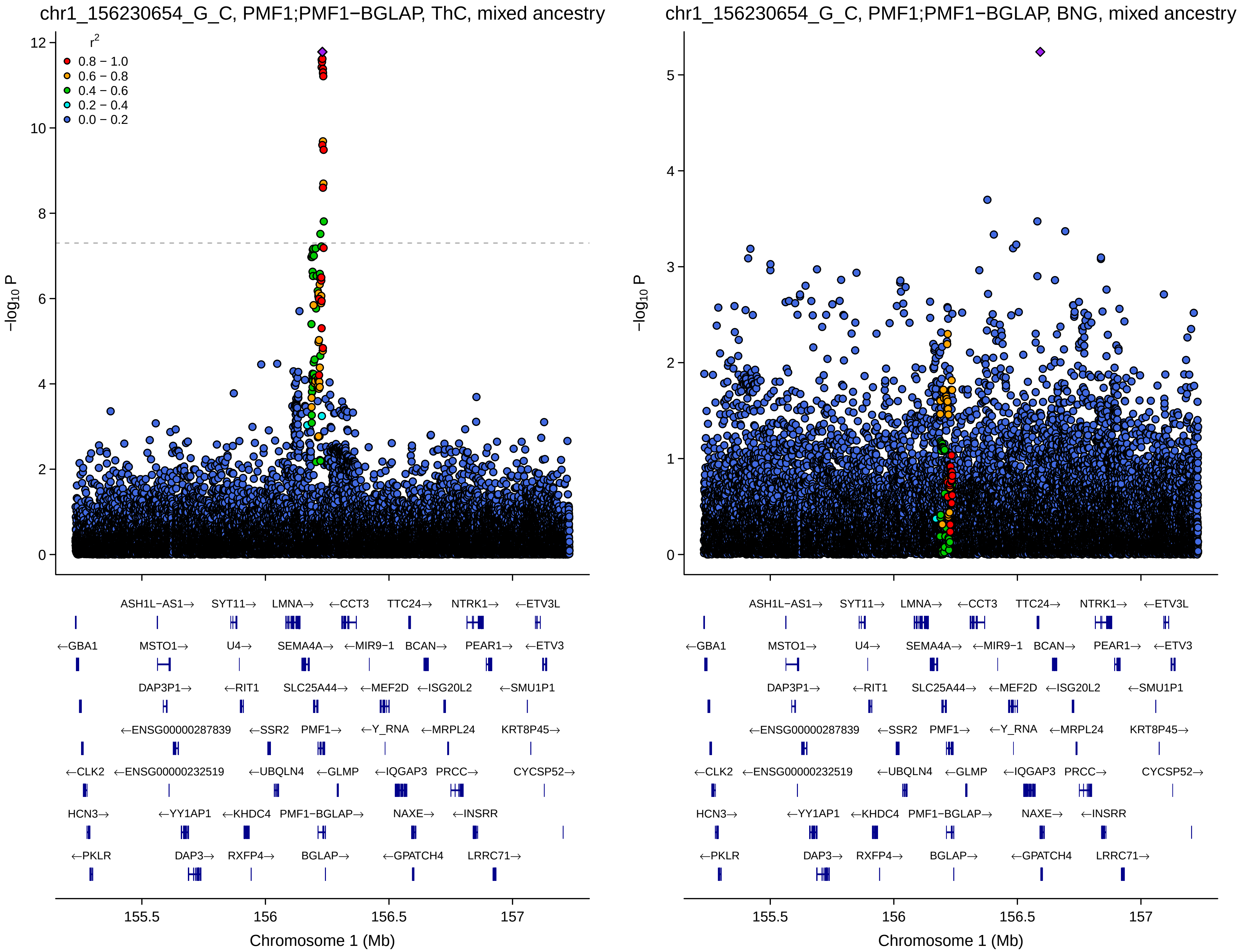

Supplementary Figure 2.1

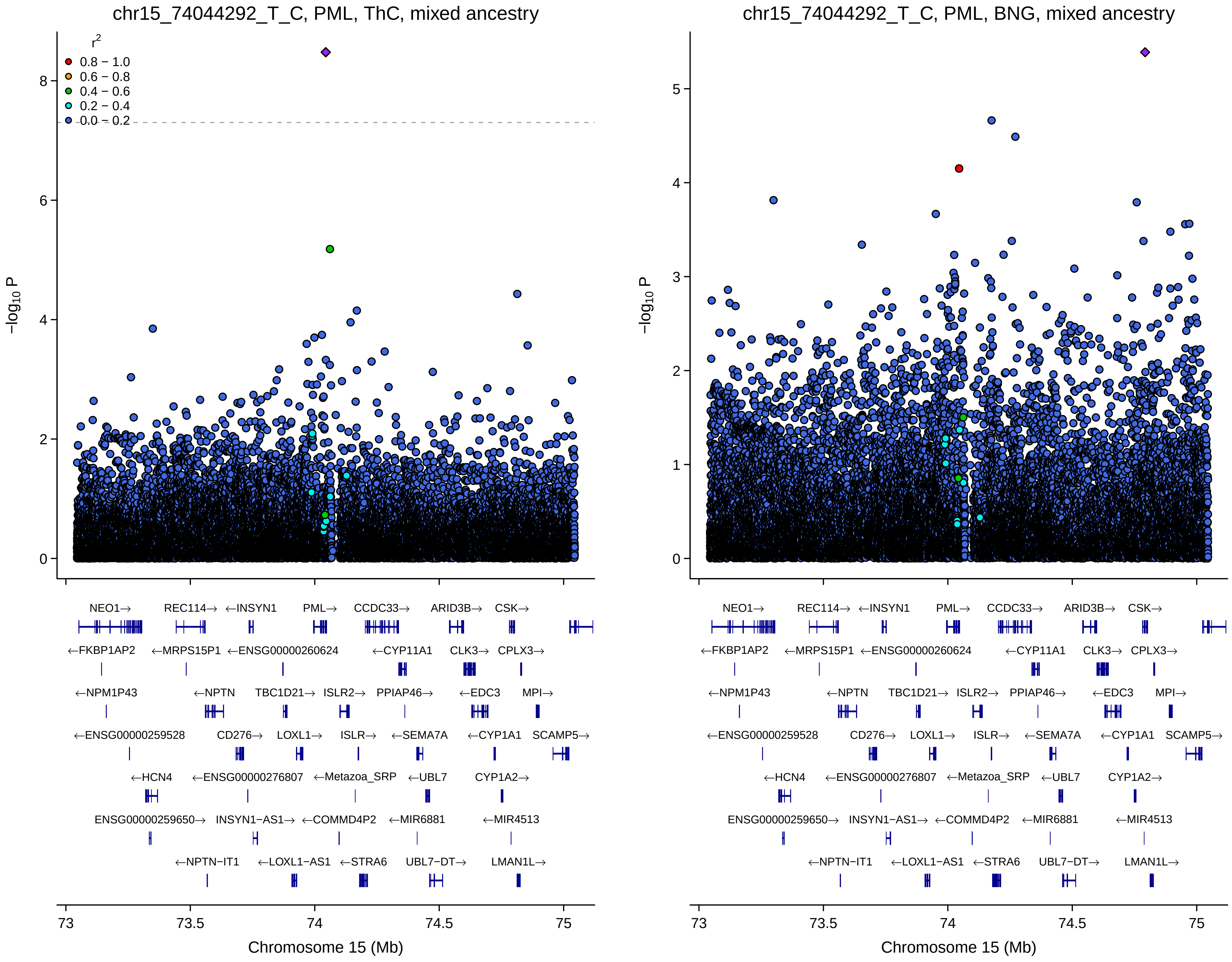

Supplementary Figure 2.1

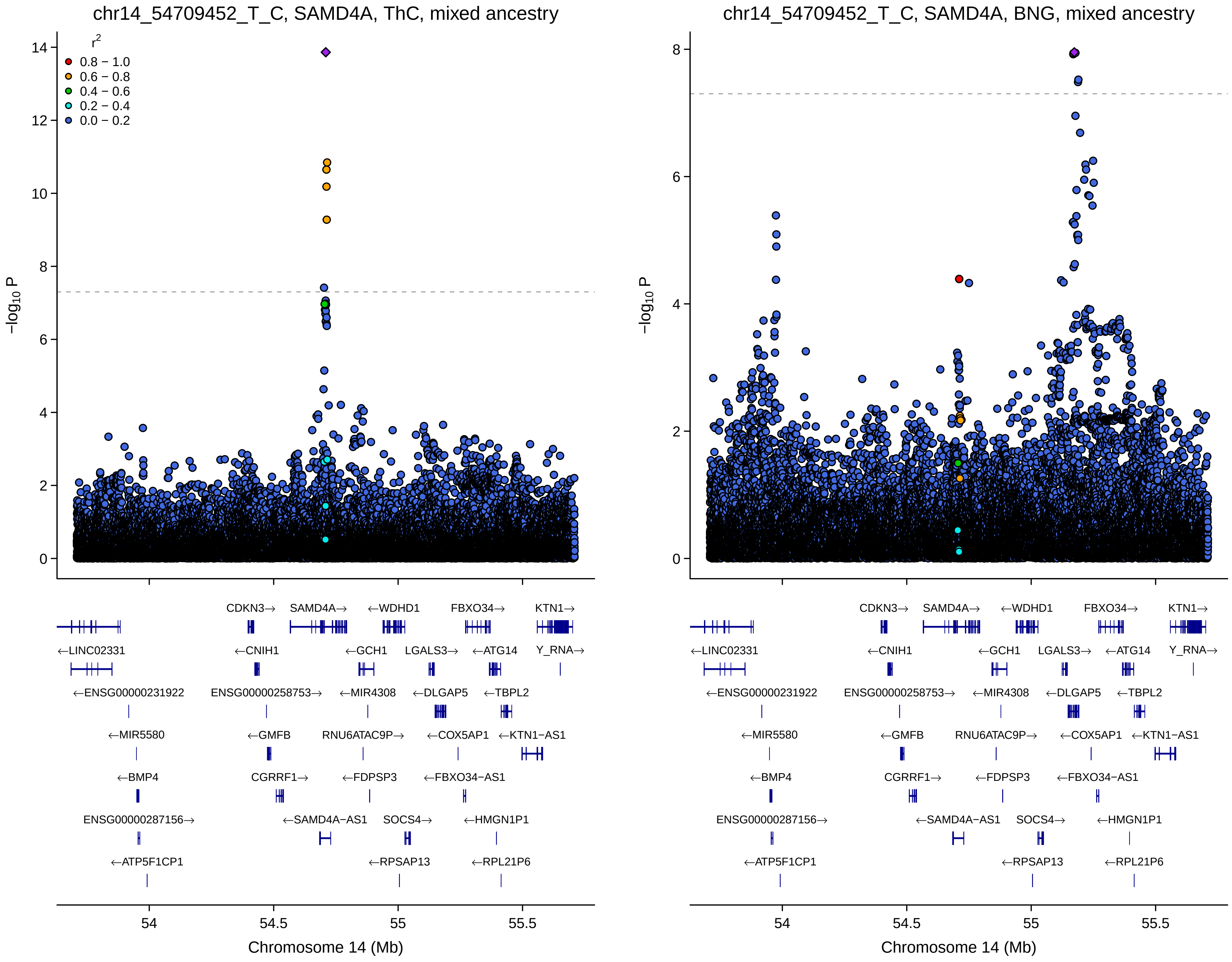

Supplementary Figure 2.1

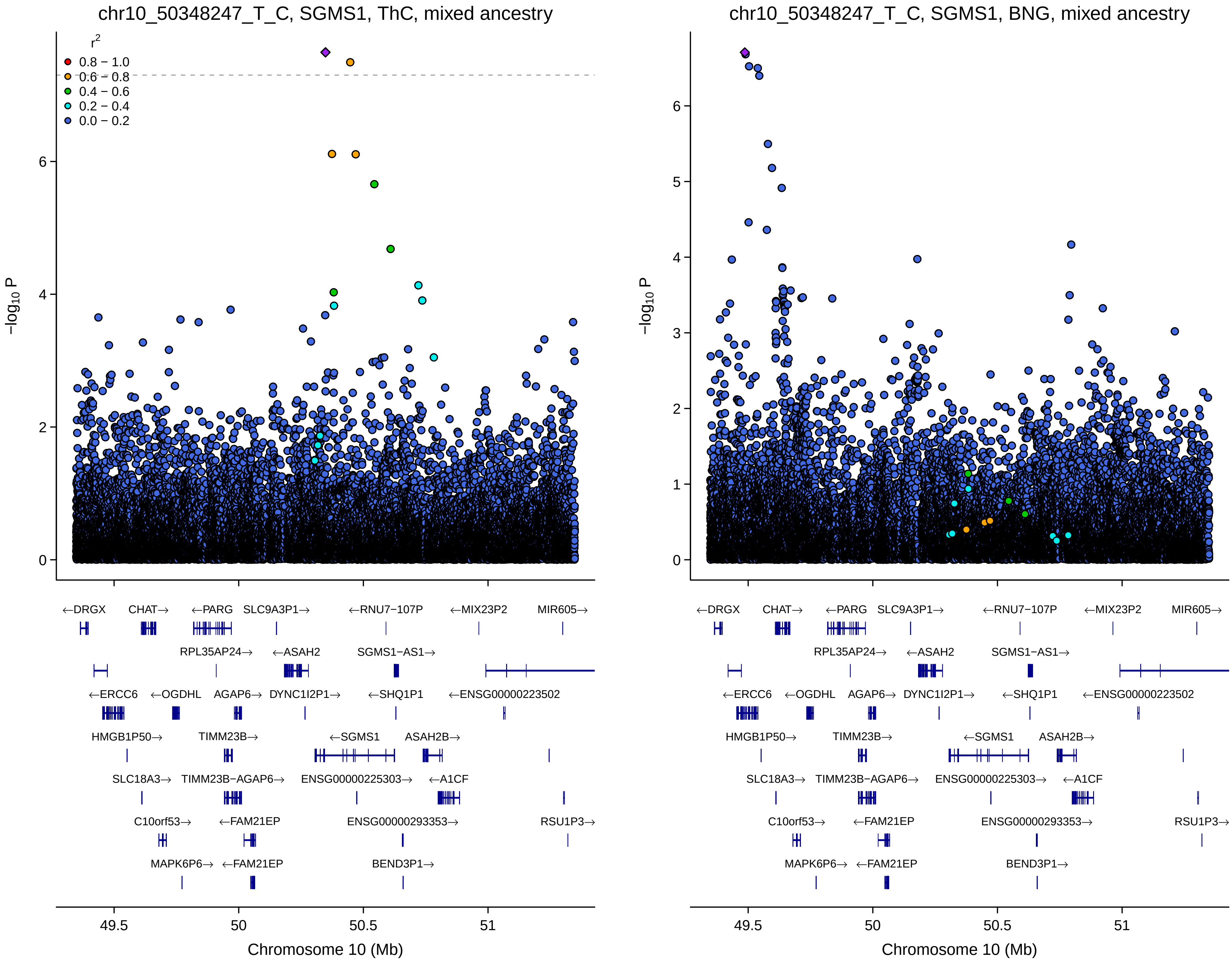

Supplementary Figure 2.1

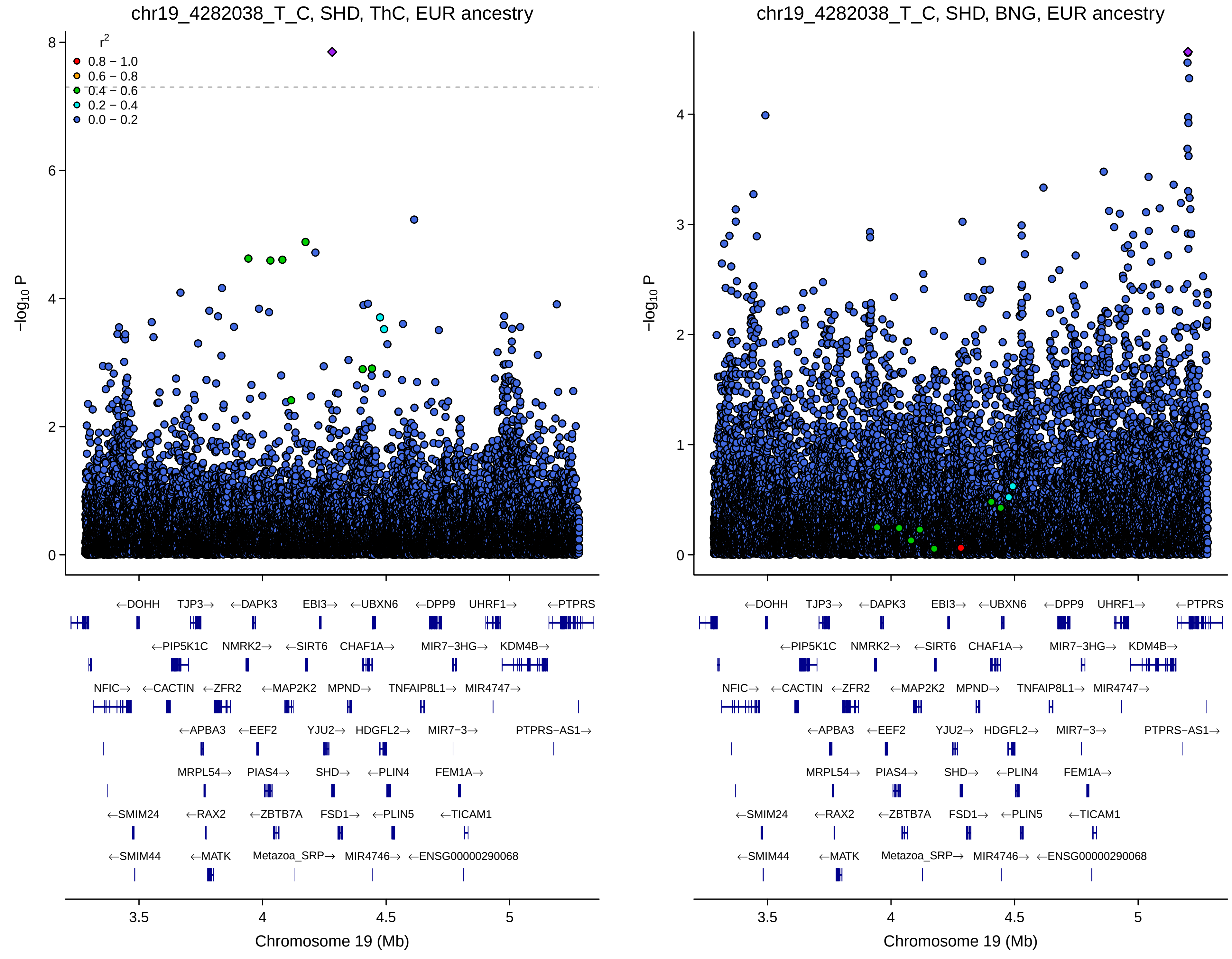

Supplementary Figure 2.1

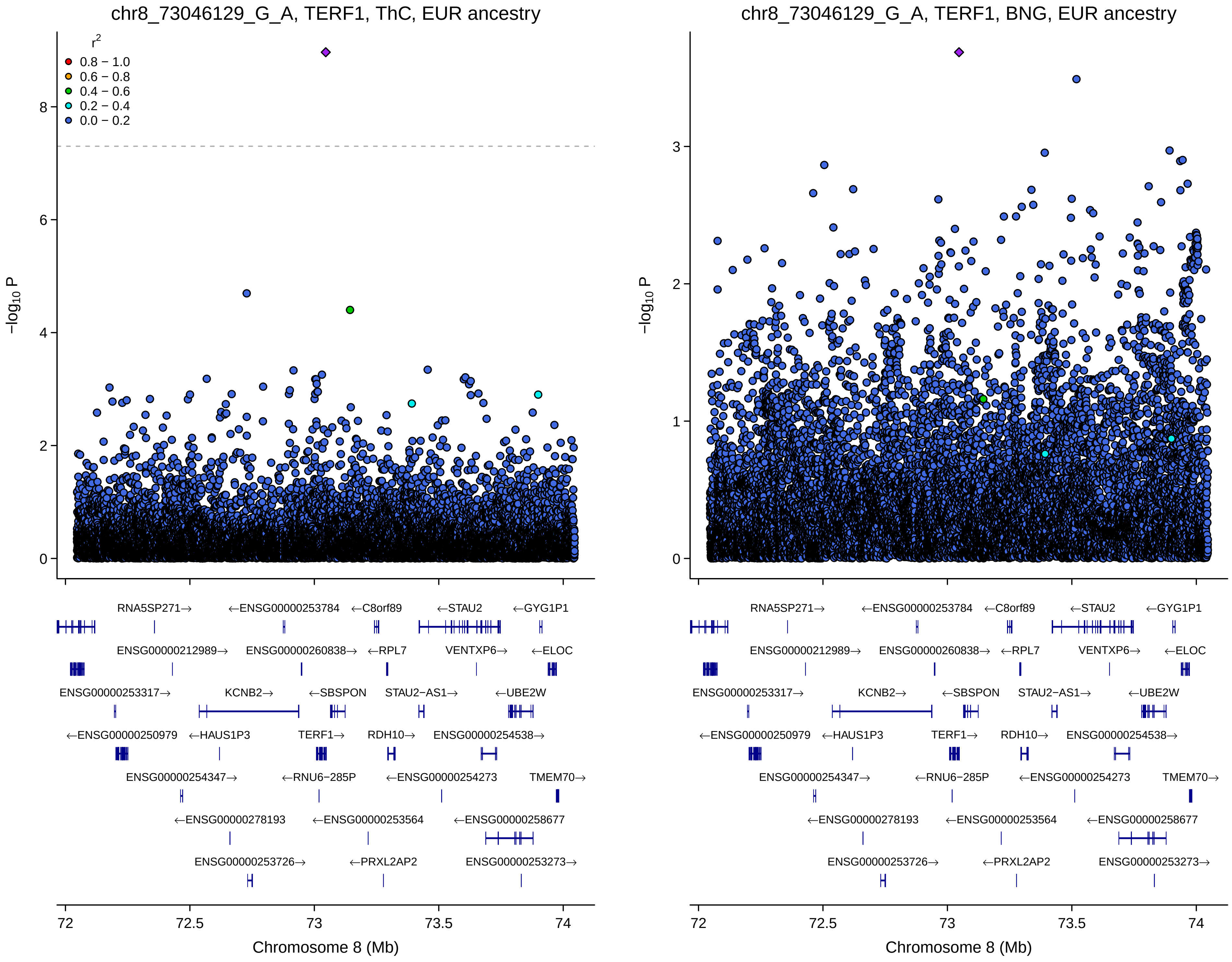

Supplementary Figure 2.1

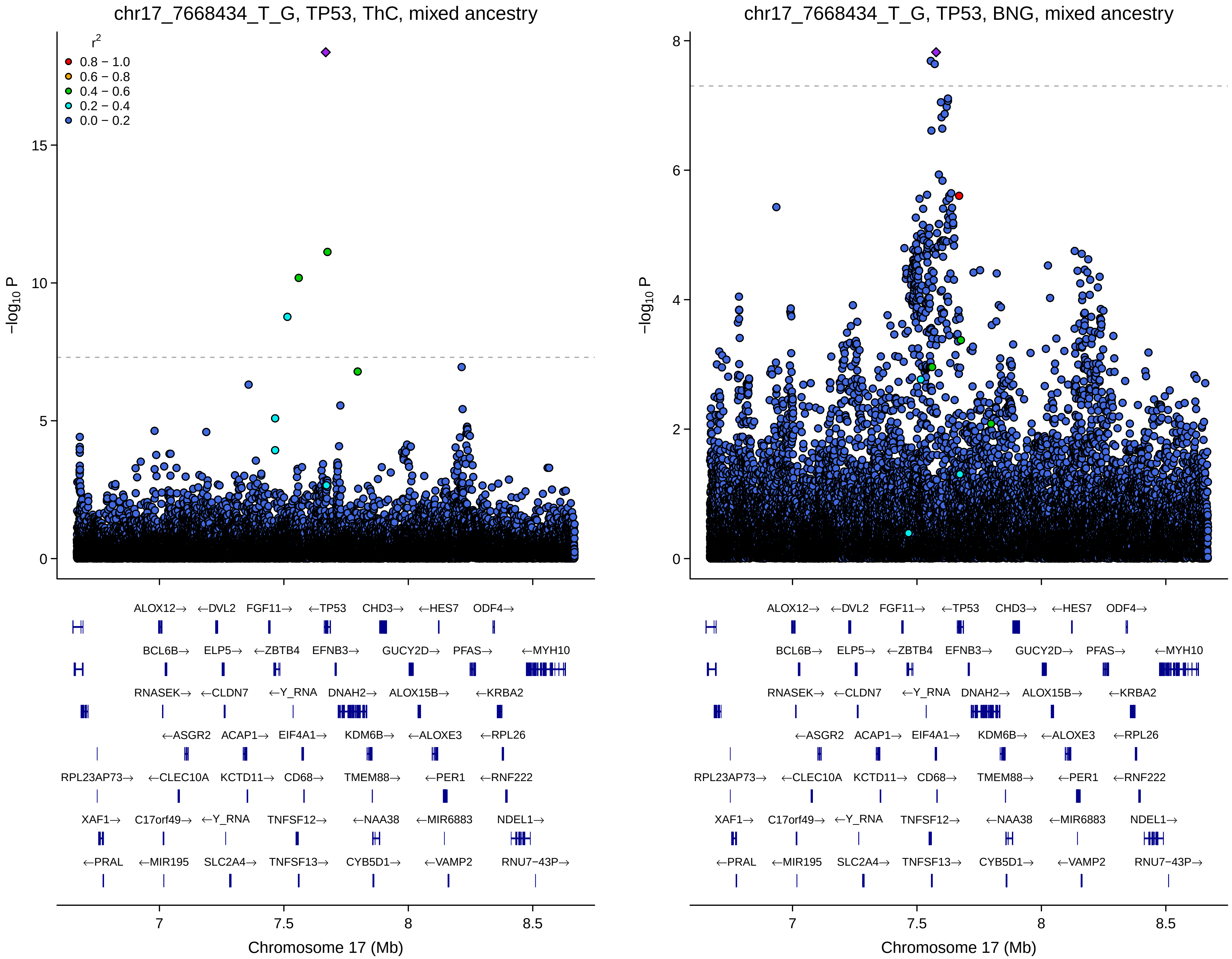

Supplementary Figure 2.1

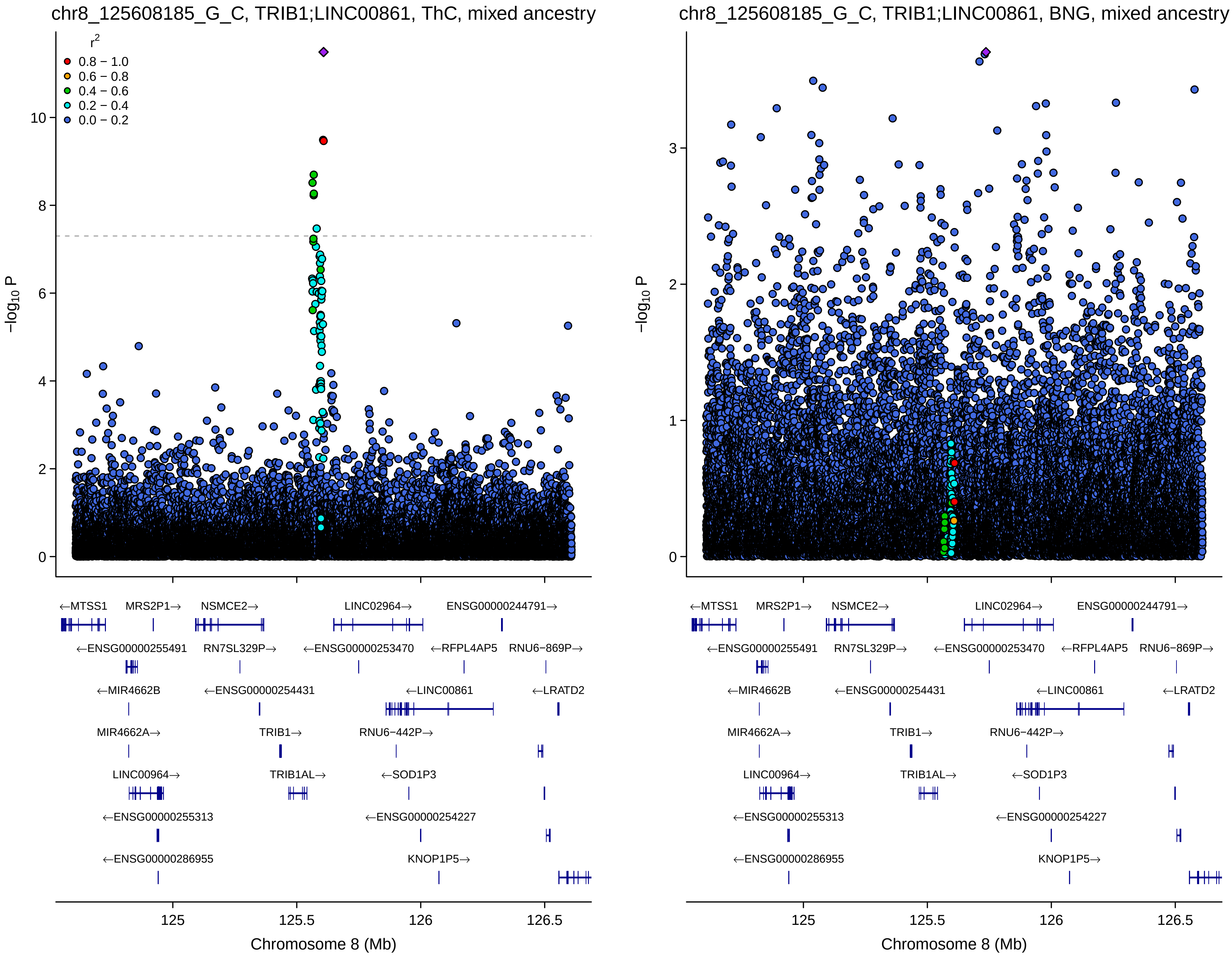

Supplementary Figure 2.1

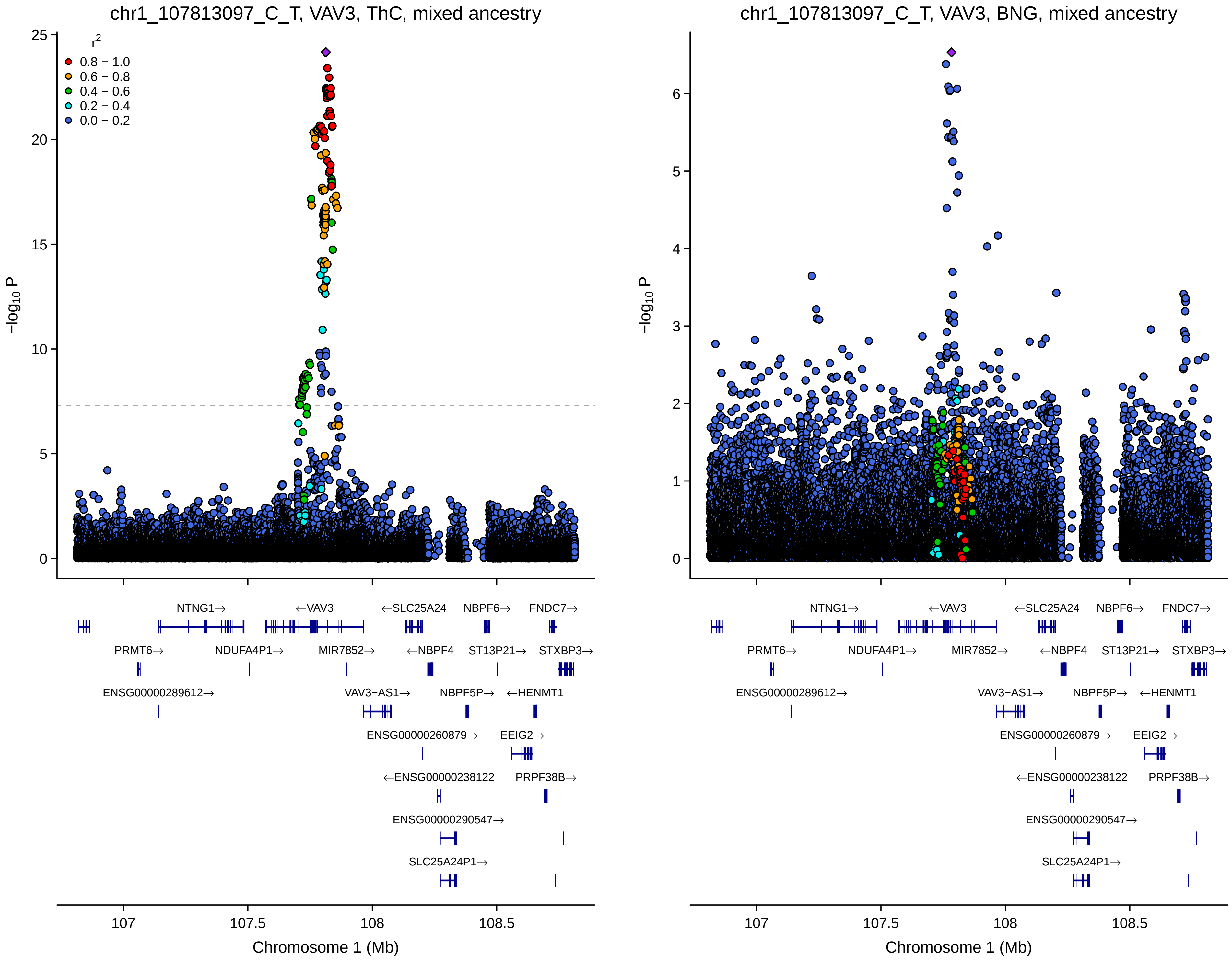

Supplementary Figure 2.1

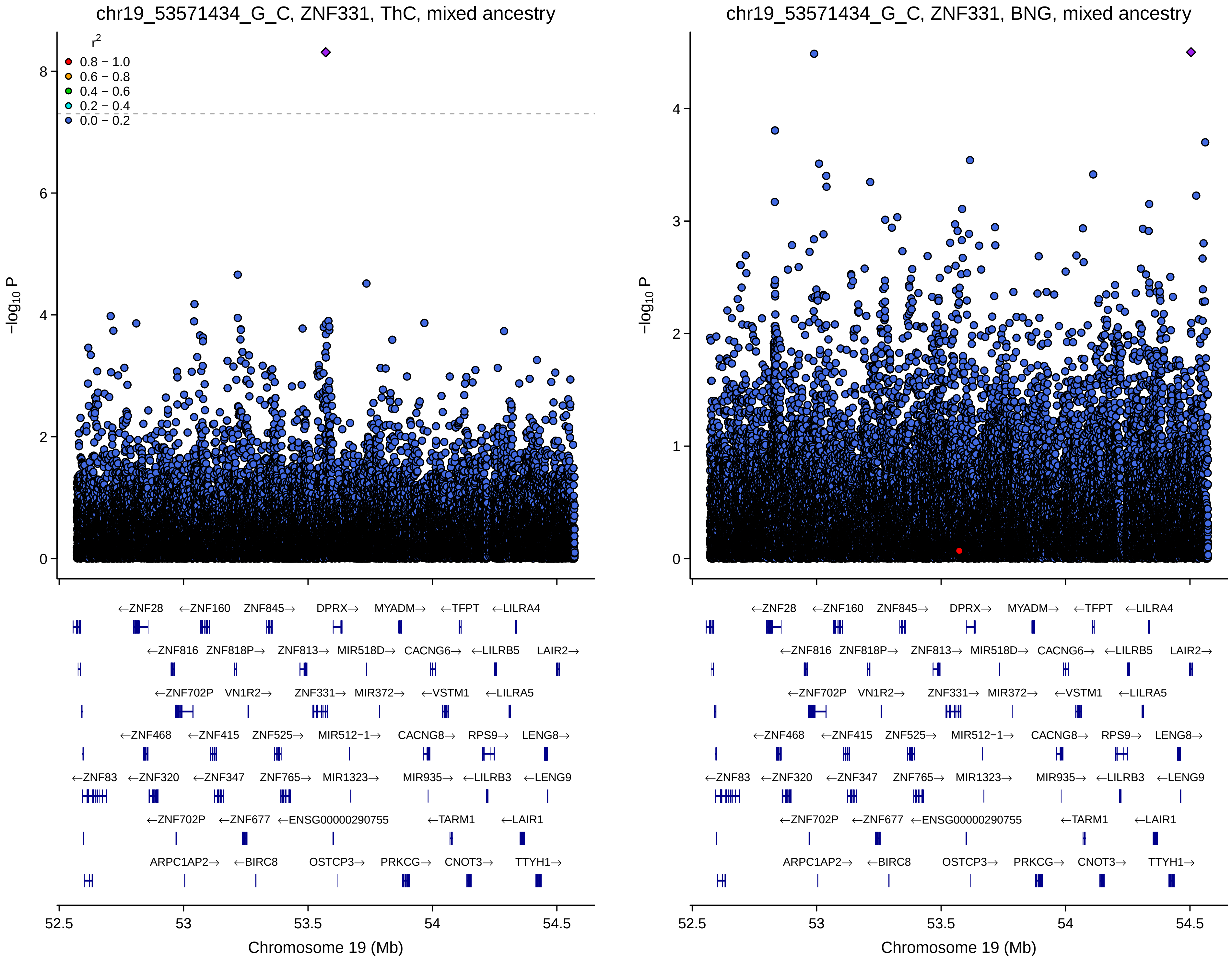

Supplementary Figure 2.2. Locus plots for loci significantly associated with benign nodular goiter but not thyroid cancer. The left plot displays -log10(p-values) for variants from thyroid cancer meta-analysis, the right plot displays -log10(p-values) for variants within the same genomic region from the benign nodular goiter meta-analysis. The lead variant is shown with a purple diamond. Genome wide significance is indicated by the dashed horizontal line at -log10(5e-8).

Supplementary Figure 2.2

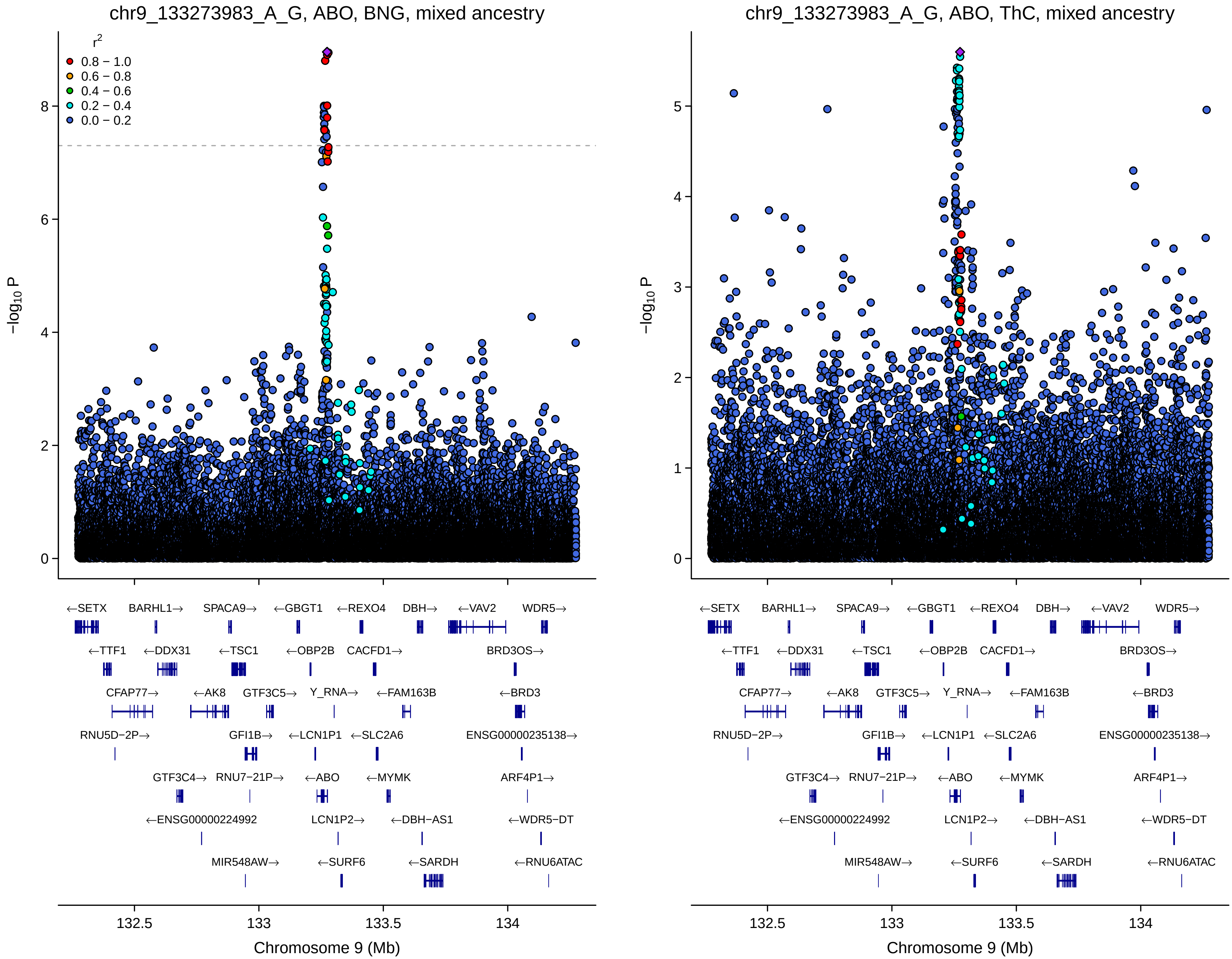

Supplementary Figure 2.2

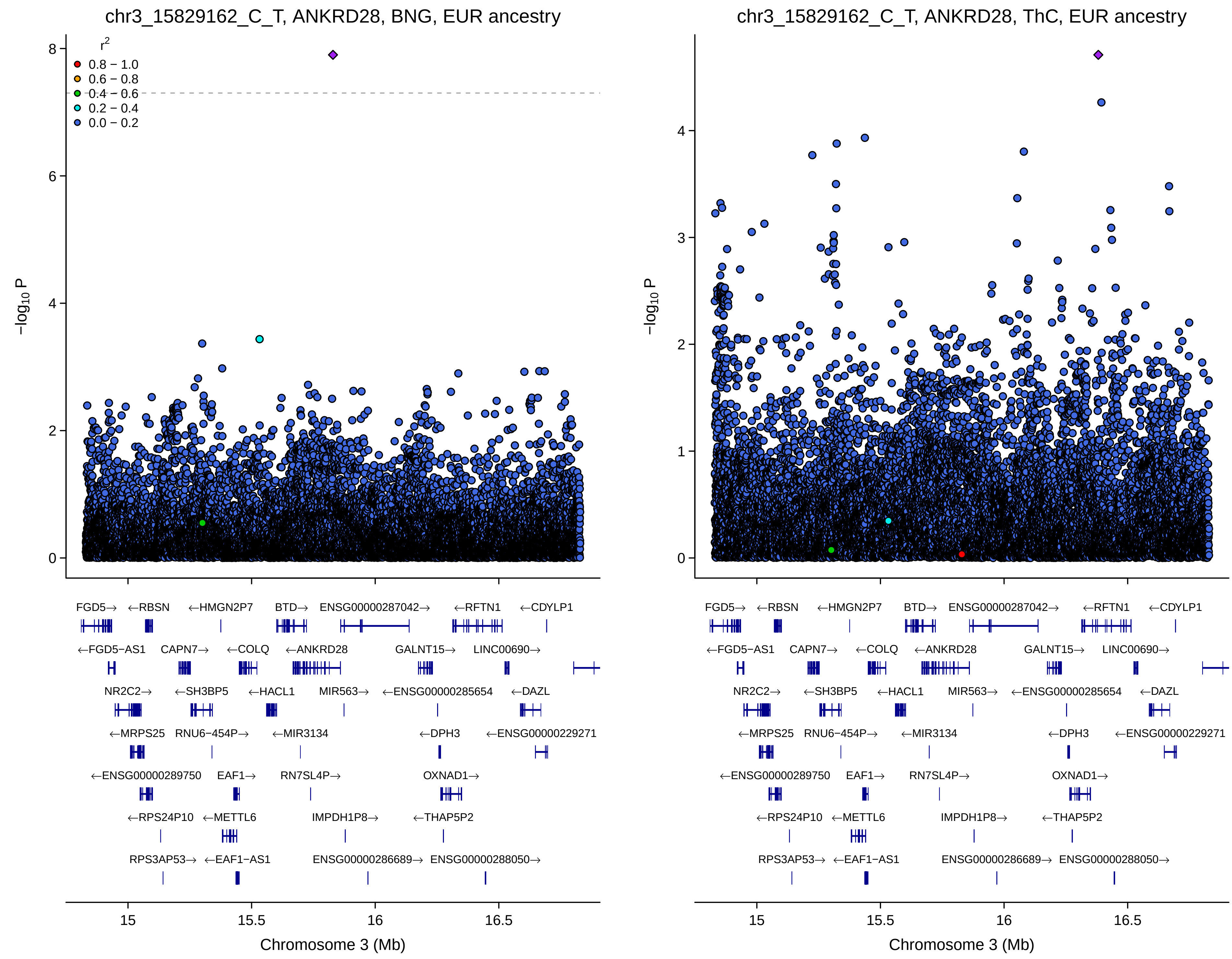

Supplementary Figure 2.2

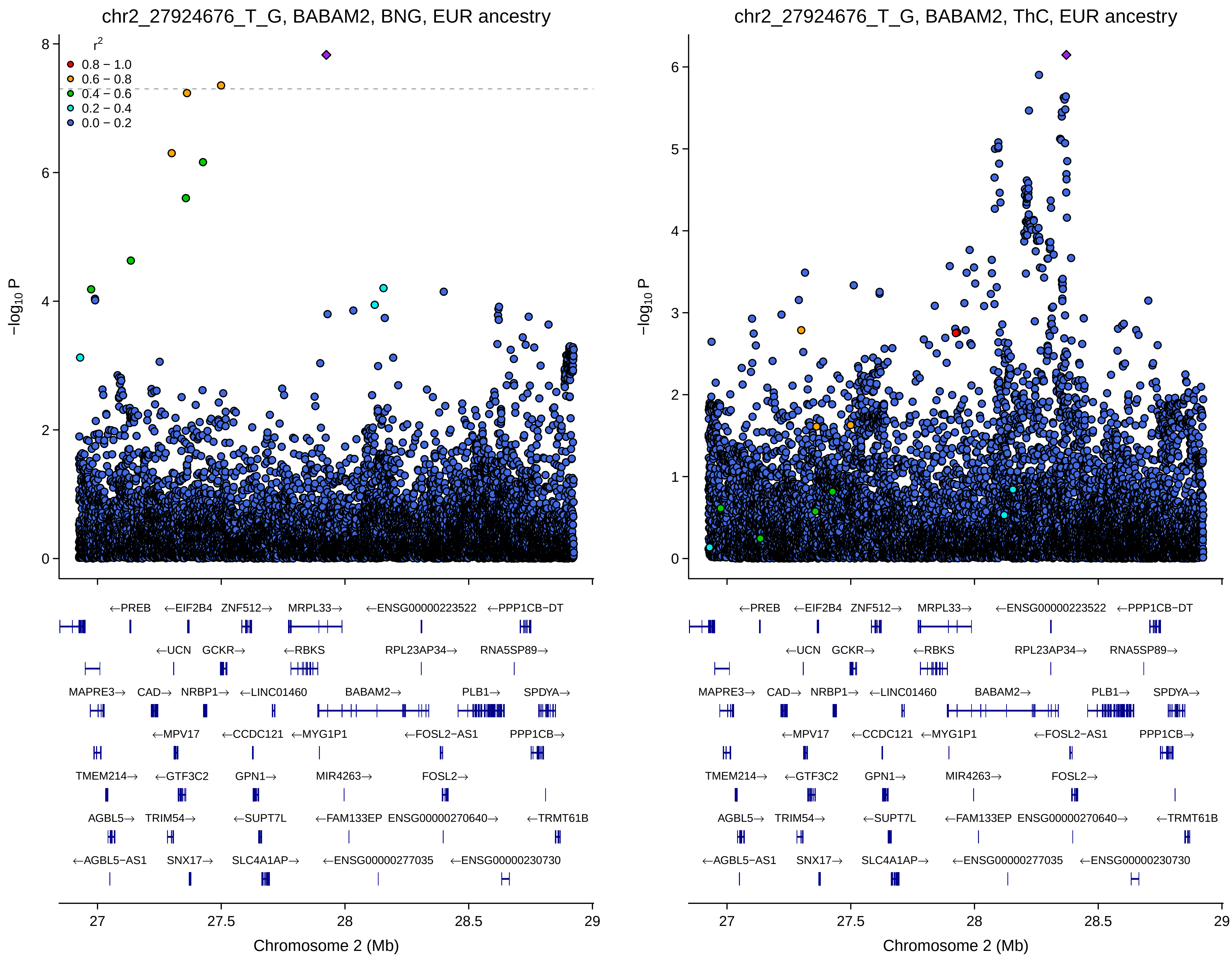

Supplementary Figure 2.2

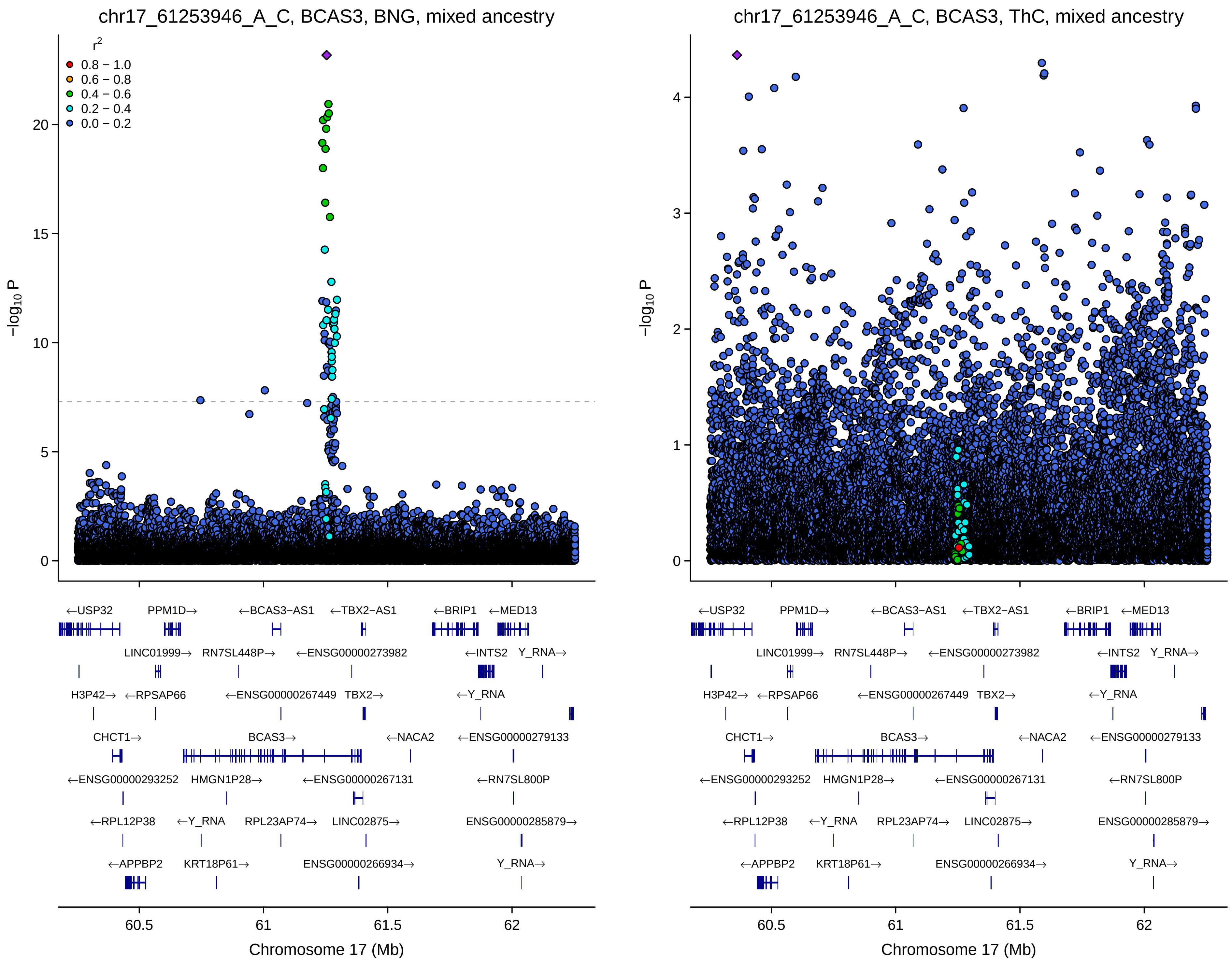

Supplementary Figure 2.2

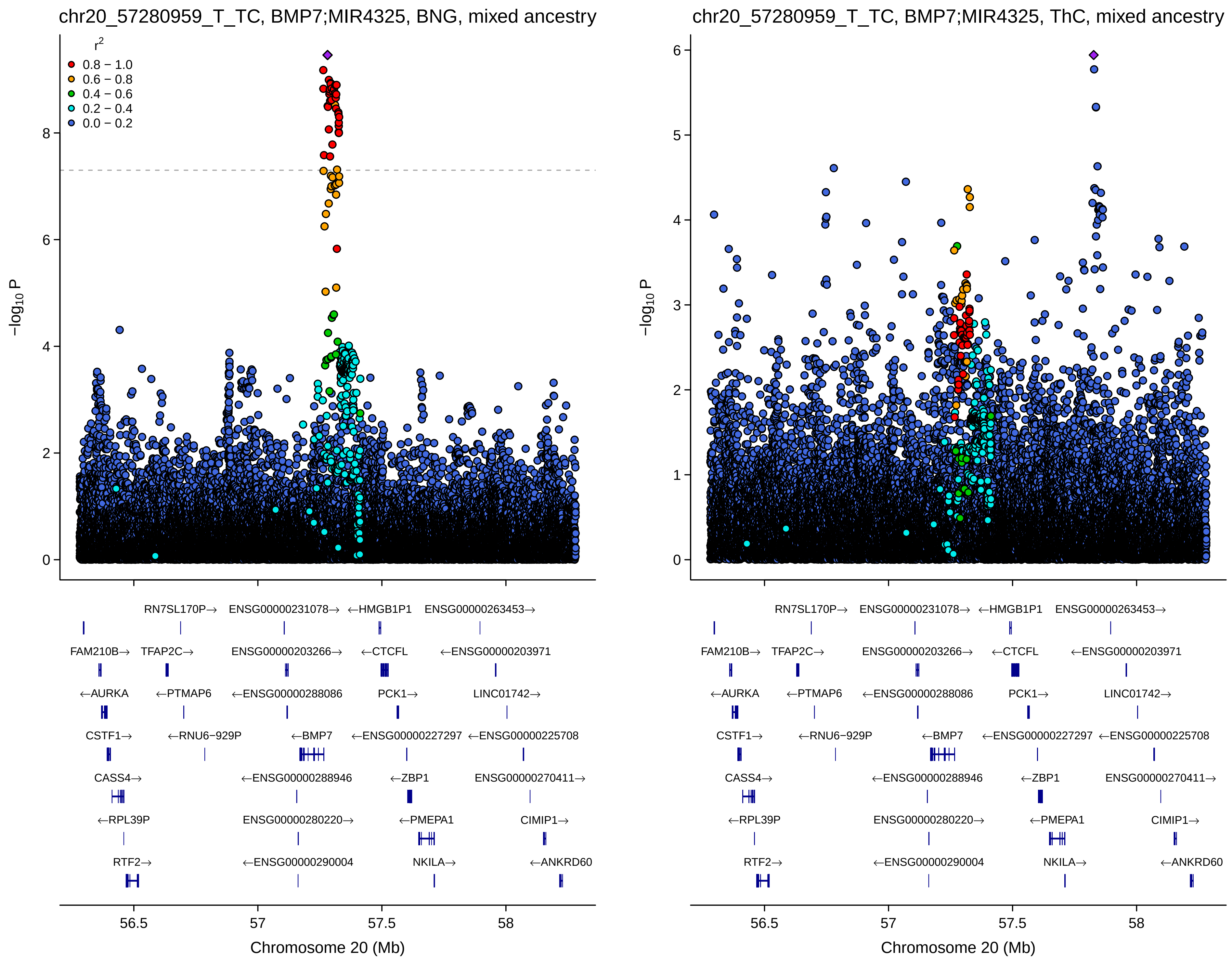

Supplementary Figure 2.2

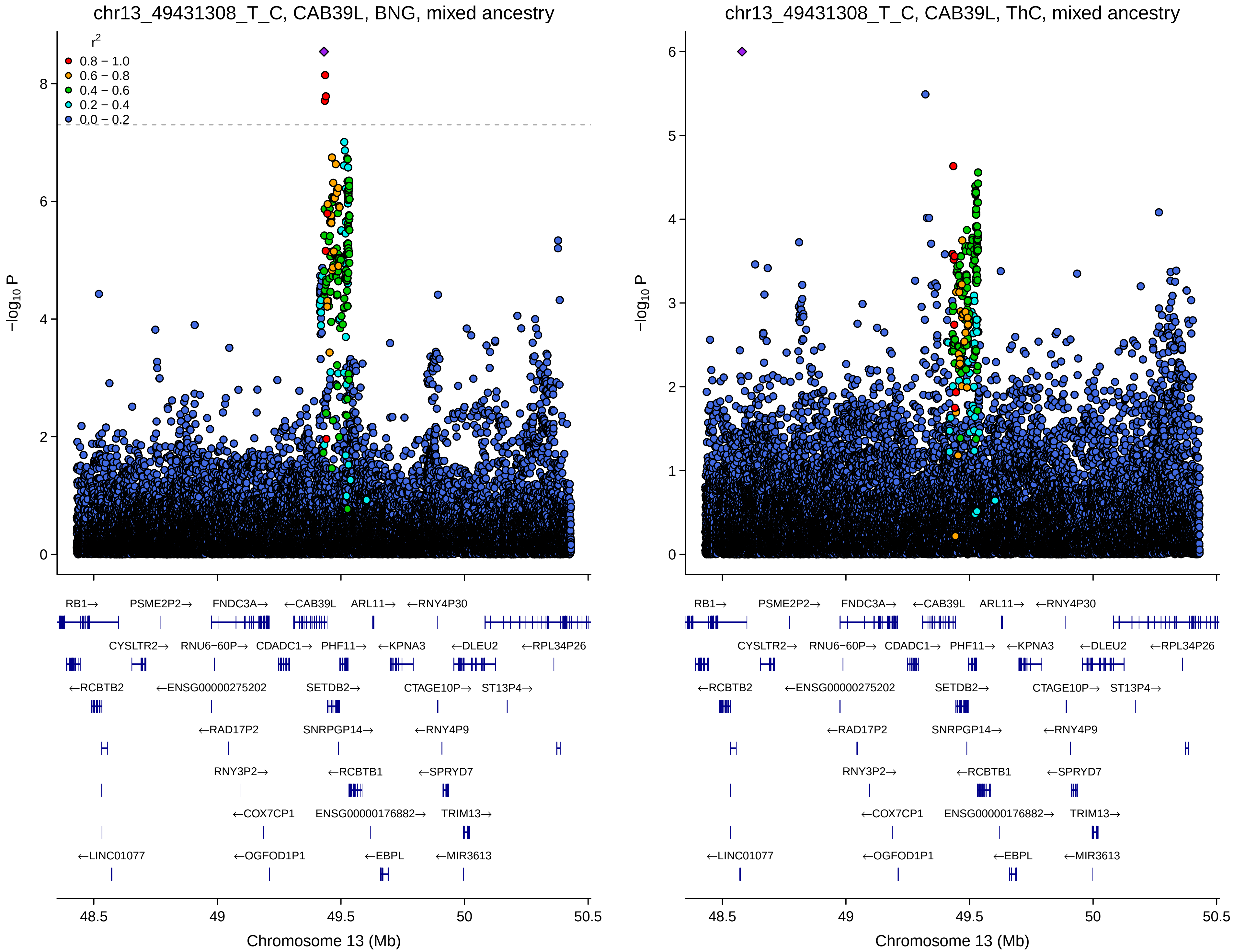

Supplementary Figure 2.2

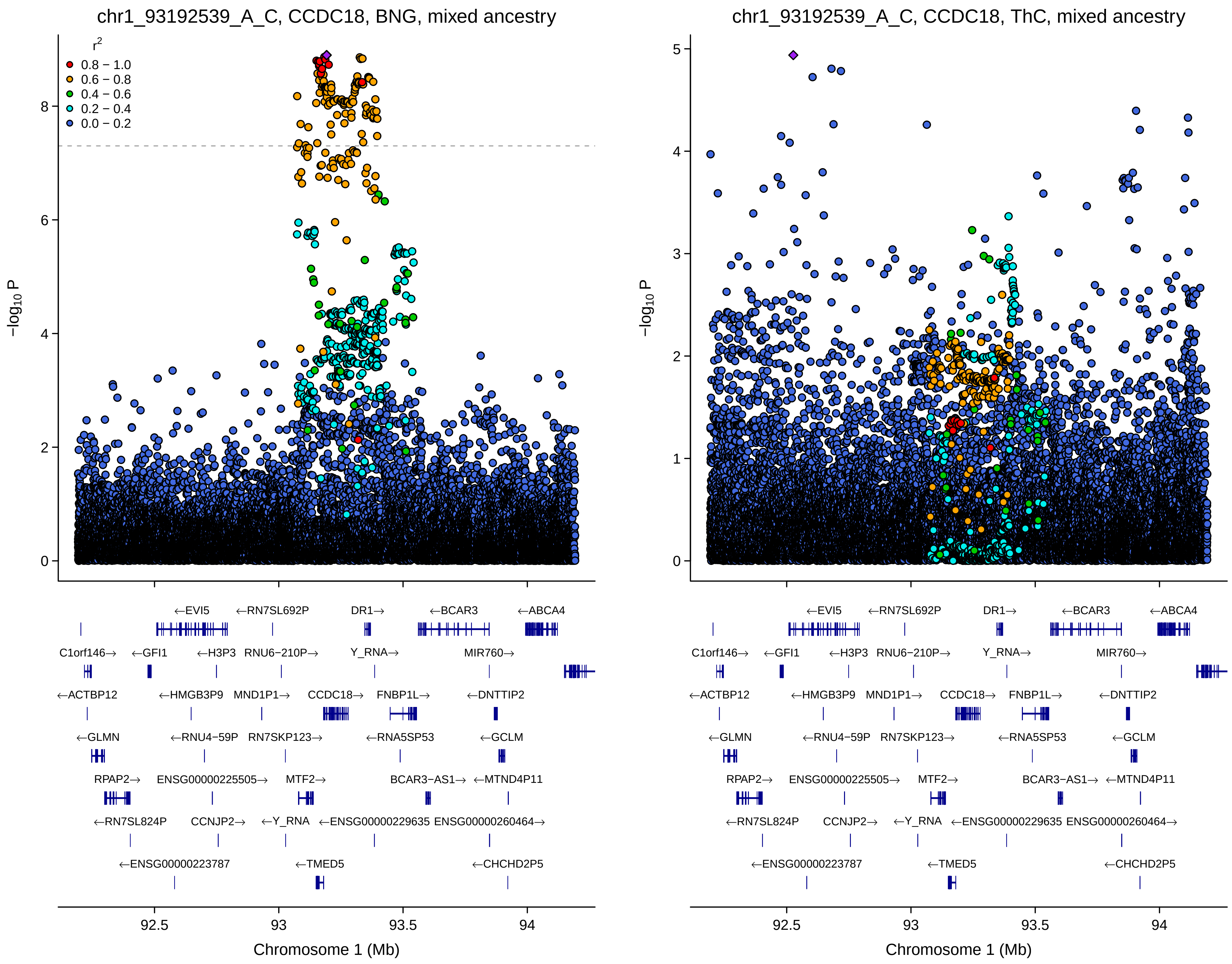

Supplementary Figure 2.2

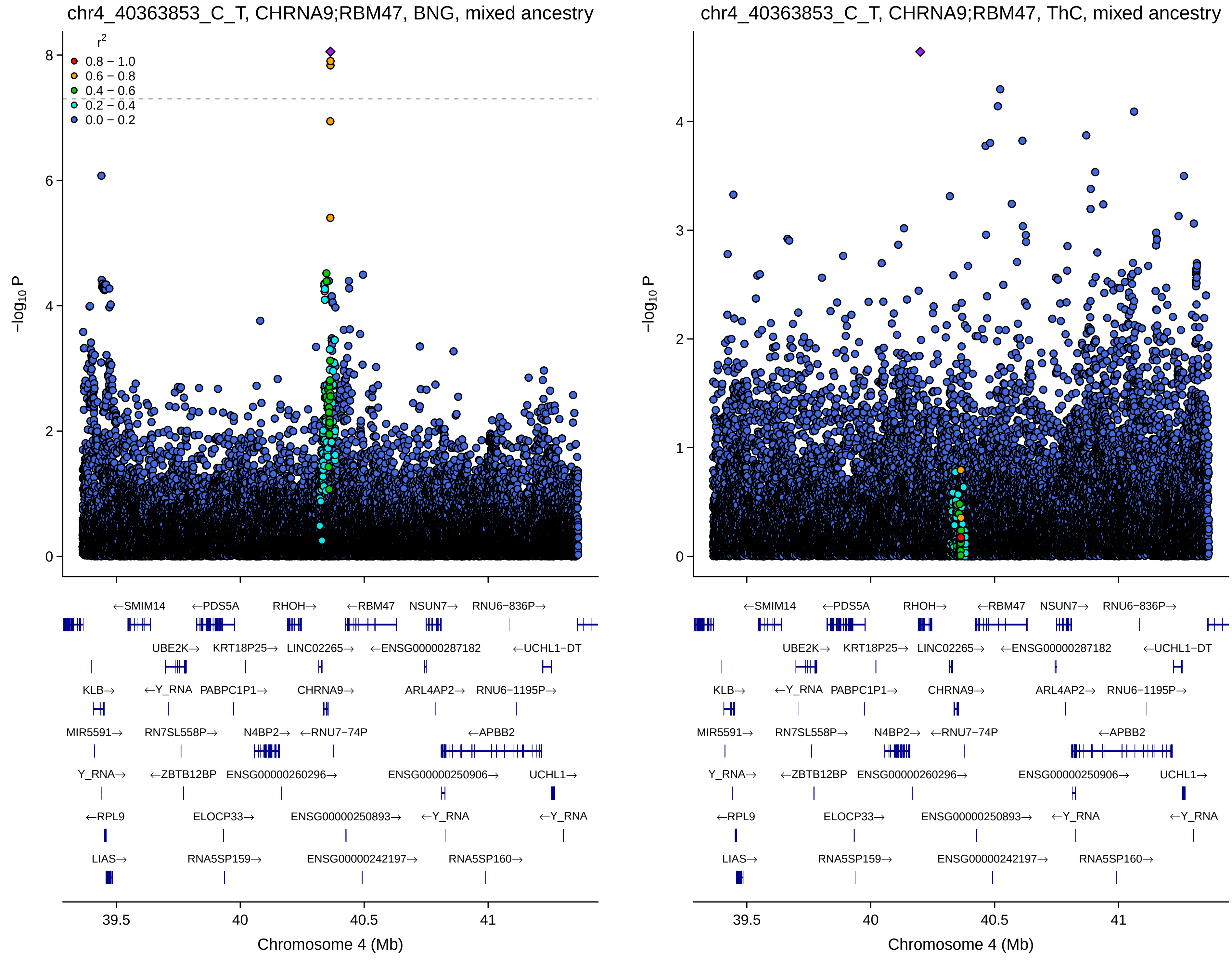

Supplementary Figure 2.2

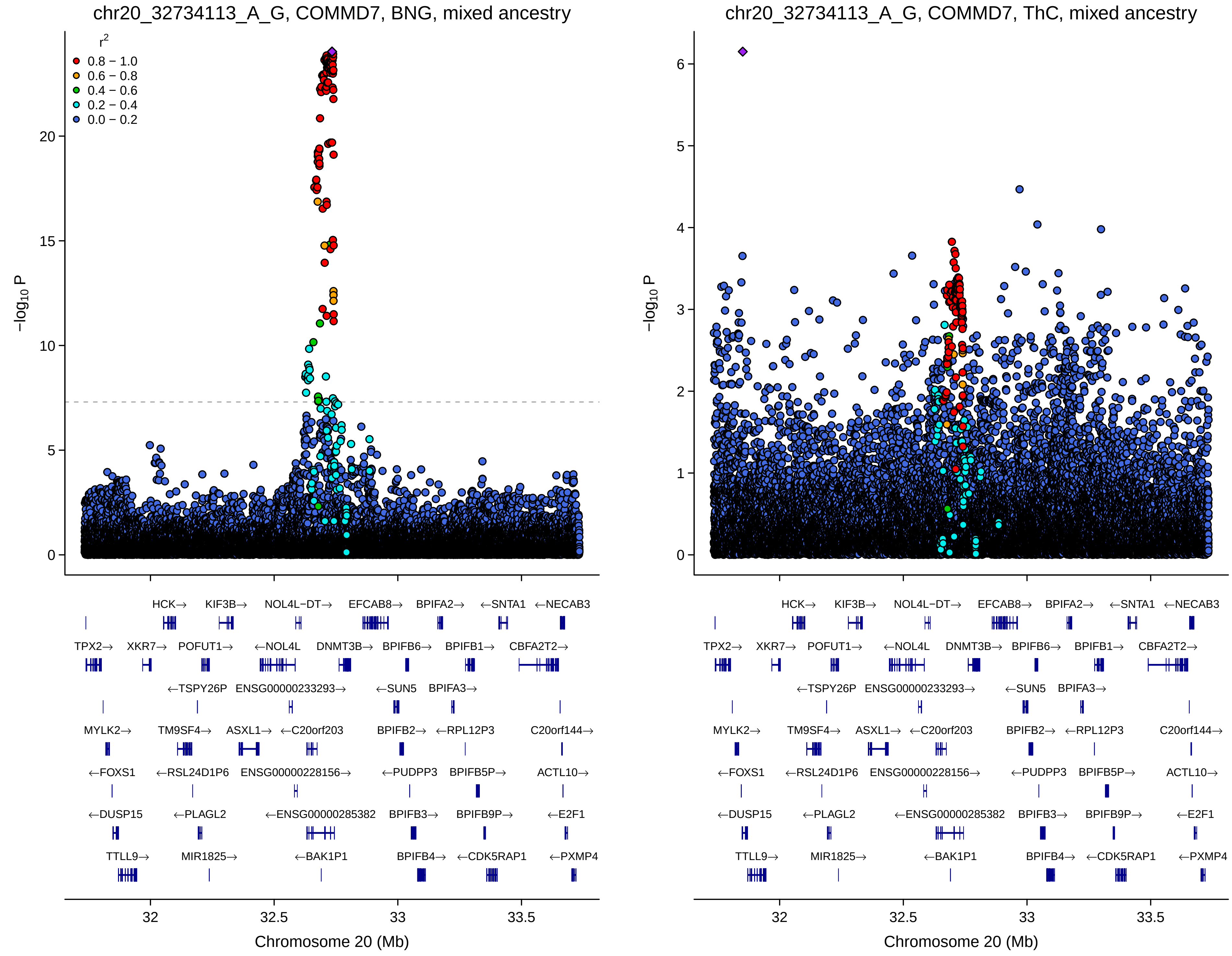

Supplementary Figure 2.2

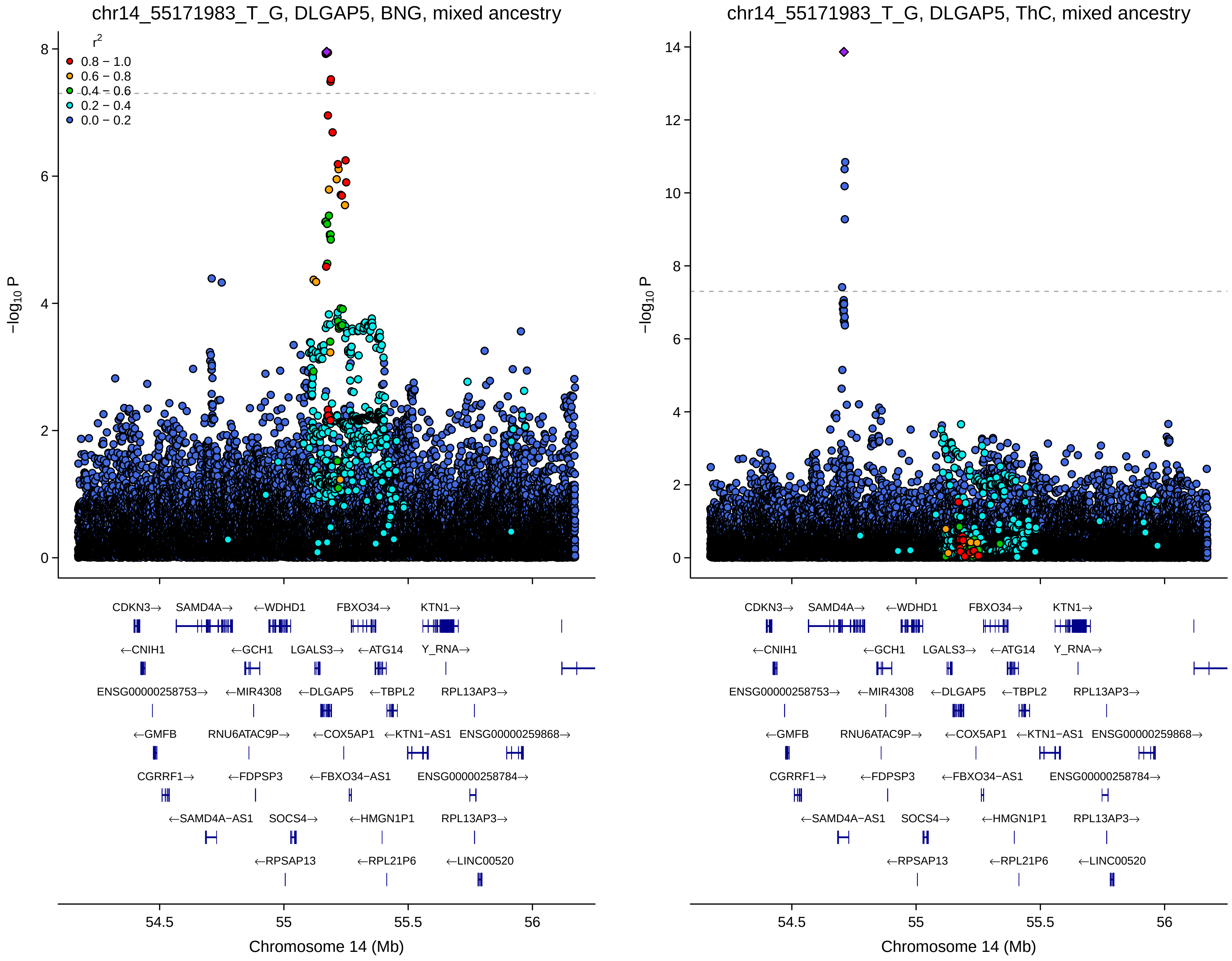

Supplementary Figure 2.2

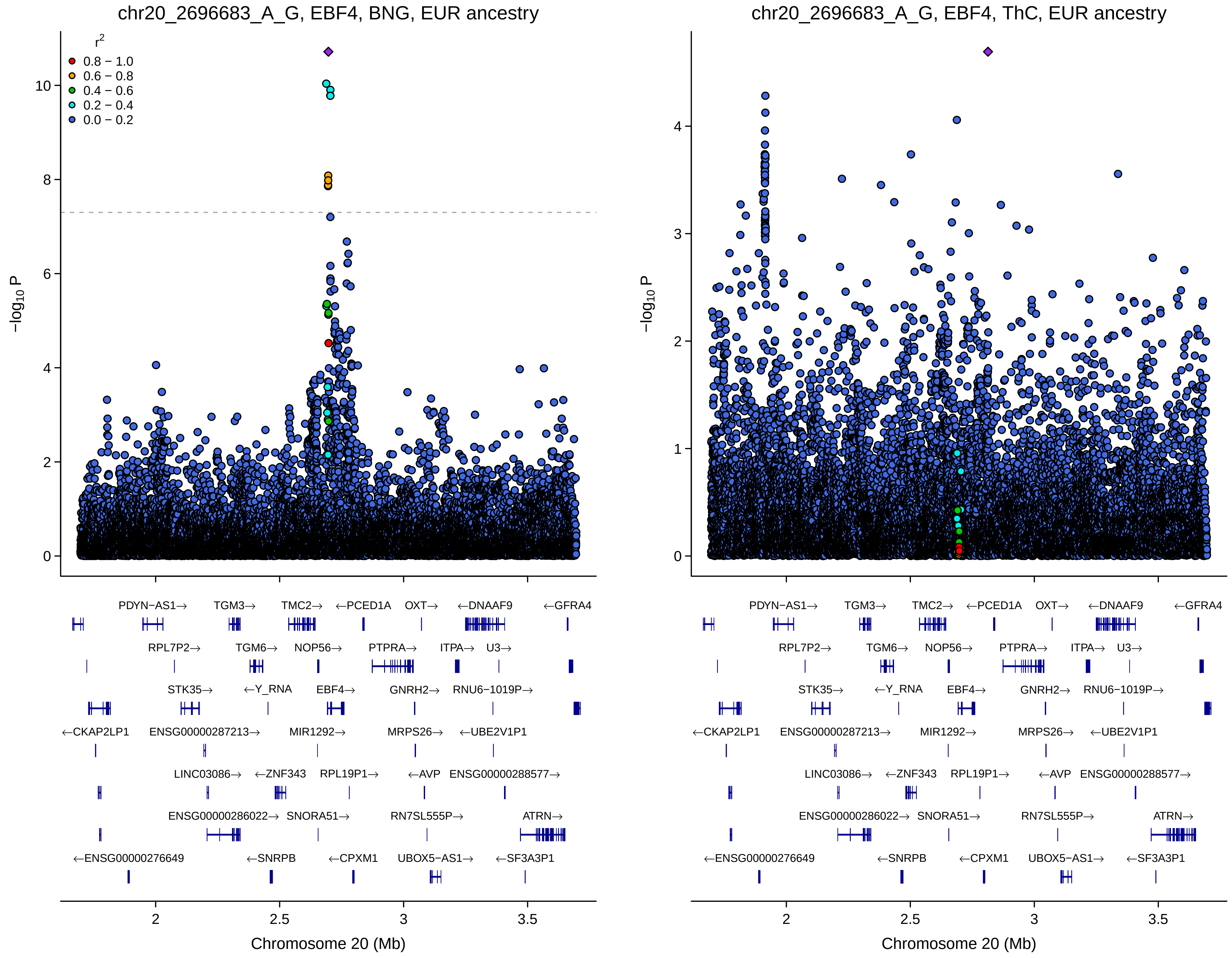

Supplementary Figure 2.2

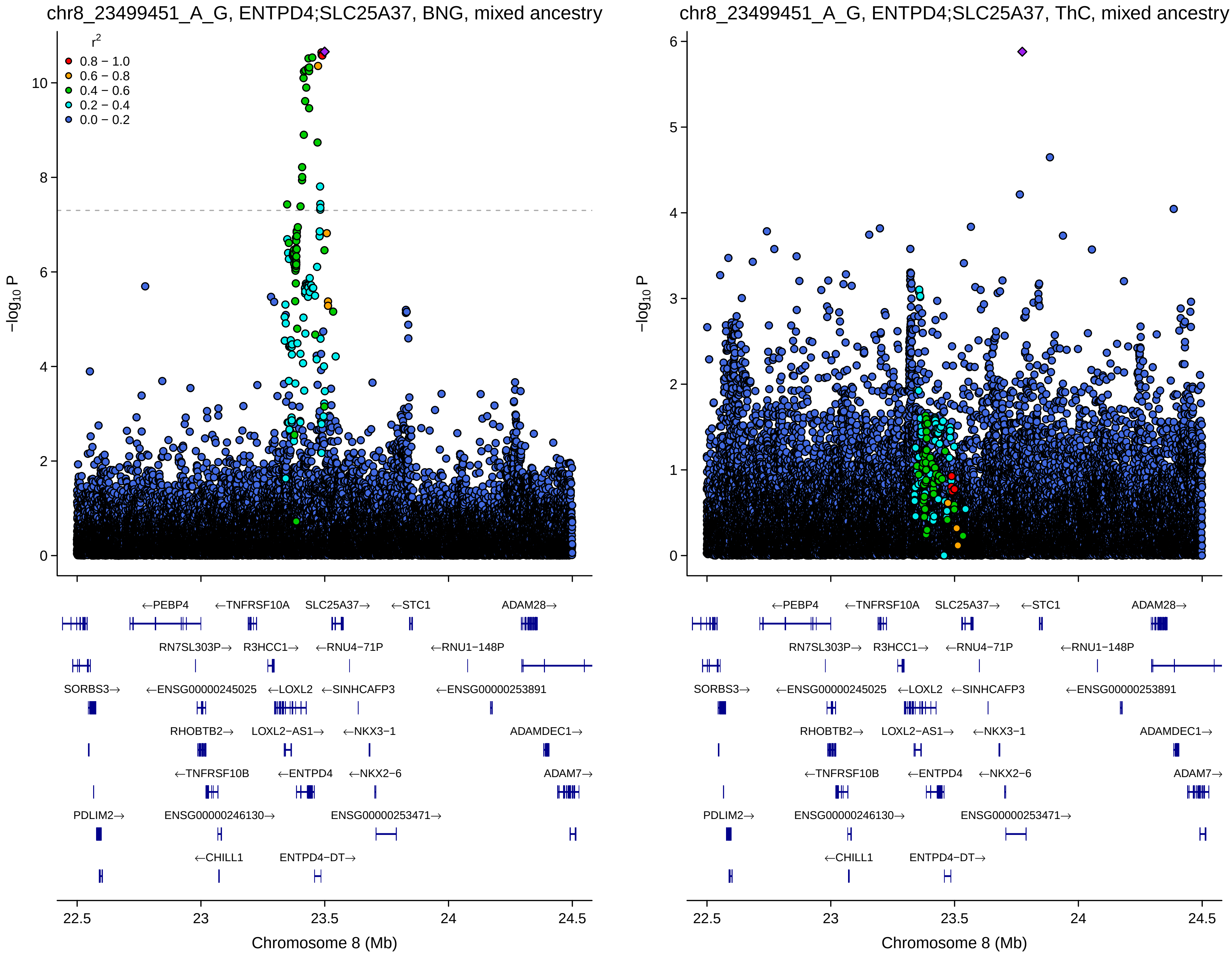

Supplementary Figure 2.2

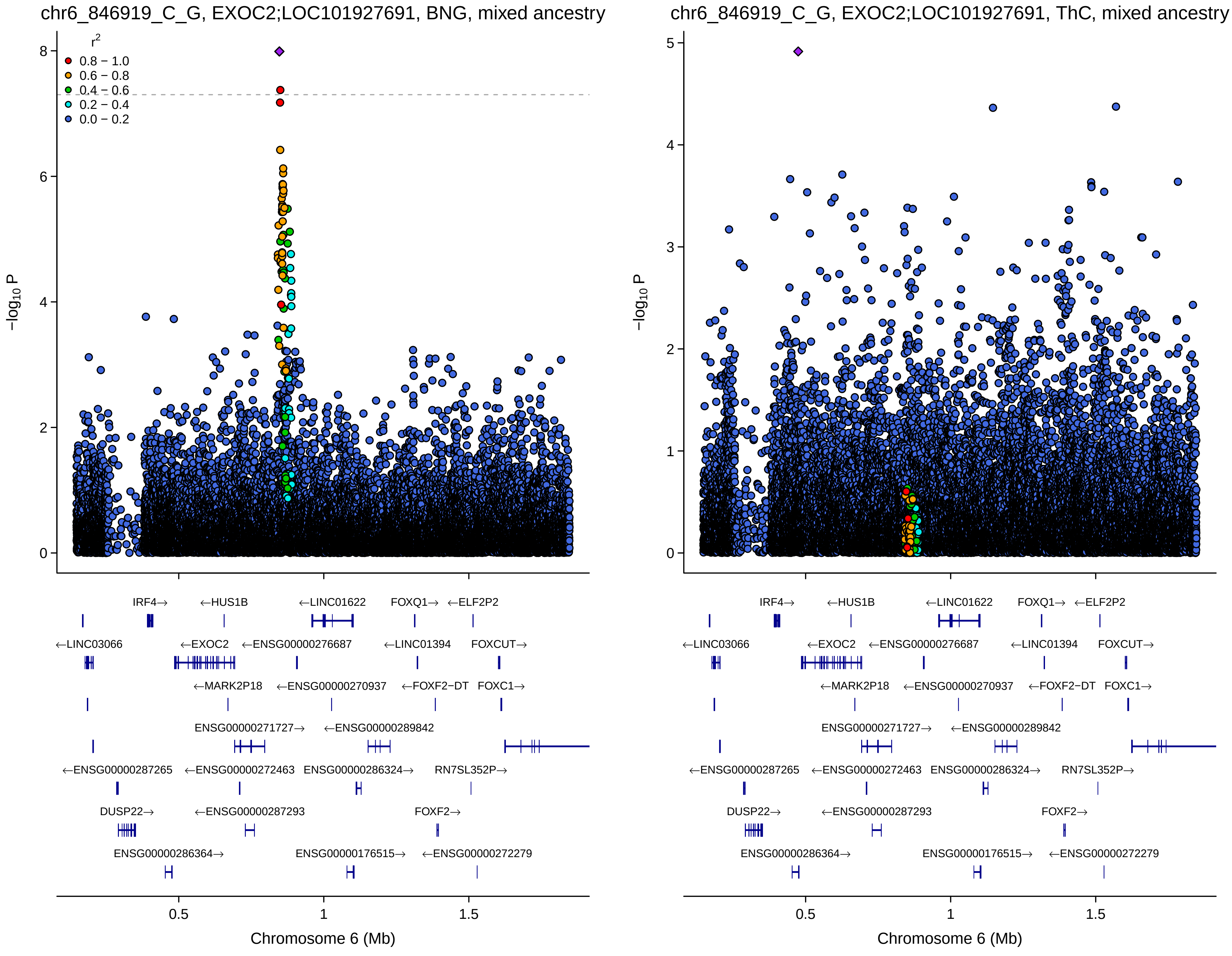

Supplementary Figure 2.2

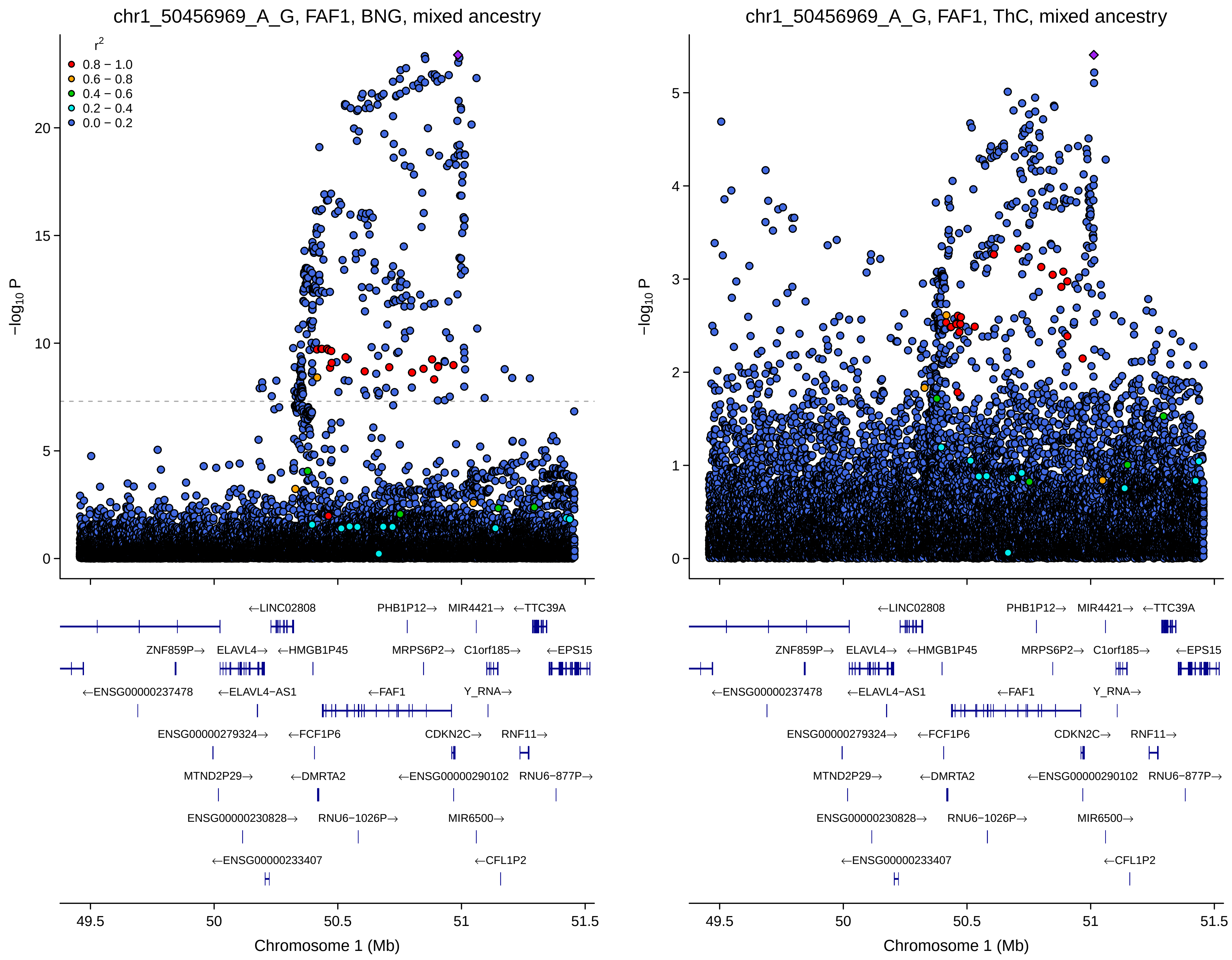

Supplementary Figure 2.2

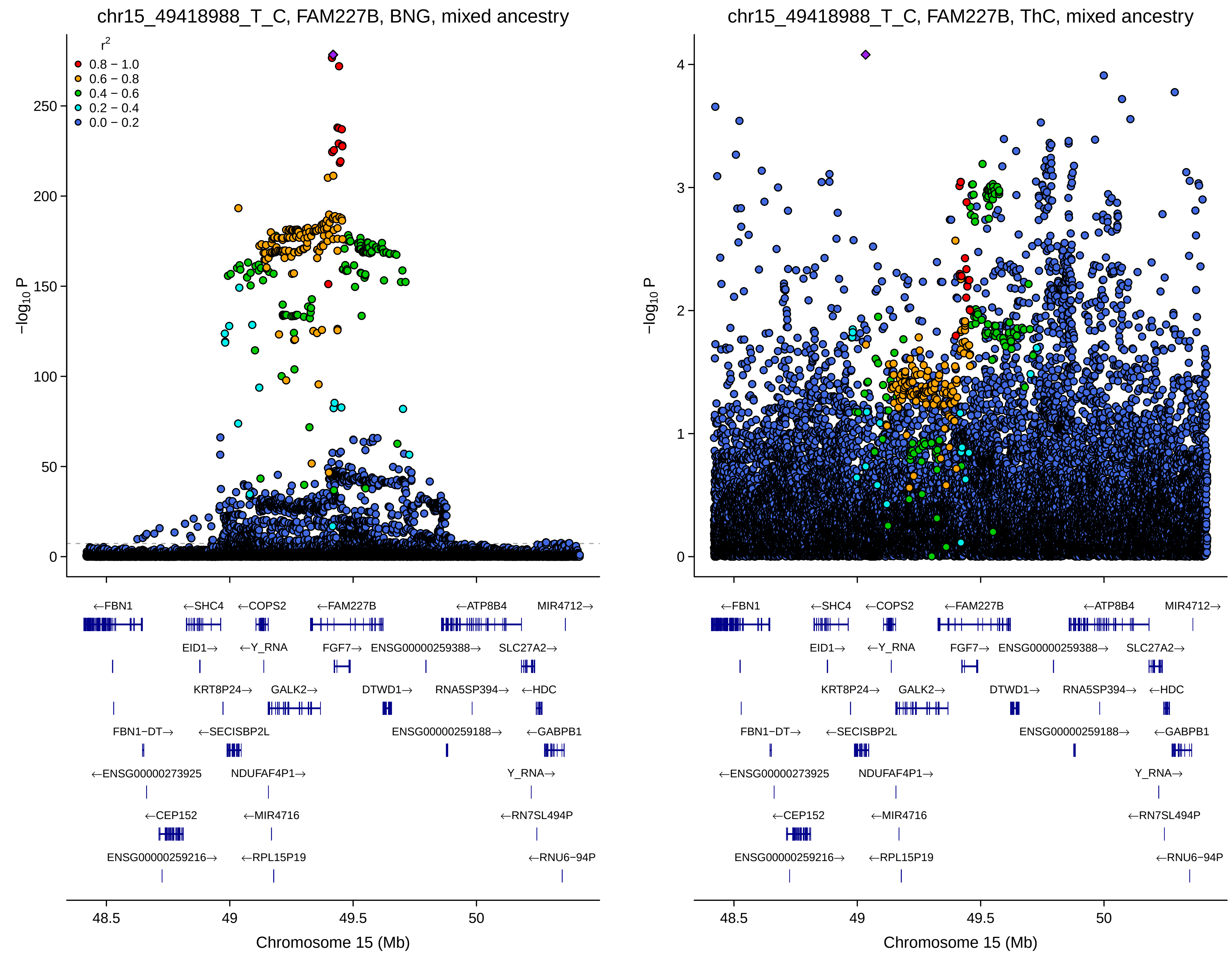

Supplementary Figure 2.2

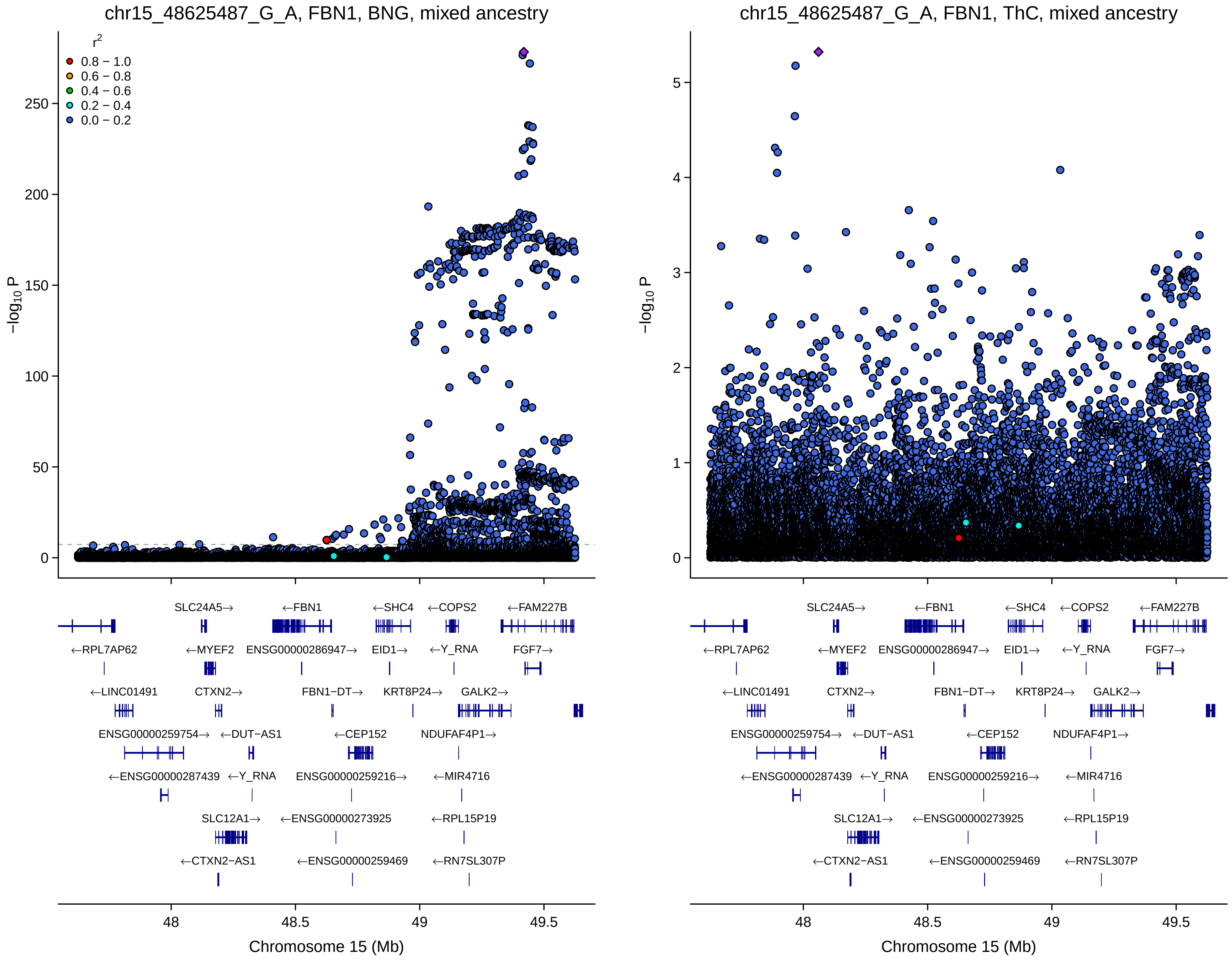

Supplementary Figure 2.2

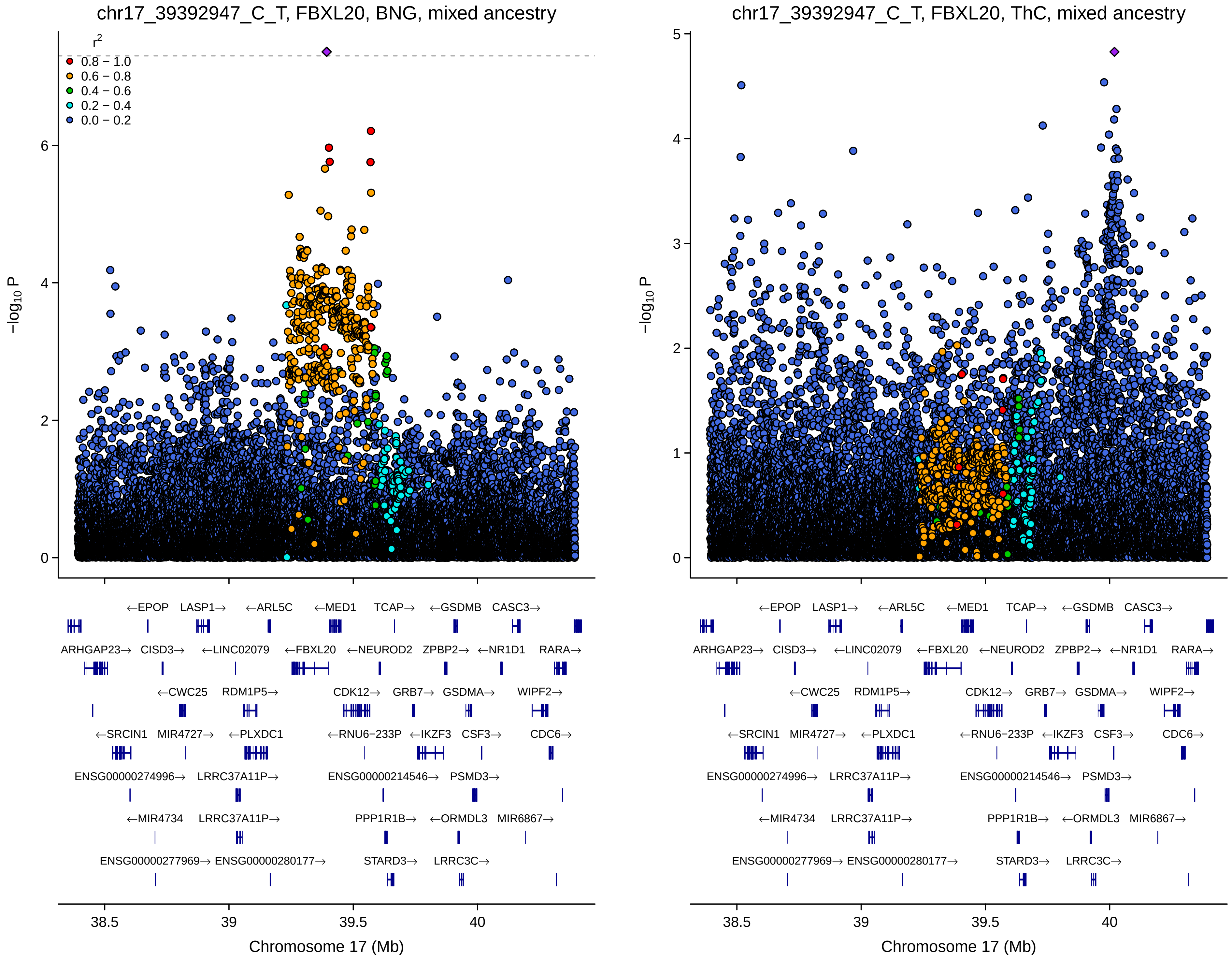

Supplementary Figure 2.2

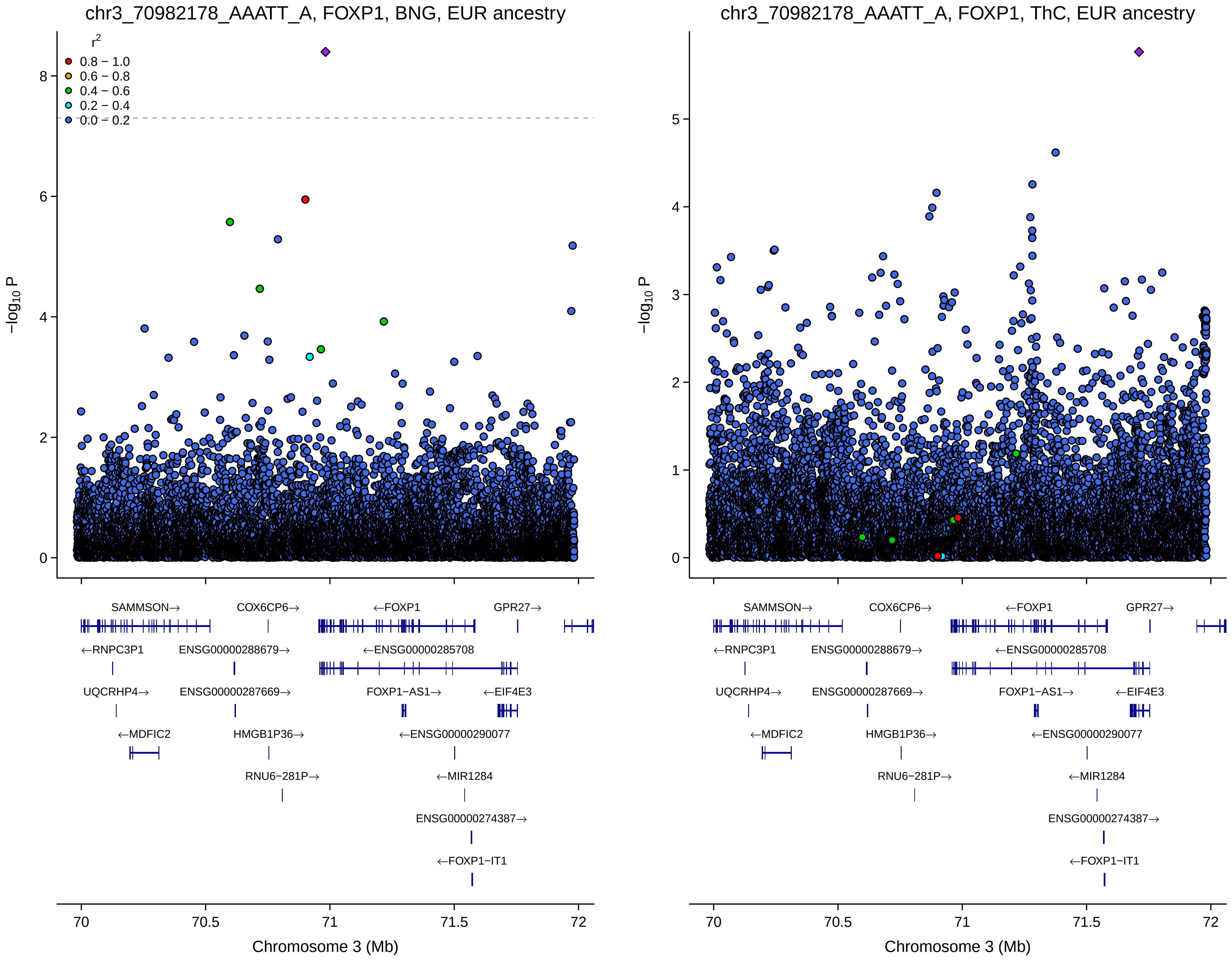

Supplementary Figure 2.2

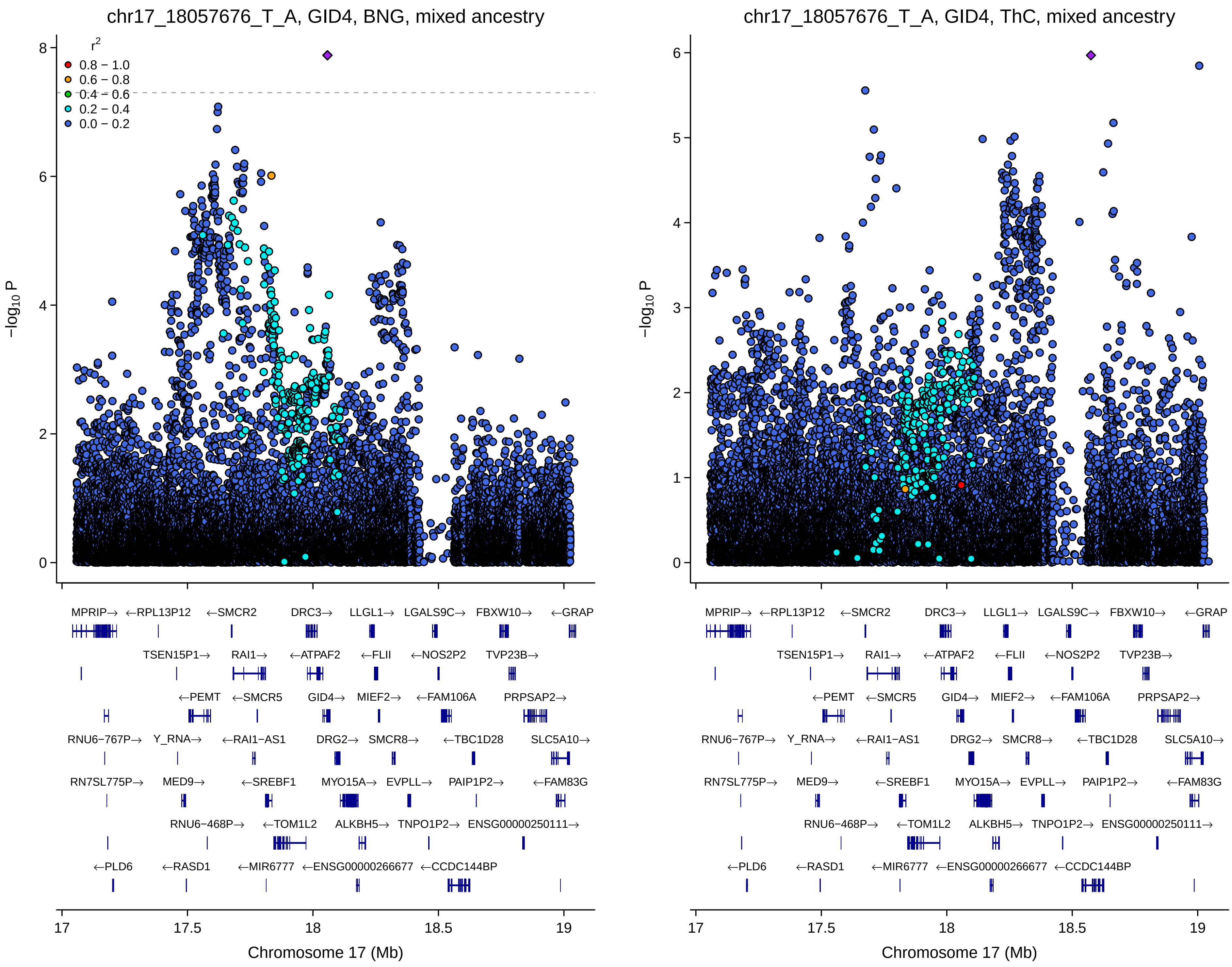

Supplementary Figure 2.2

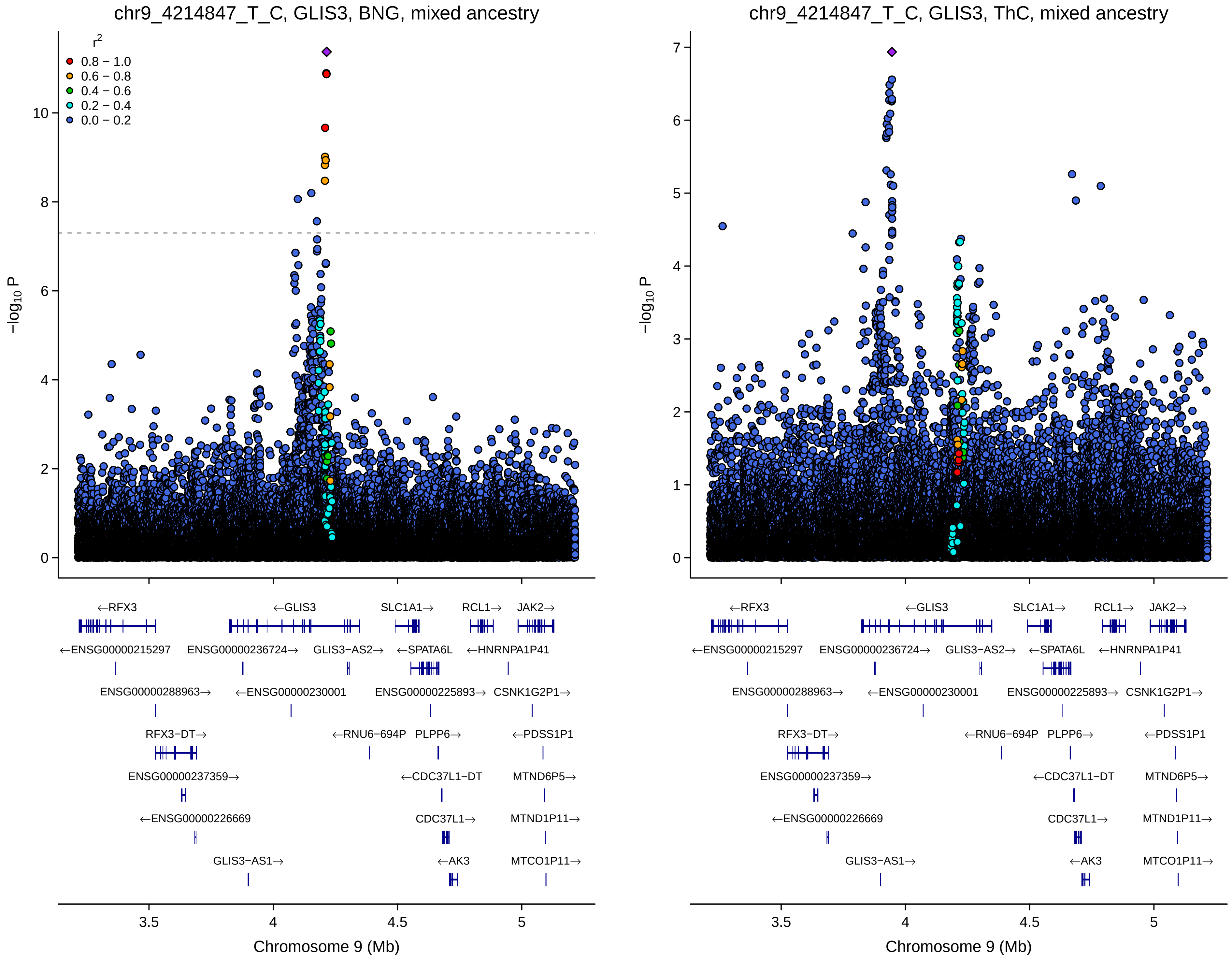

Supplementary Figure 2.2

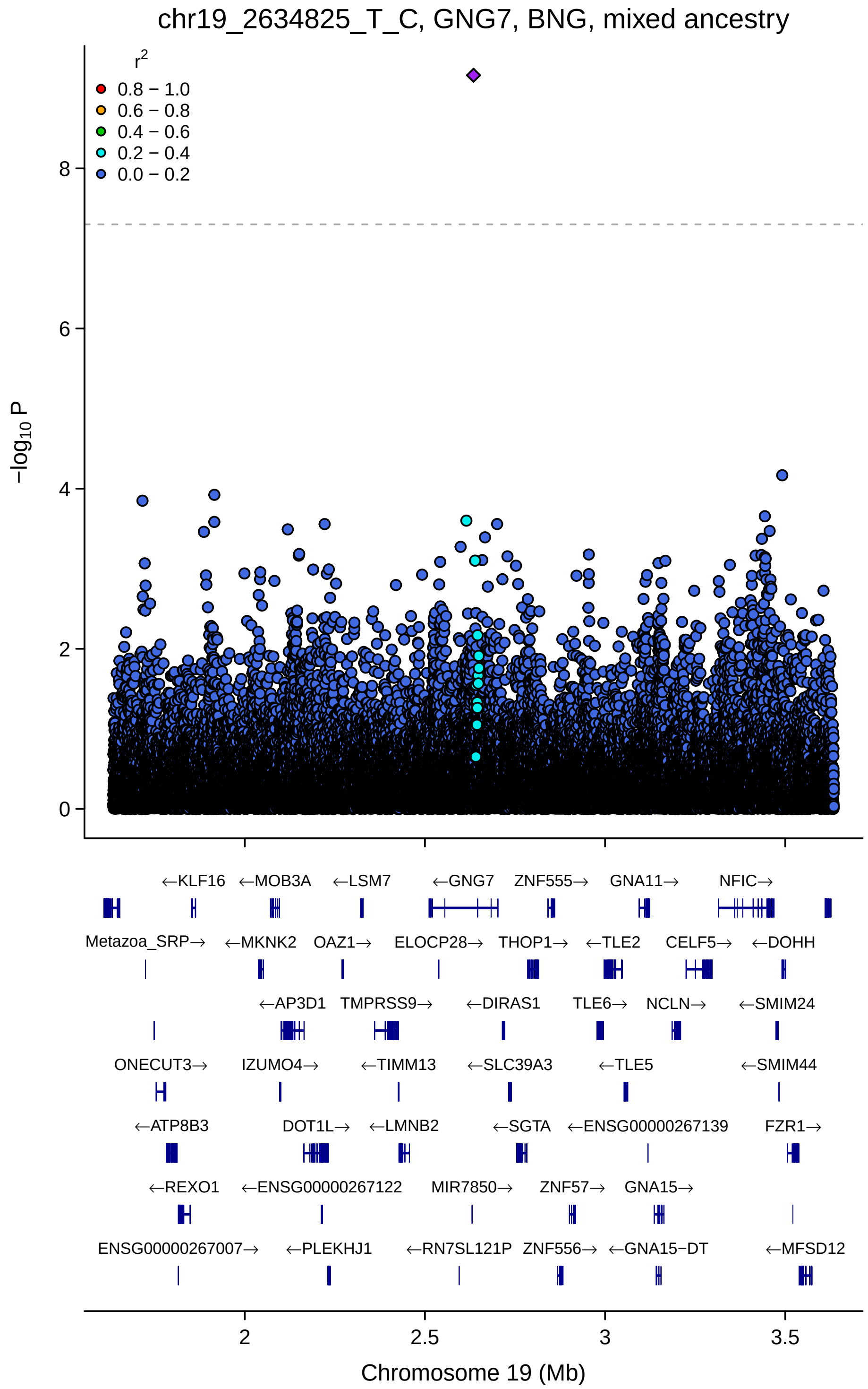

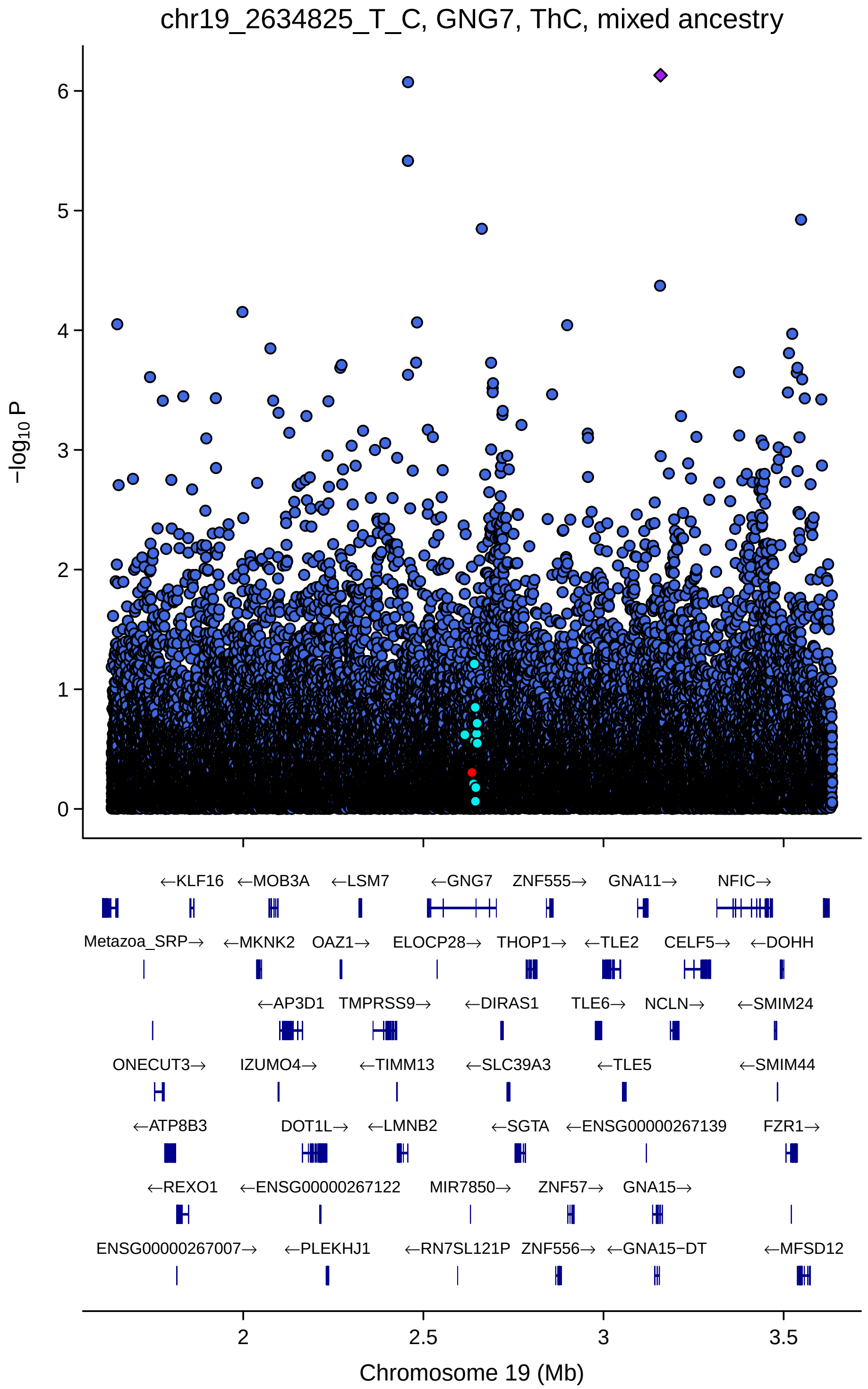

Supplementary Figure 2.2

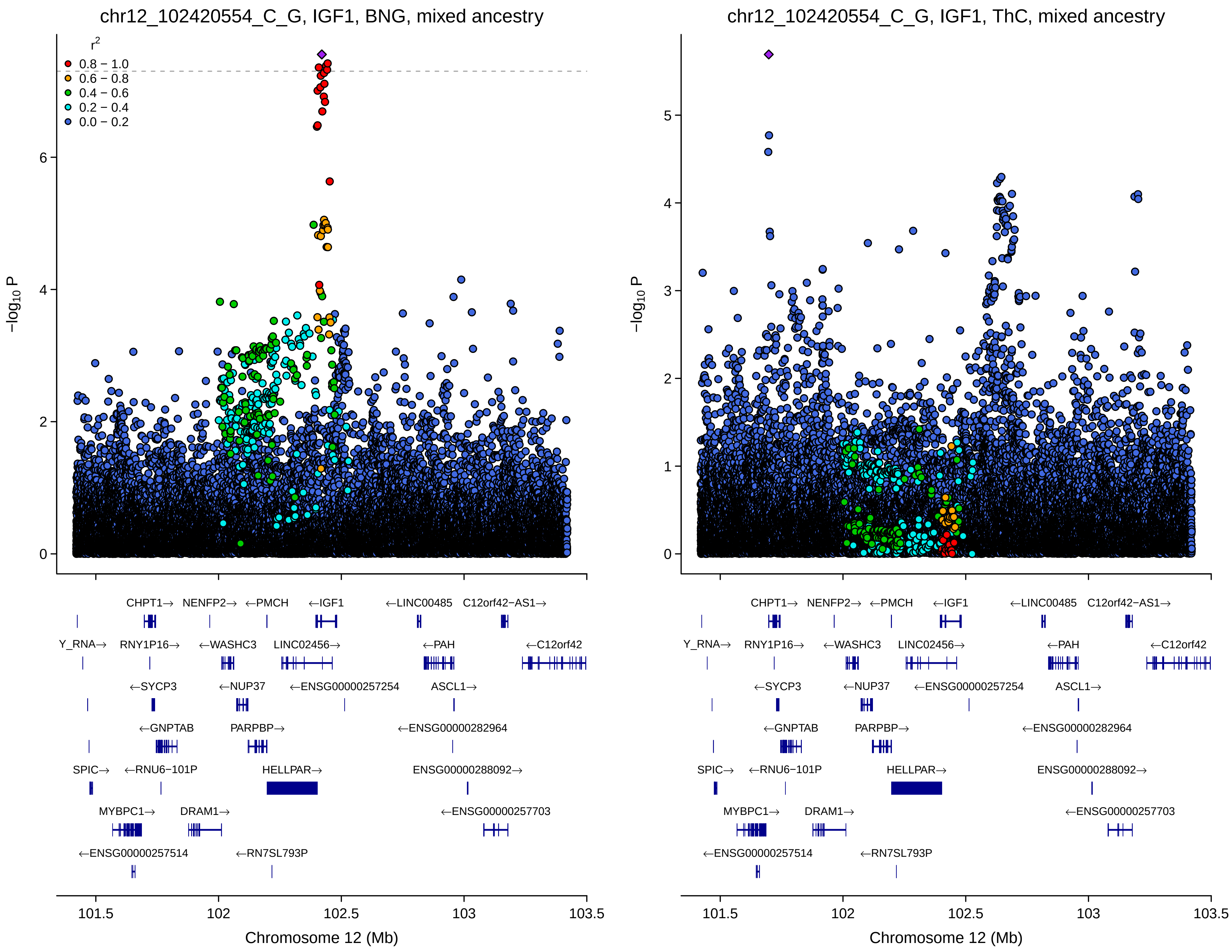

Supplementary Figure 2.2

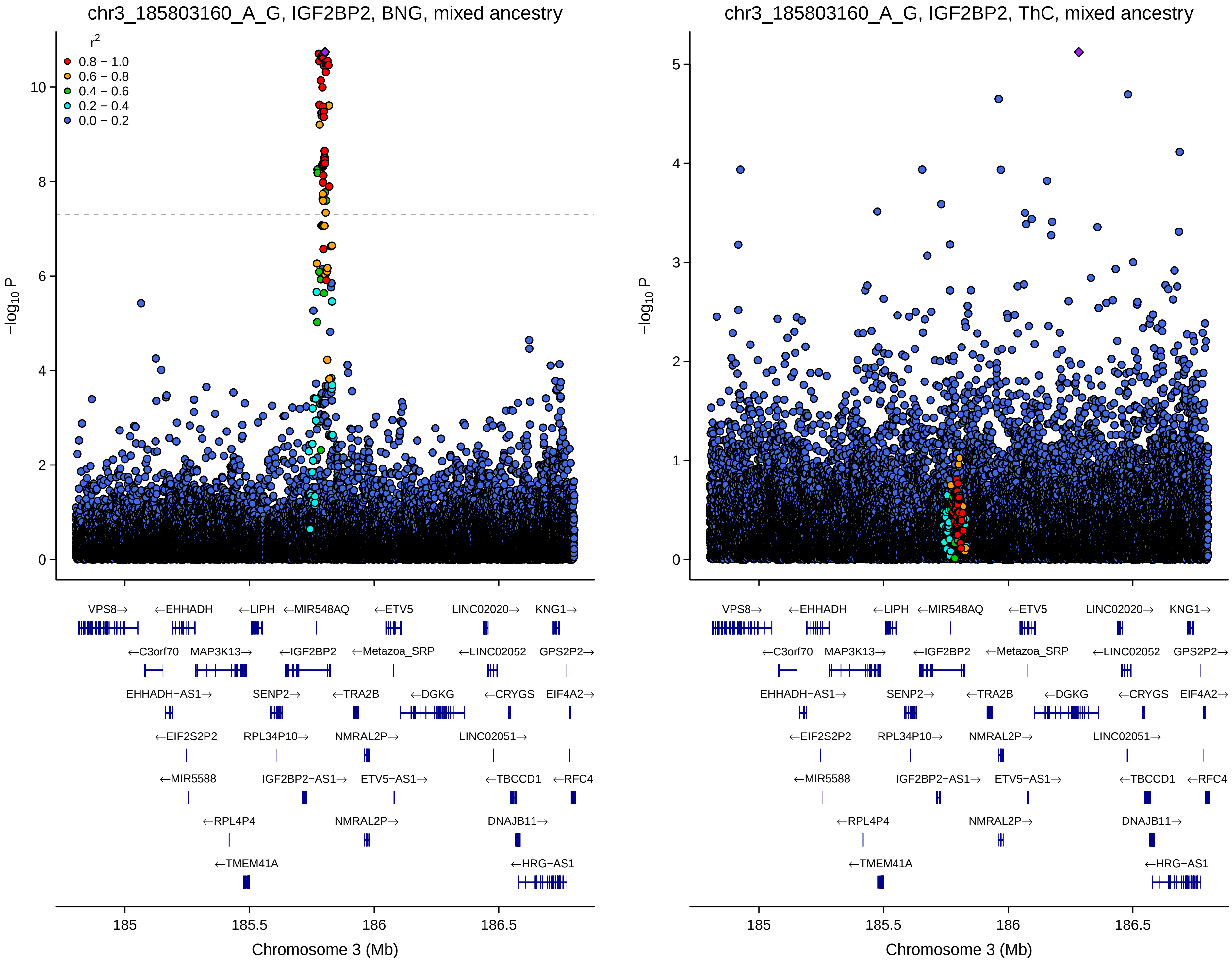

Supplementary Figure 2.2

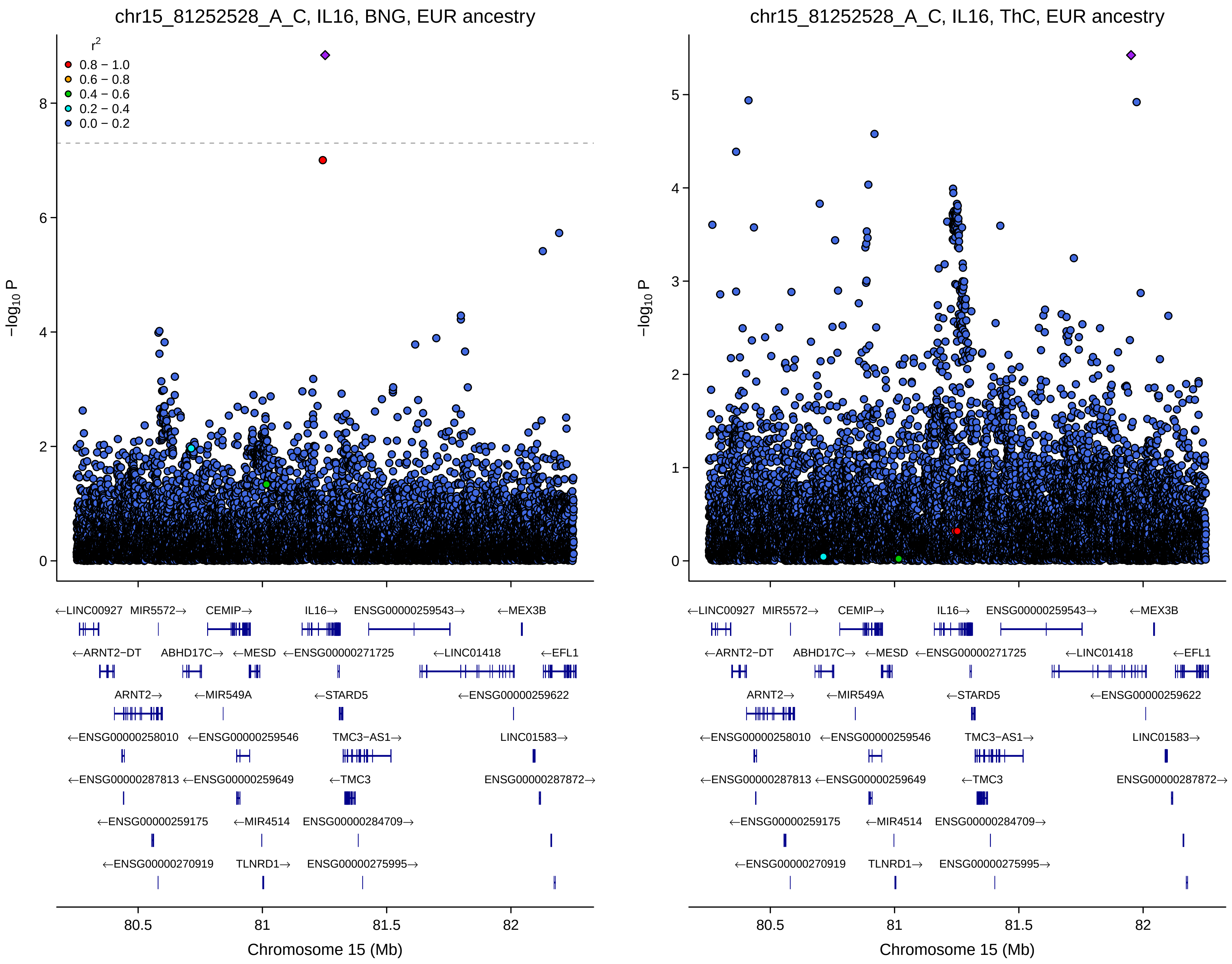

Supplementary Figure 2.2

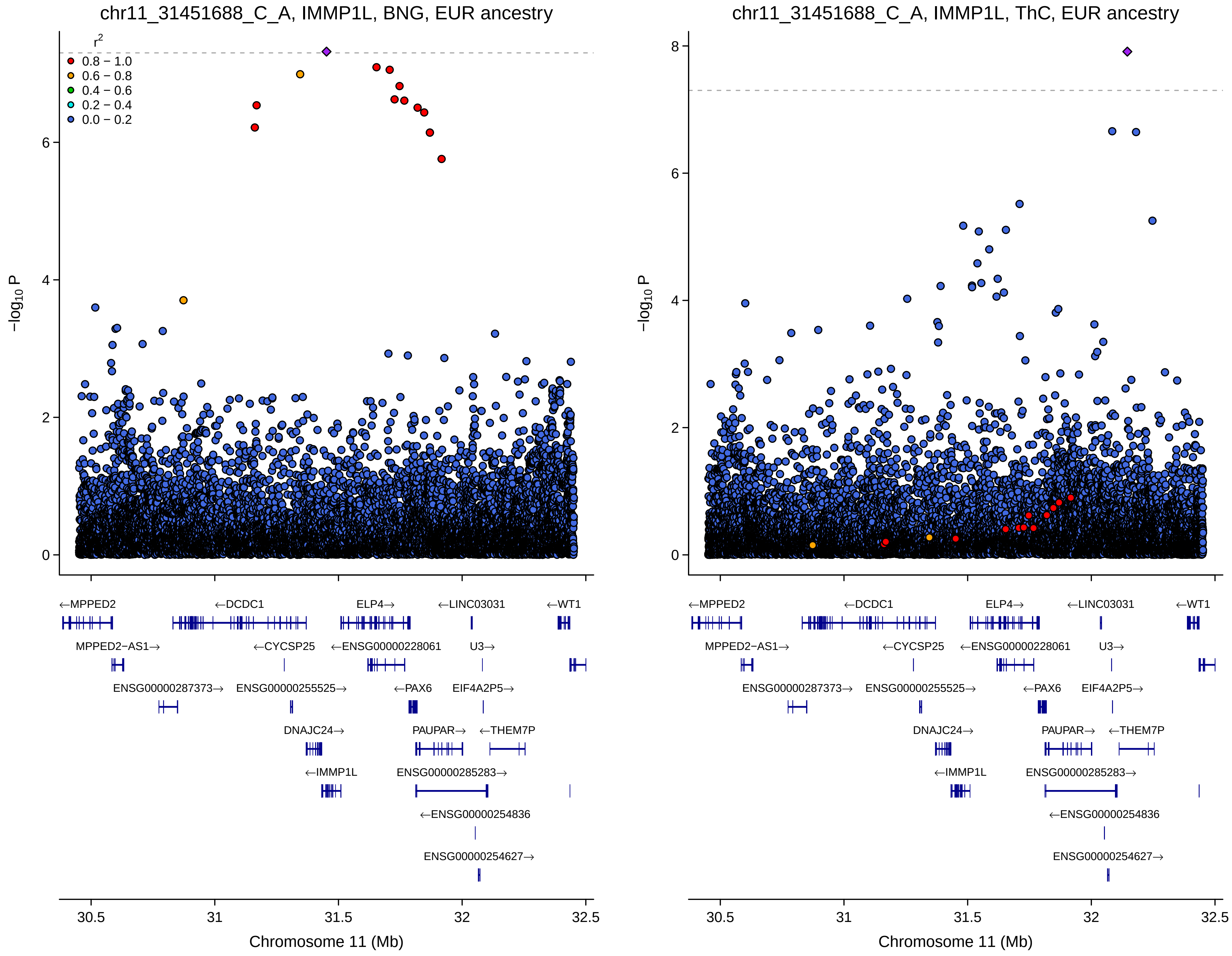

Supplementary Figure 2.2

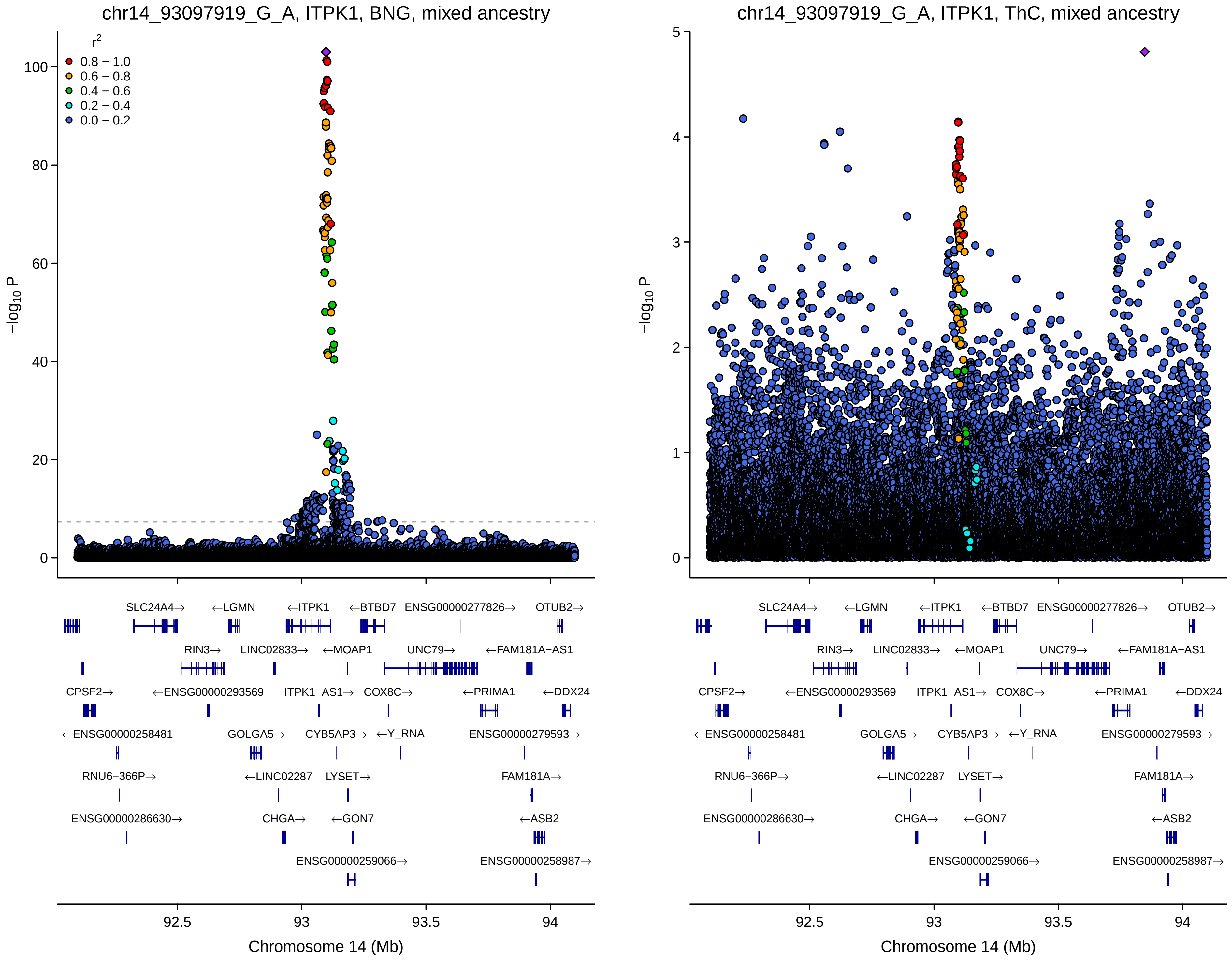

Supplementary Figure 2.2

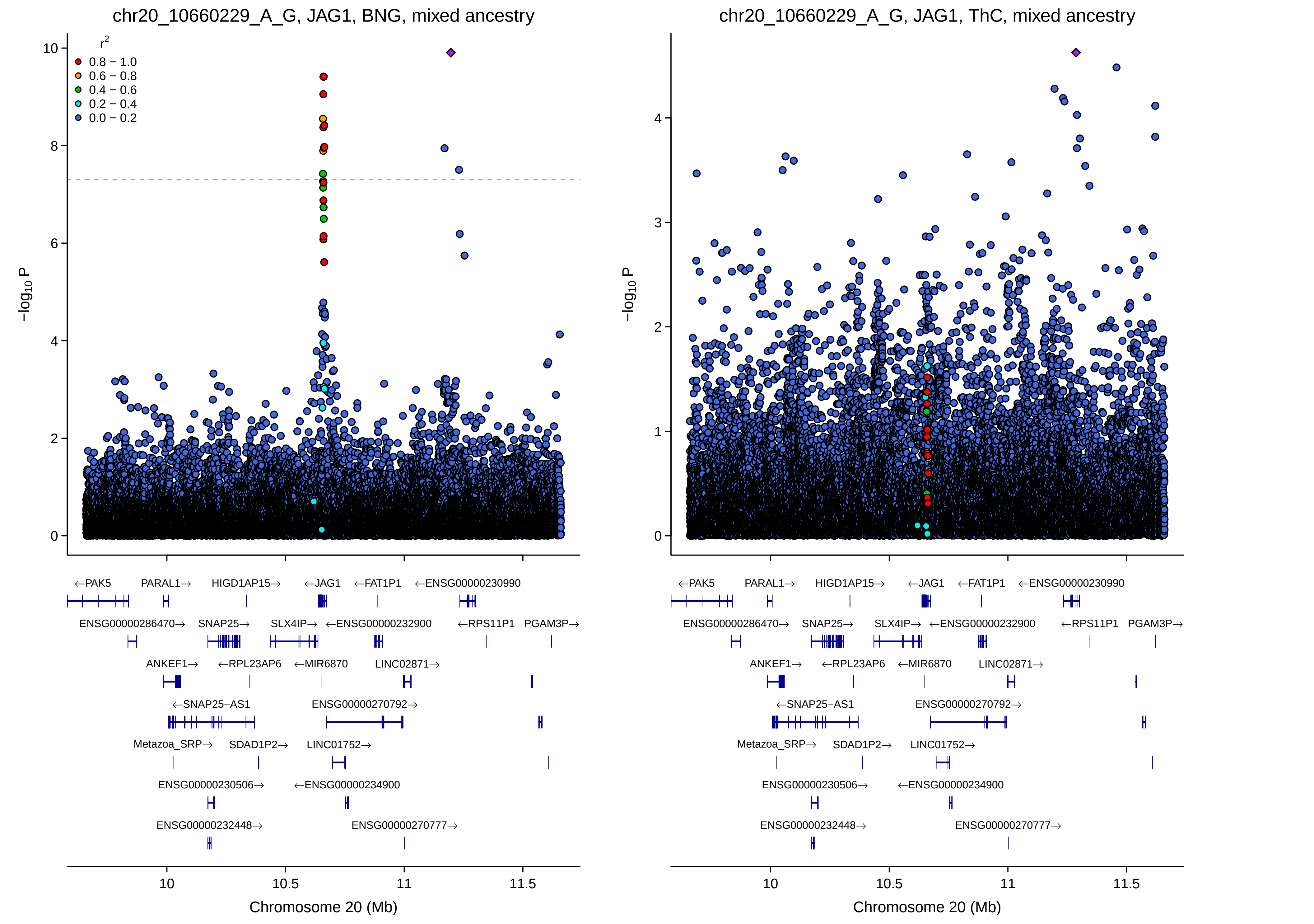

Supplementary Figure 2.2

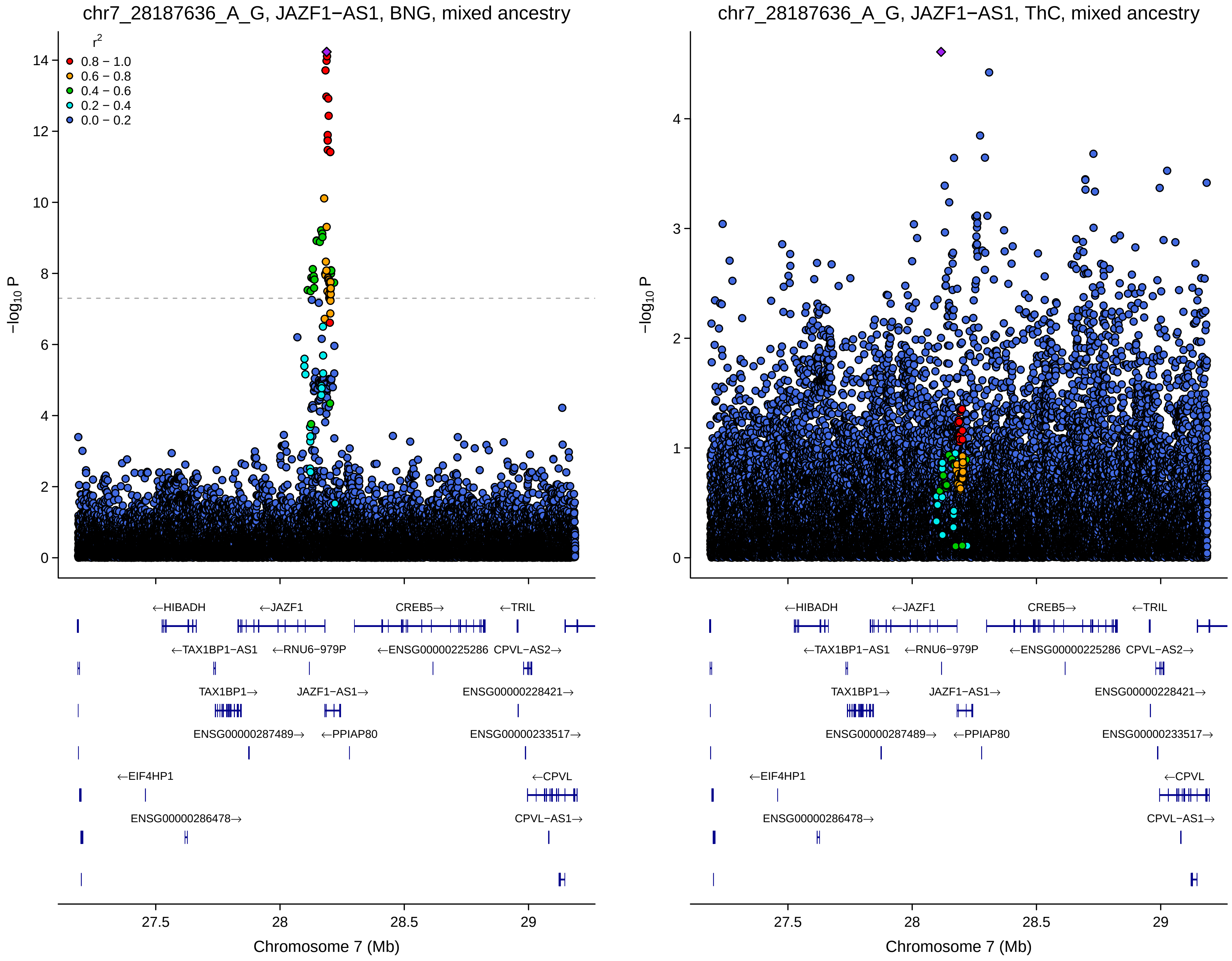

Supplementary Figure 2.2

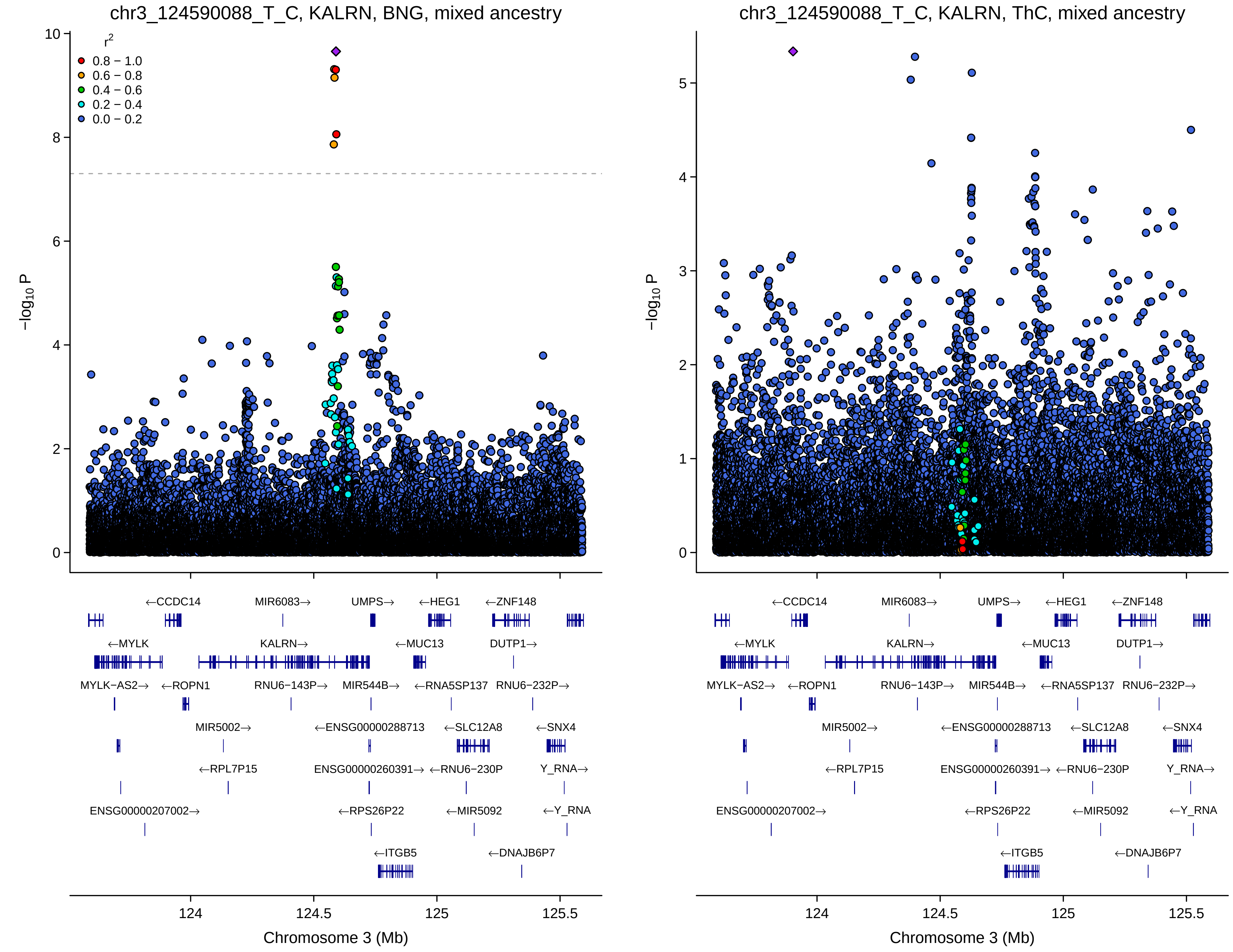

Supplementary Figure 2.2

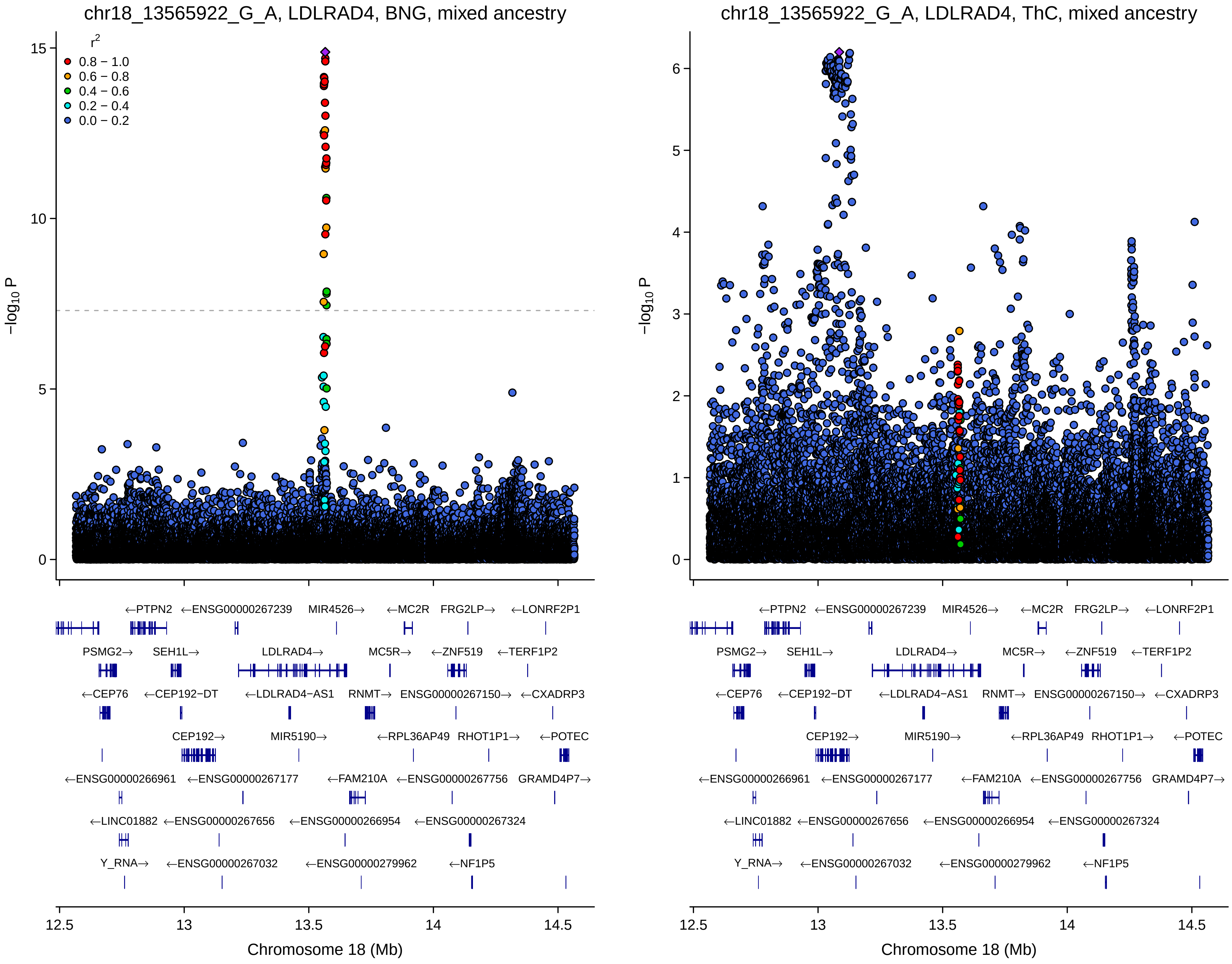

Supplementary Figure 2.2

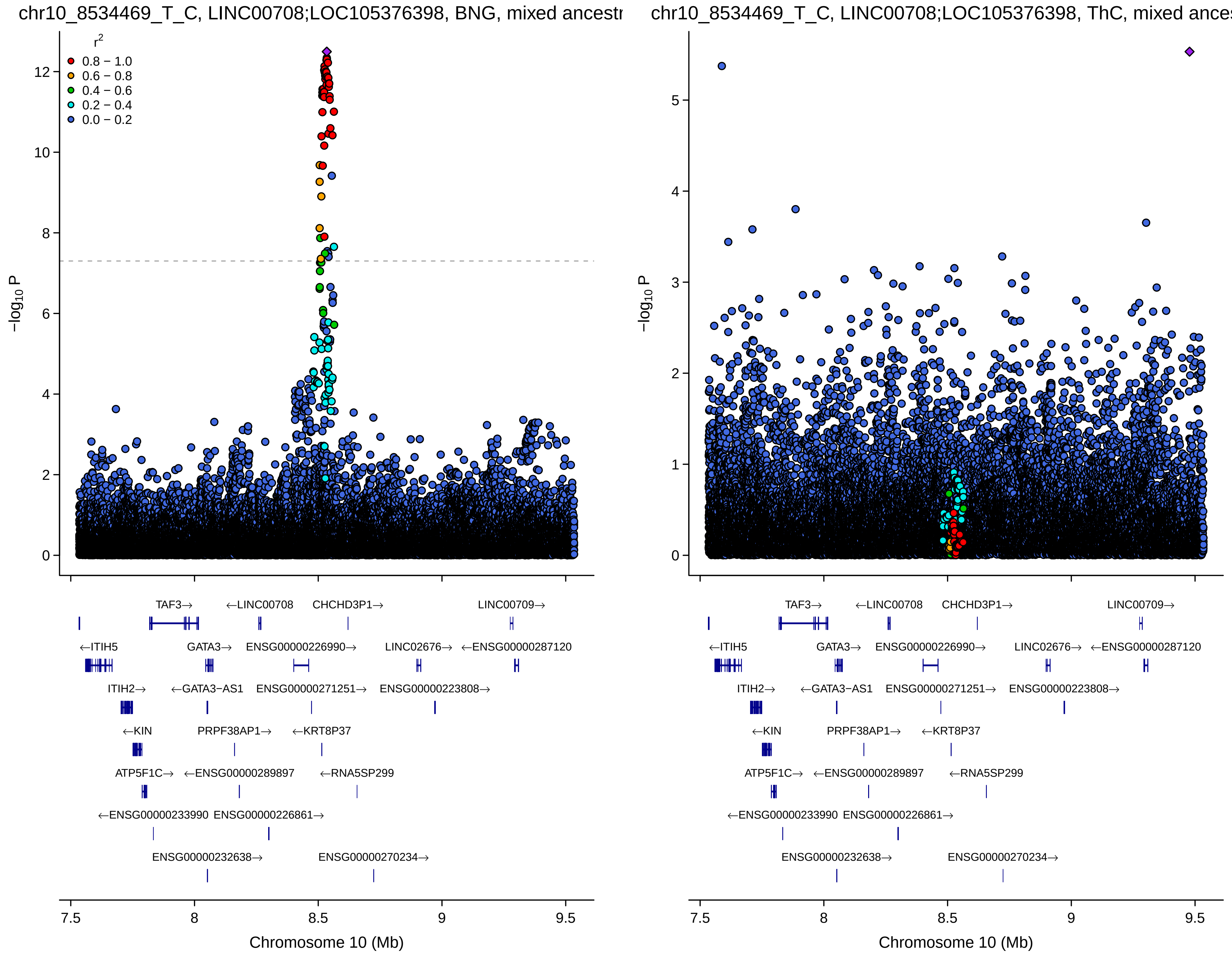

Supplementary Figure 2.2

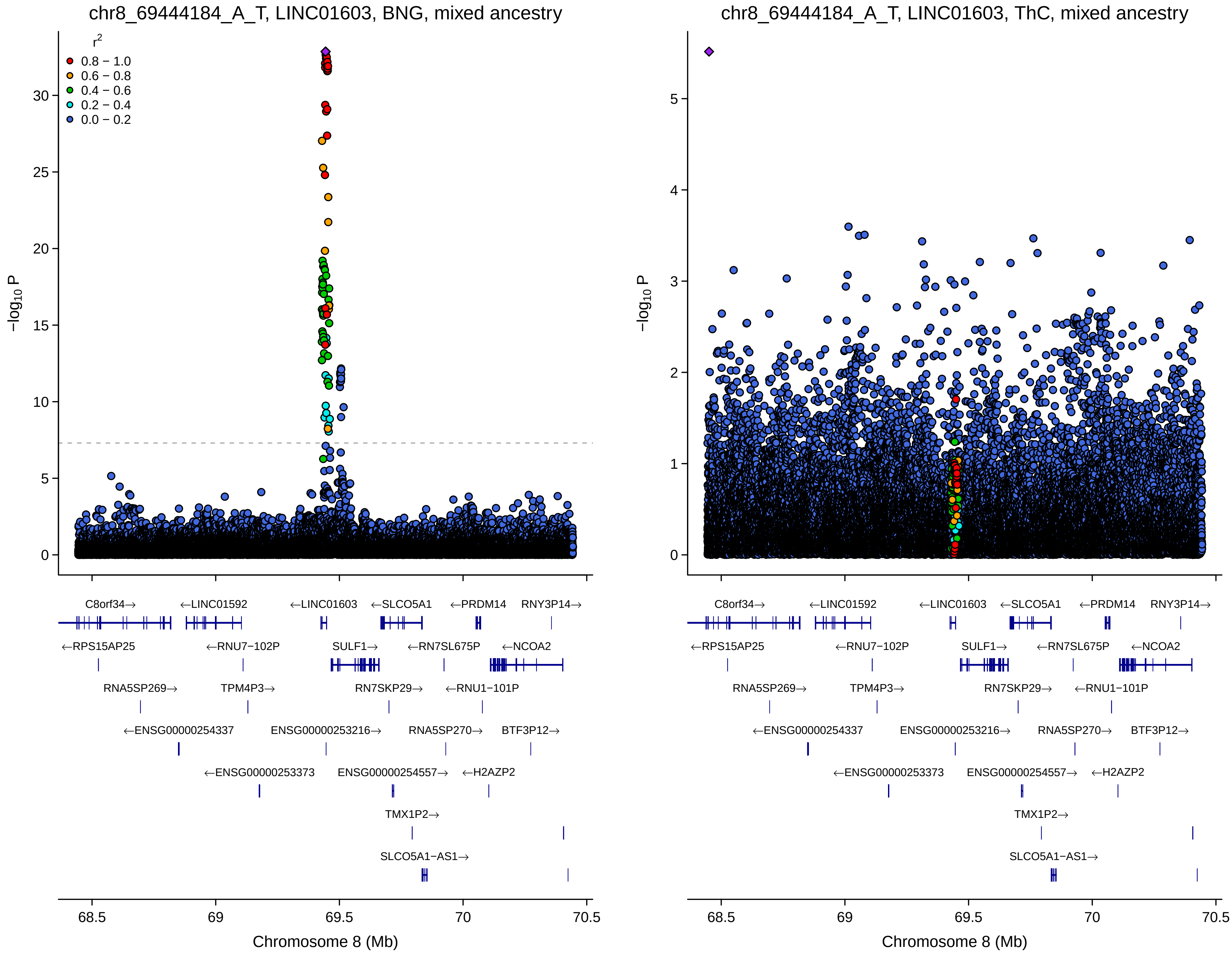

Supplementary Figure 2.2

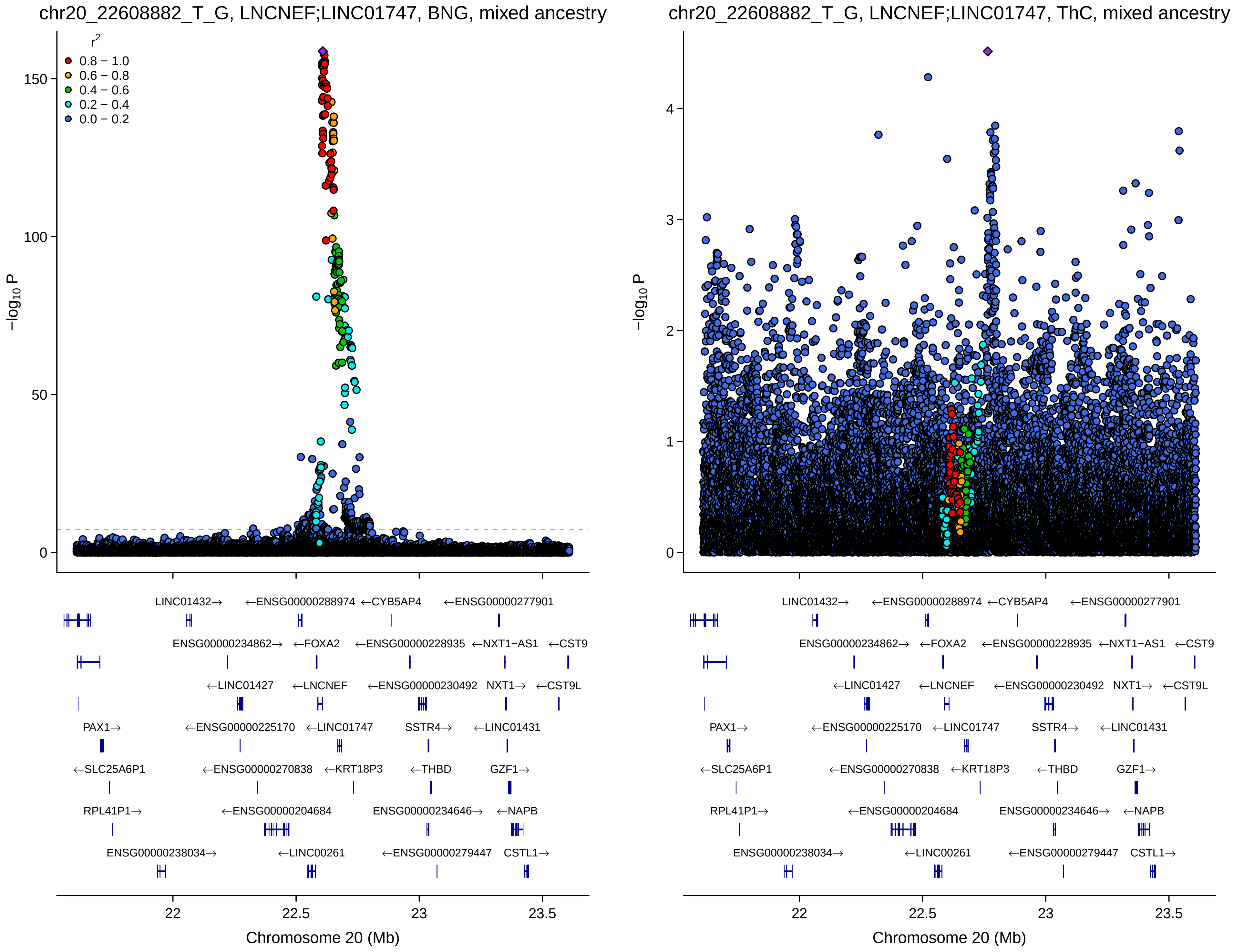

Supplementary Figure 2.2

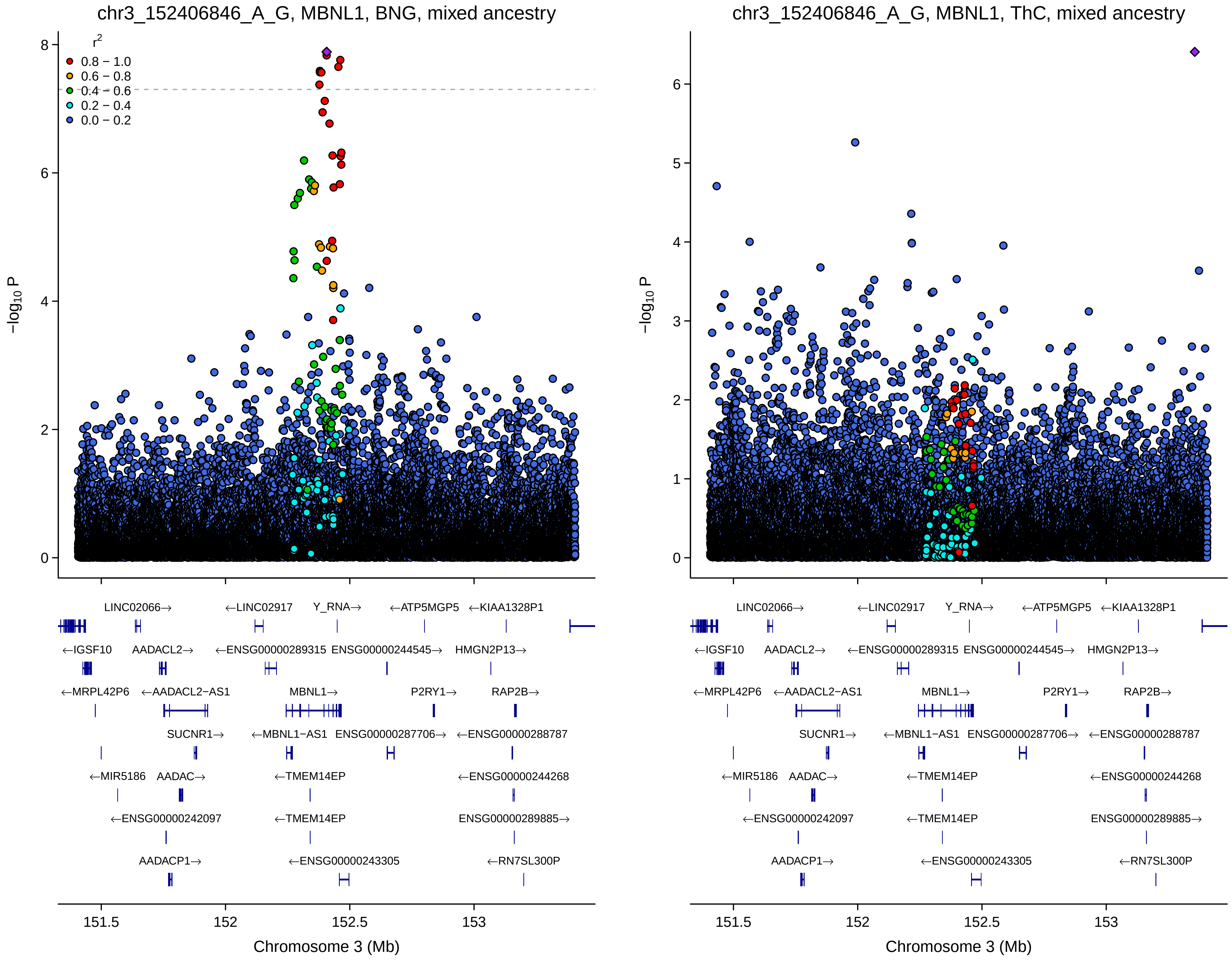

Supplementary Figure 2.2

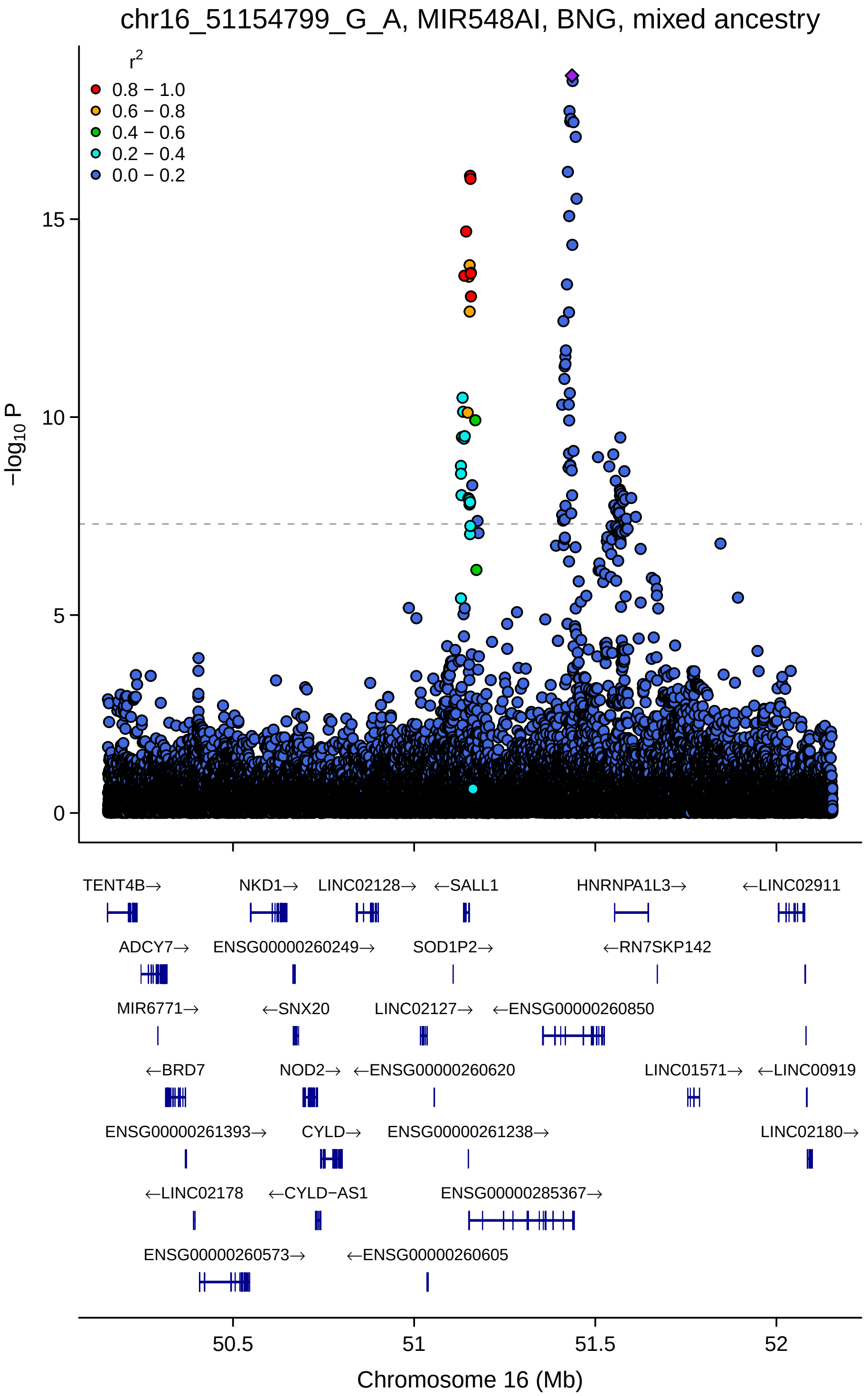

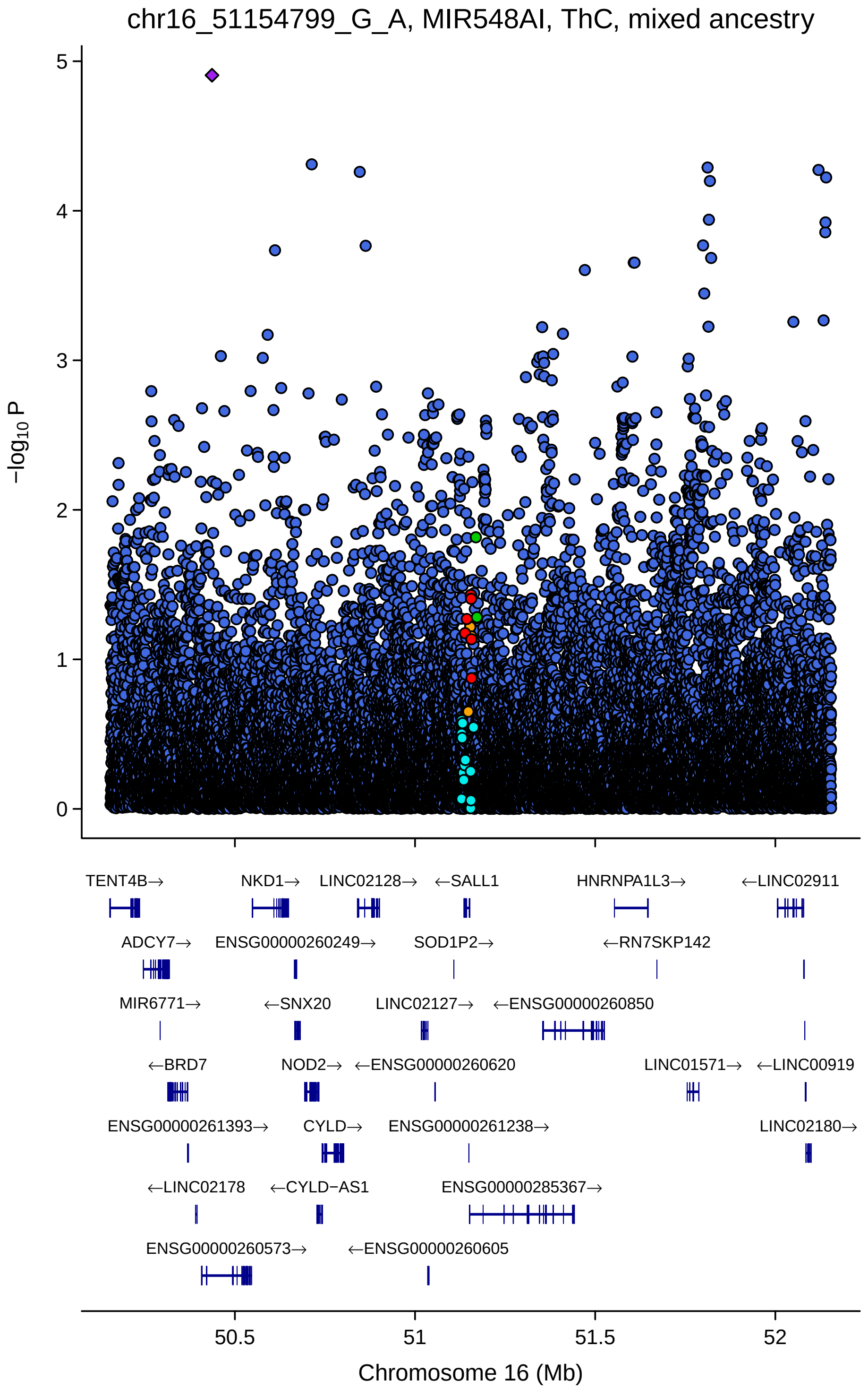

Supplementary Figure 2.2

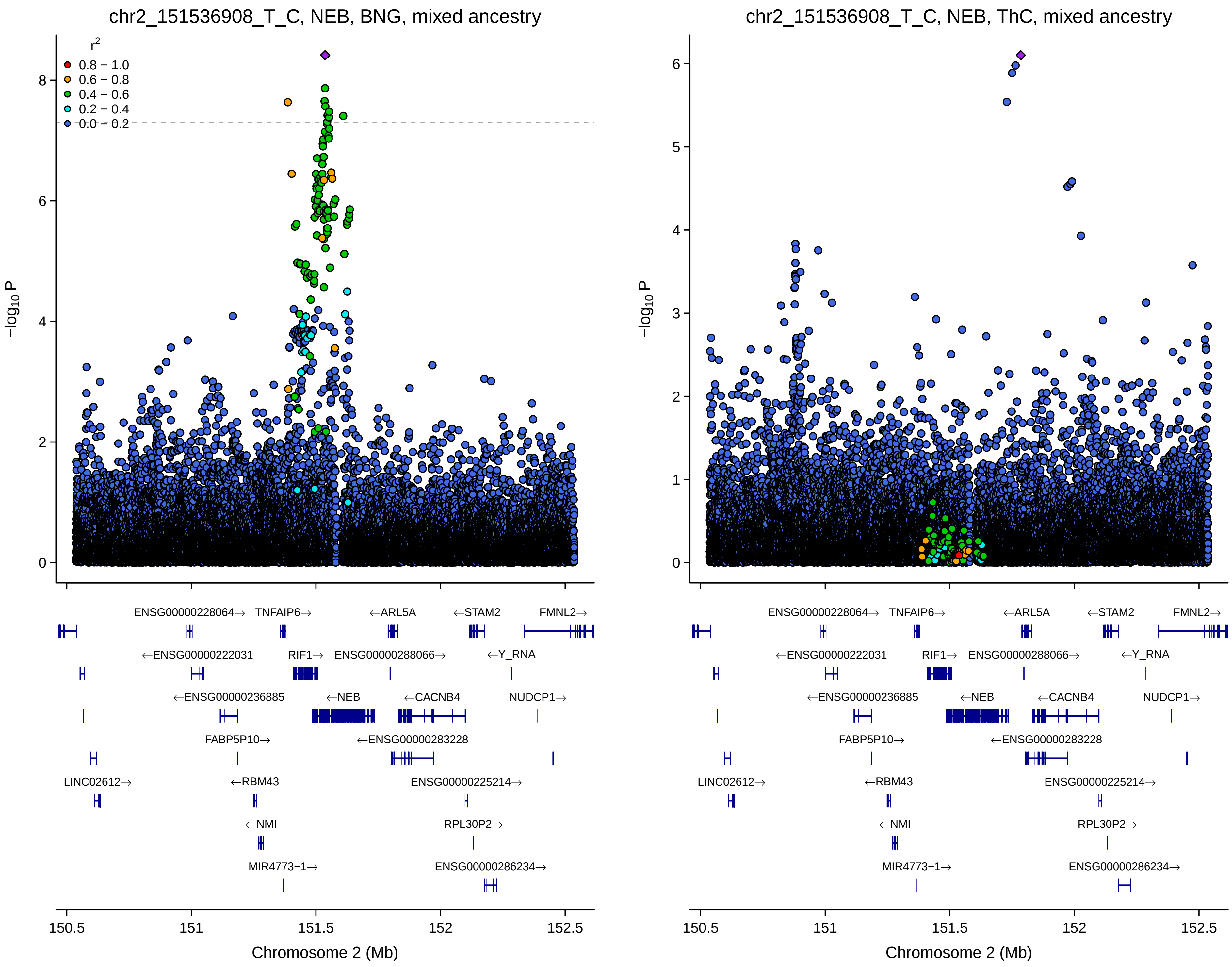

Supplementary Figure 2.2

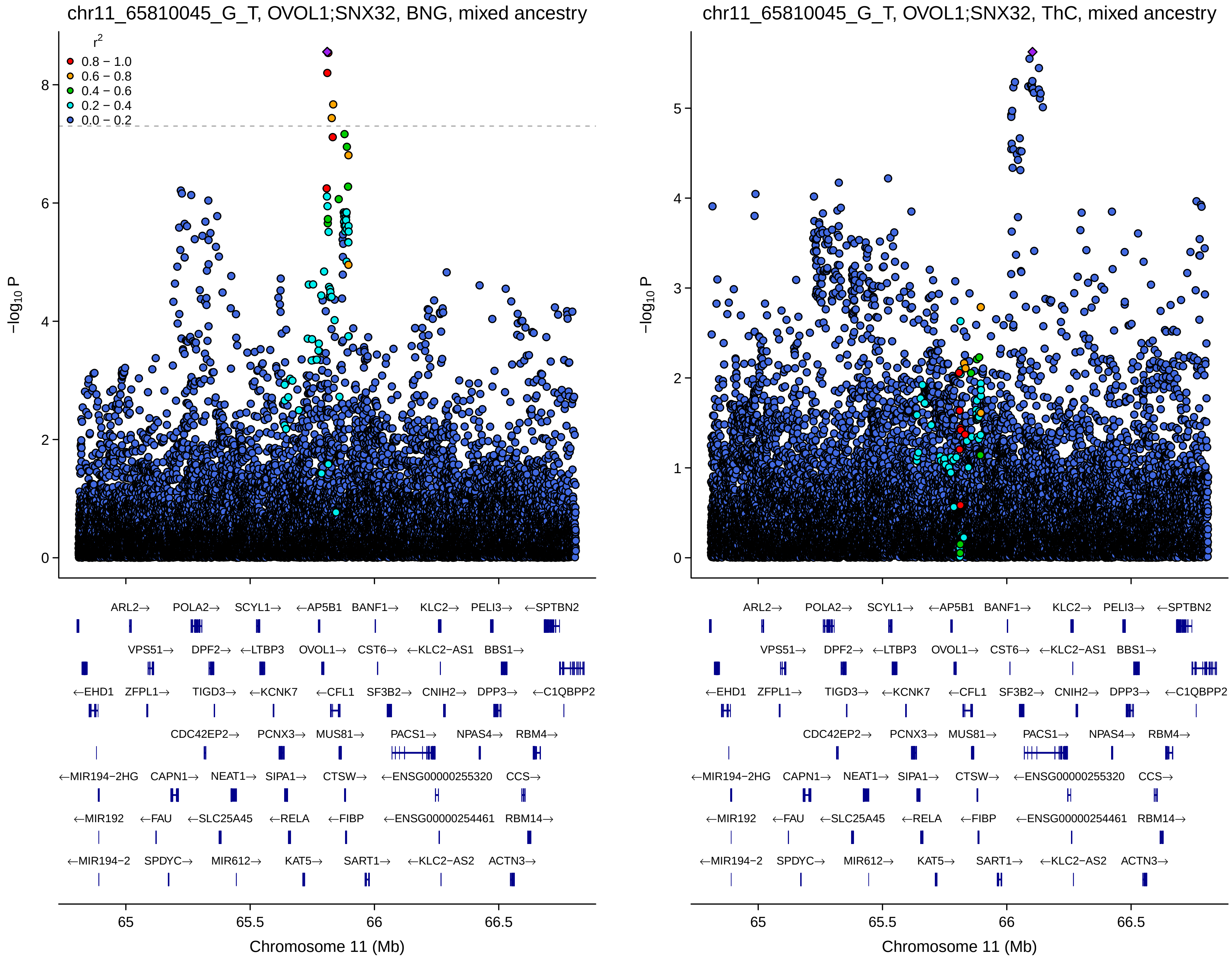

Supplementary Figure 2.2

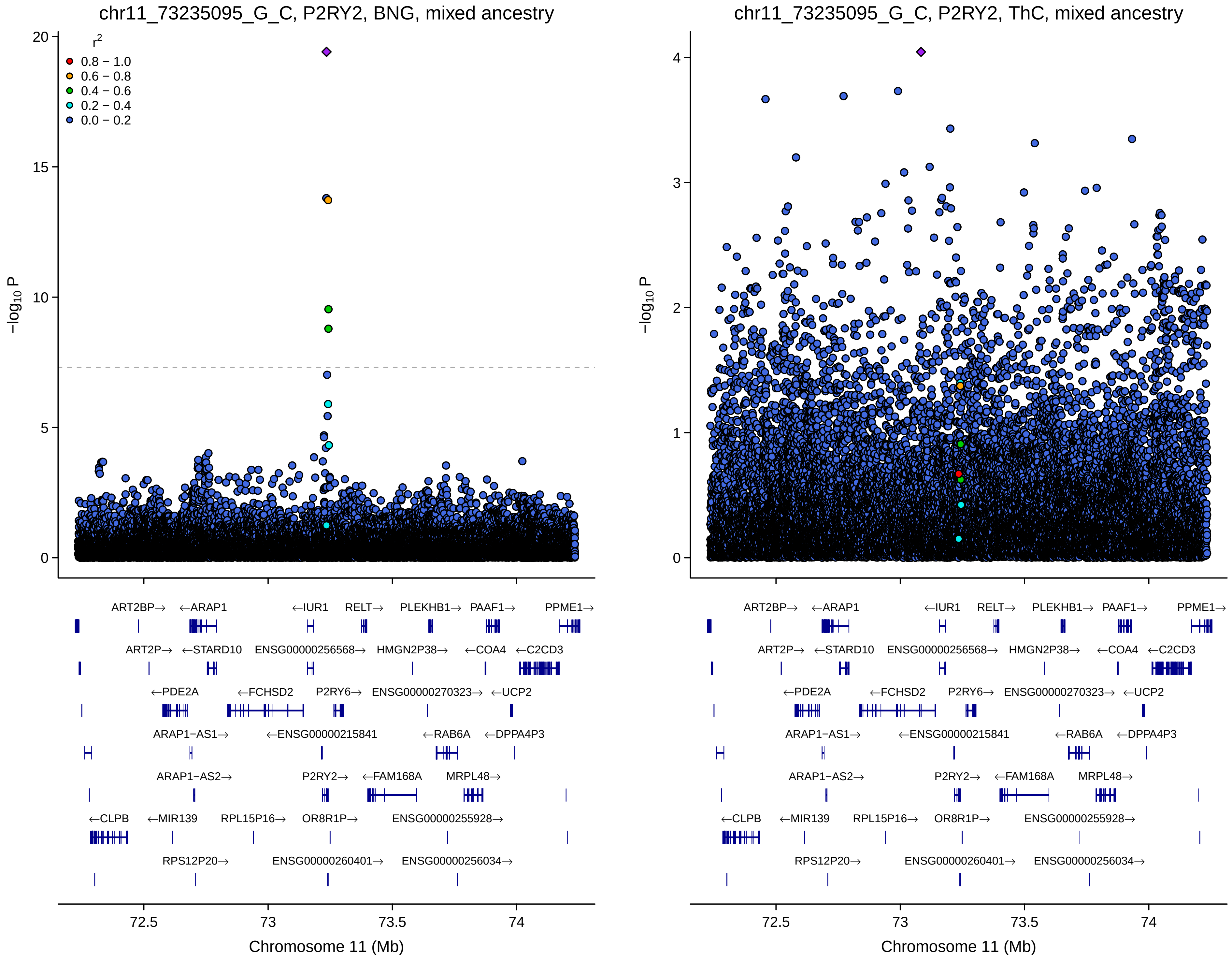

Supplementary Figure 2.2

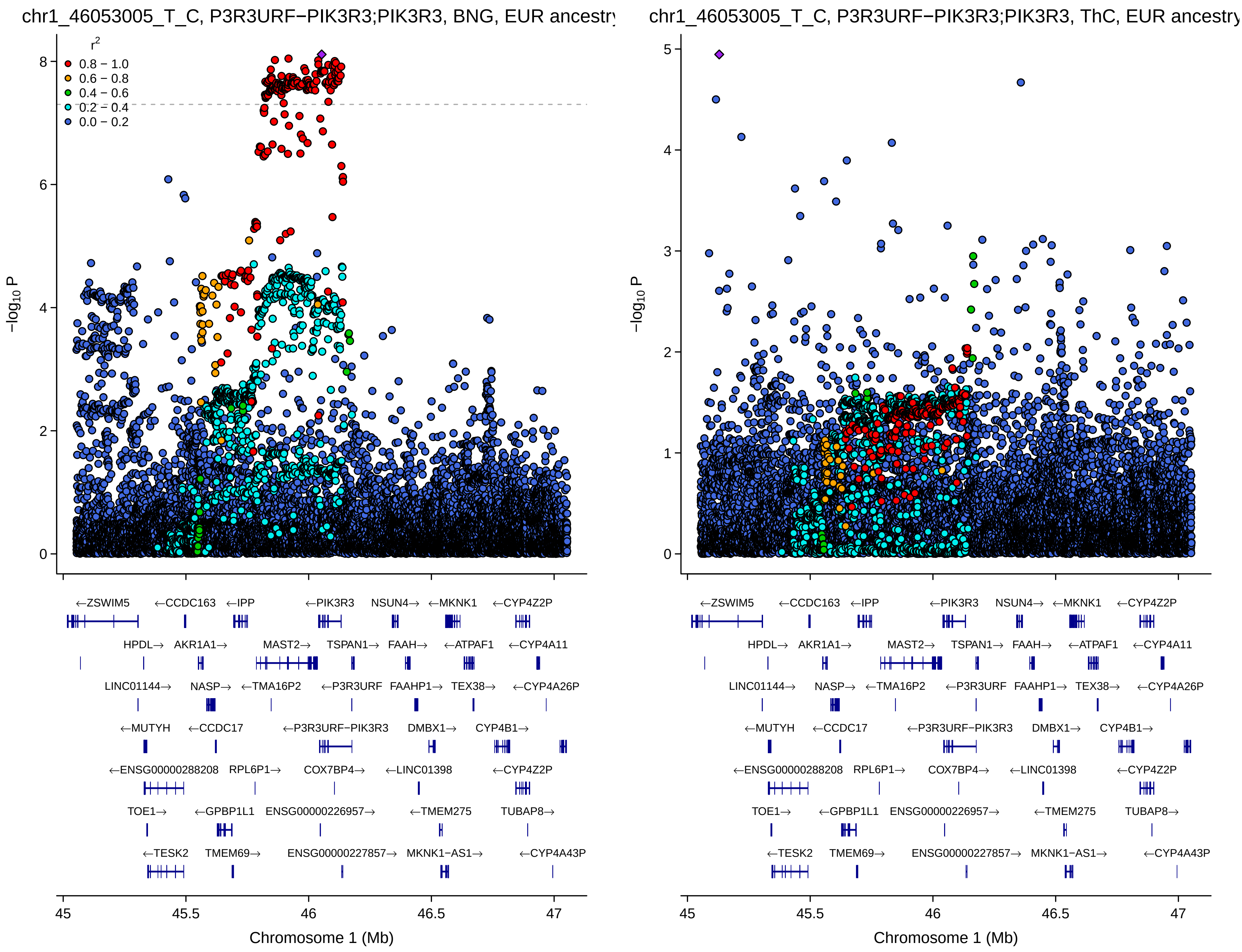

Supplementary Figure 2.2

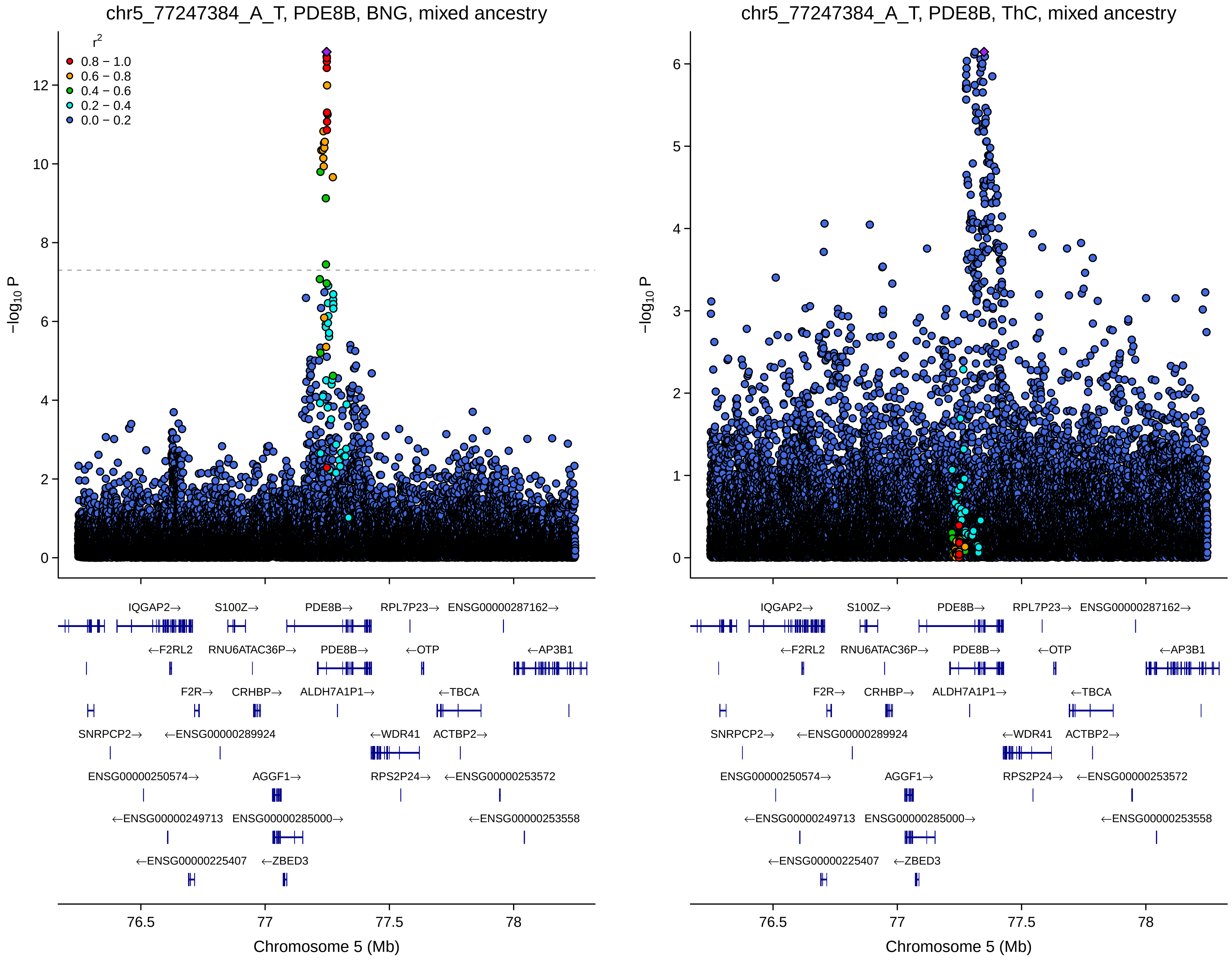

Supplementary Figure 2.2

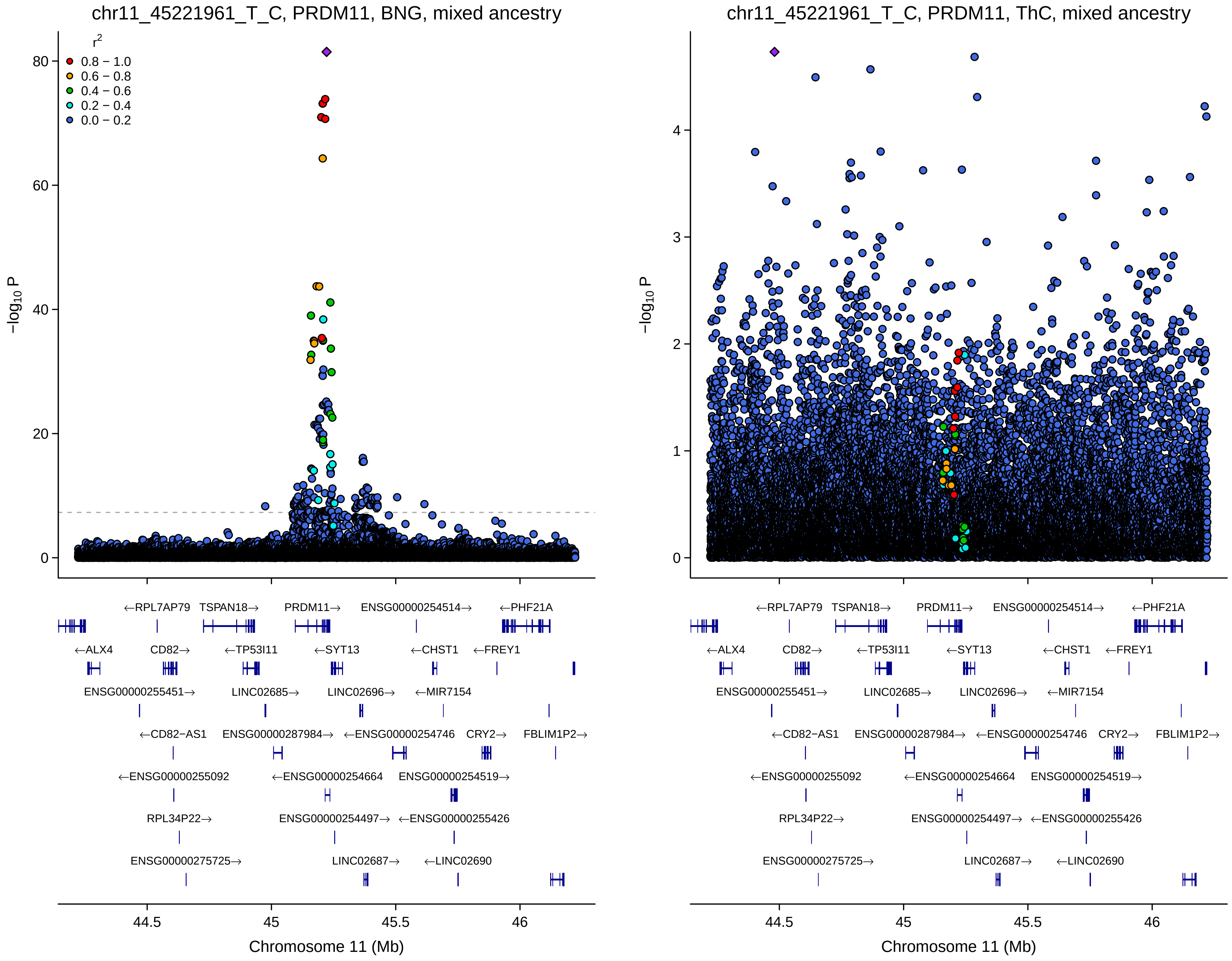

Supplementary Figure 2.2

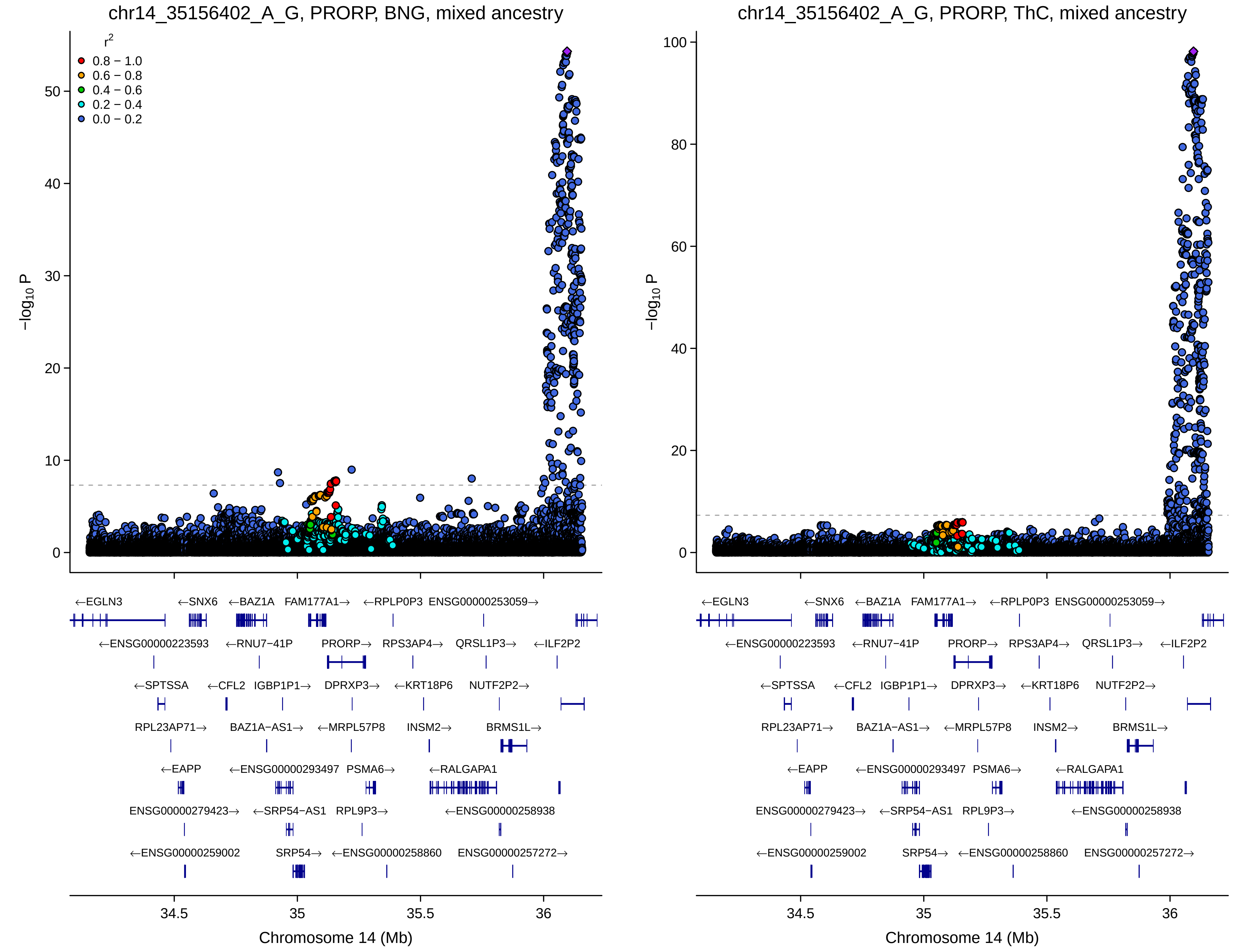

Supplementary Figure 2.2

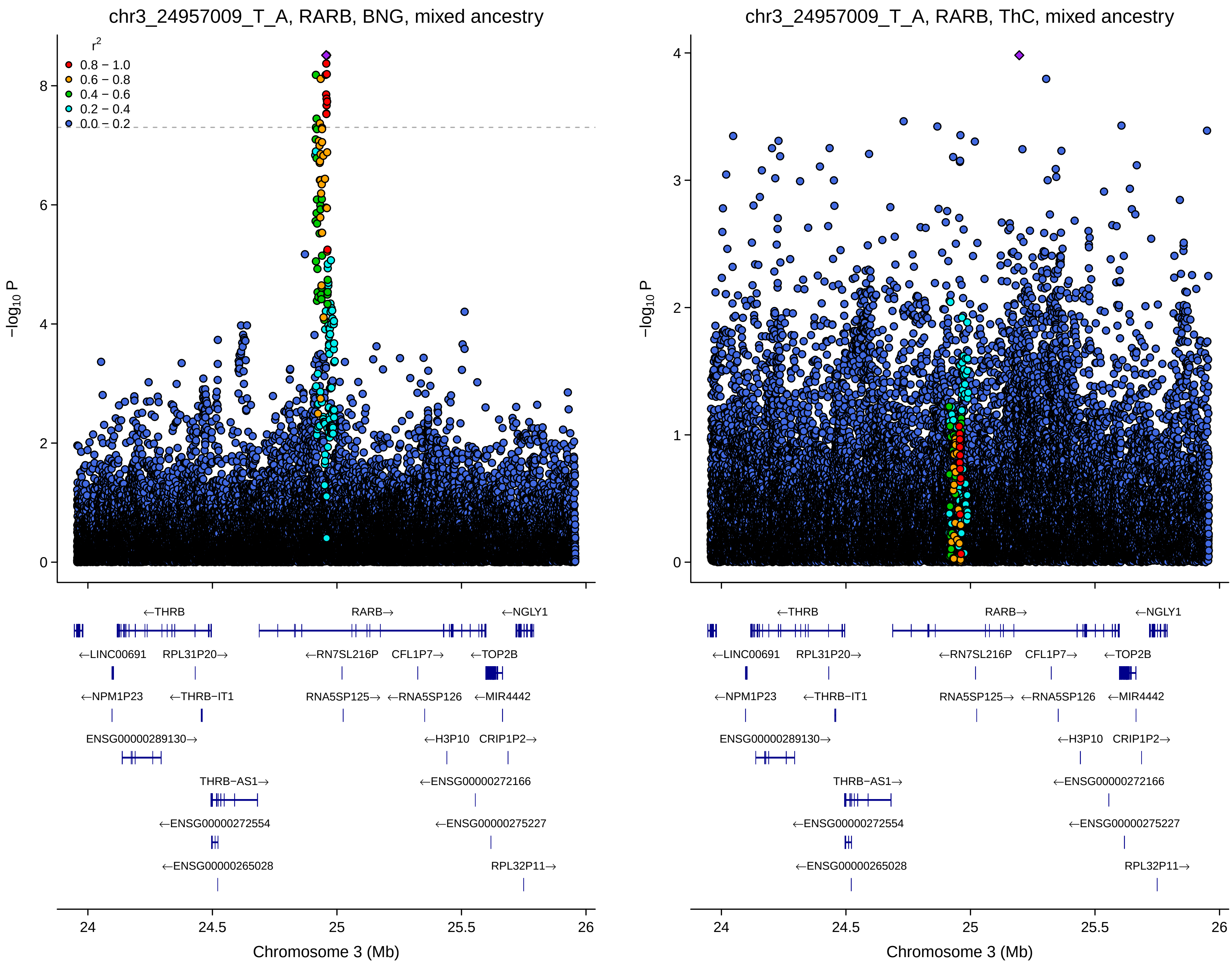

Supplementary Figure 2.2

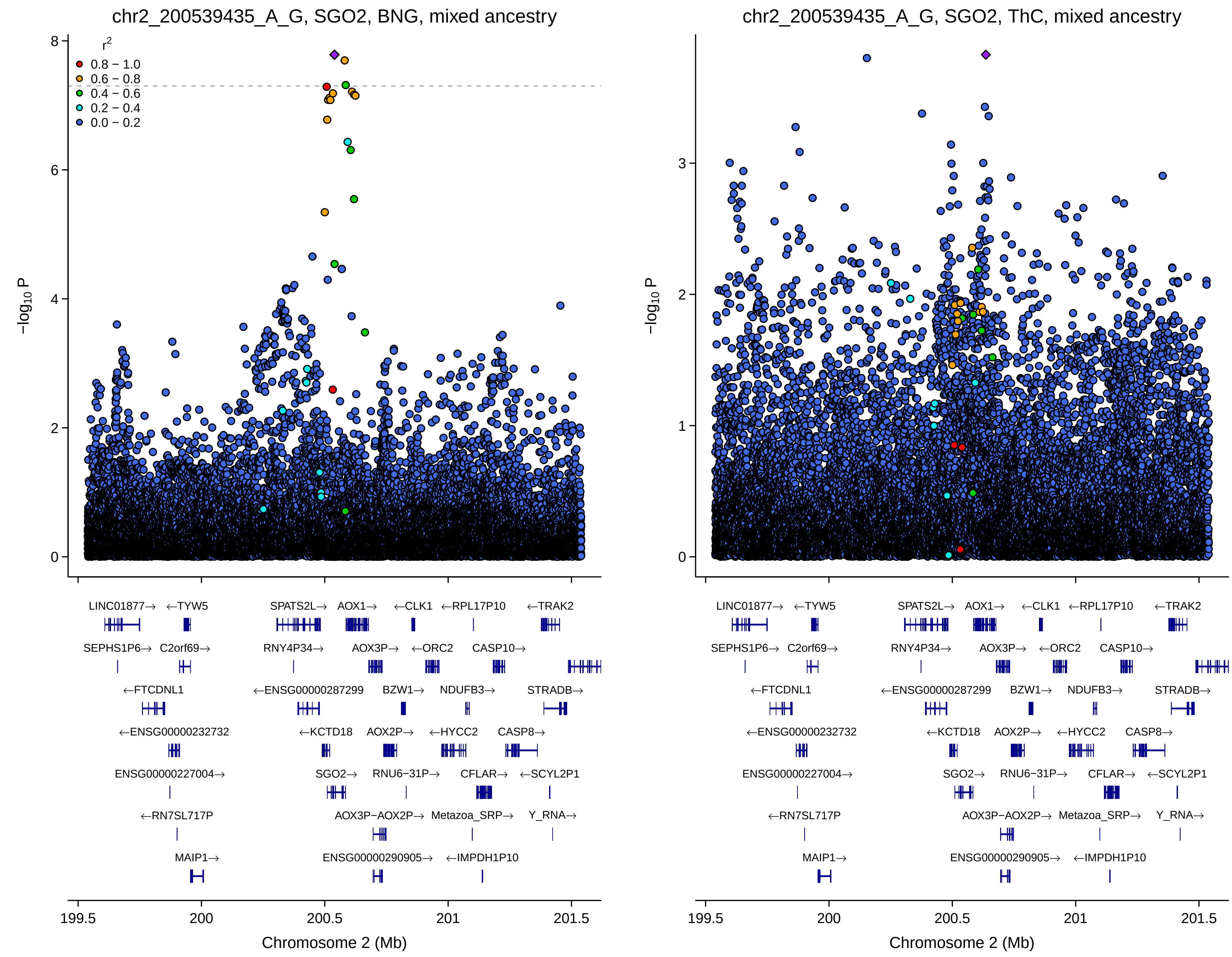

Supplementary Figure 2.2

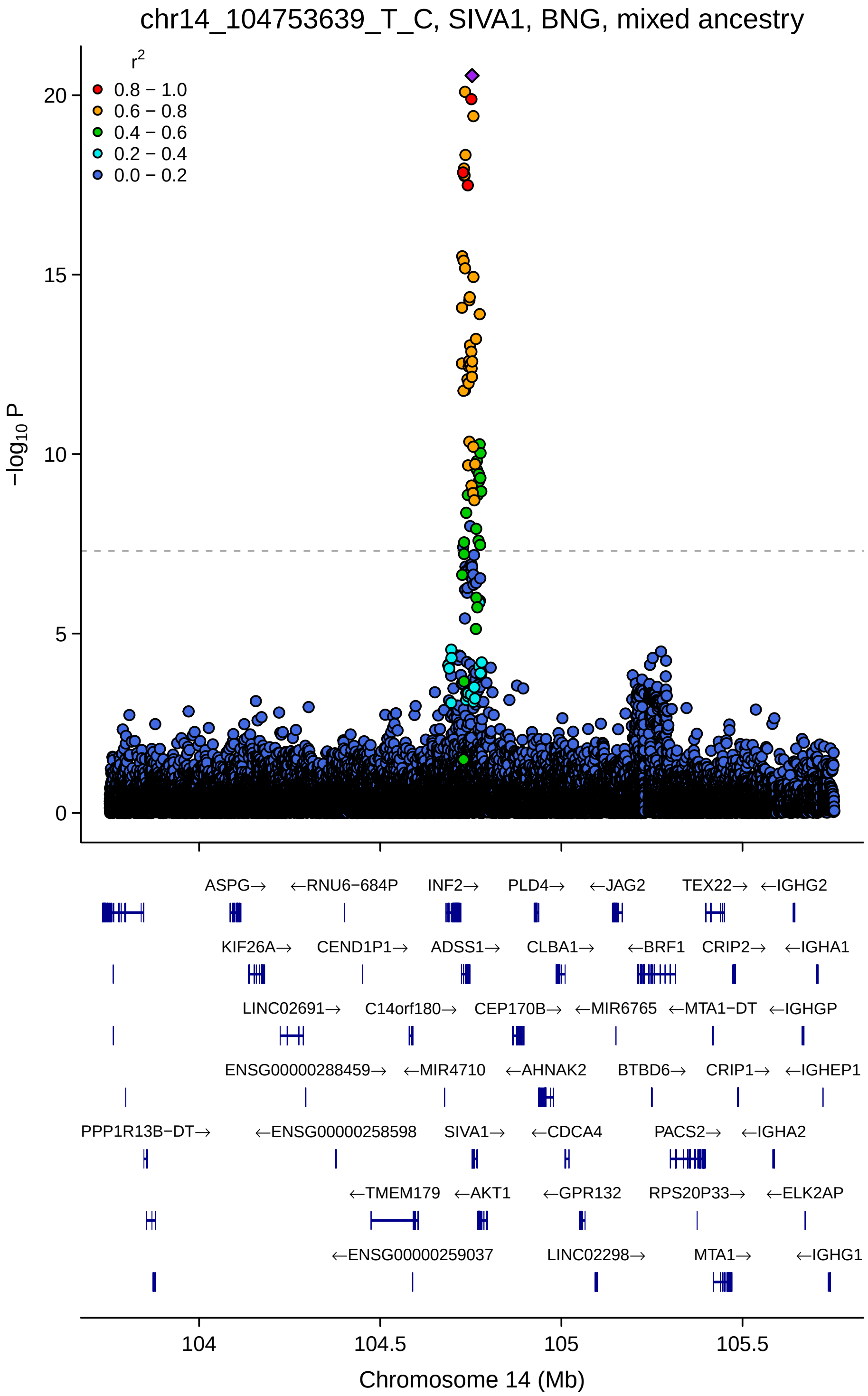

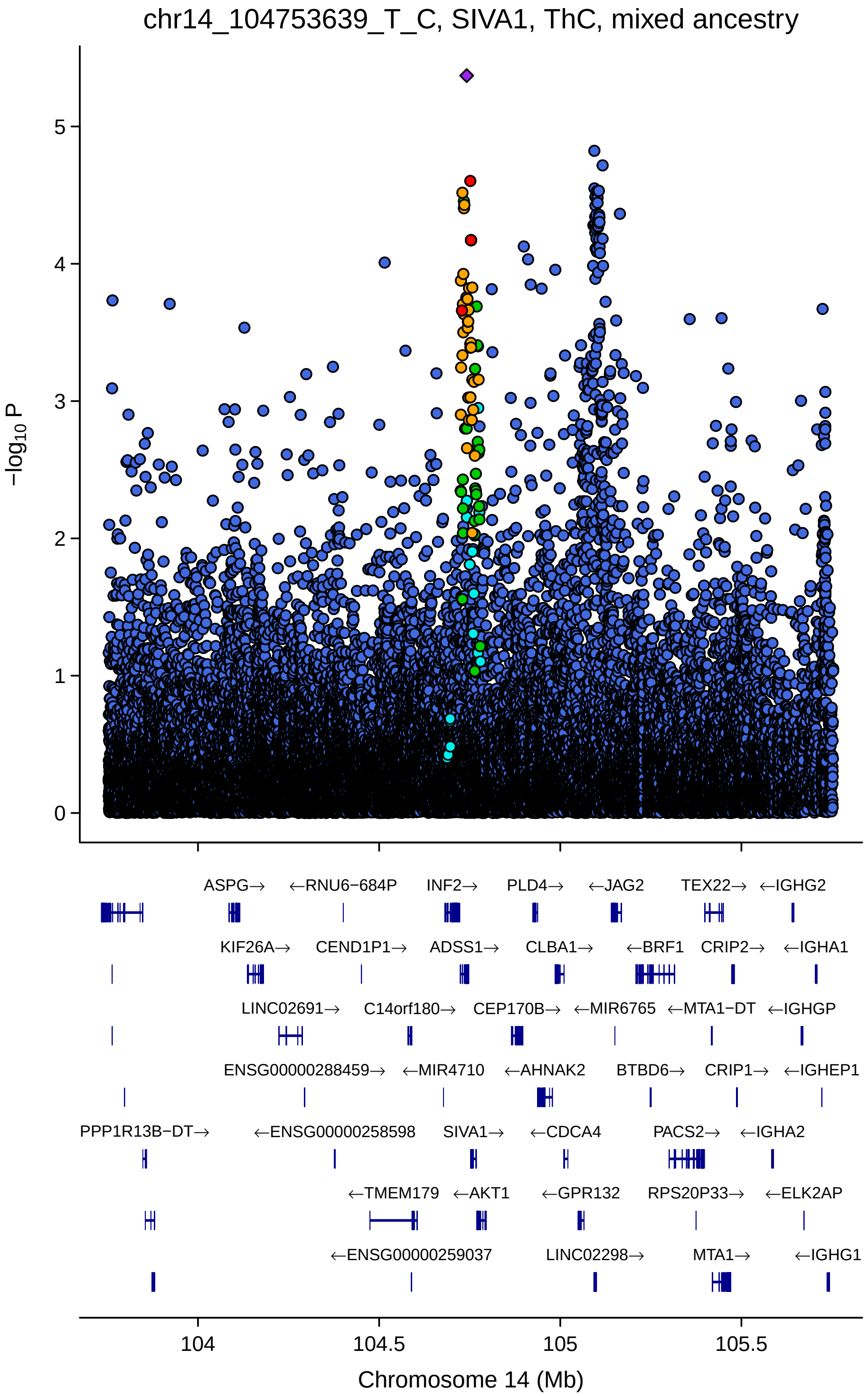

Supplementary Figure 2.2

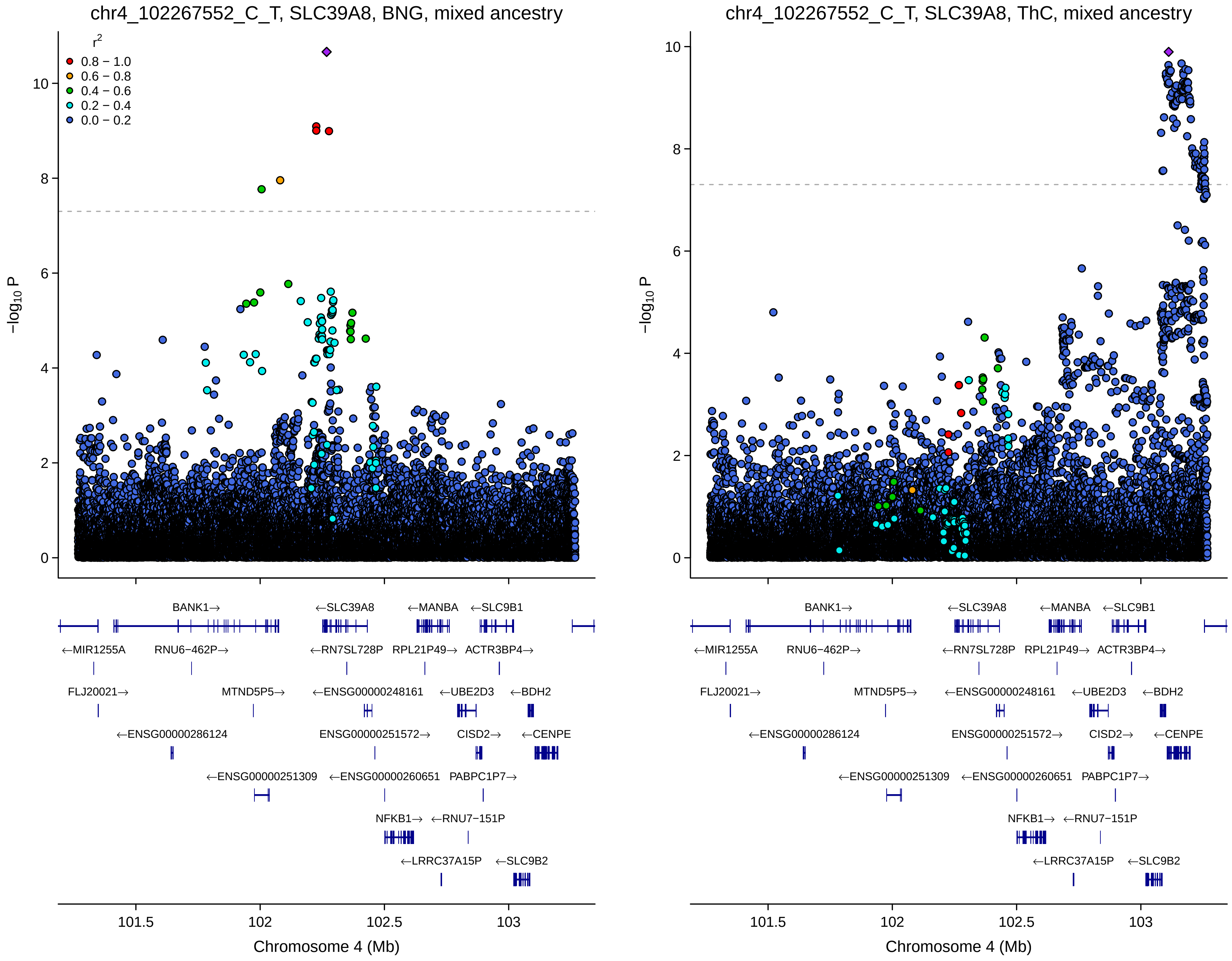

Supplementary Figure 2.2

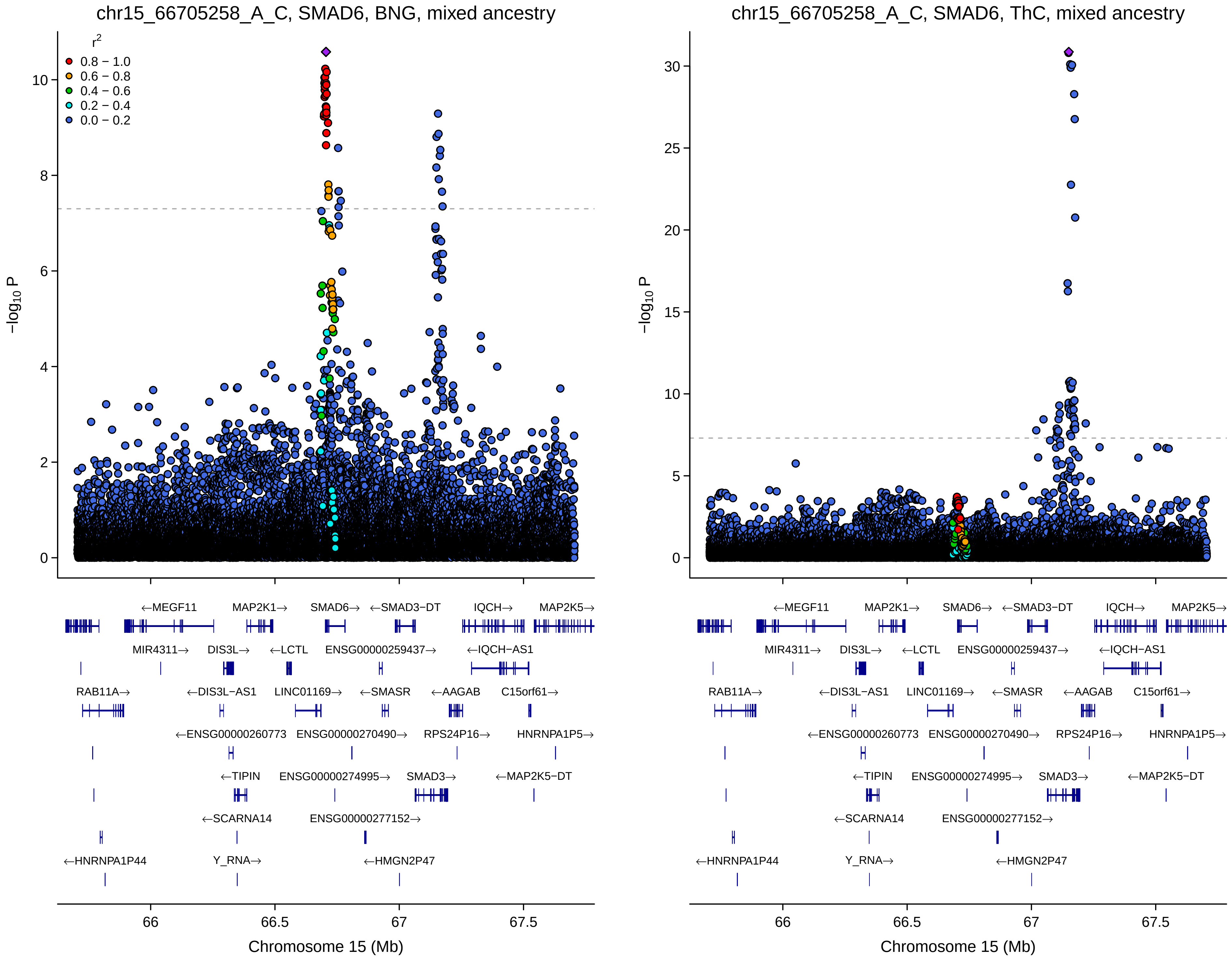

Supplementary Figure 2.2

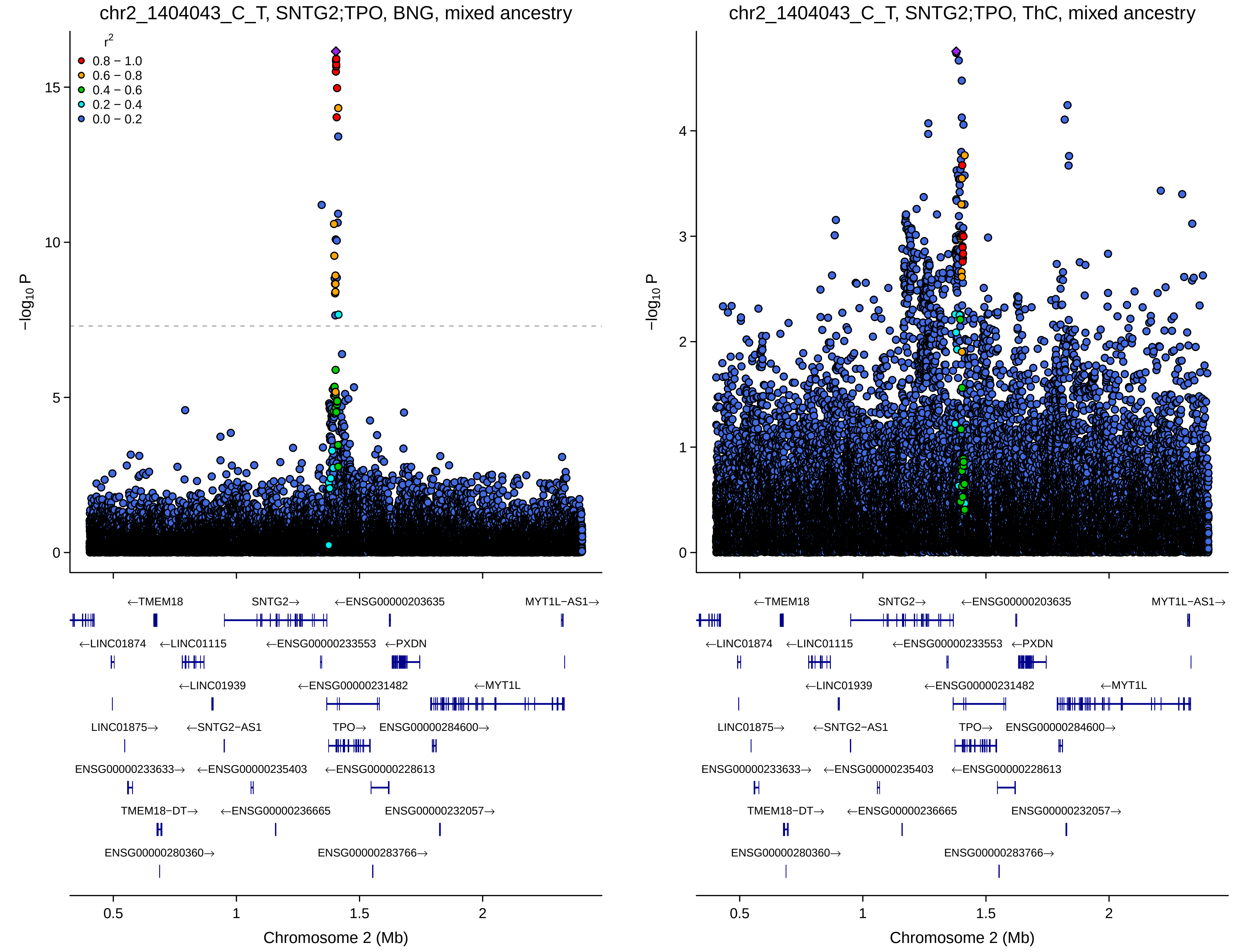

Supplementary Figure 2.2

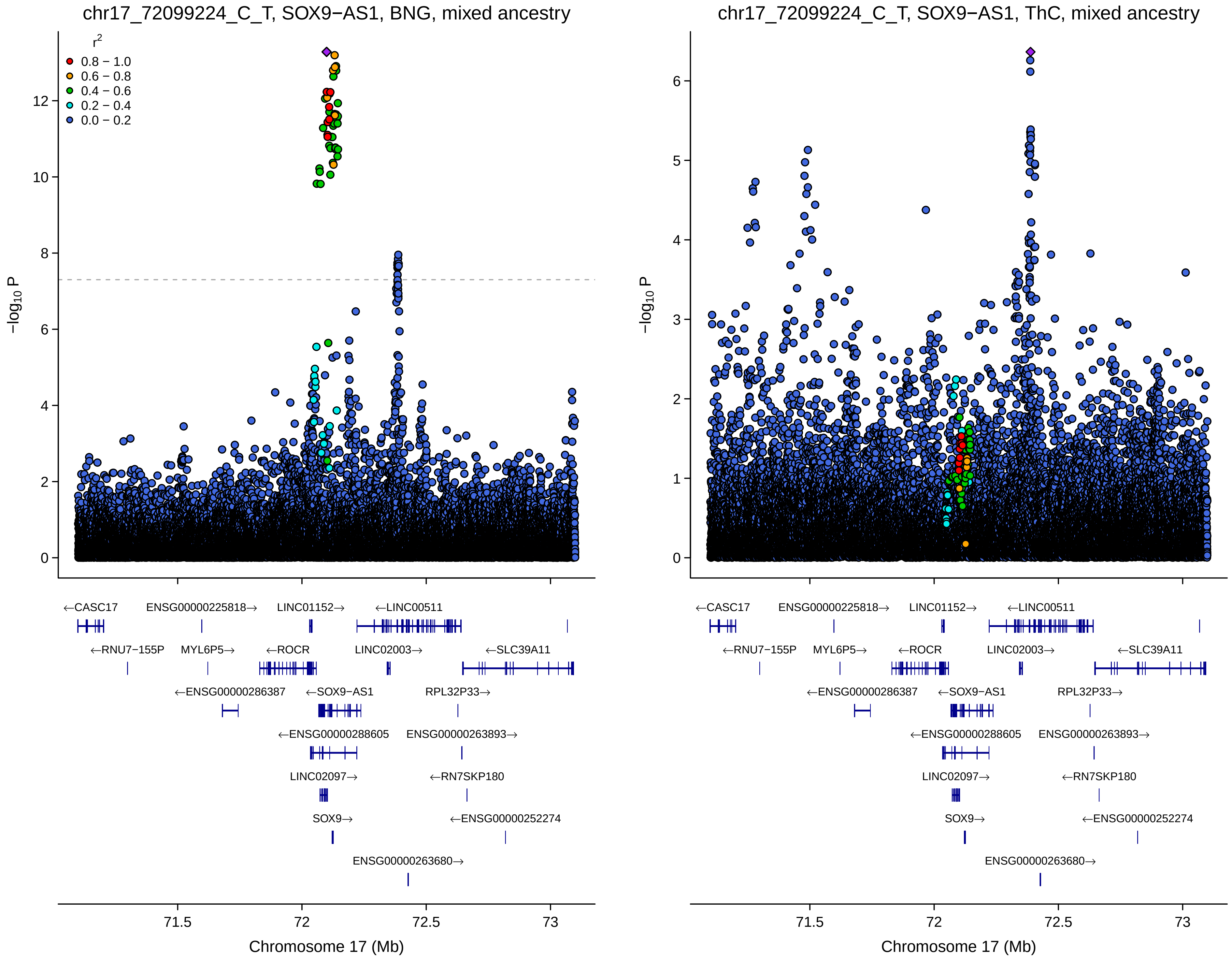

Supplementary Figure 2.2

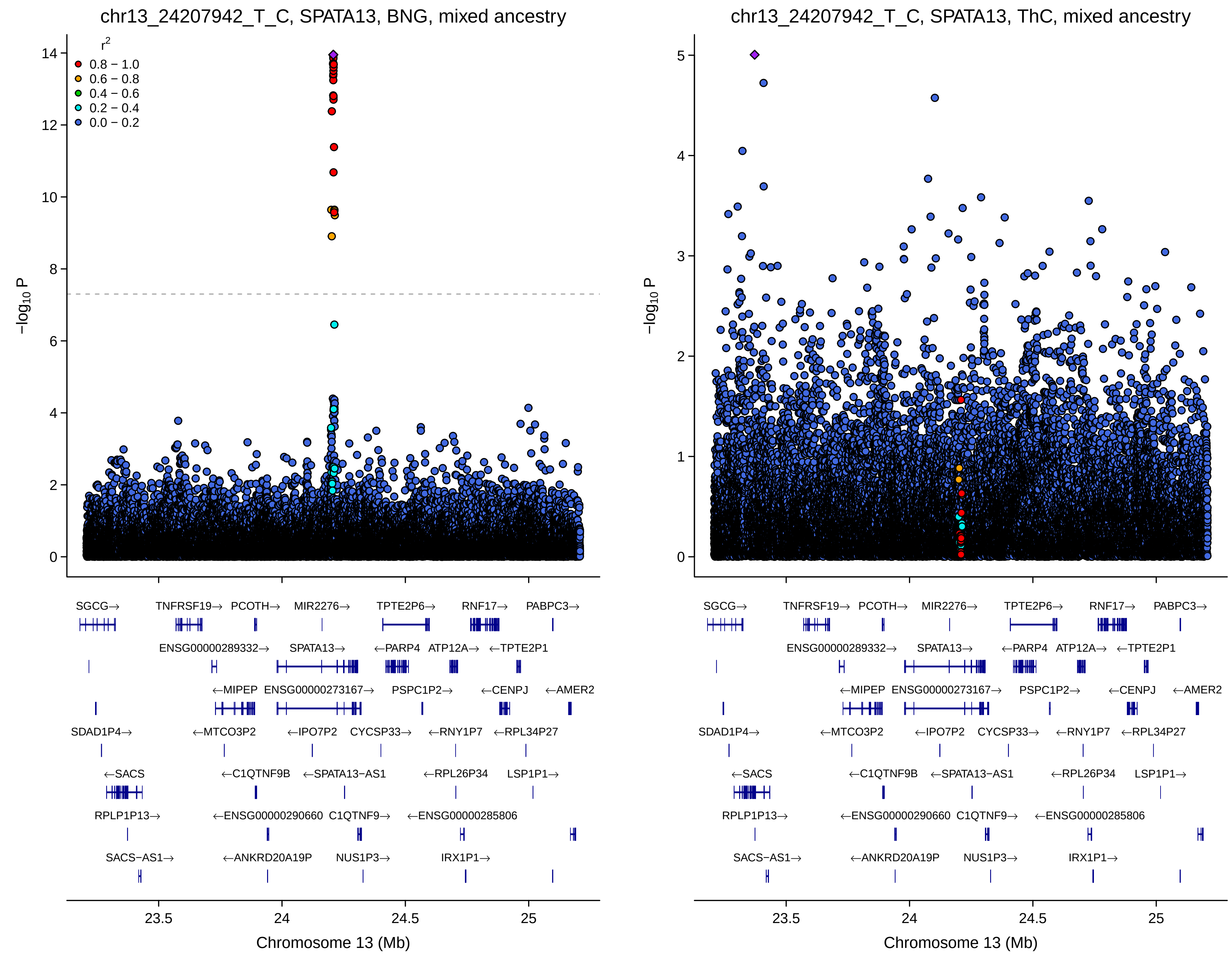

Supplementary Figure 2.2

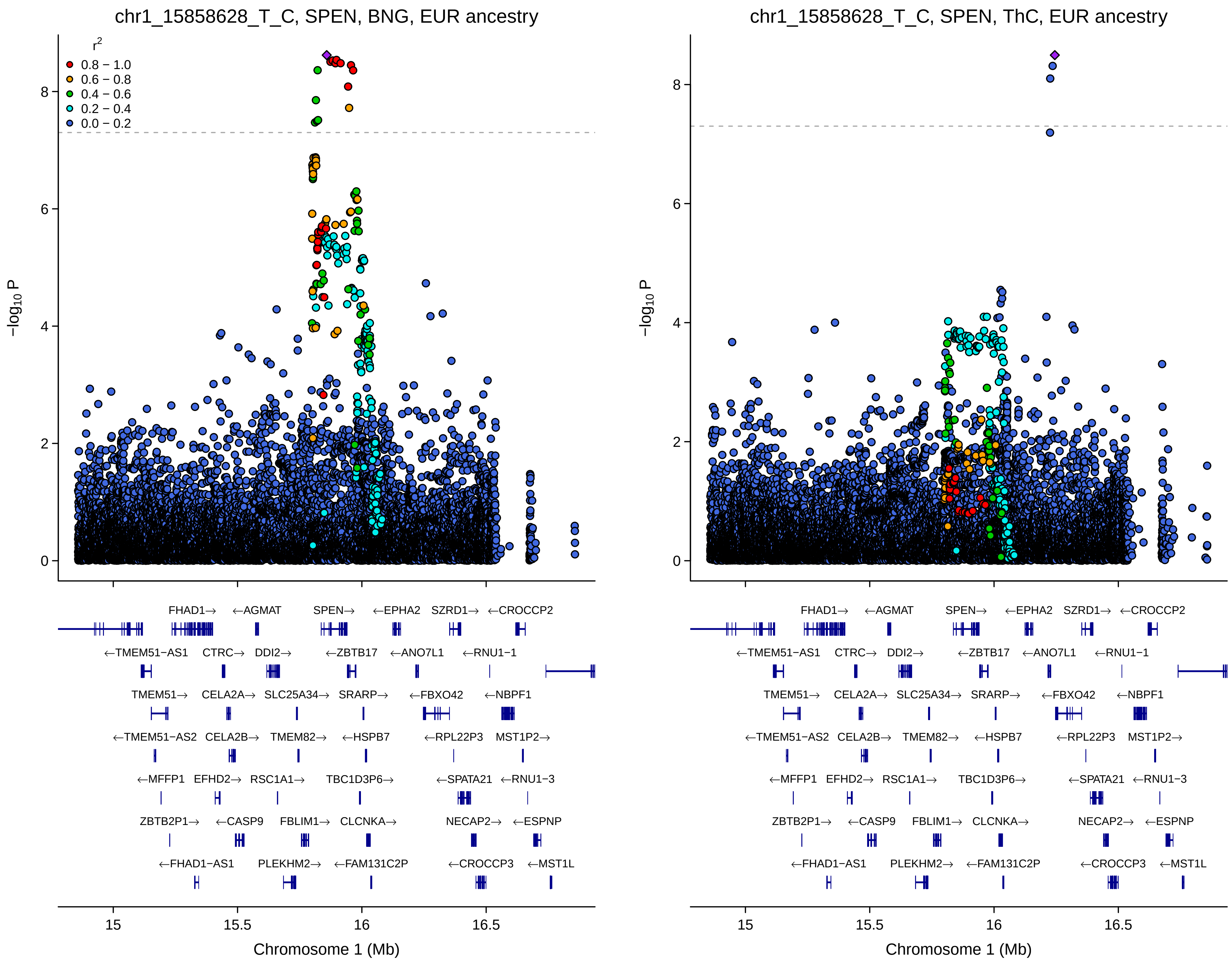

Supplementary Figure 2.2

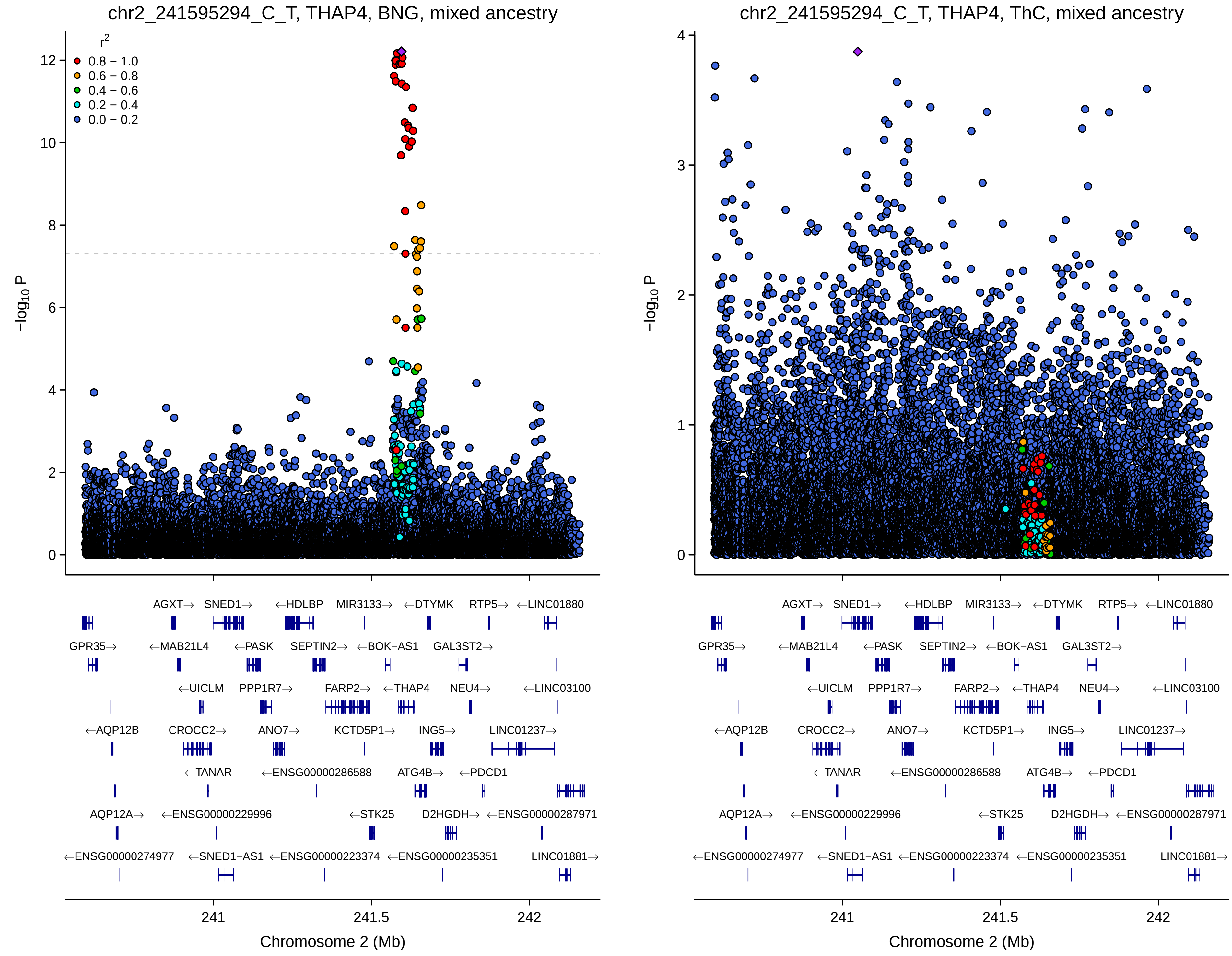

Supplementary Figure 2.2

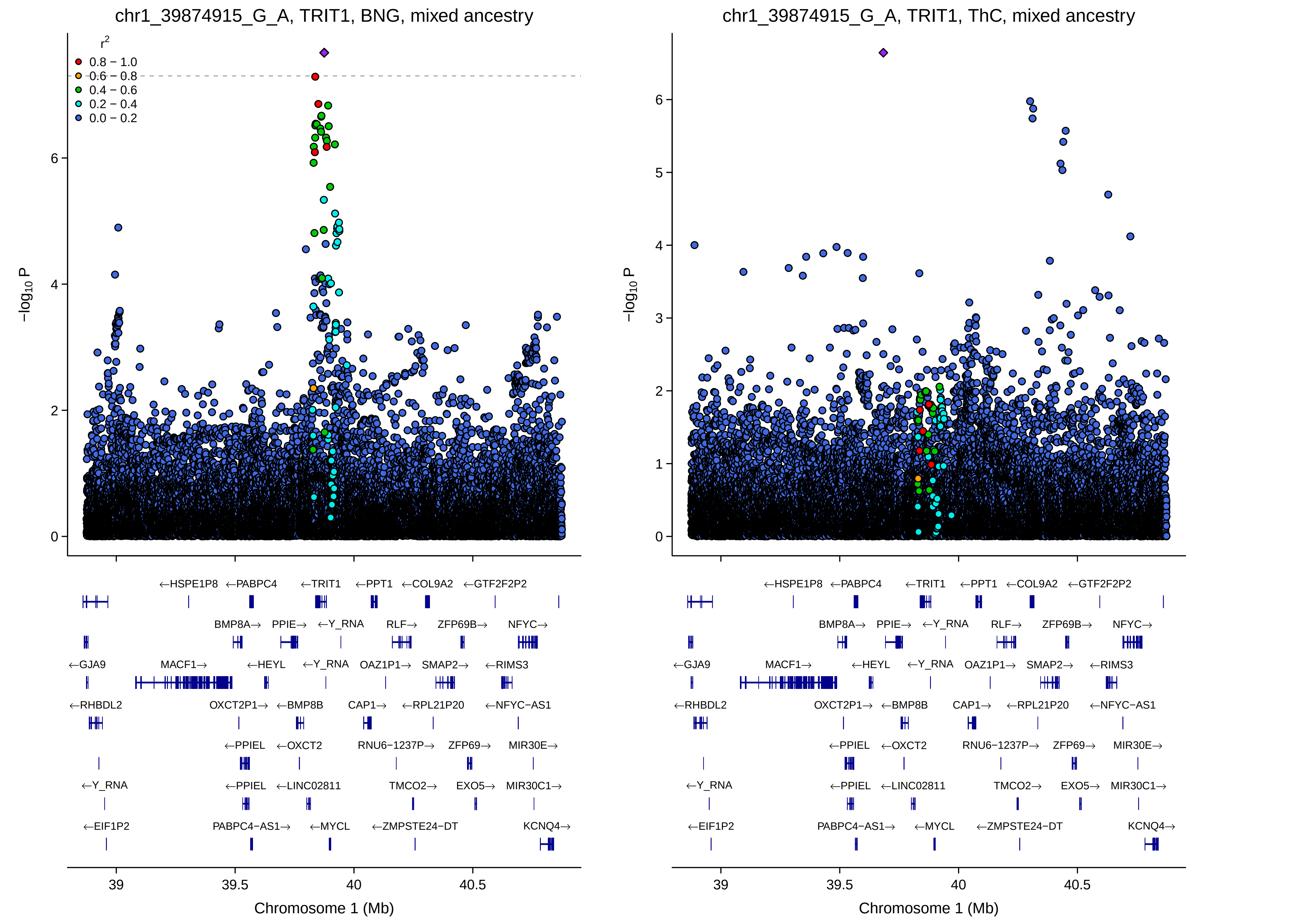

Supplementary Figure 2.2

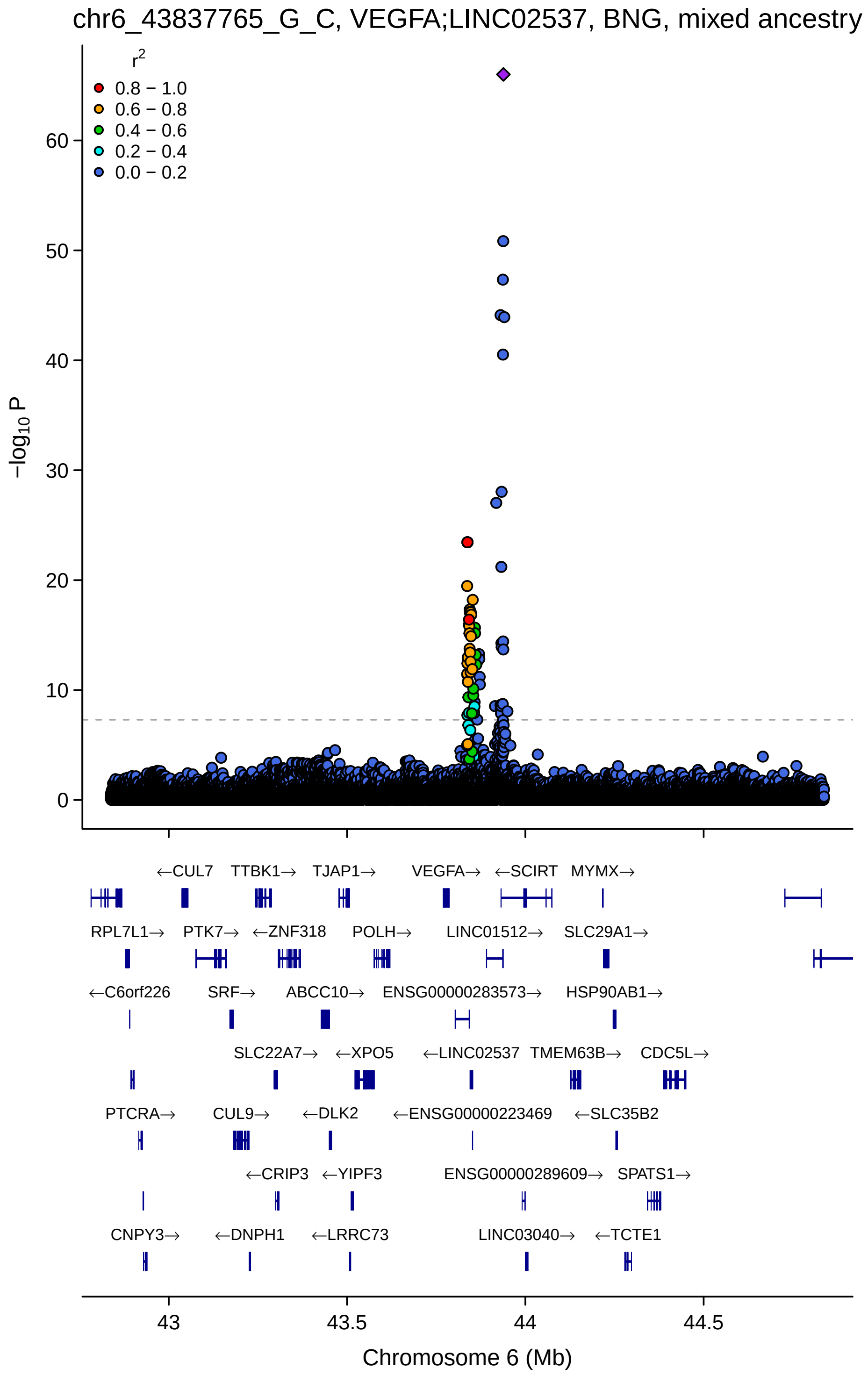

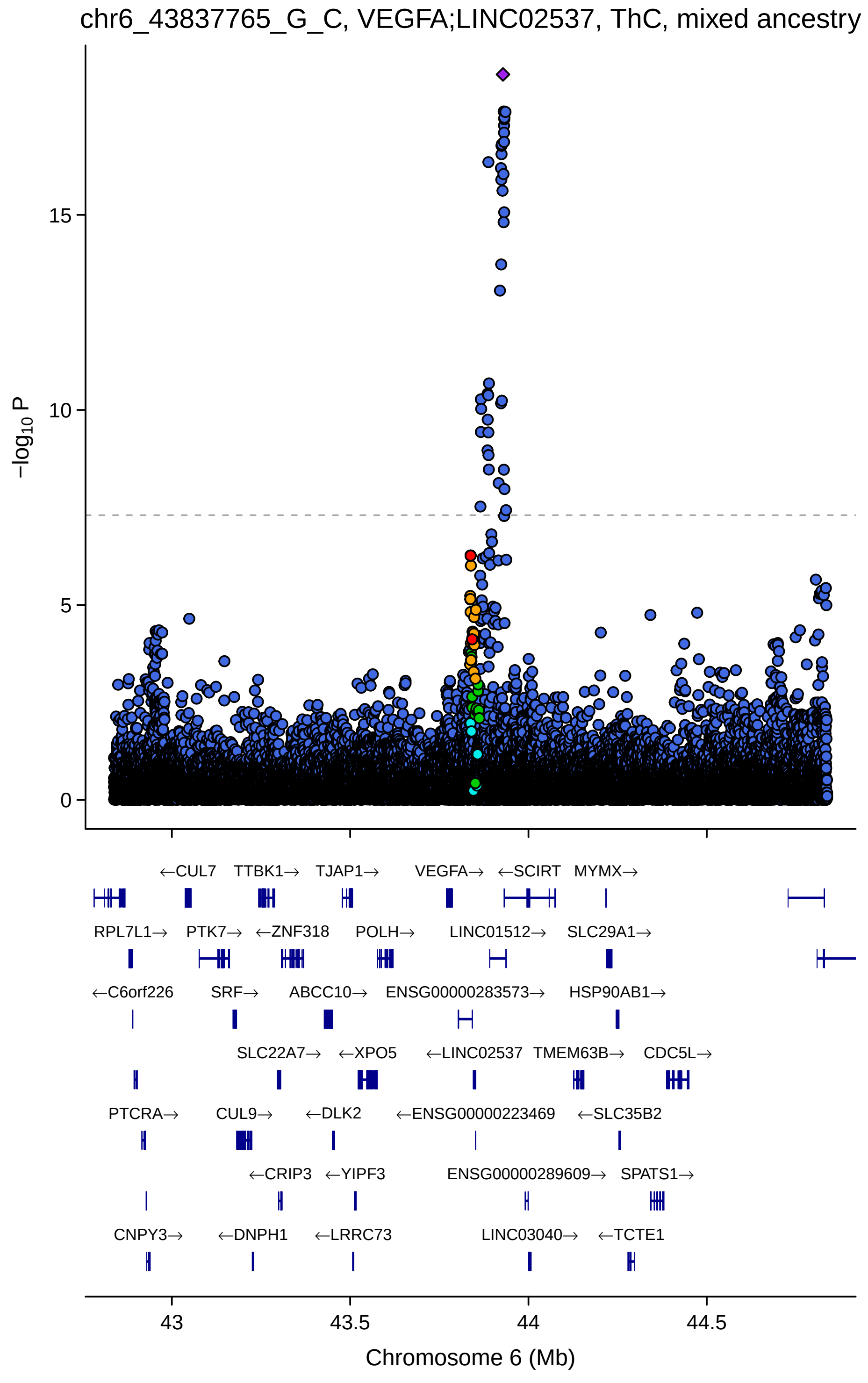

Supplementary Figure 2.2

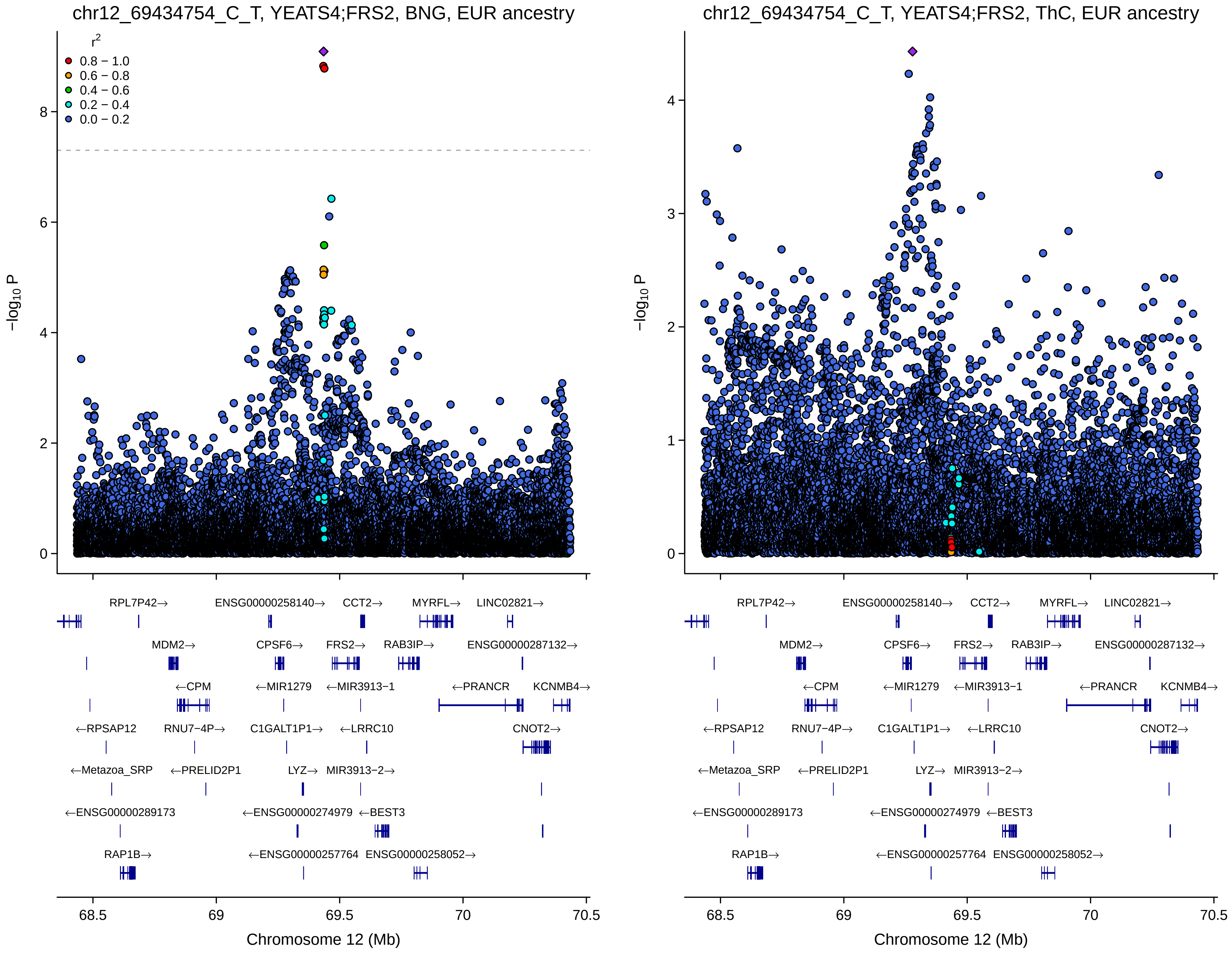

Supplementary Figure 2.3. Locus plots for loci significantly associated with both benign and malignant thyroid nodules. The left plot displays -log10(p-values) for variants from thyroid cancer meta-analysis, the right plot displays -log10(p-values) for variants within the same genomic region from the benign nodular goiter meta-analysis. The lead variant is shown with a purple diamond. Genome wide significance is indicated by the dashed horizontal line at -log10(5e-8).

Supplementary Figure 2.3

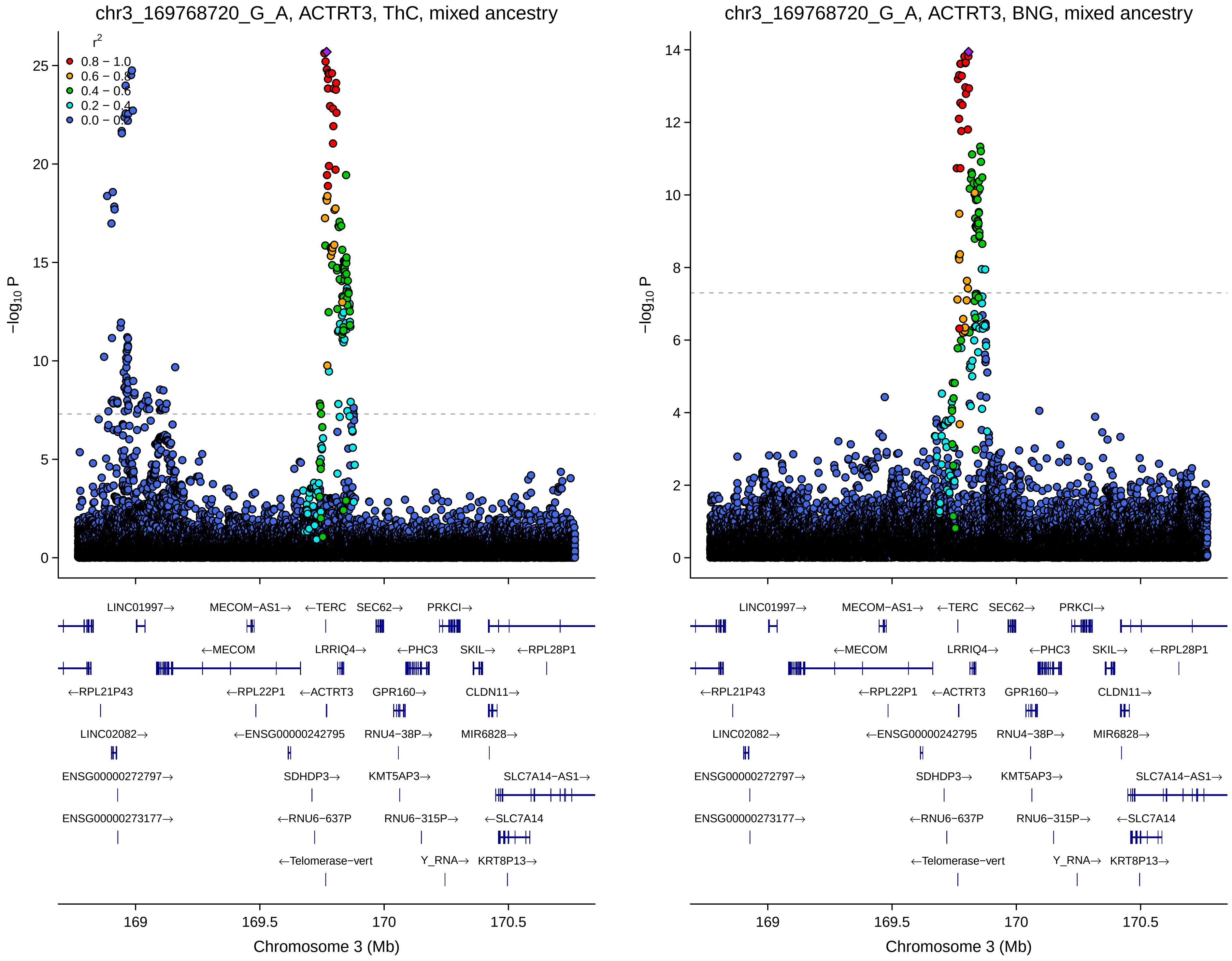

Supplementary Figure 2.3

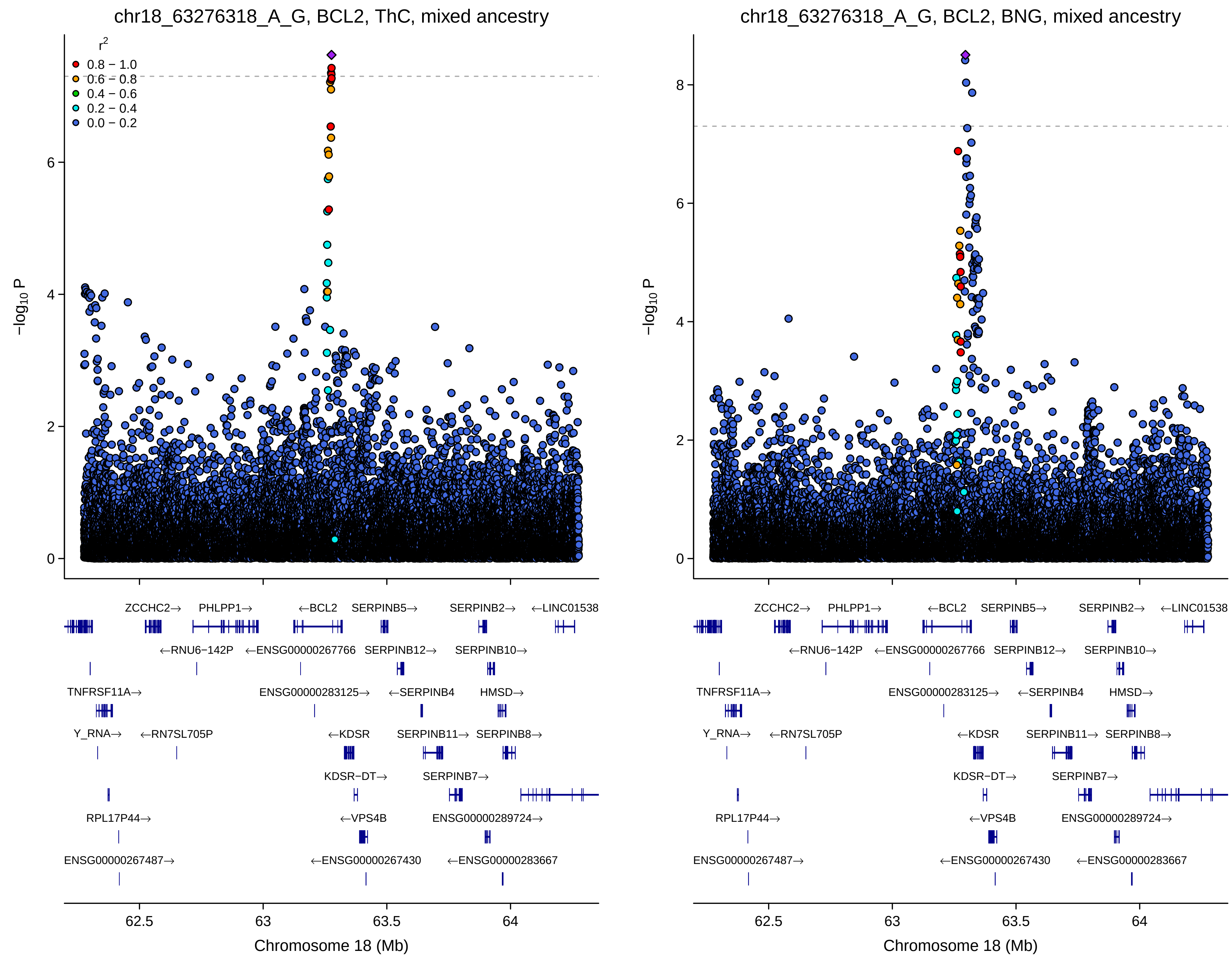

Supplementary Figure 2.3

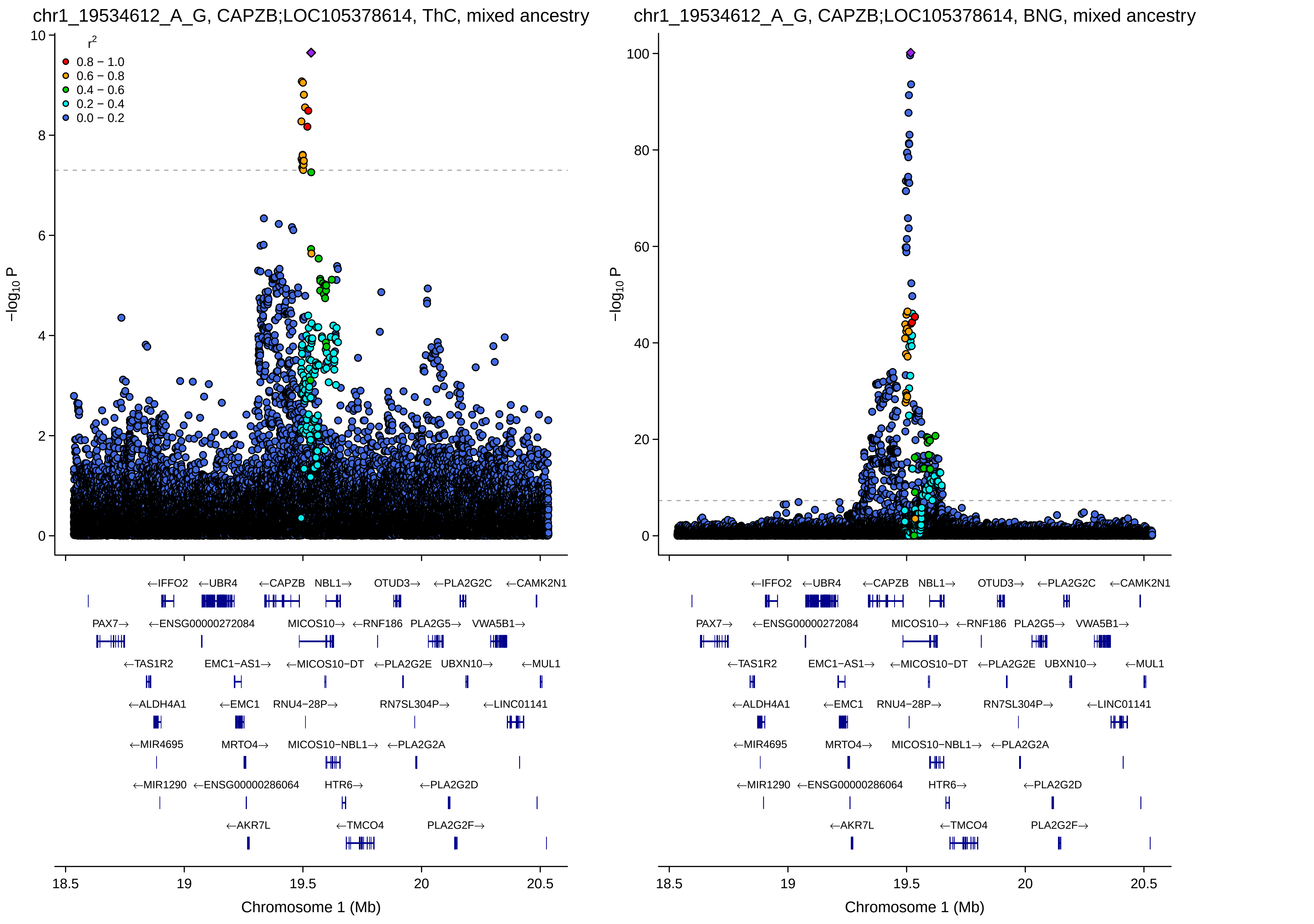

Supplementary Figure 2.3

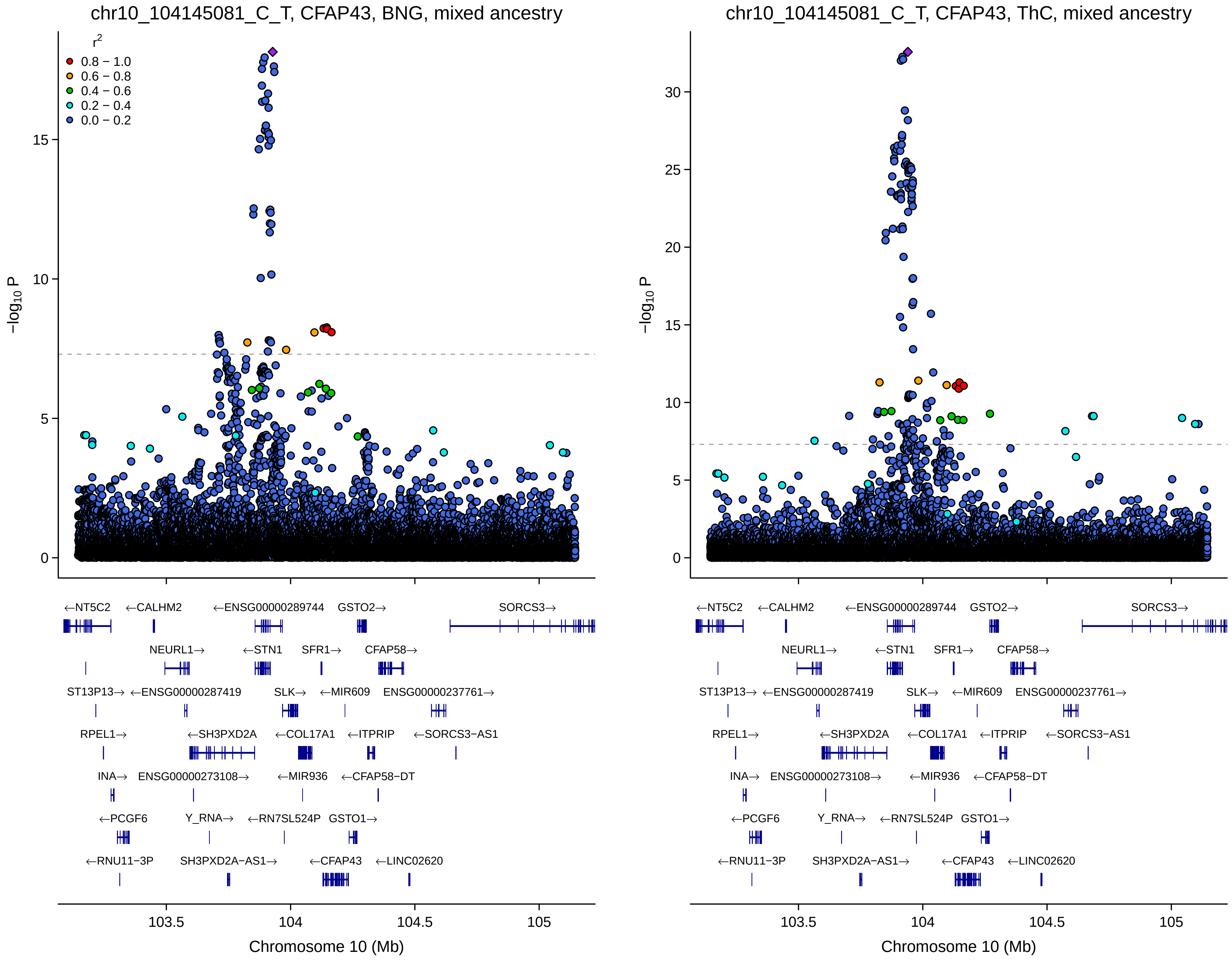

Supplementary Figure 2.3

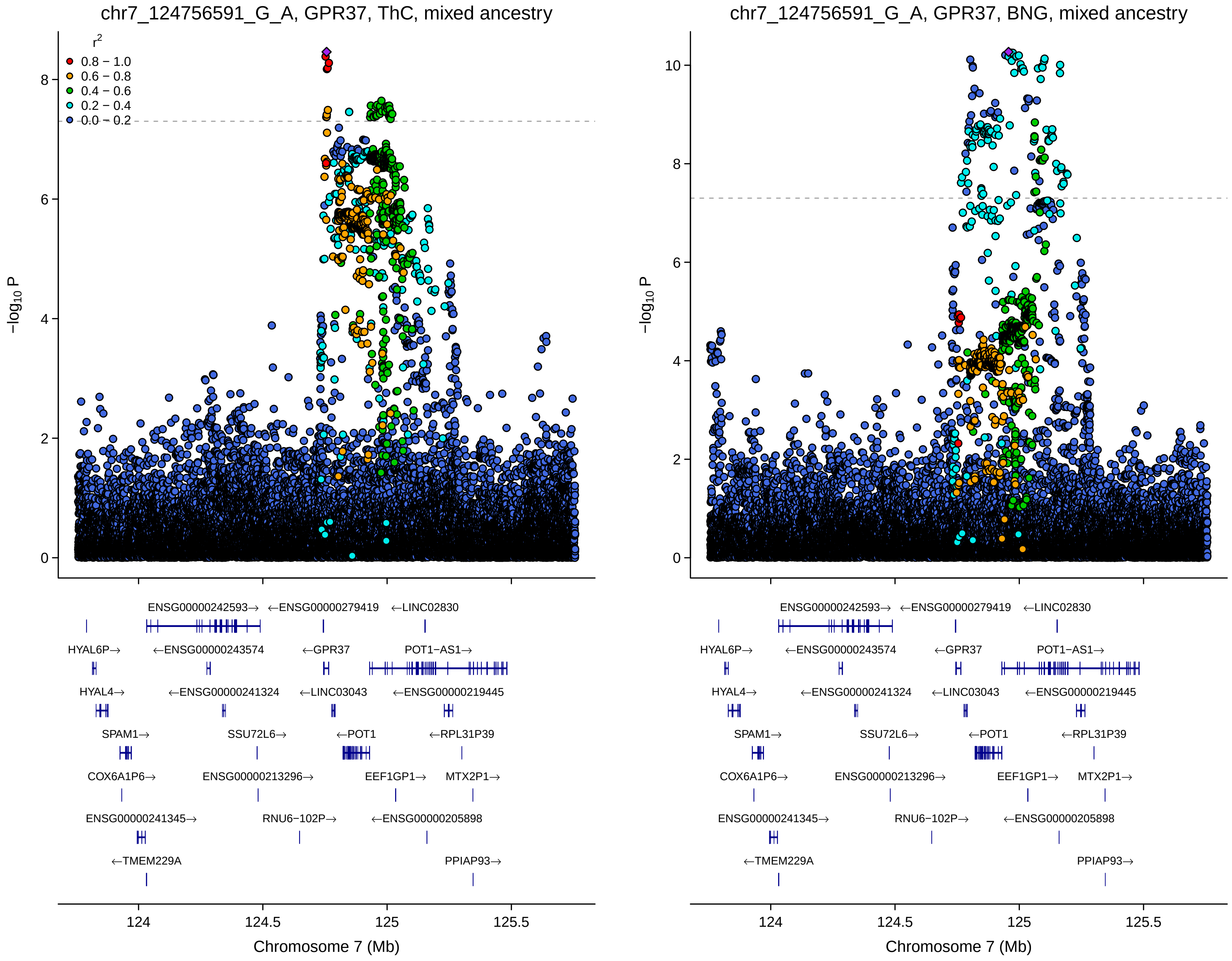

Supplementary Figure 2.3

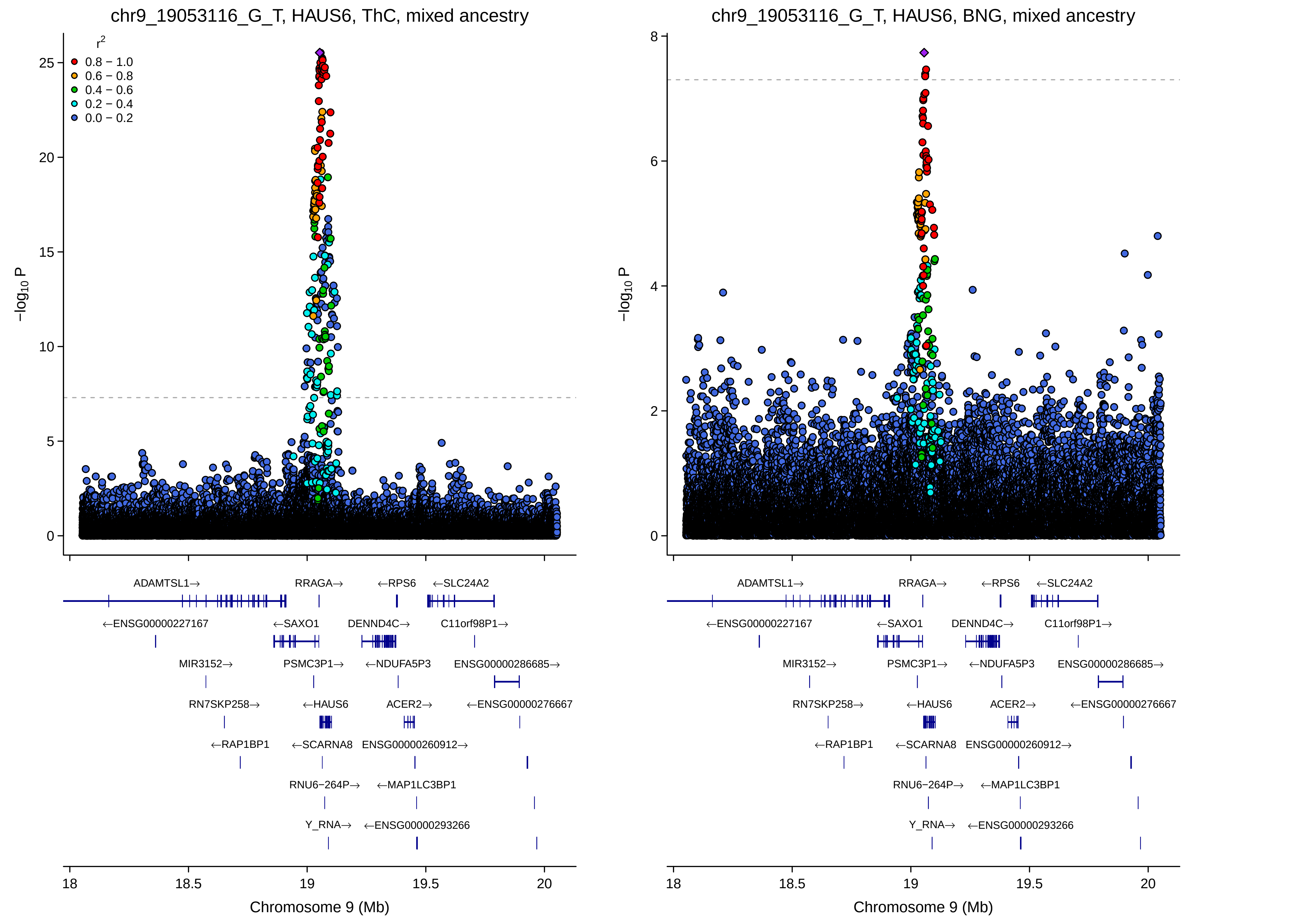

Supplementary Figure 2.3

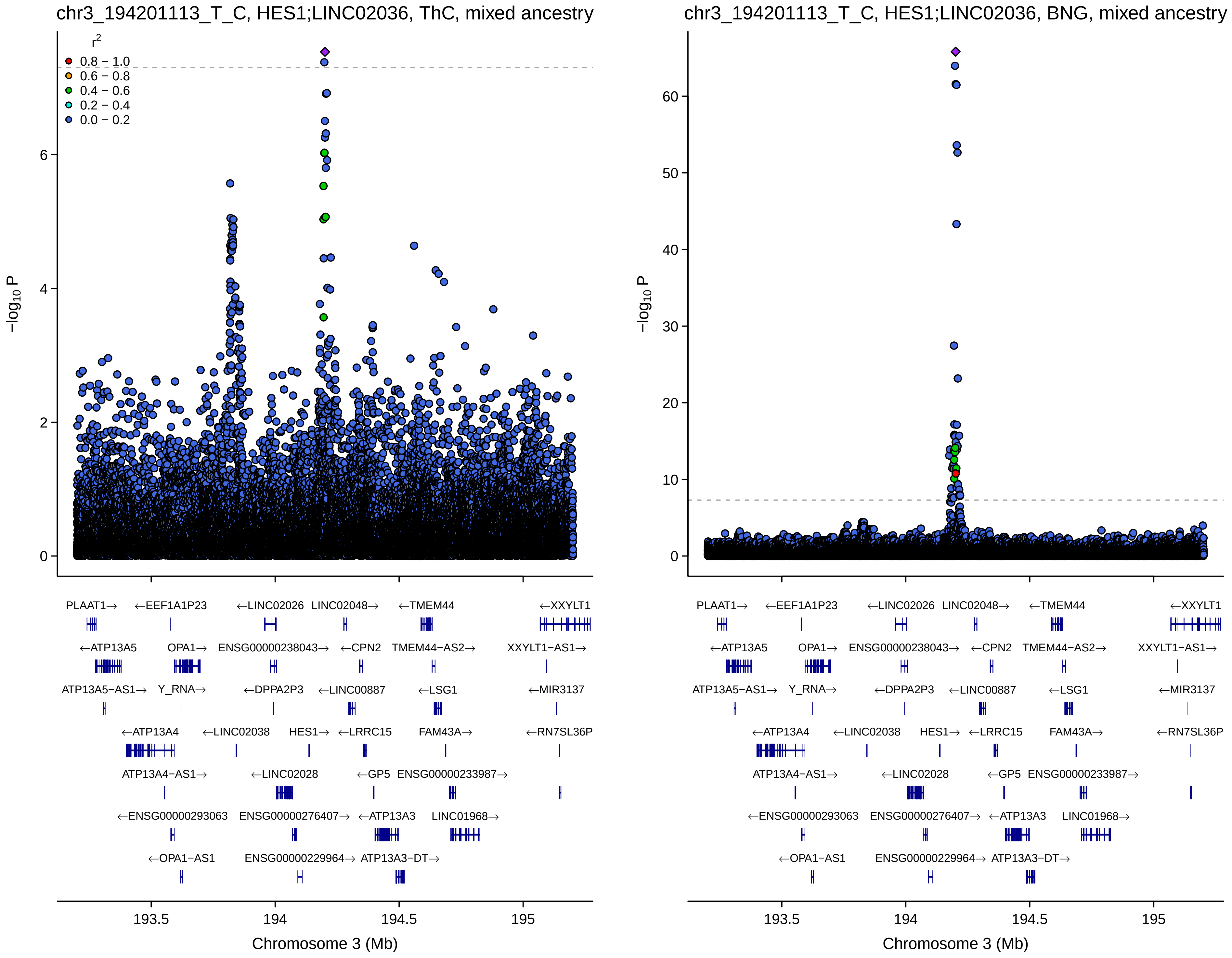

Supplementary Figure 2.3

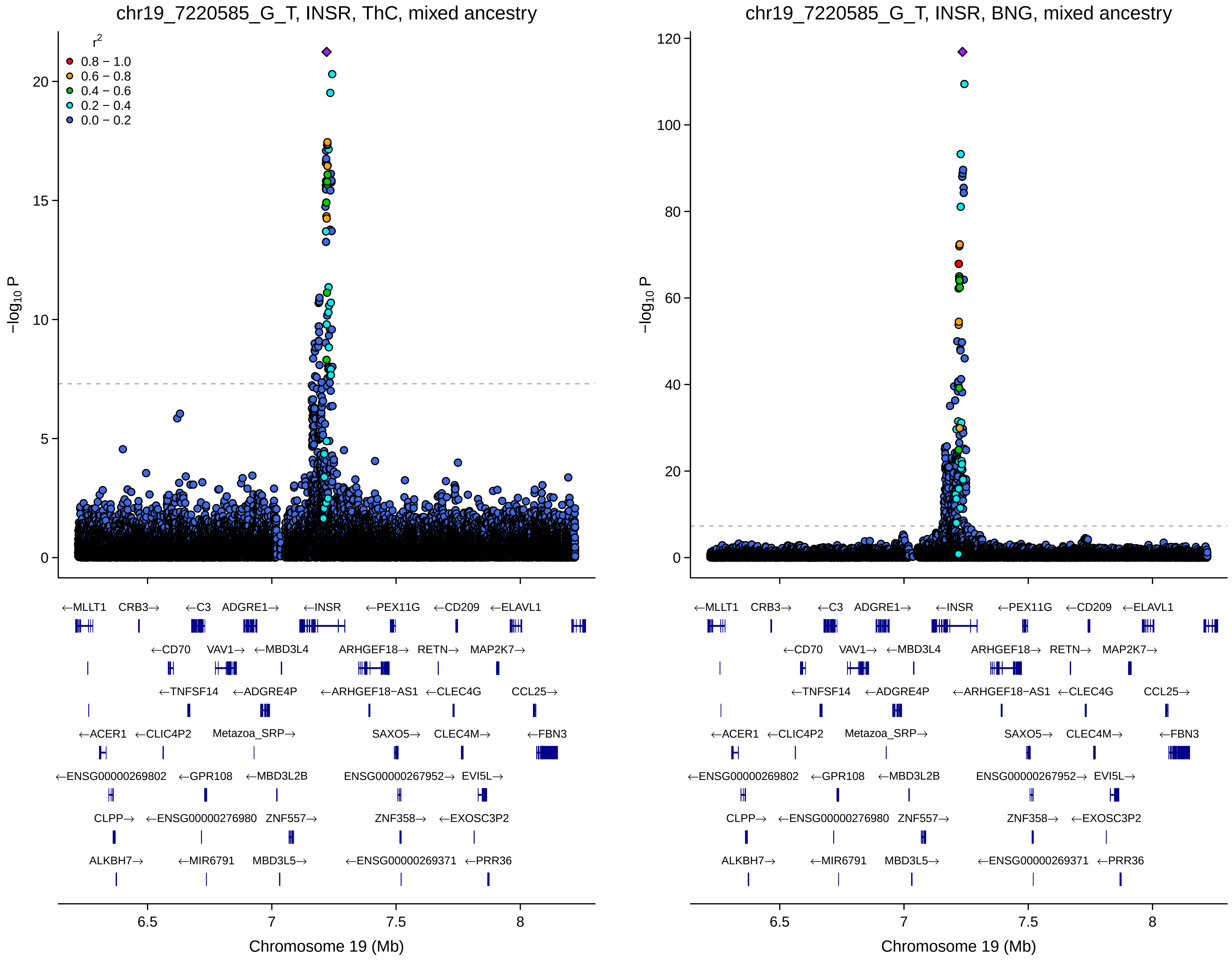

Supplementary Figure 2.3

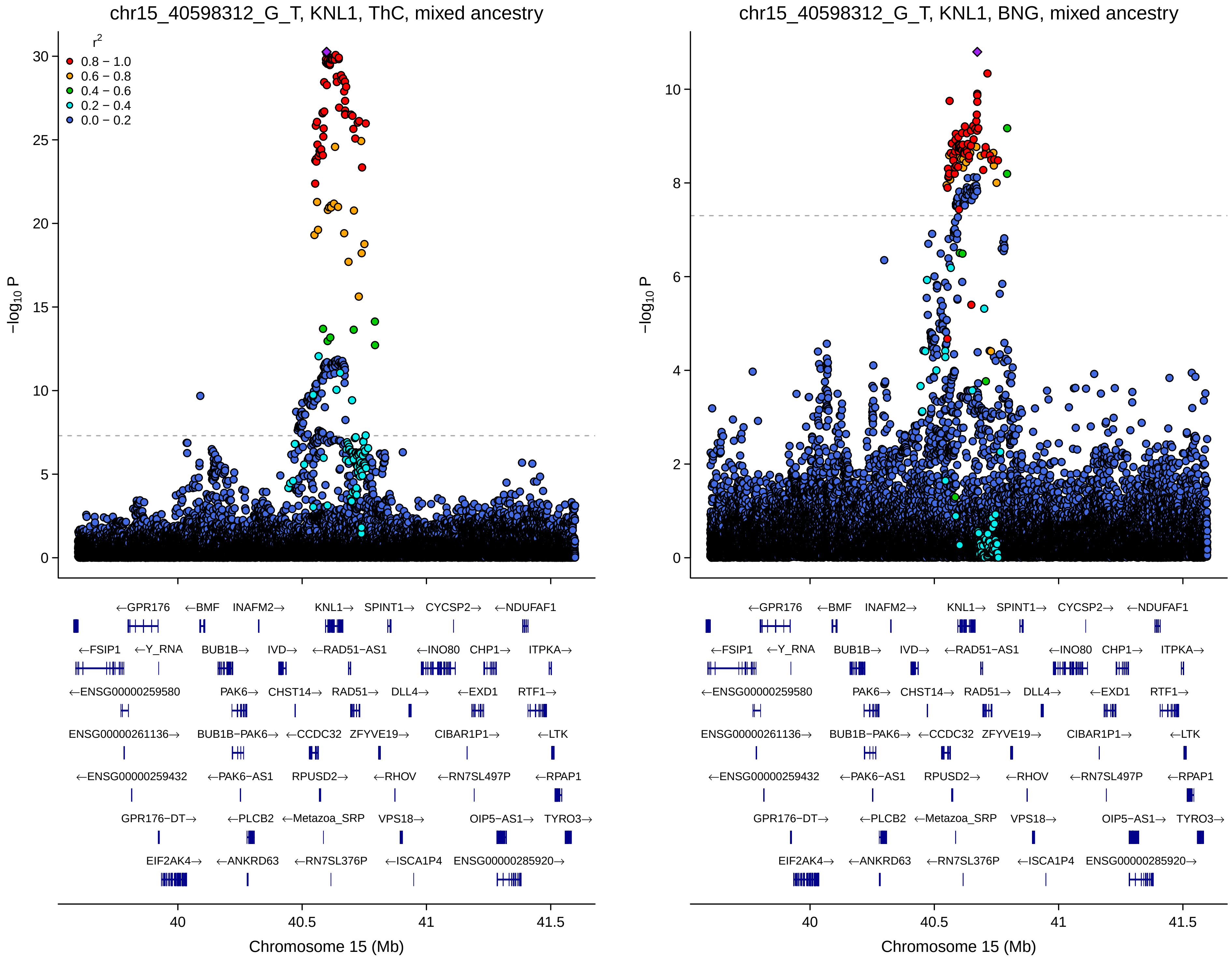

Supplementary Figure 2.3

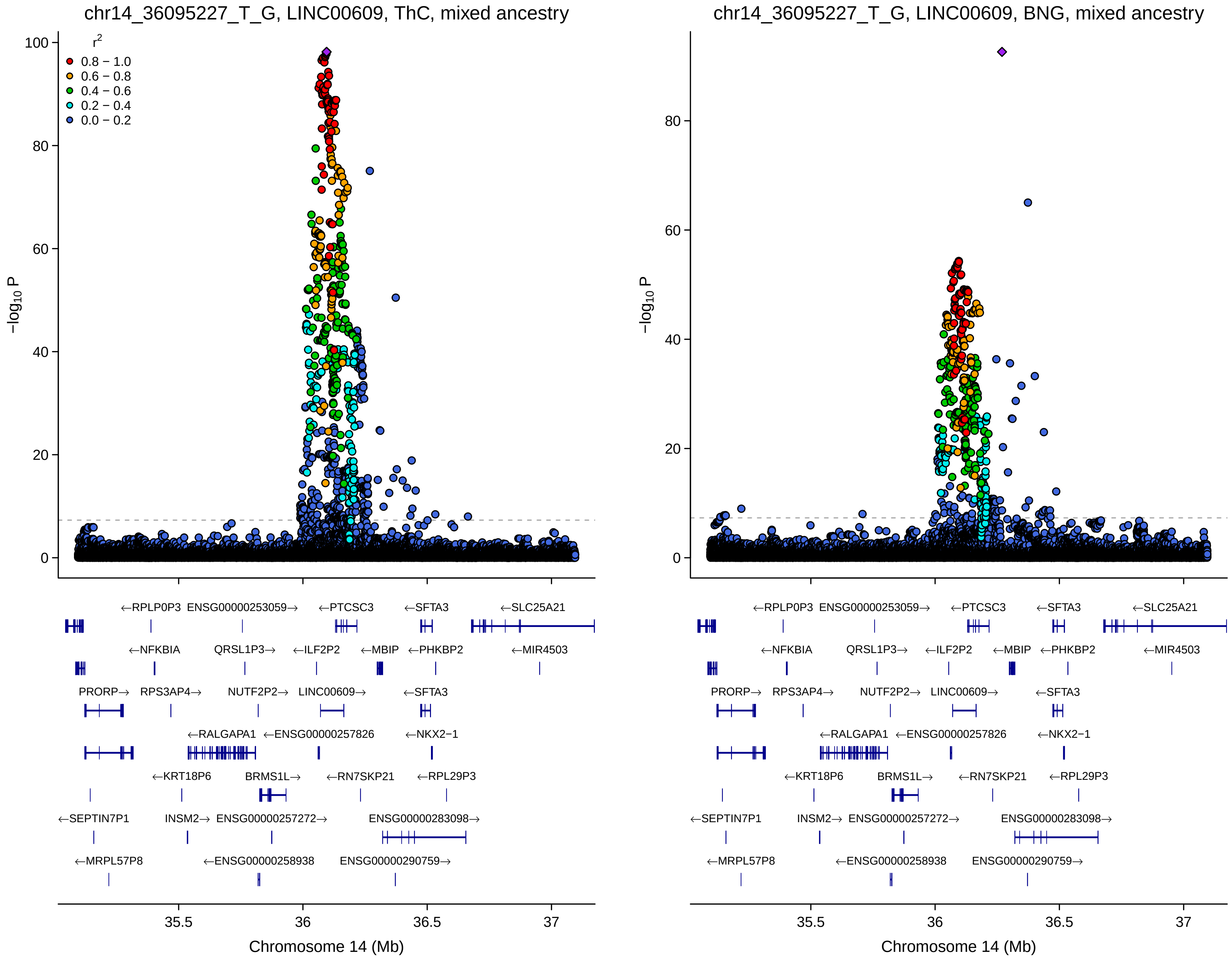

Supplementary Figure 2.3

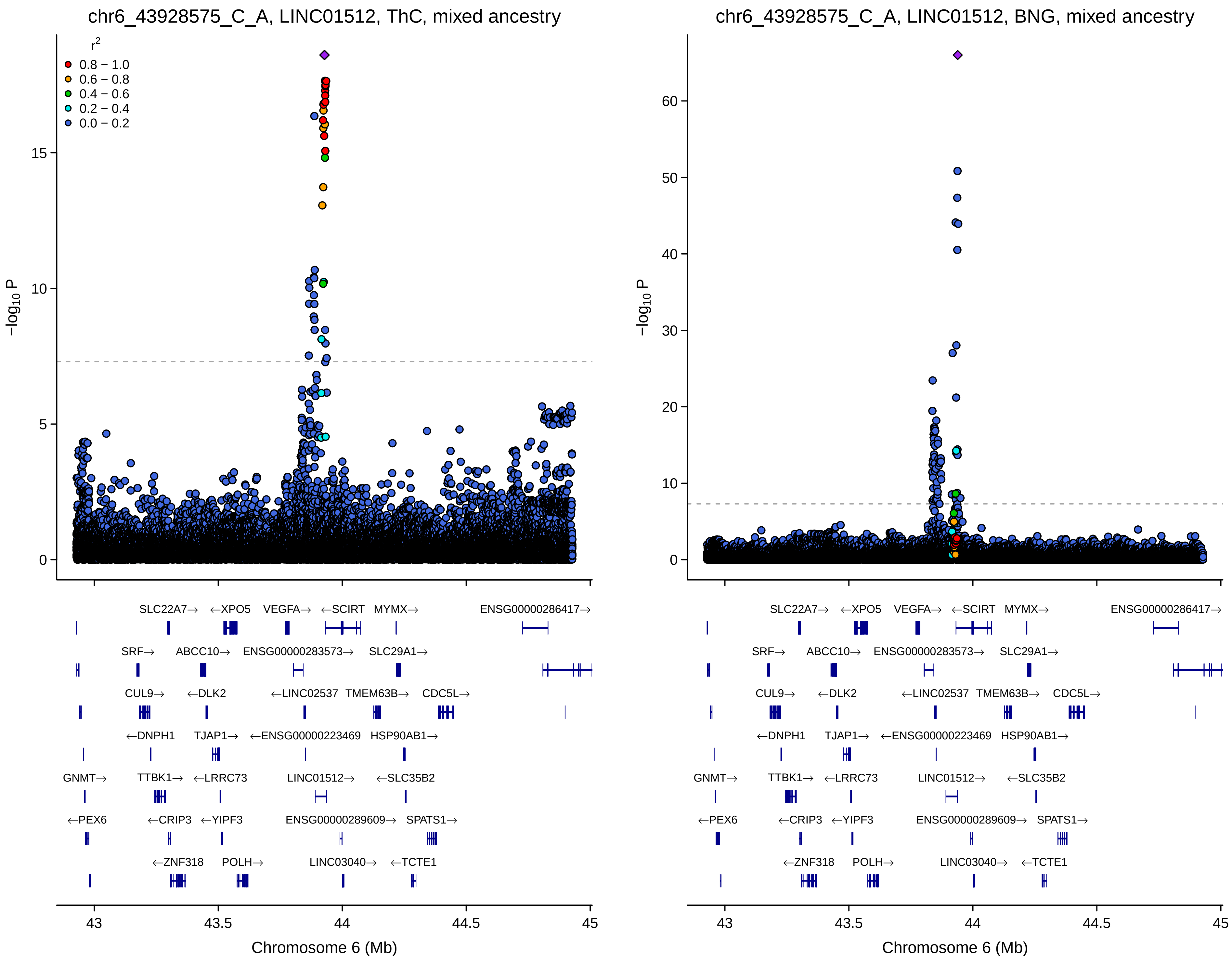

Supplementary Figure 2.3

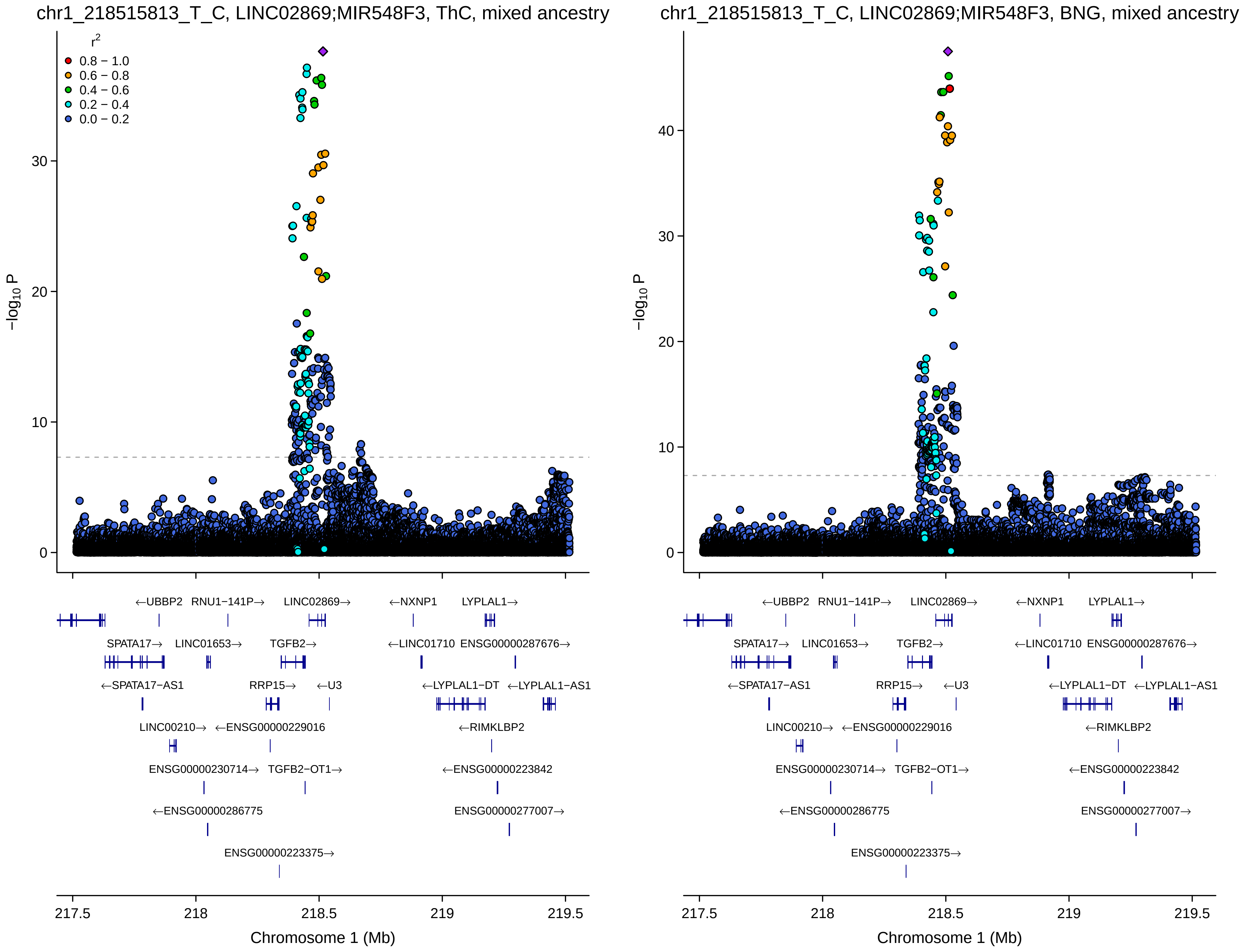

Supplementary Figure 2.3

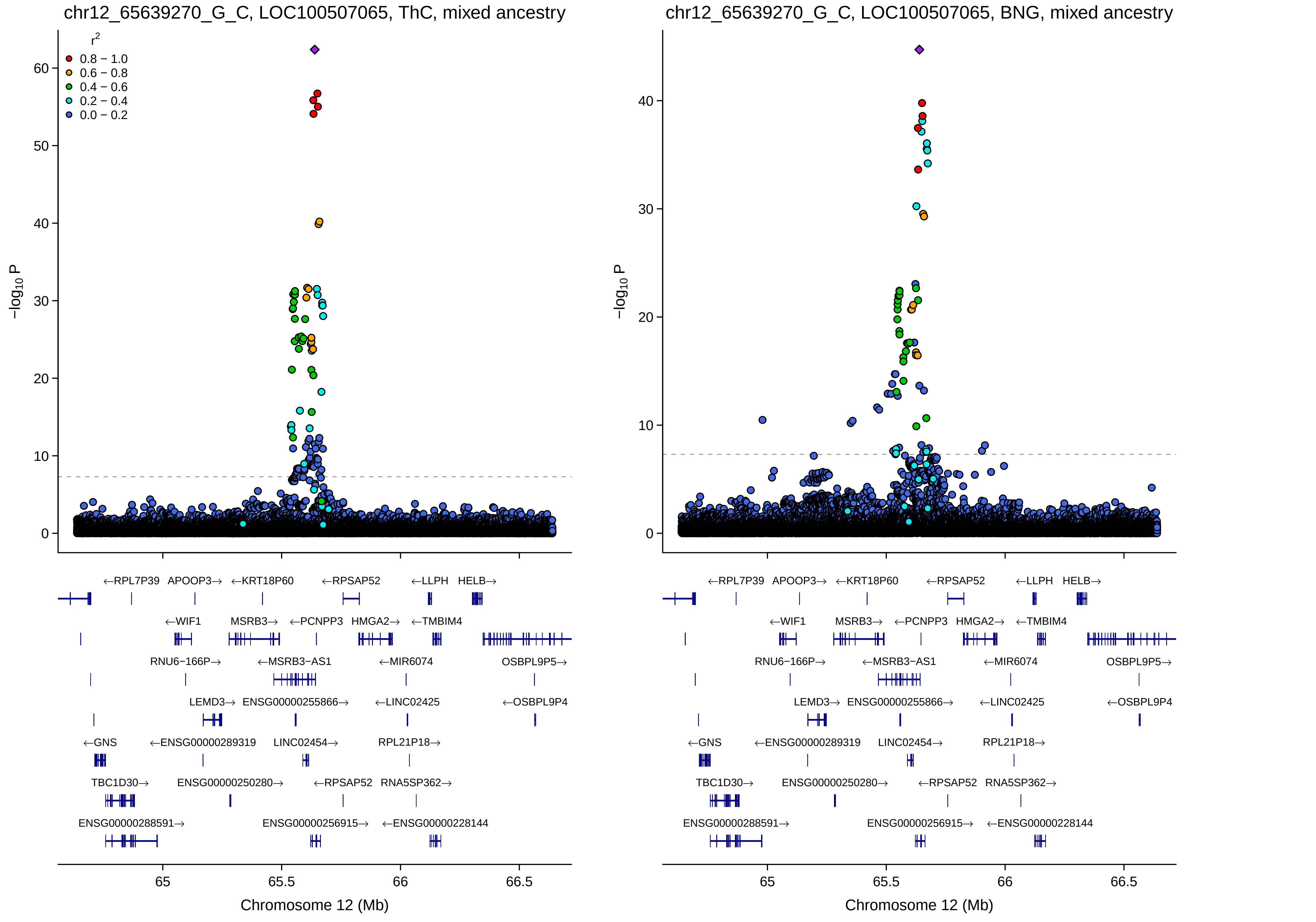

Supplementary Figure 2.3

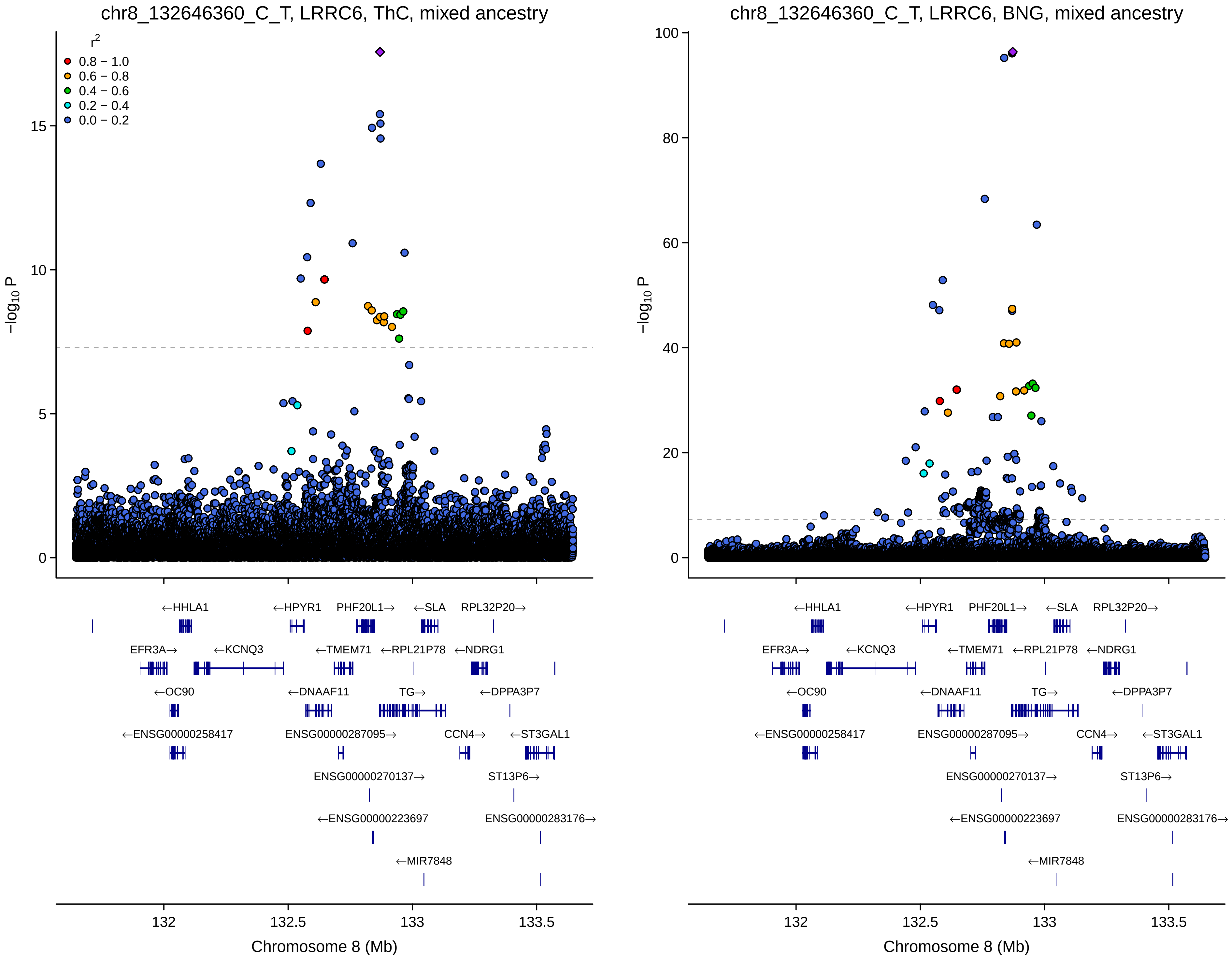

Supplementary Figure 2.3

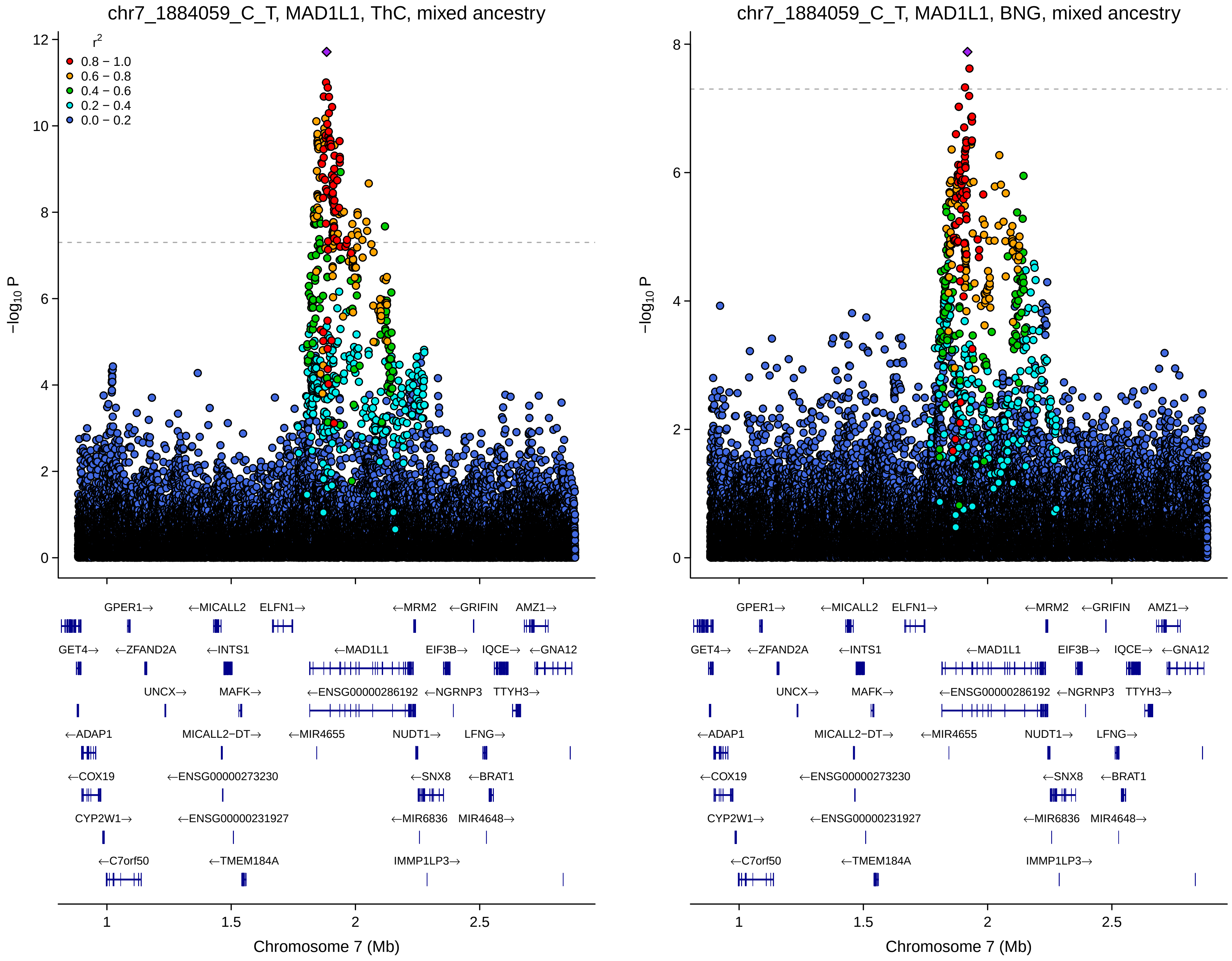

Supplementary Figure 2.3

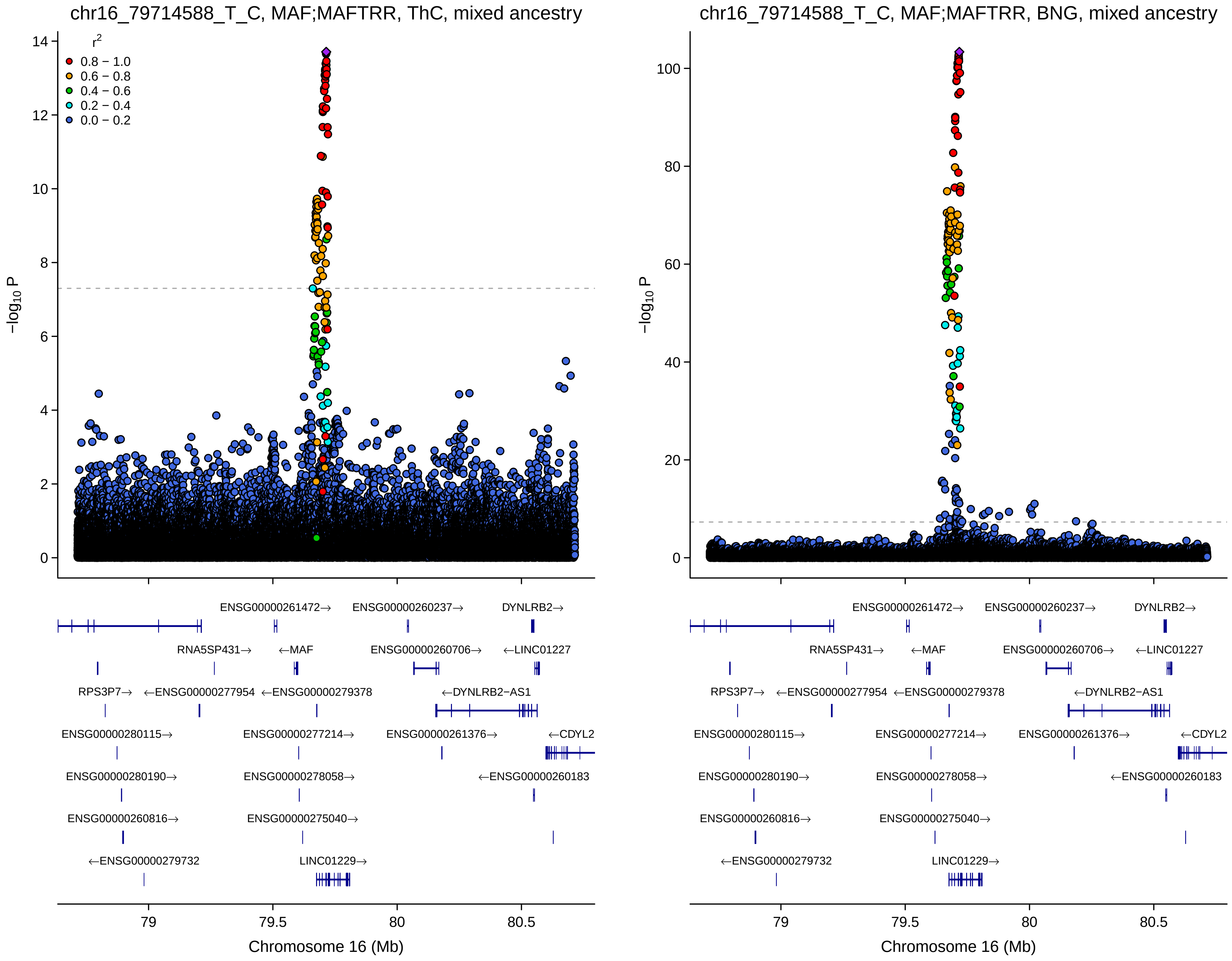

Supplementary Figure 2.3

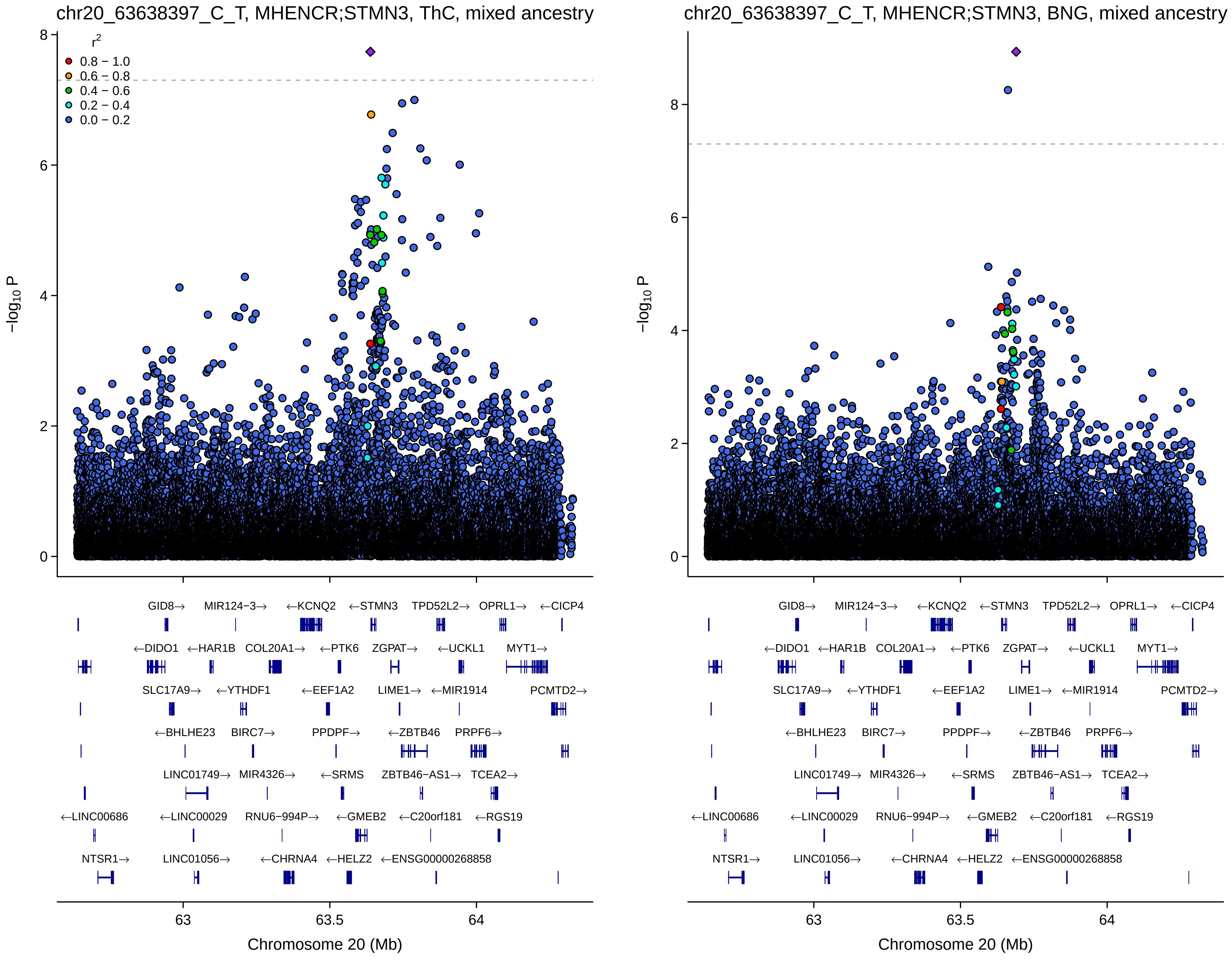

Supplementary Figure 2.3

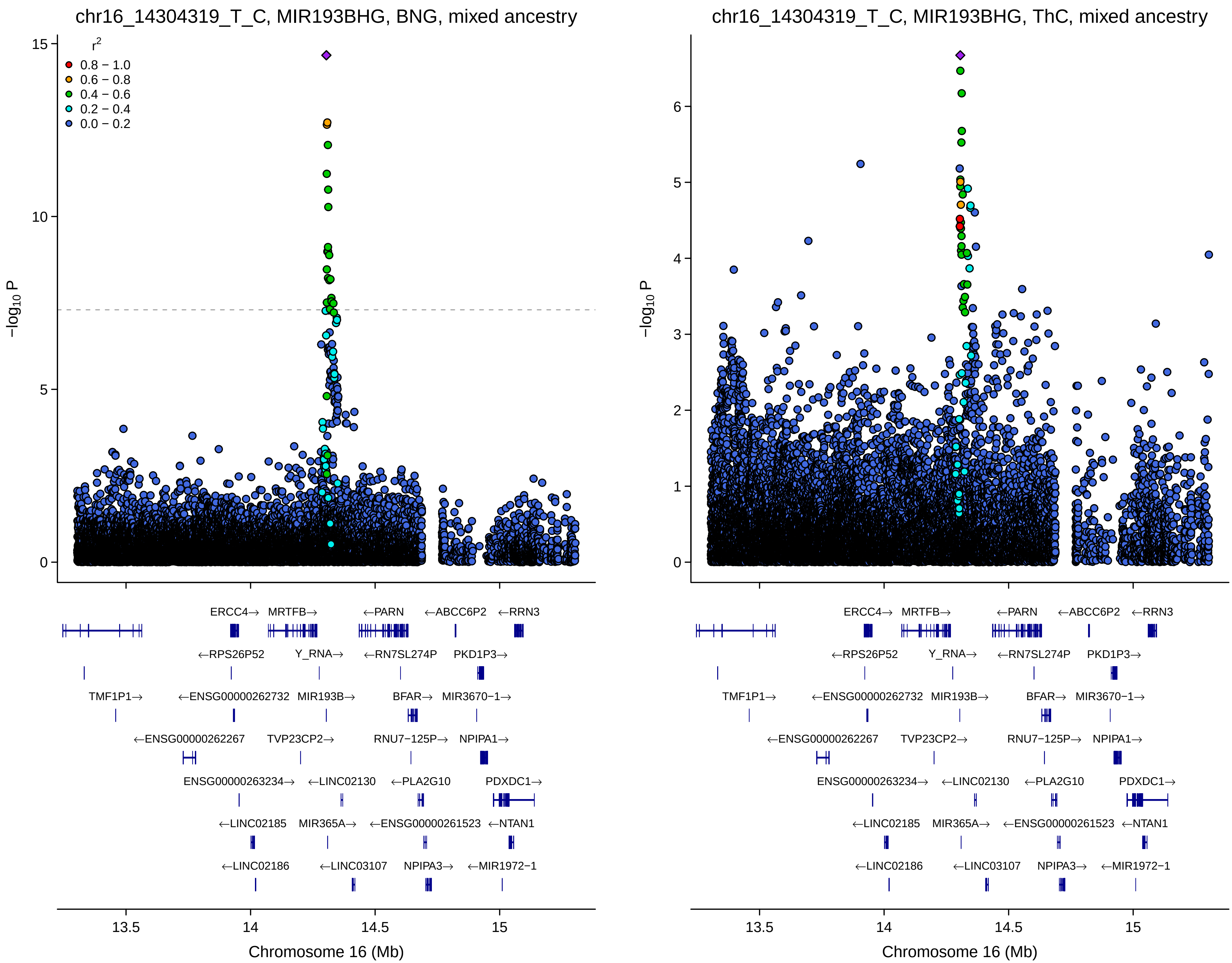

Supplementary Figure 2.3

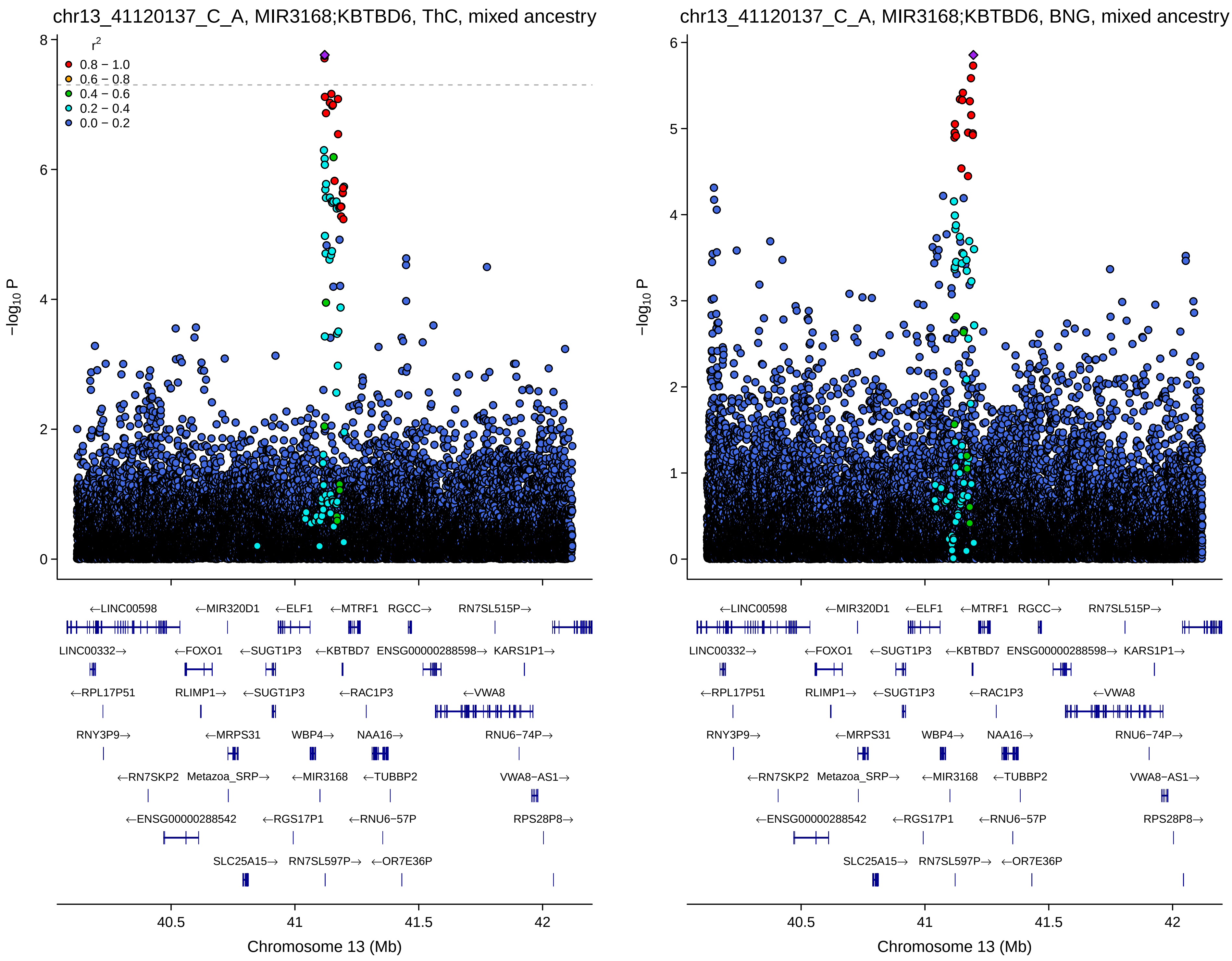

Supplementary Figure 2.3

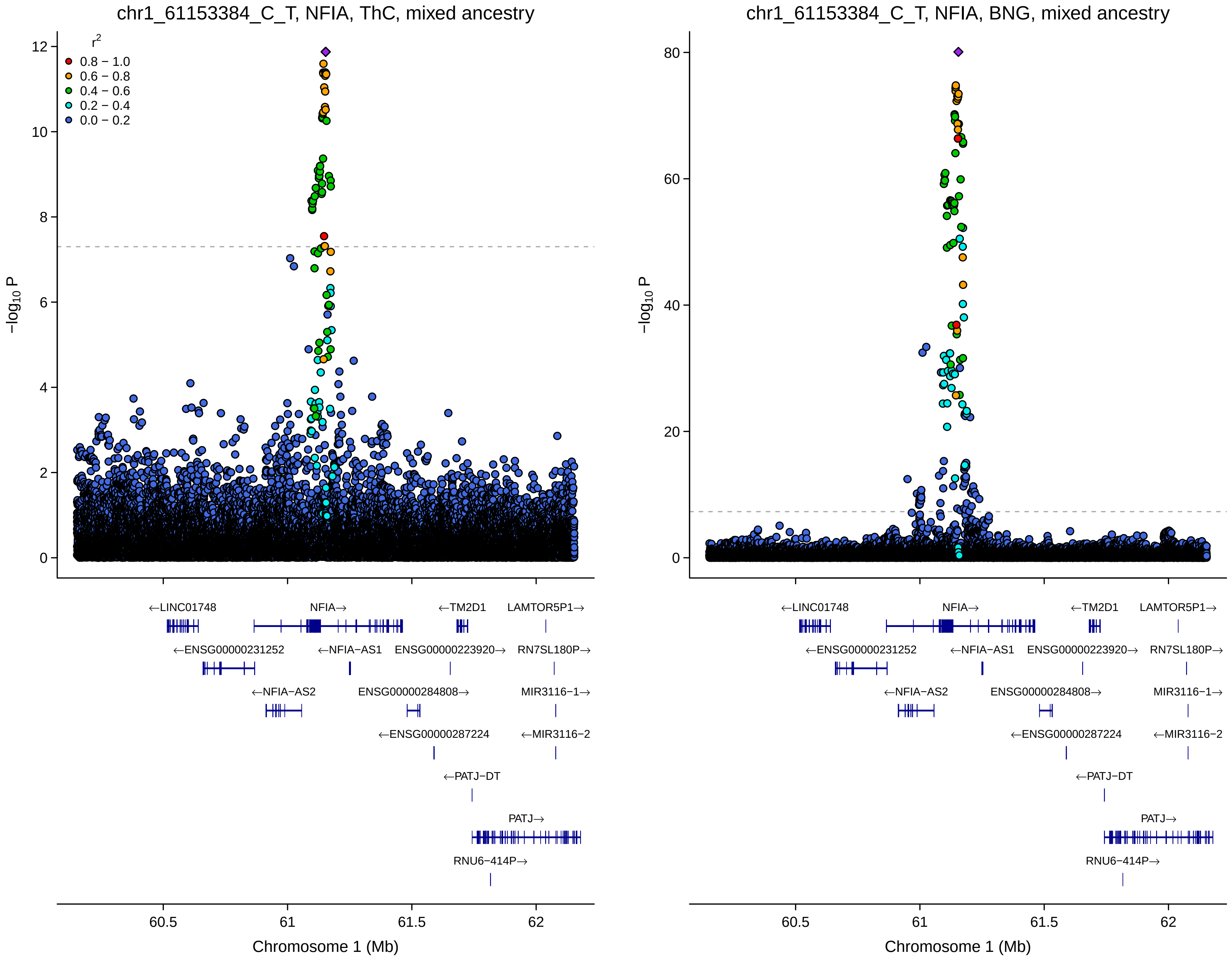

Supplementary Figure 2.3

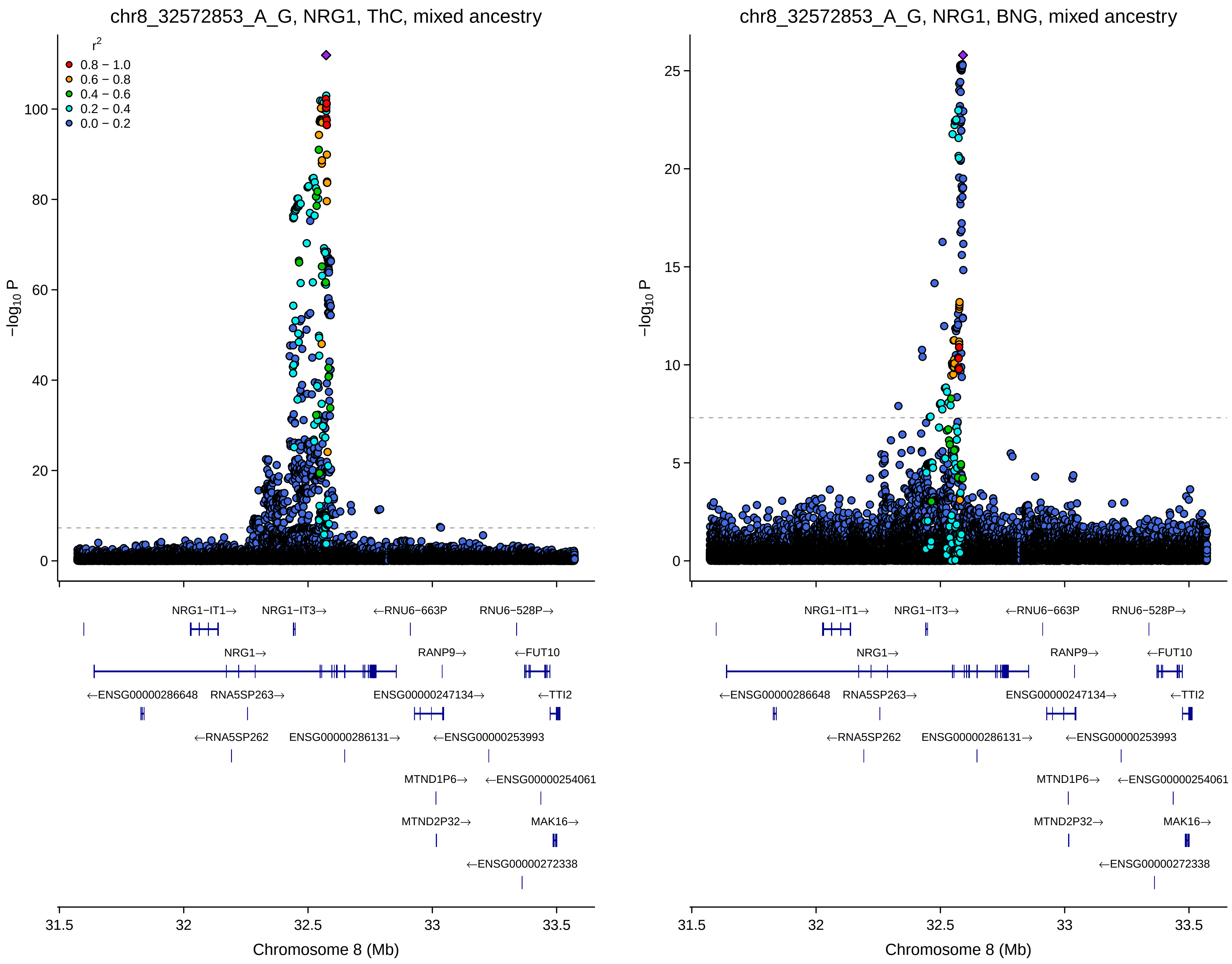

Supplementary Figure 2.3

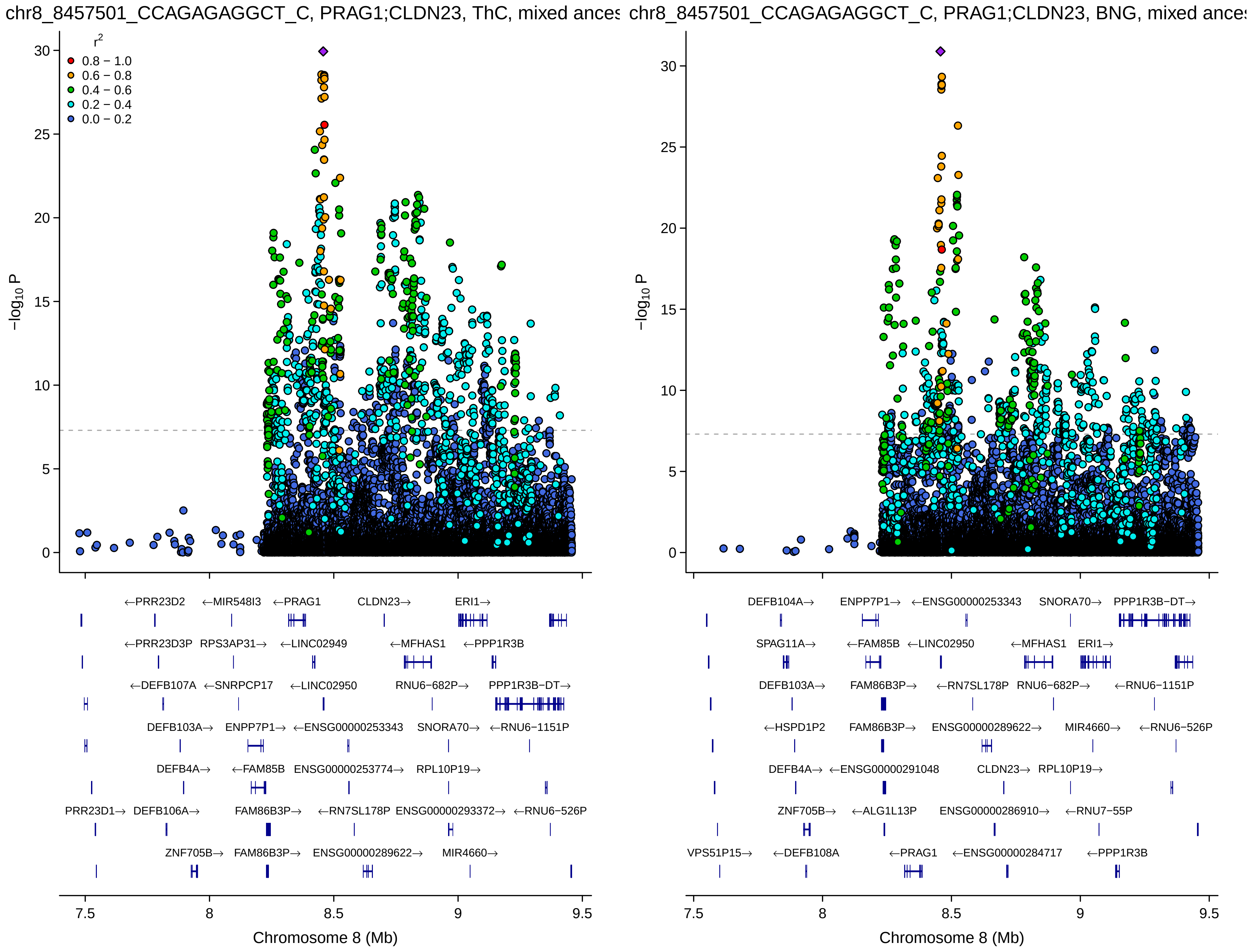

Supplementary Figure 2.3

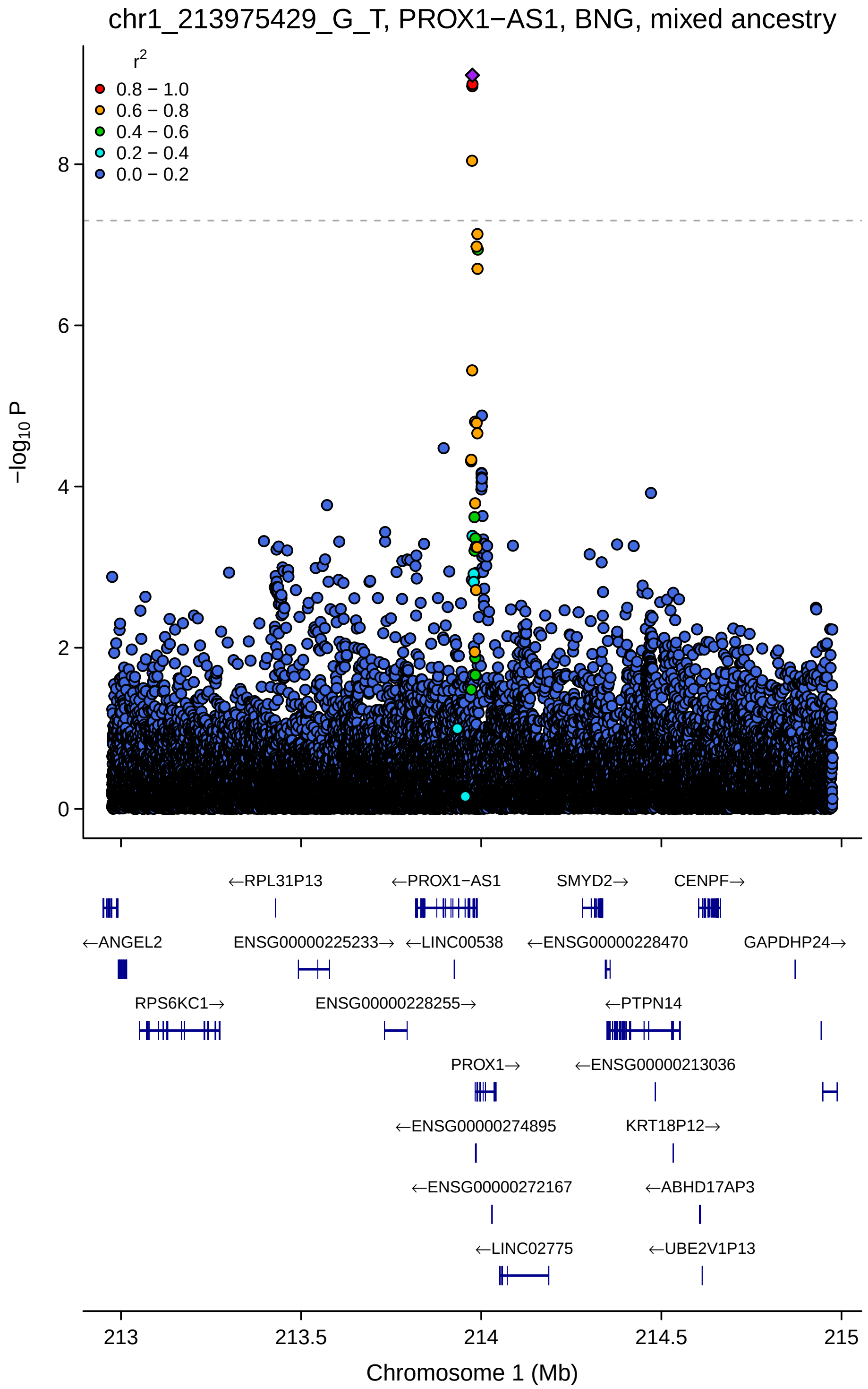

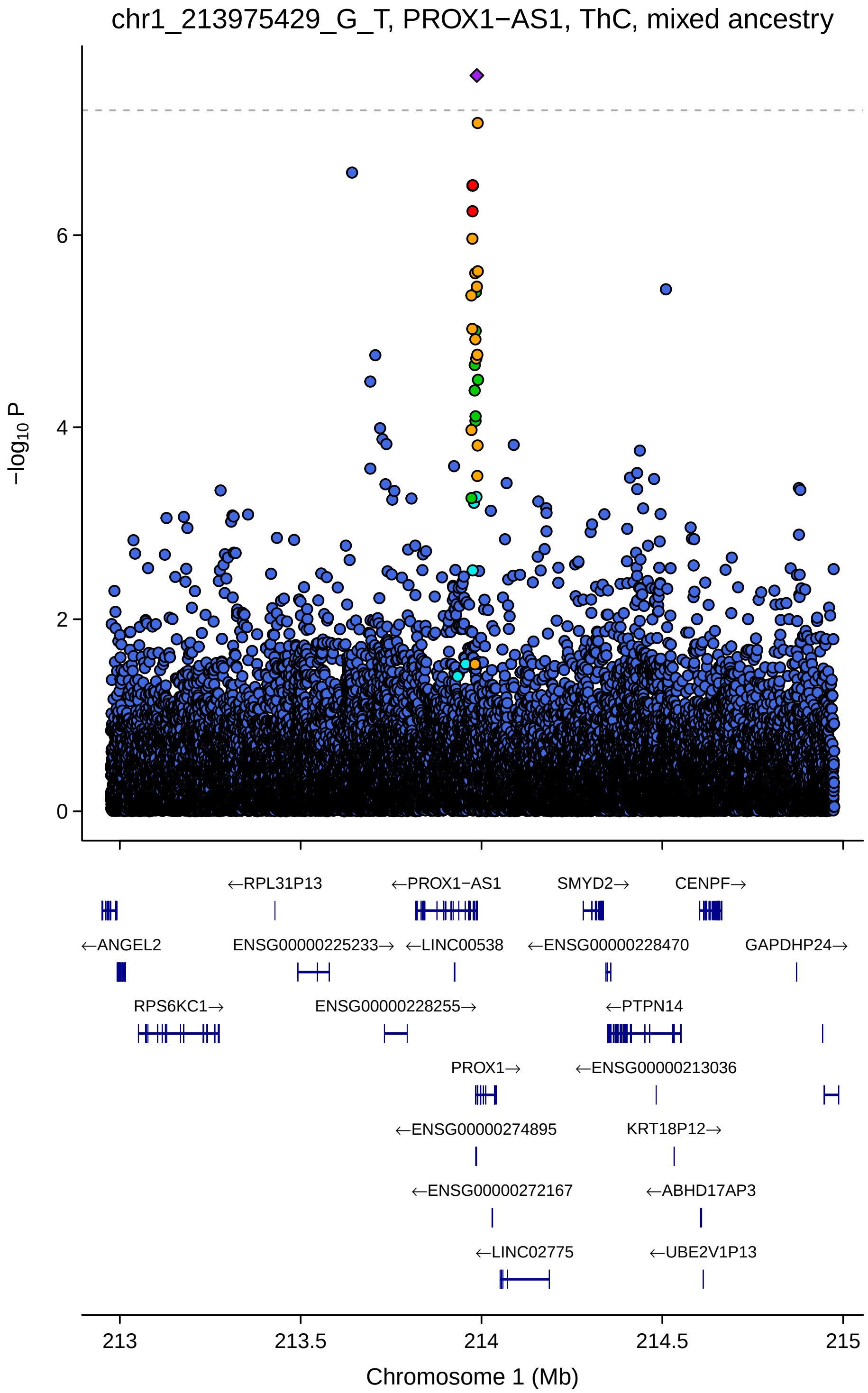

Supplementary Figure 2.3

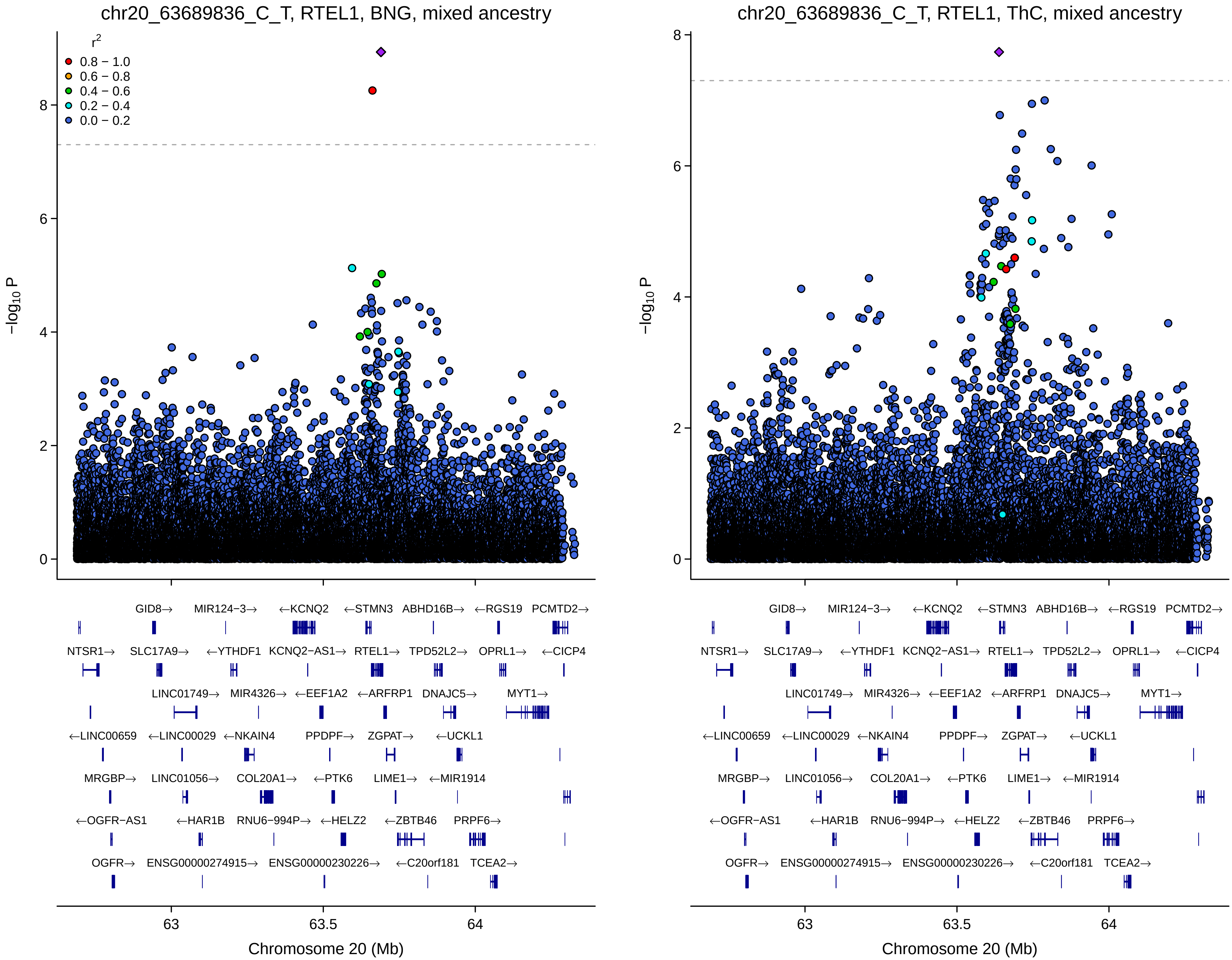

Supplementary Figure 2.3

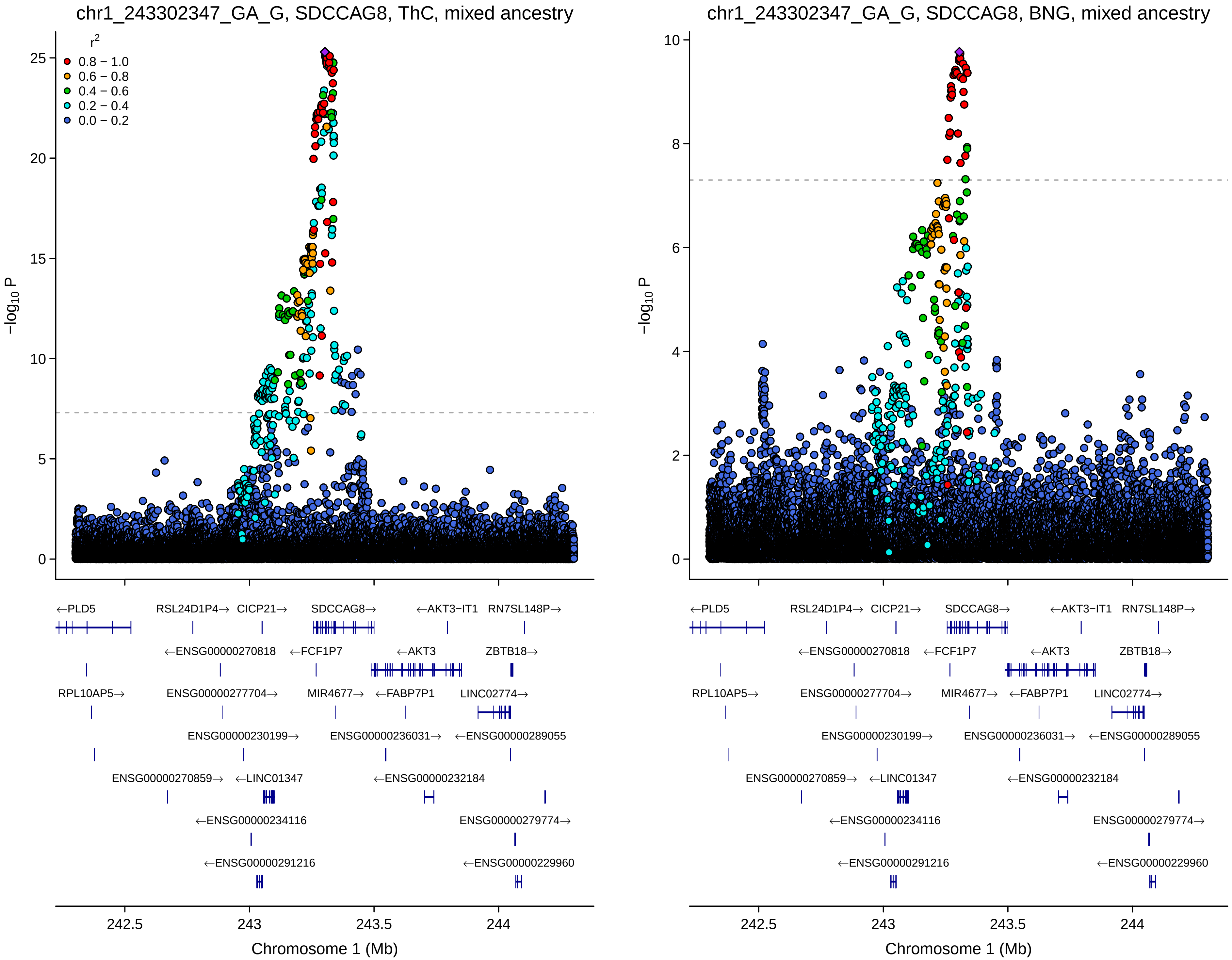

Supplementary Figure 2.3

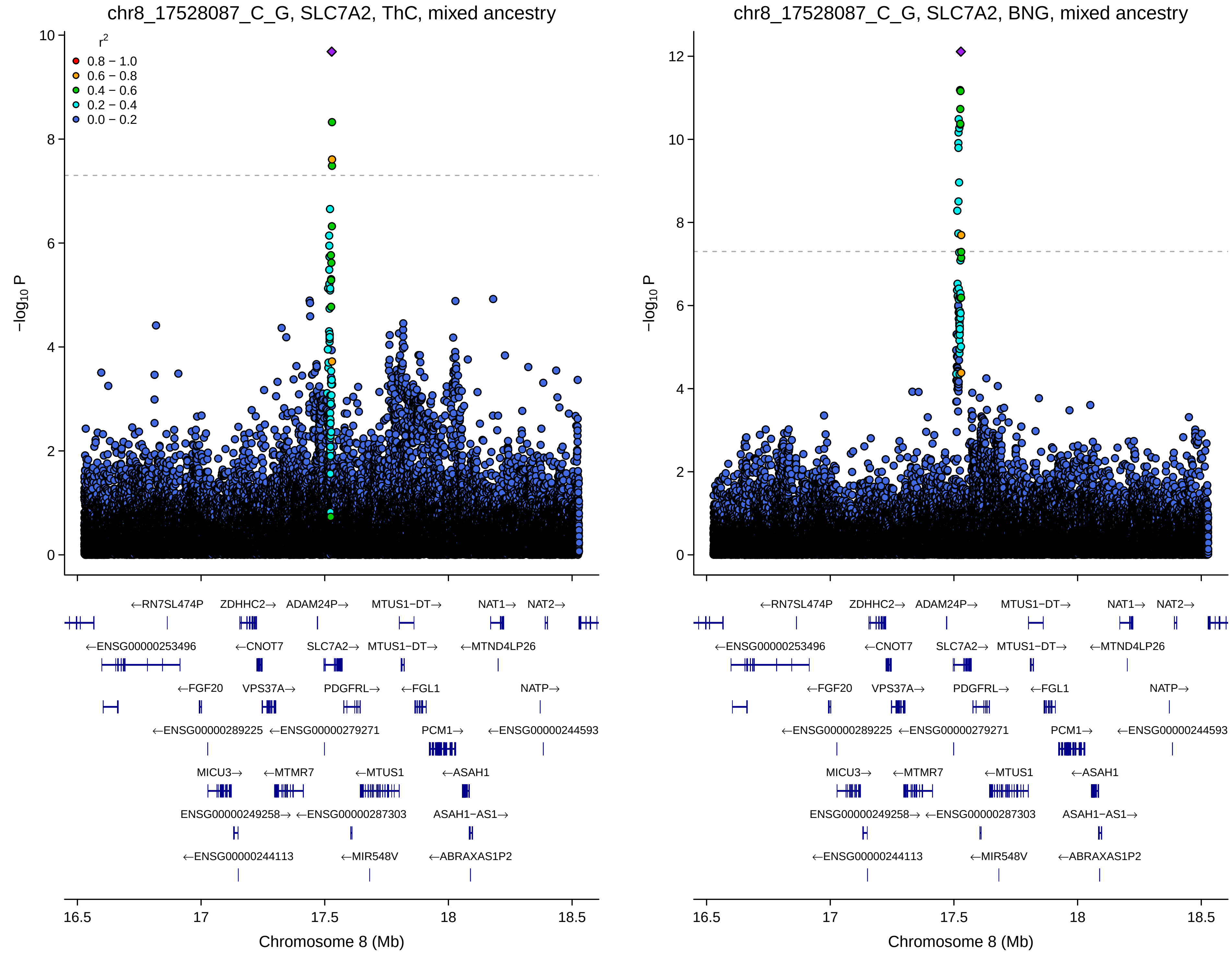

Supplementary Figure 2.3

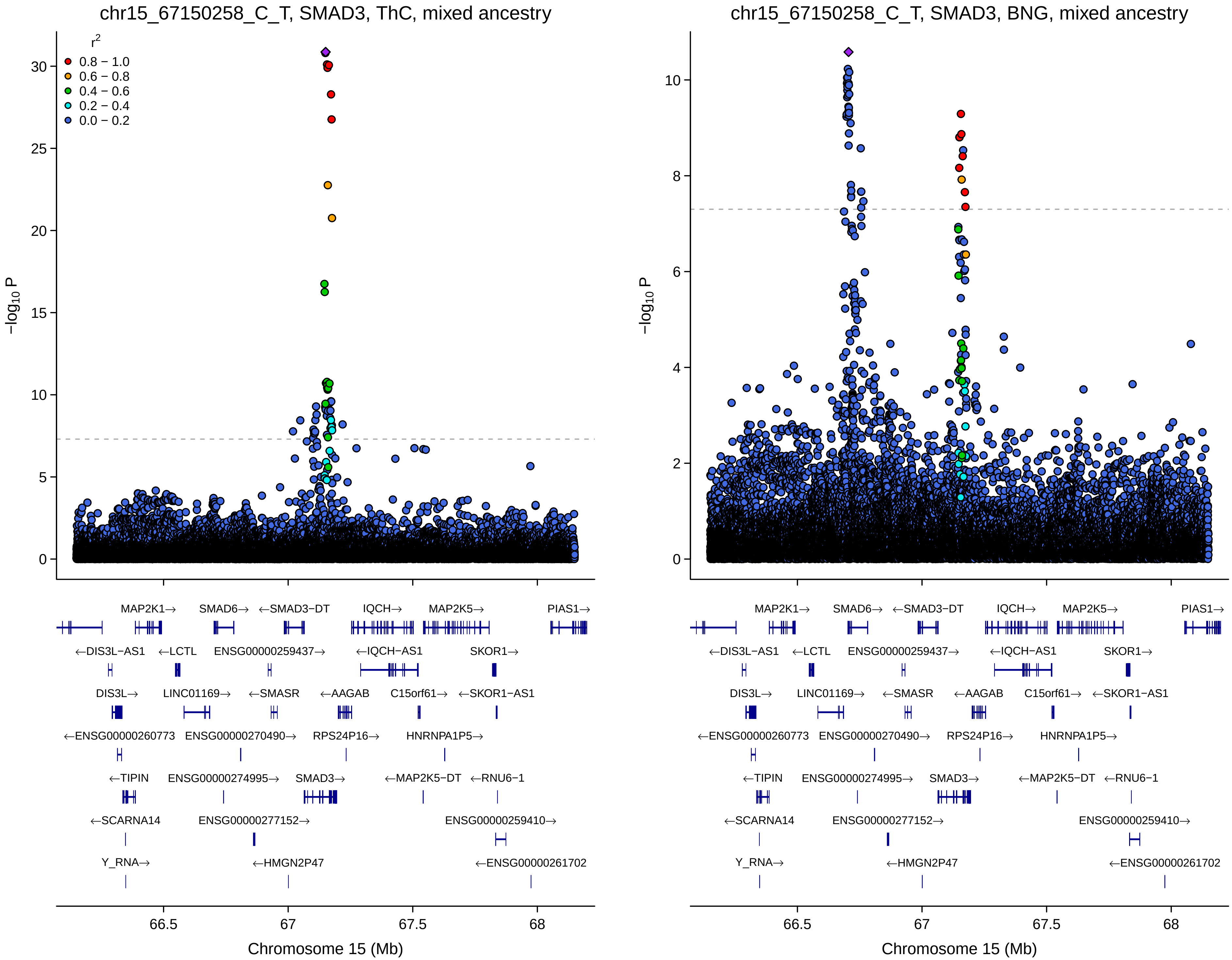

Supplementary Figure 2.3

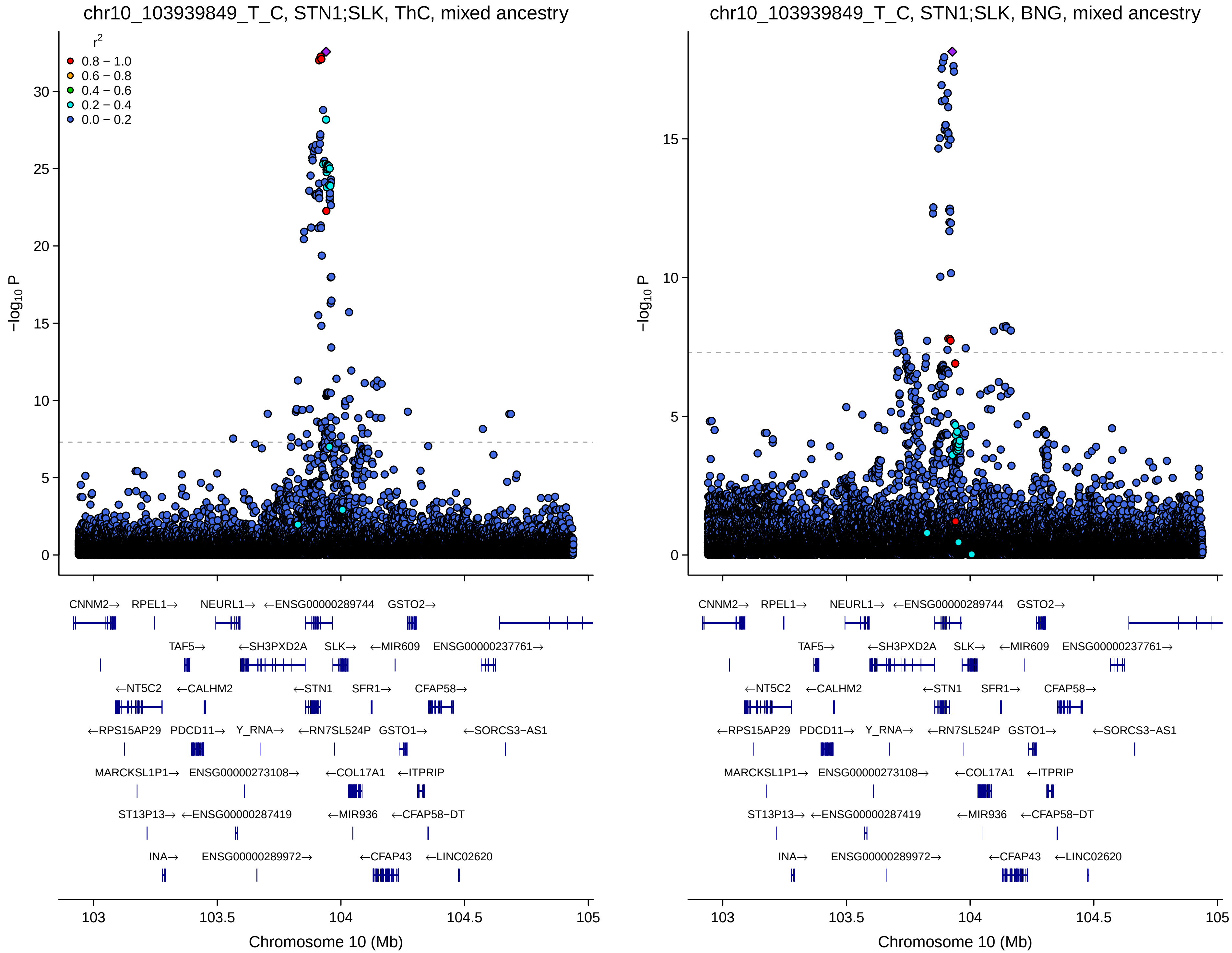

Supplementary Figure 2.3

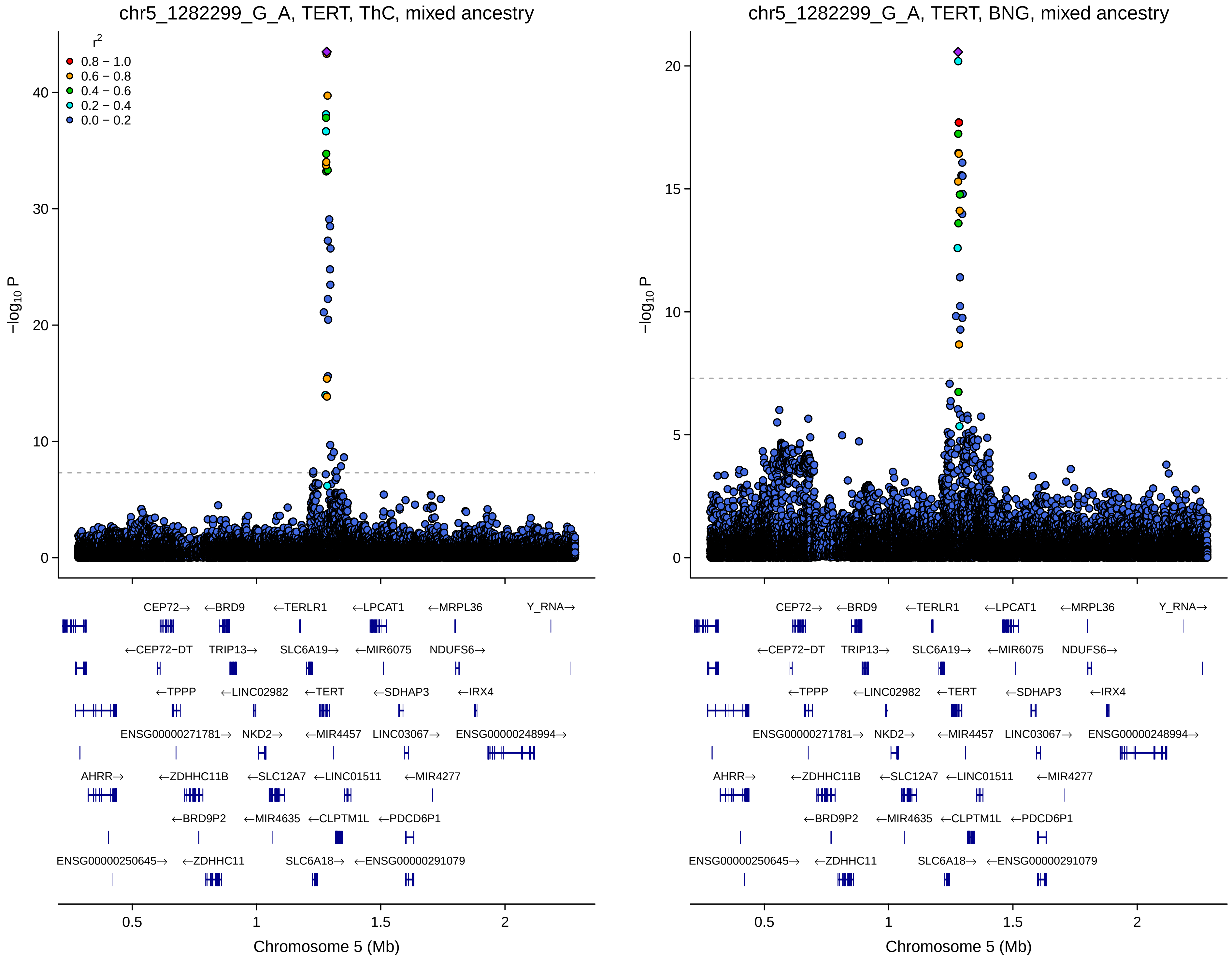

Supplementary Figure 2.3

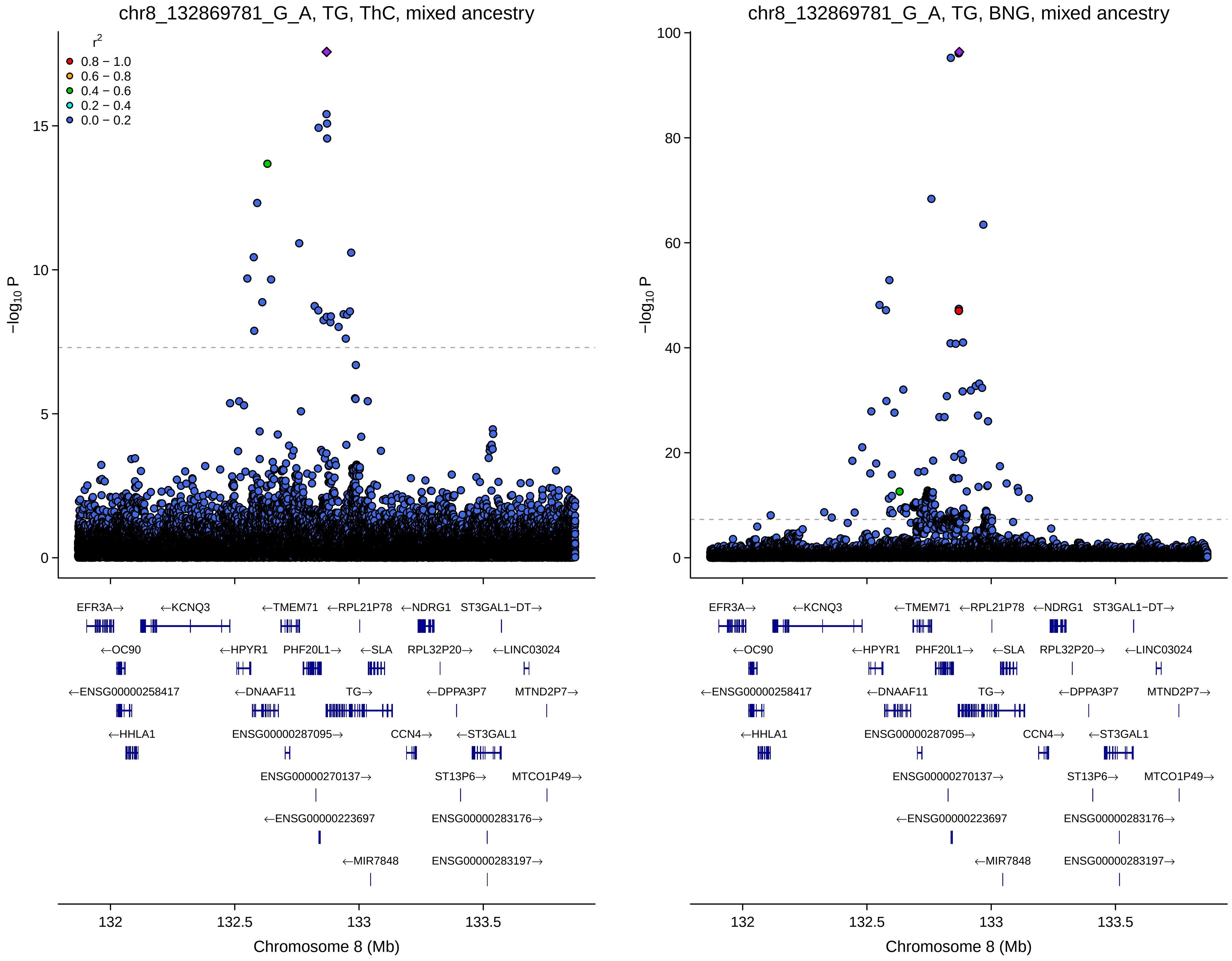

Supplementary Figure 2.3

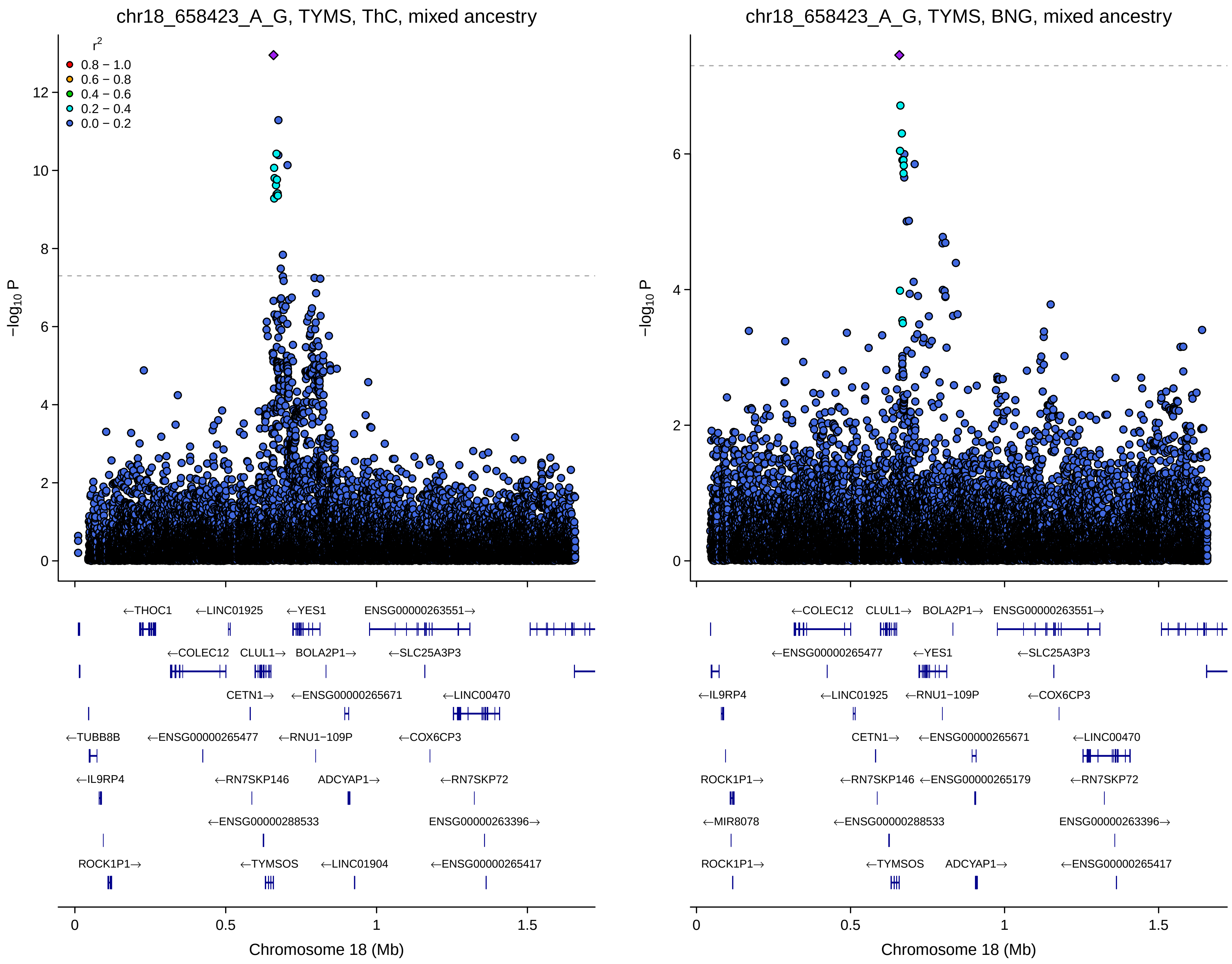

Supplementary Figure 2.3

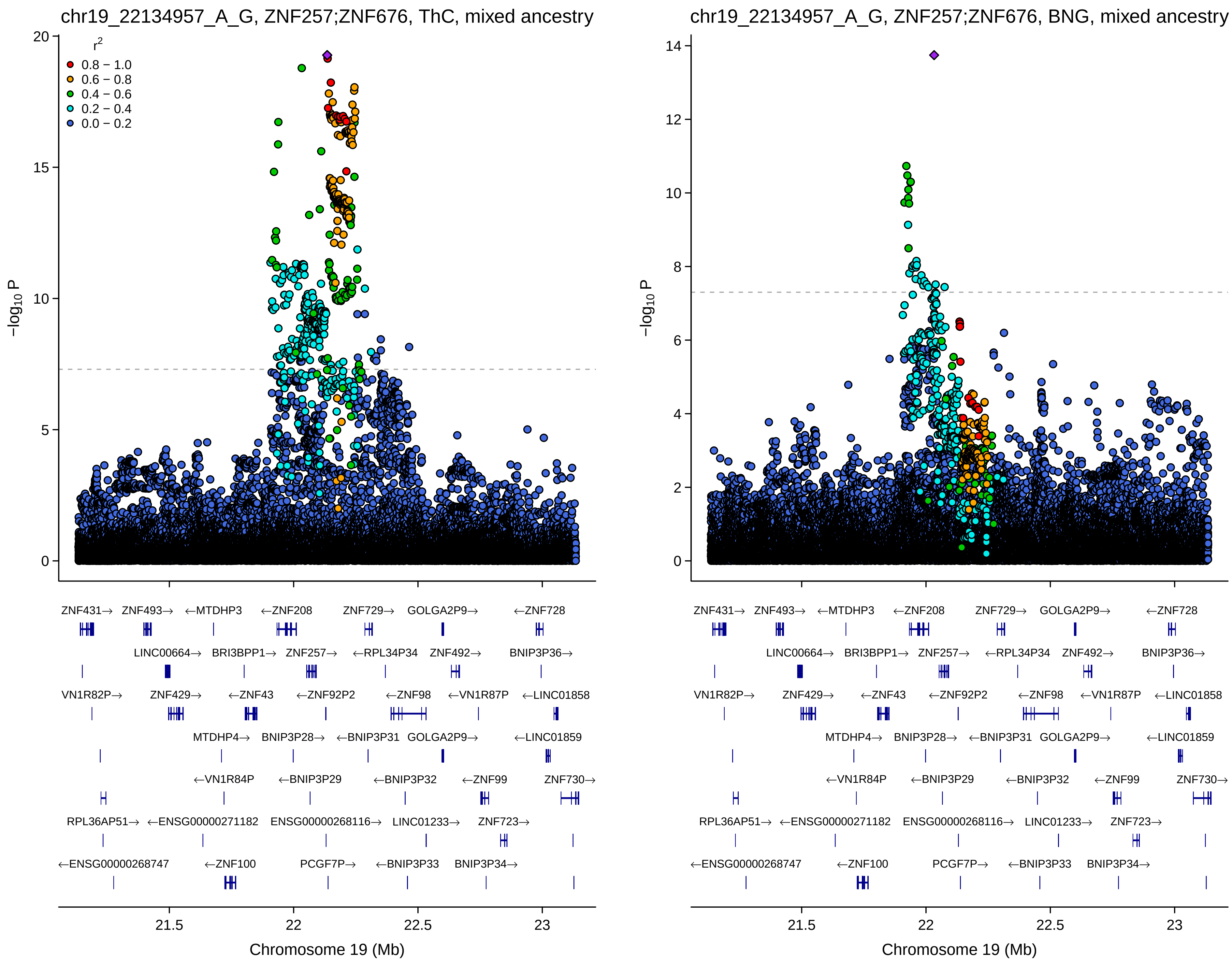

Supplementary Figure 3.1. Locus plots for loci significantly associated with Graves' disease but not hypothyroidism. The left plot displays -log10(p-values) for variants from the Graves' disease meta-analysis, the right plot displays -log10(p-values) for variants within the same genomic region from the hypothyroidism meta-analysis. The lead variant is shown with a purple diamond. Genome wide significance is indicated by the dashed horizontal line at -log10(5e-8).

Supplementary Figure 3.1

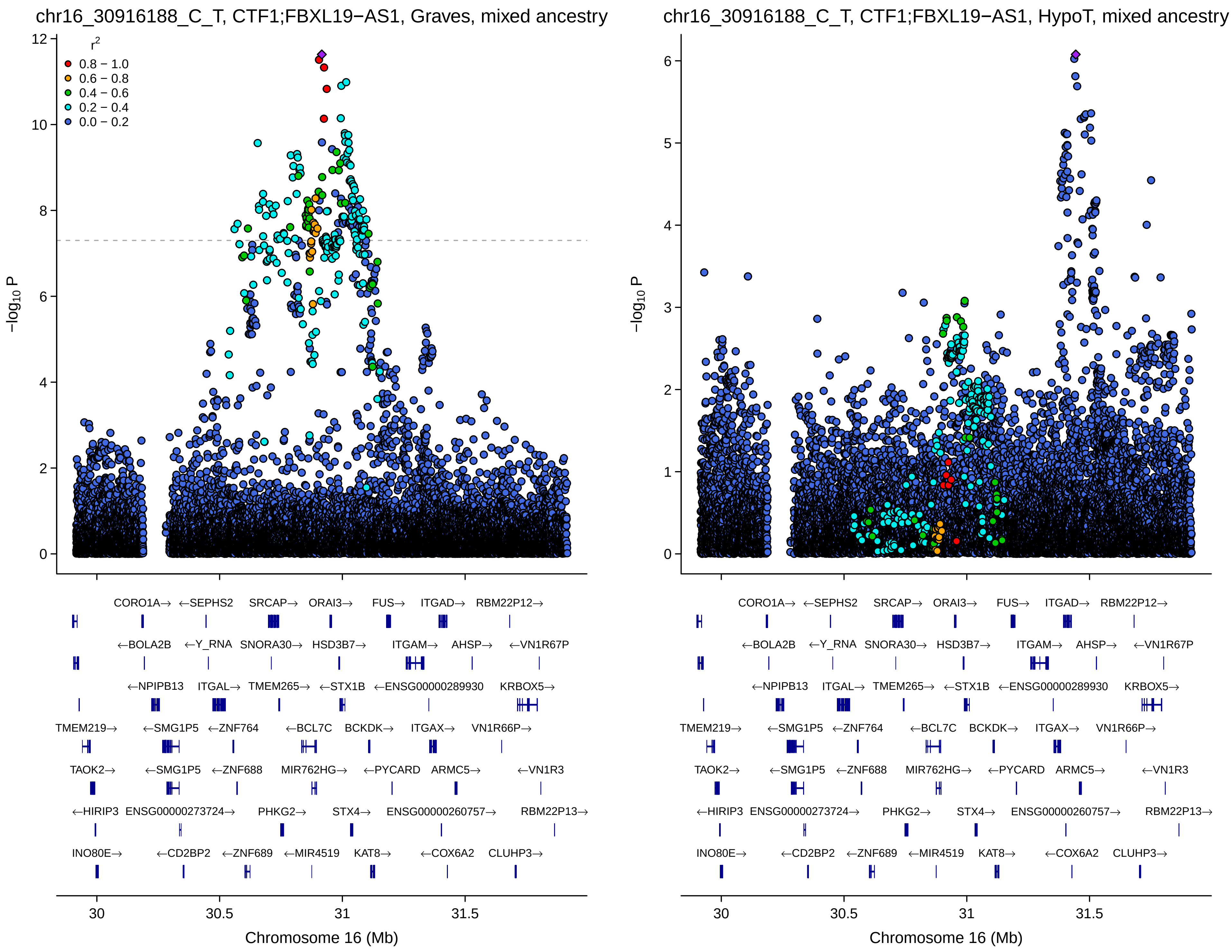

Supplementary Figure 3.1

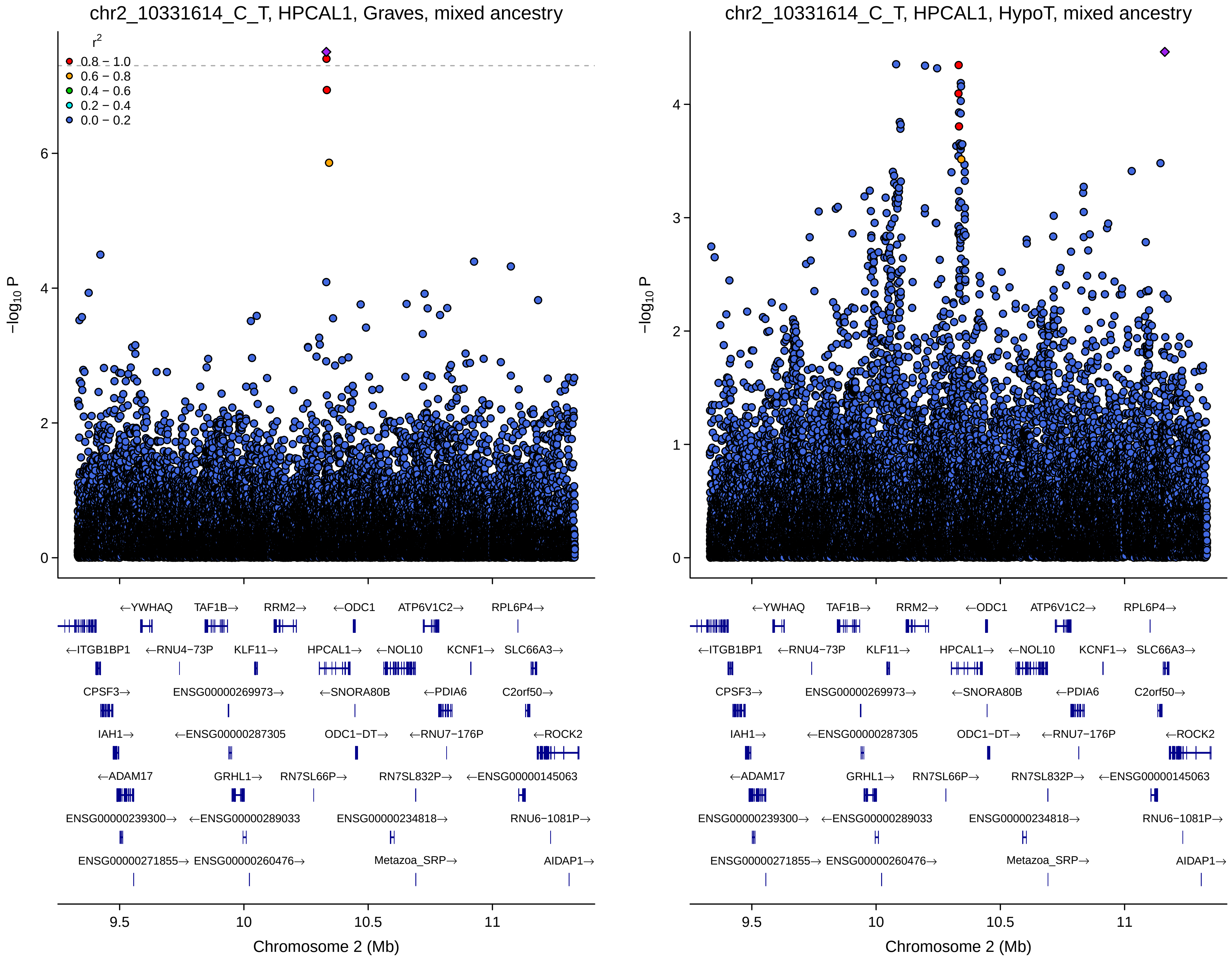

Supplementary Figure 3.1

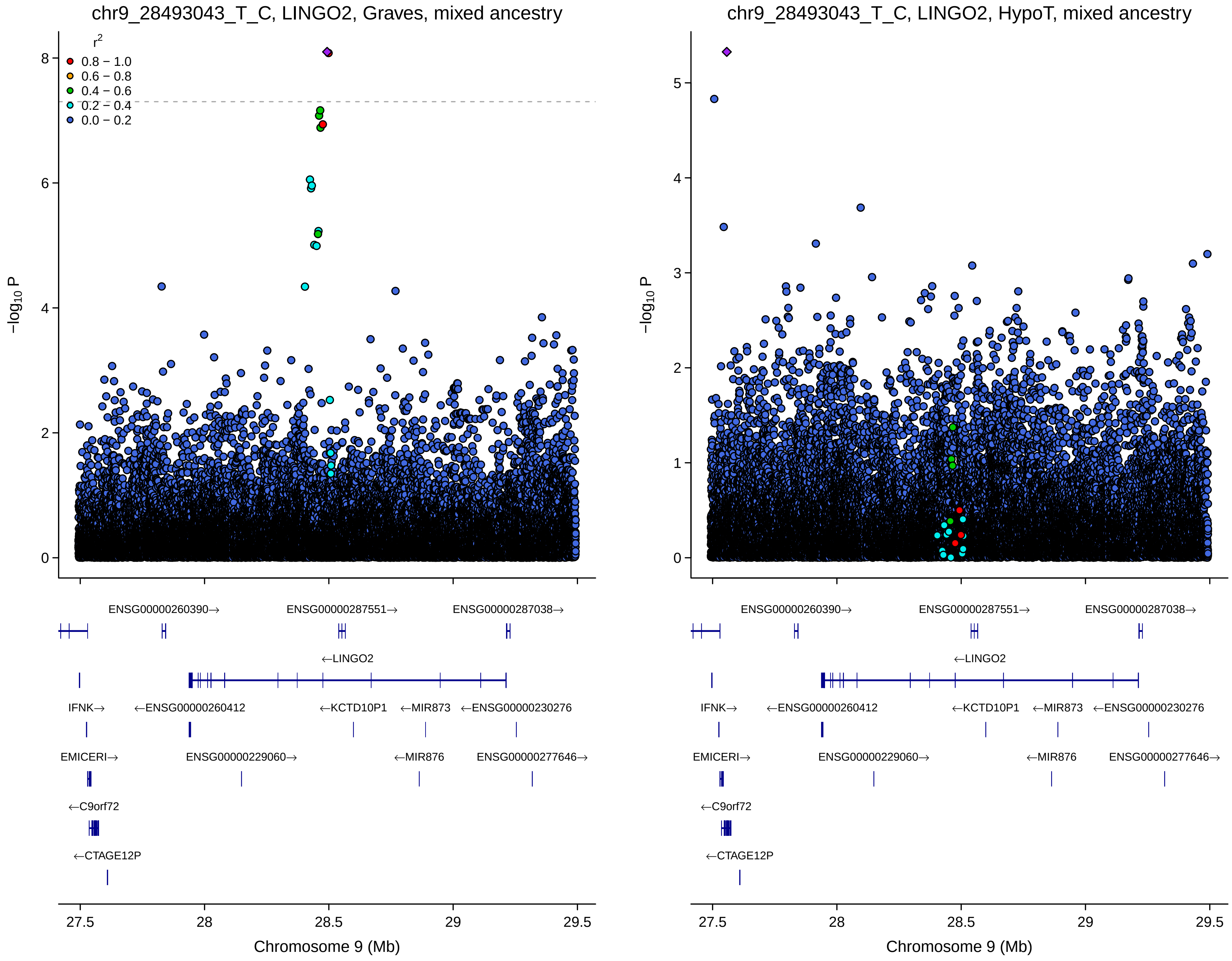

Supplementary Figure 3.1

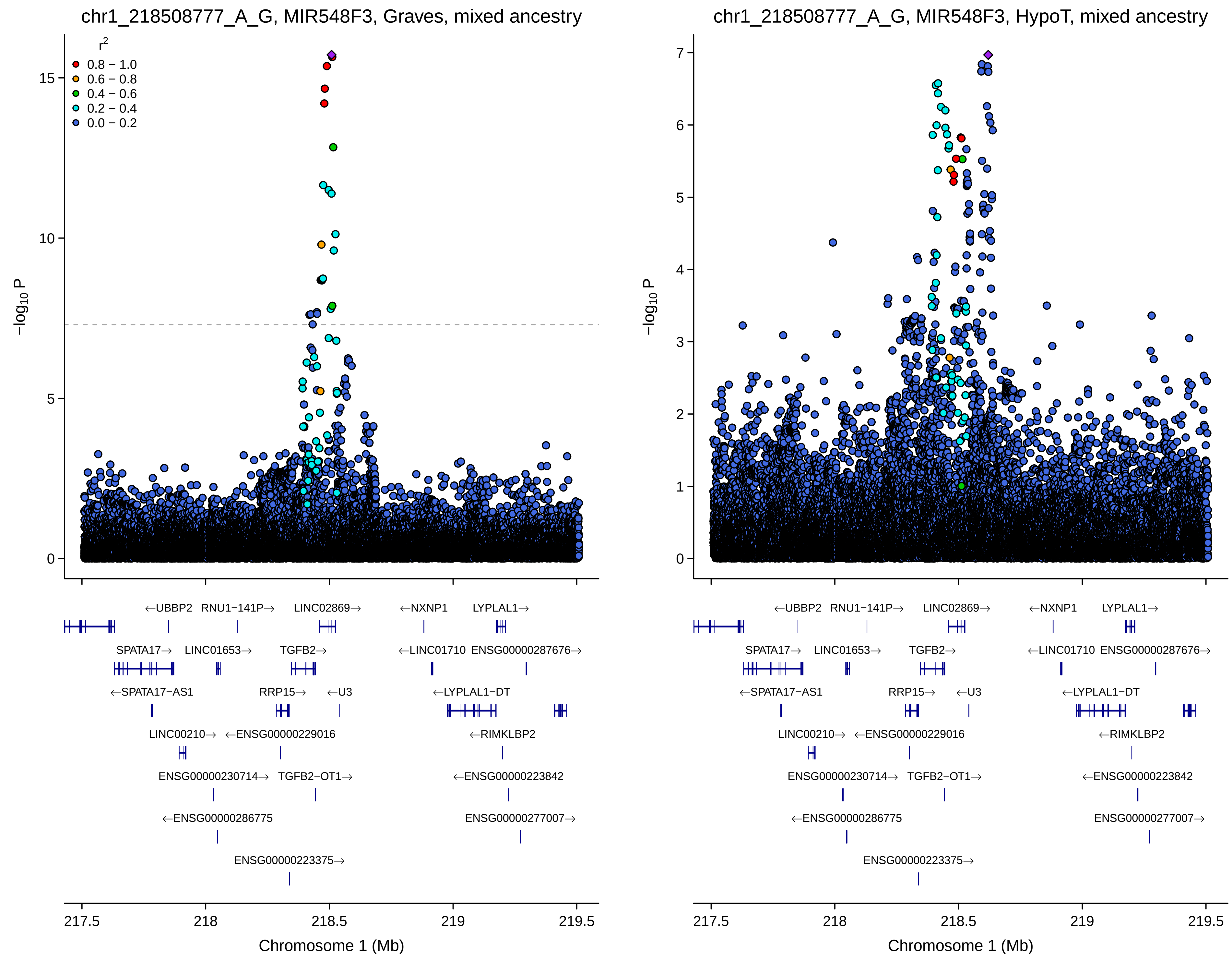

Supplementary Figure 3.1

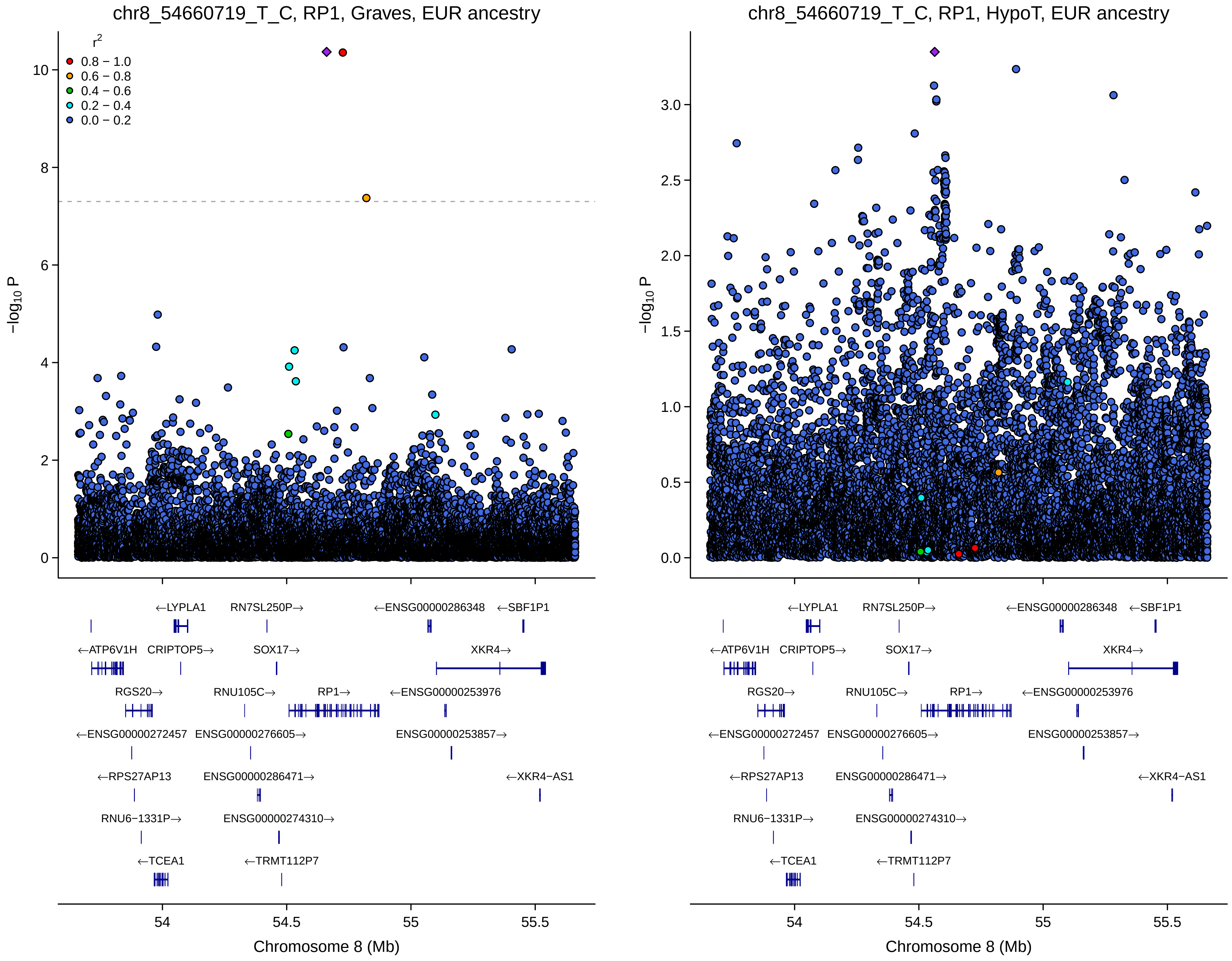

Supplementary Figure 3.1

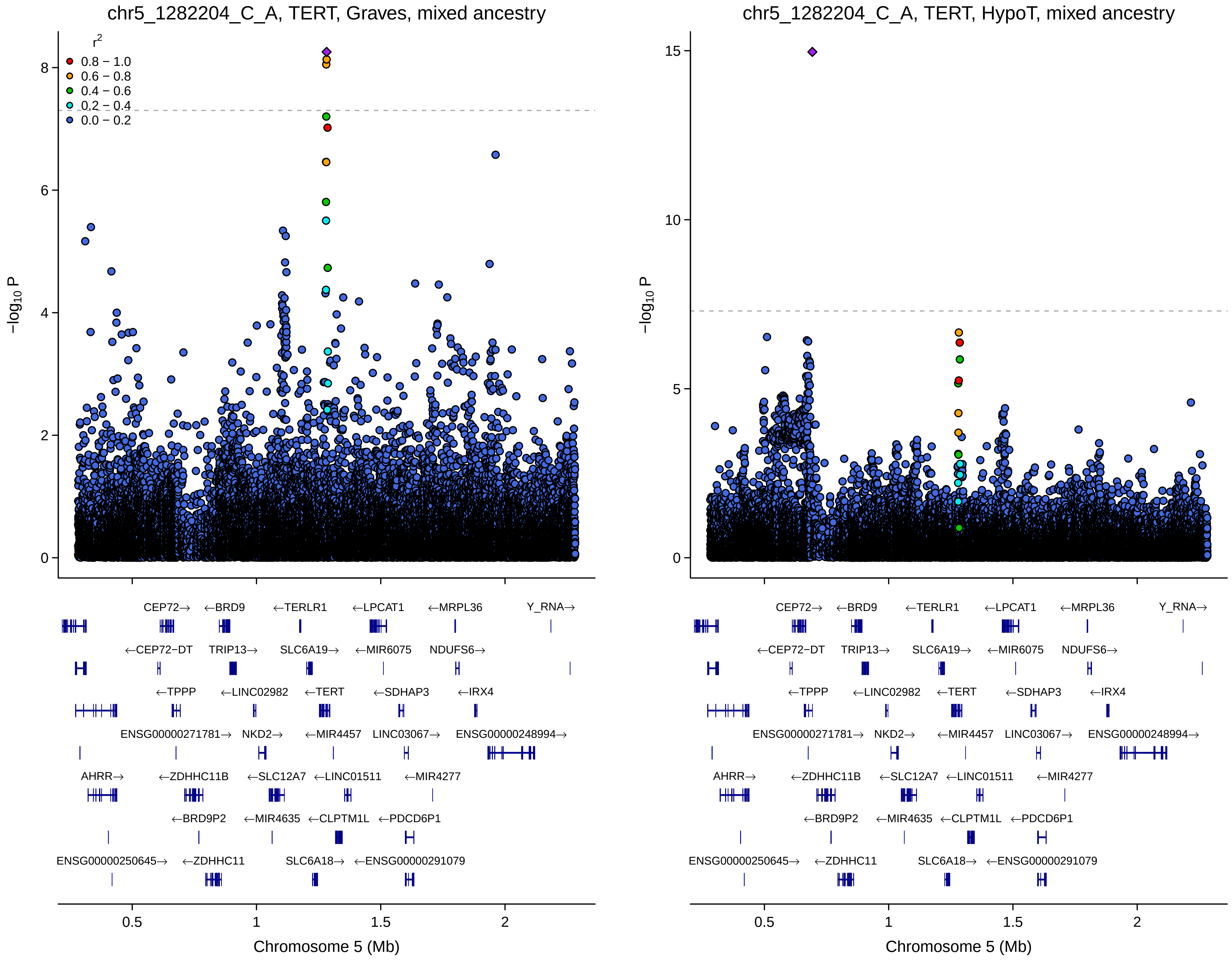

Supplementary Figure 3.1

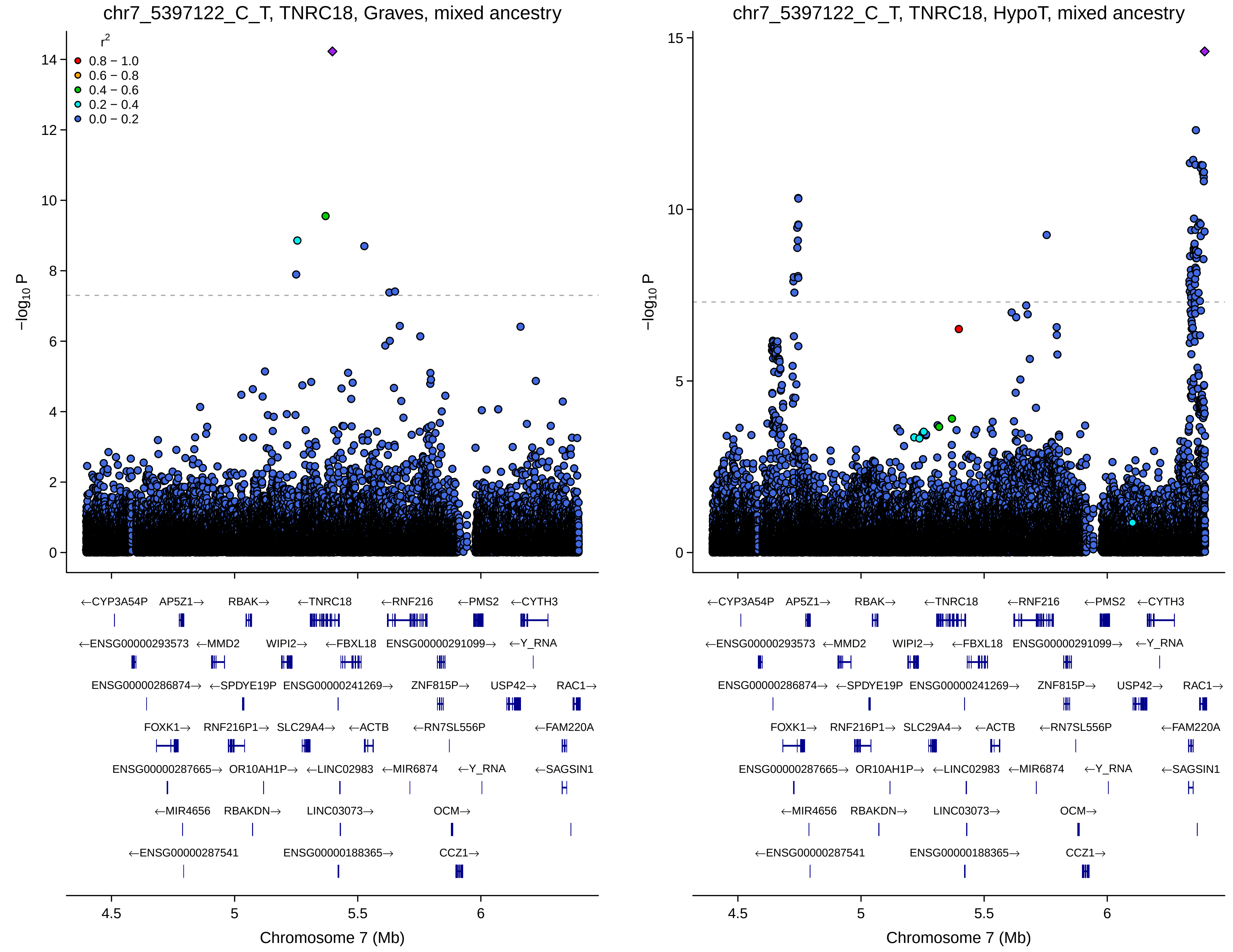

Supplementary Figure 3.1

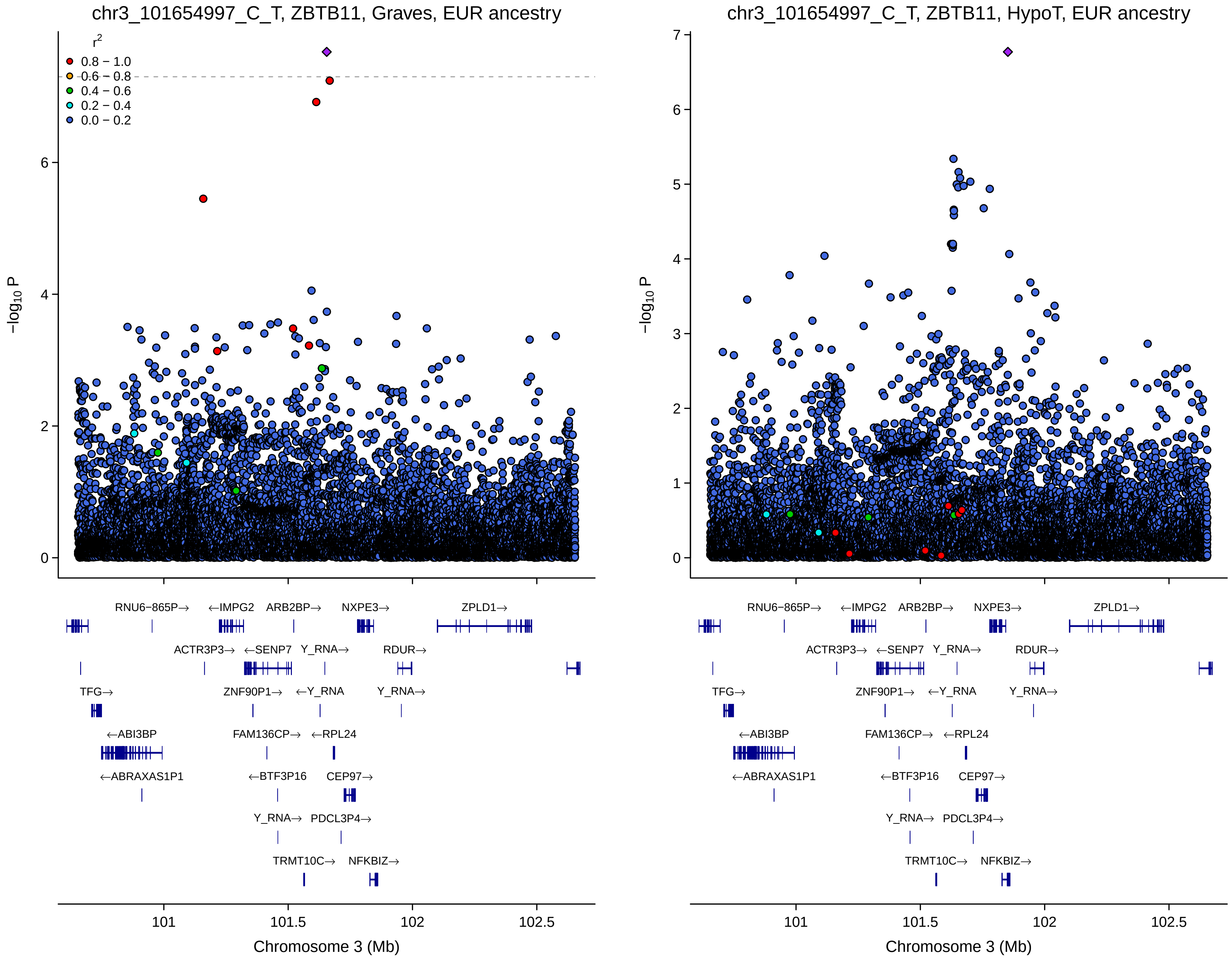

Supplementary Figure 3.1

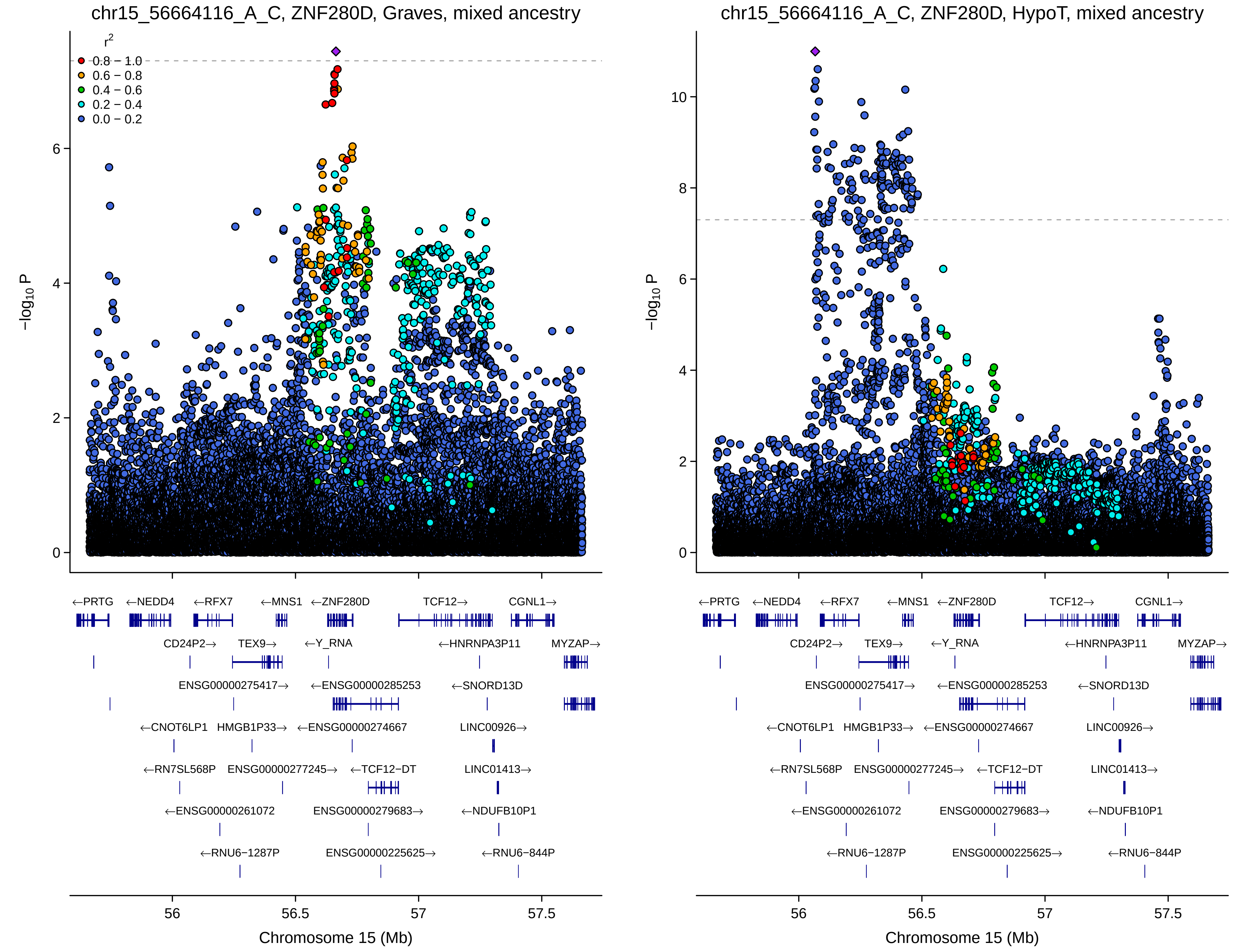

Supplementary Figure 3.2. Locus plots for loci significantly associated with both Graves' disease and hypothyroidism. The left plot displays -log10(p-values) for variants from the Graves' disease meta-analysis, the right plot displays -log10(p-values) for variants within the same genomic region from the hypothyroidism meta-analysis. The lead variant is shown with a purple diamond. Genome wide significance is indicated by the dashed horizontal line at -log10(5e-8).

Supplementary Figure 3.2

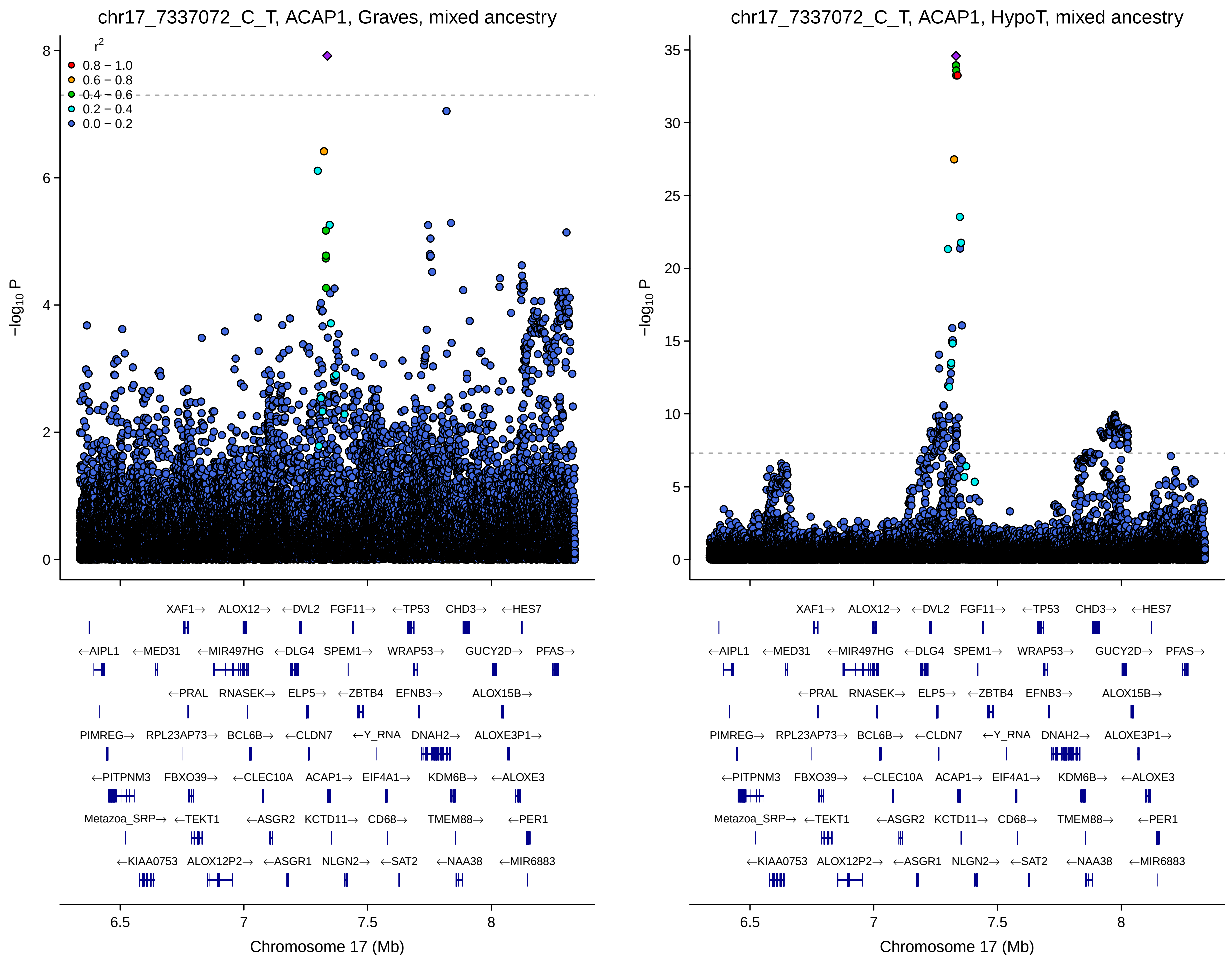

Supplementary Figure 3.2

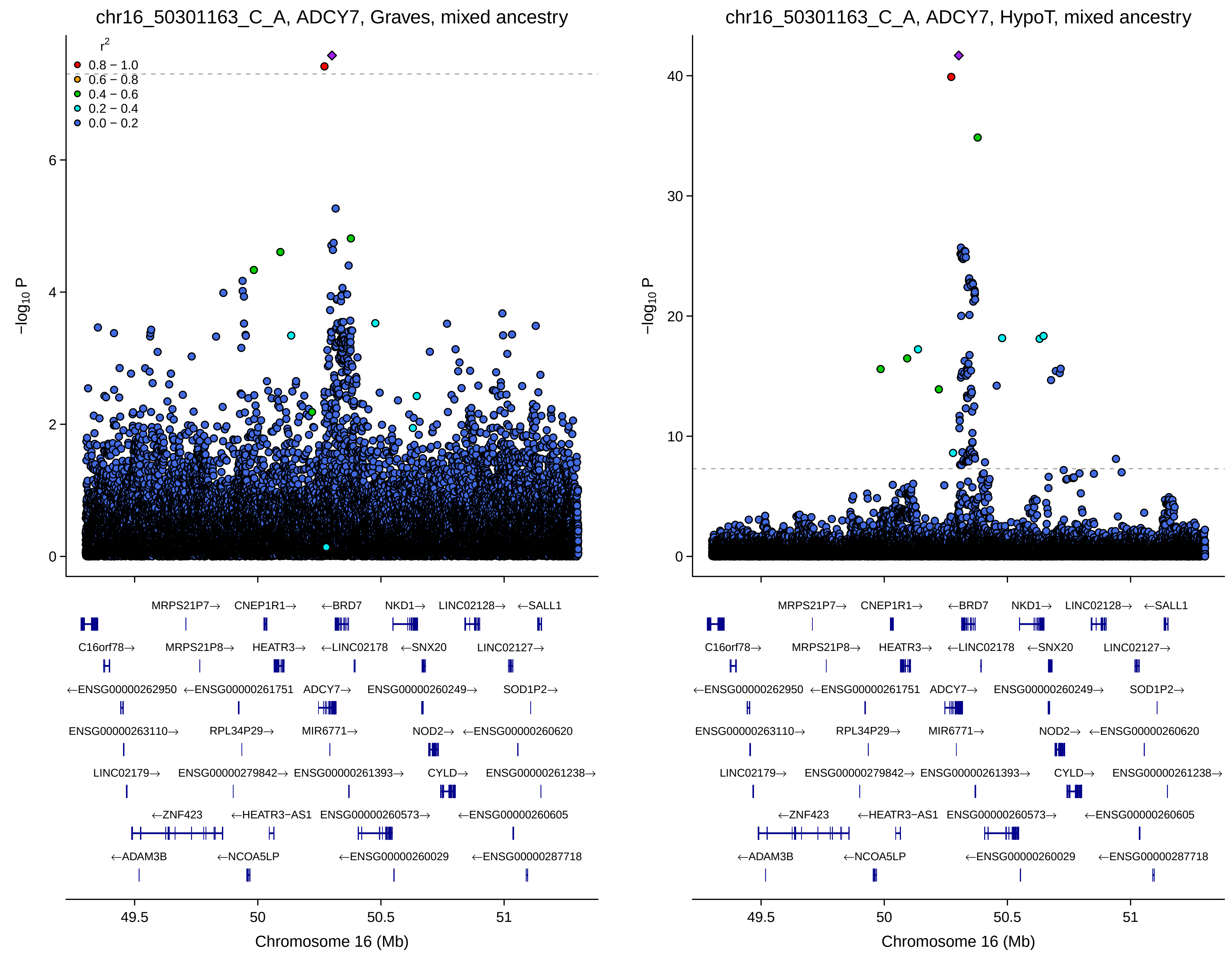

Supplementary Figure 3.2

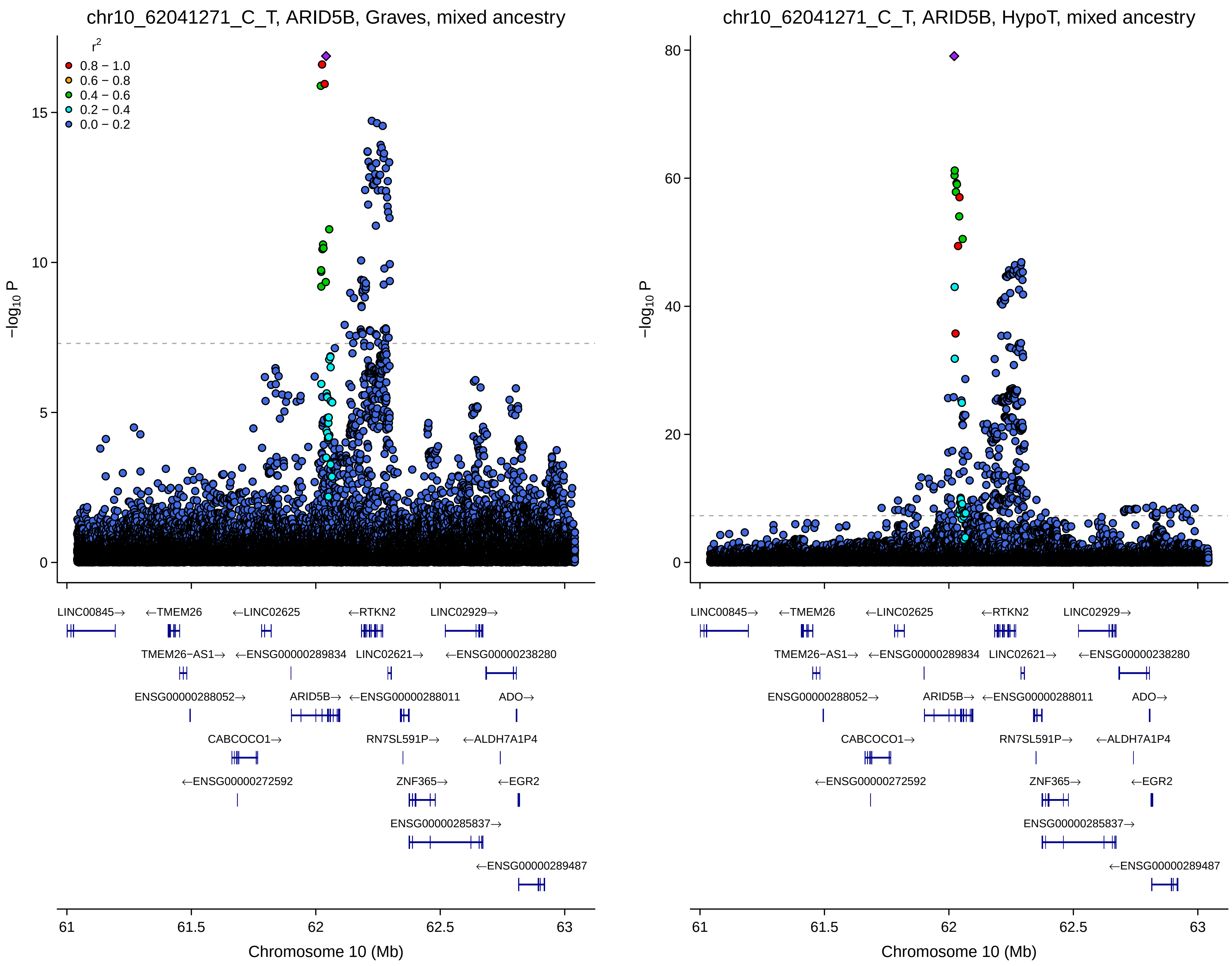

Supplementary Figure 3.2

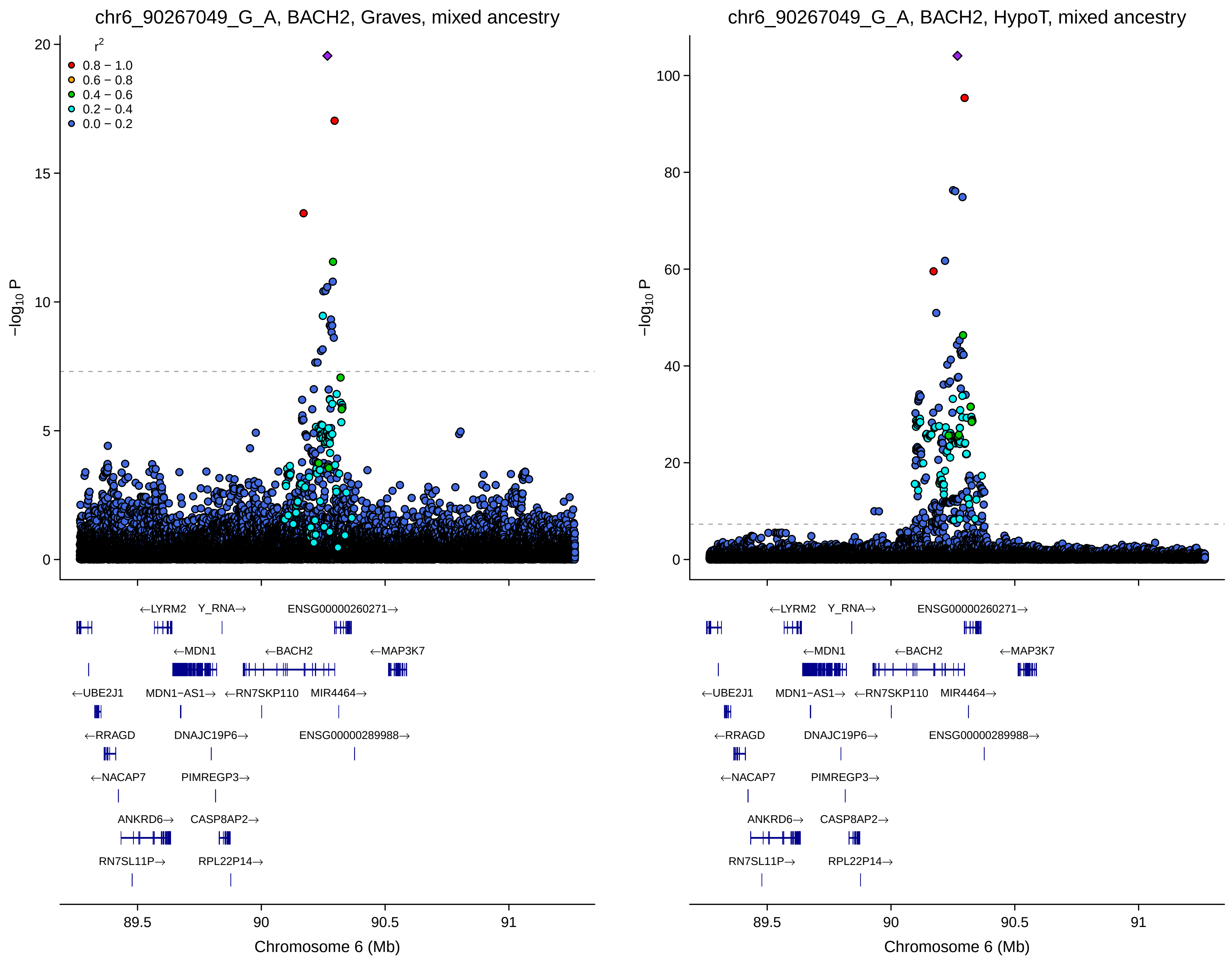

Supplementary Figure 3.2

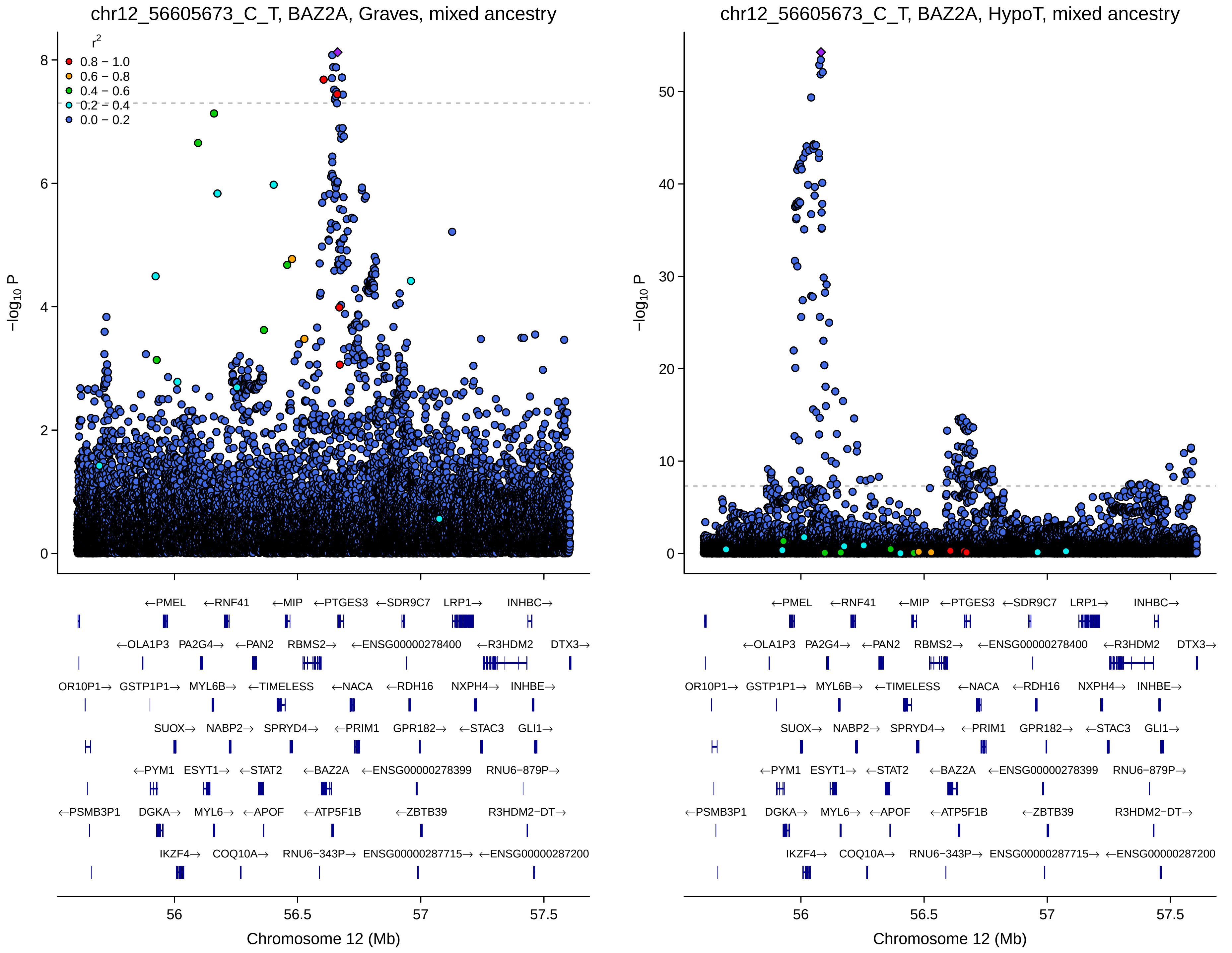

Supplementary Figure 3.2

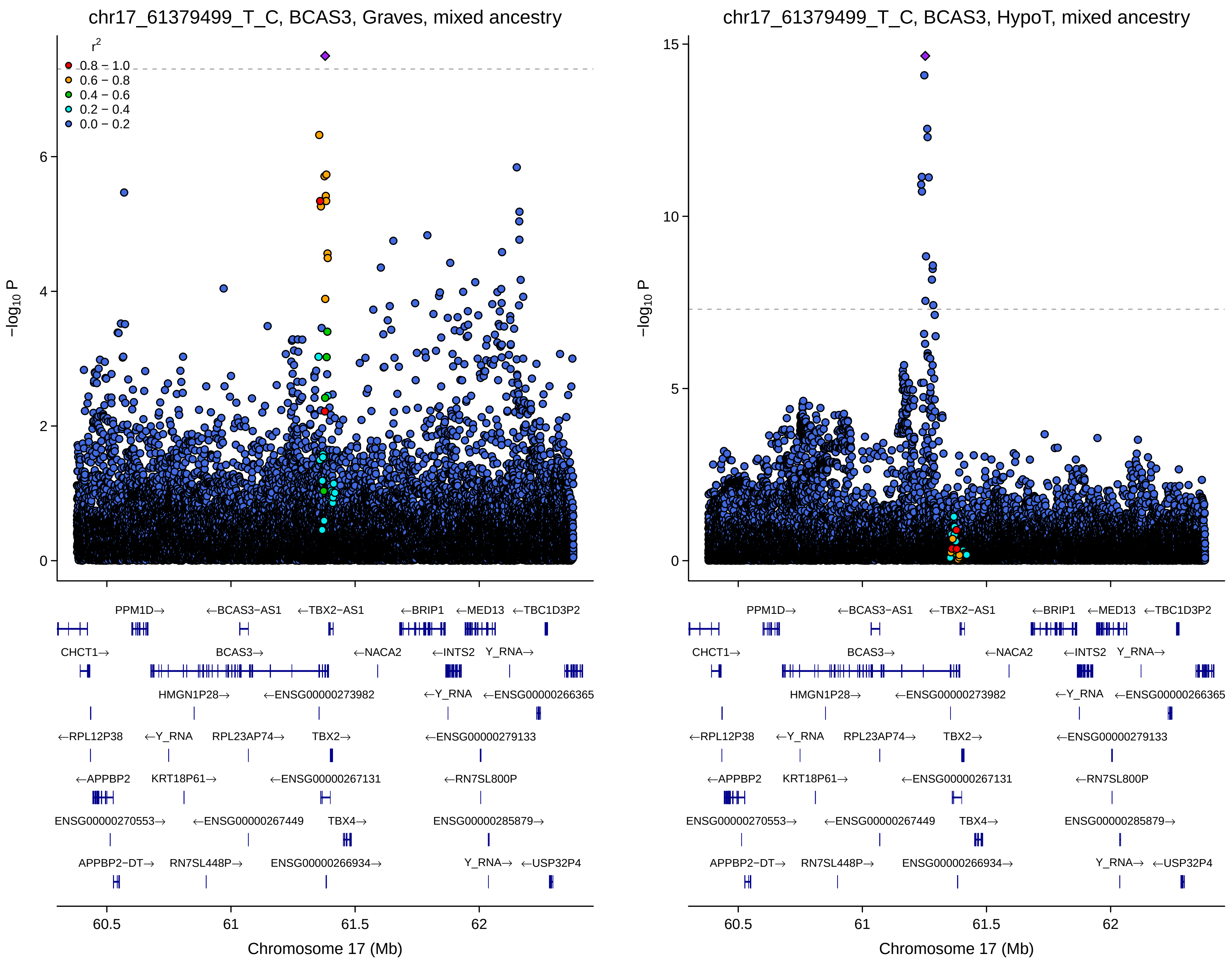

Supplementary Figure 3.2

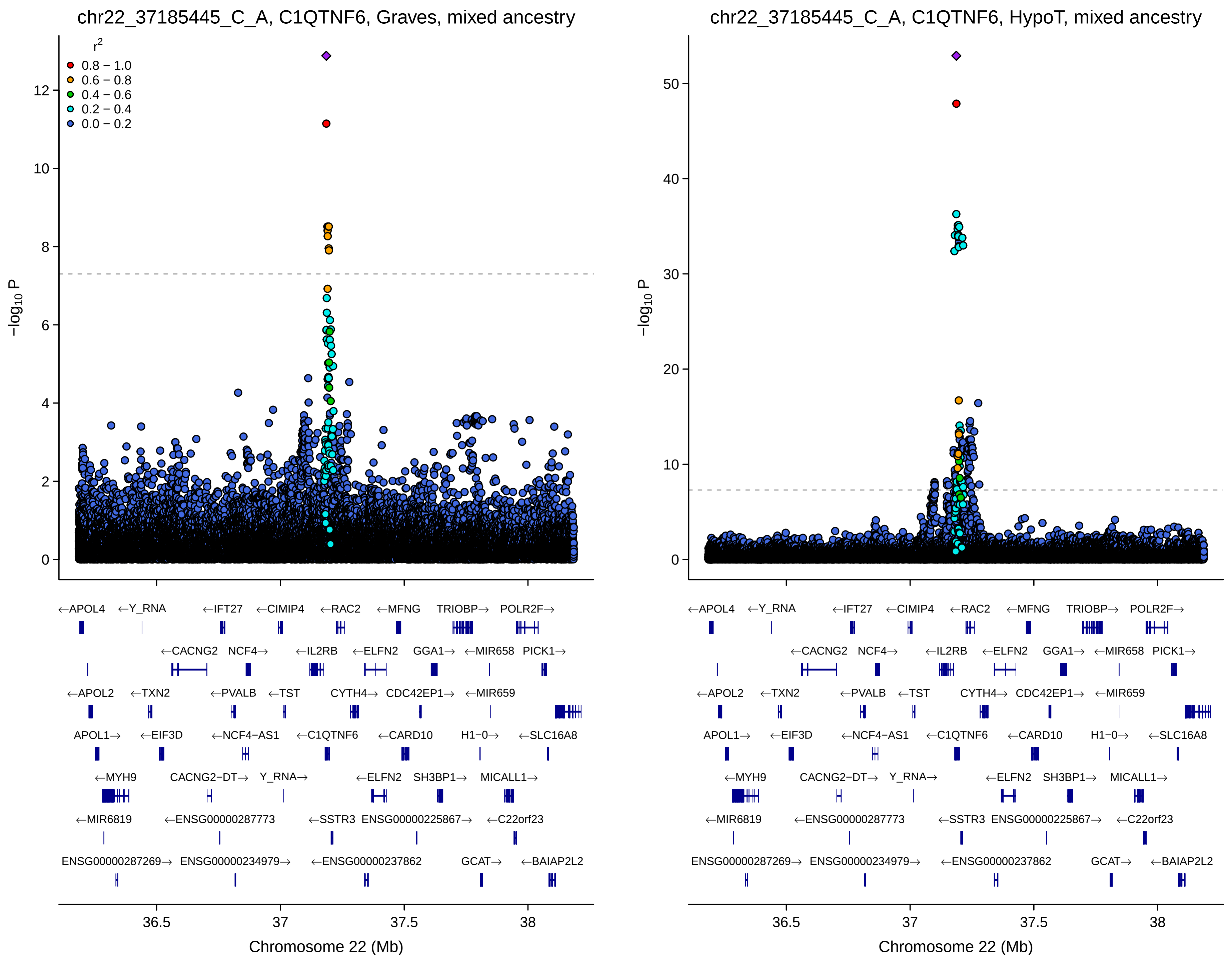

Supplementary Figure 3.2

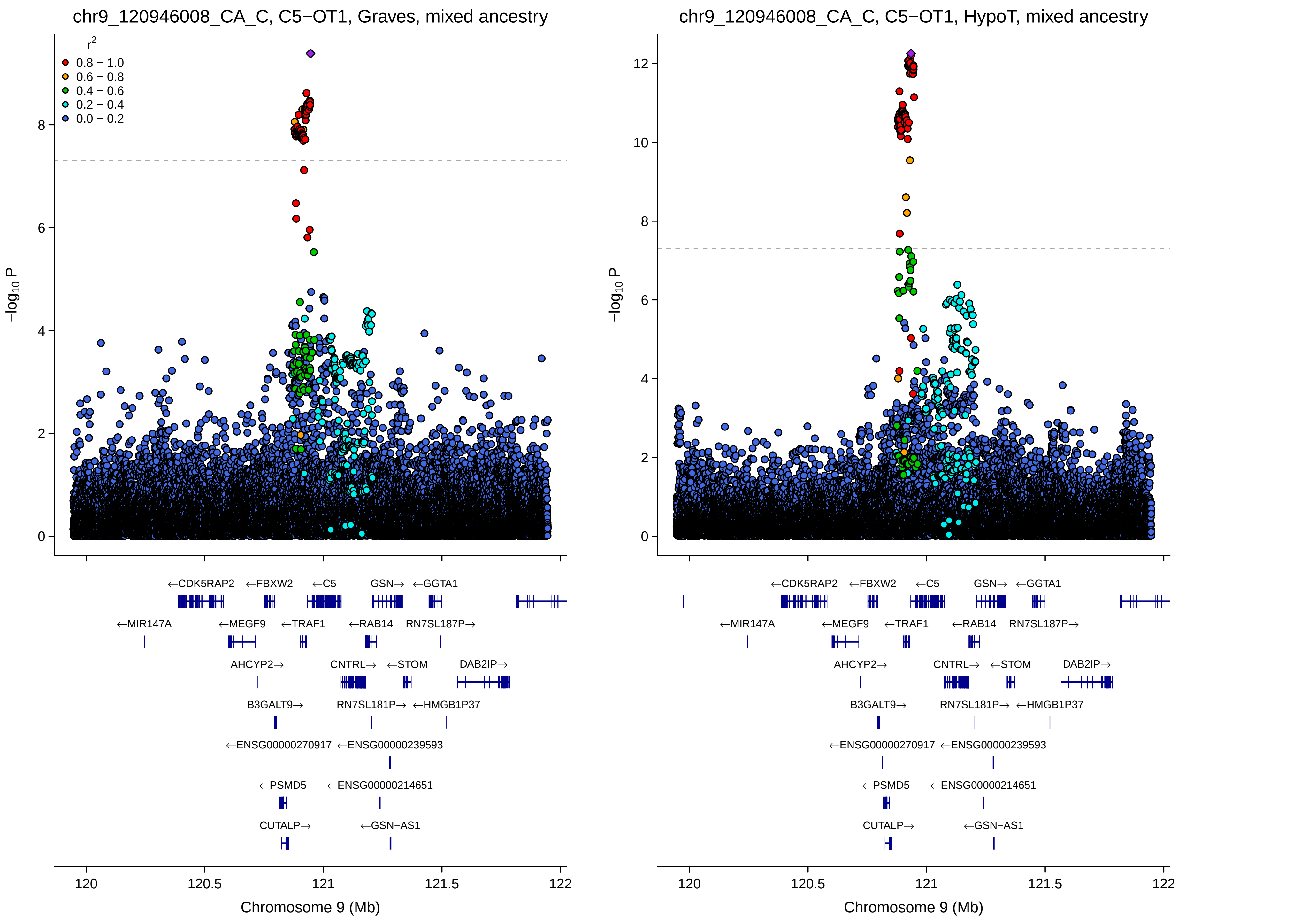

Supplementary Figure 3.2

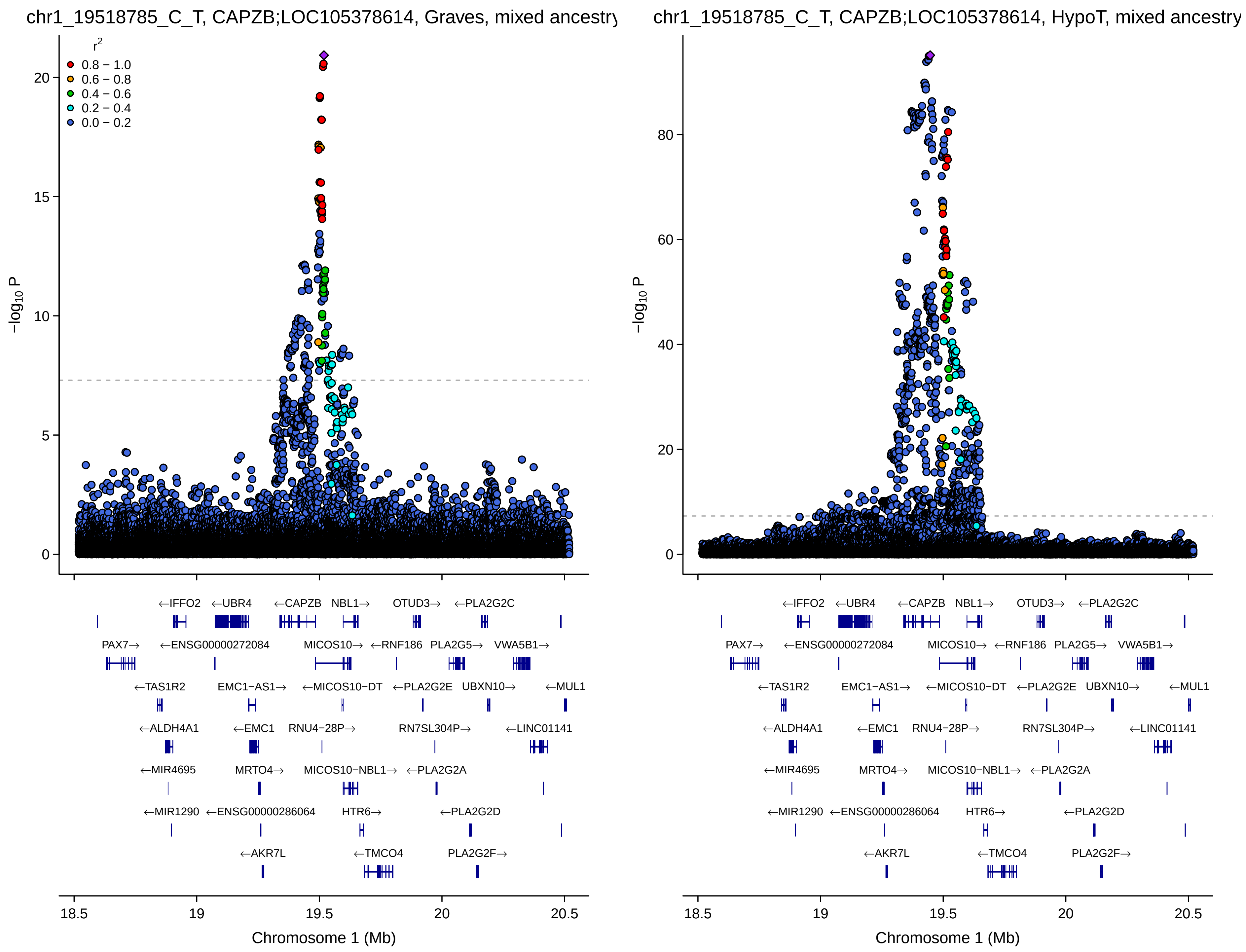

Supplementary Figure 3.2

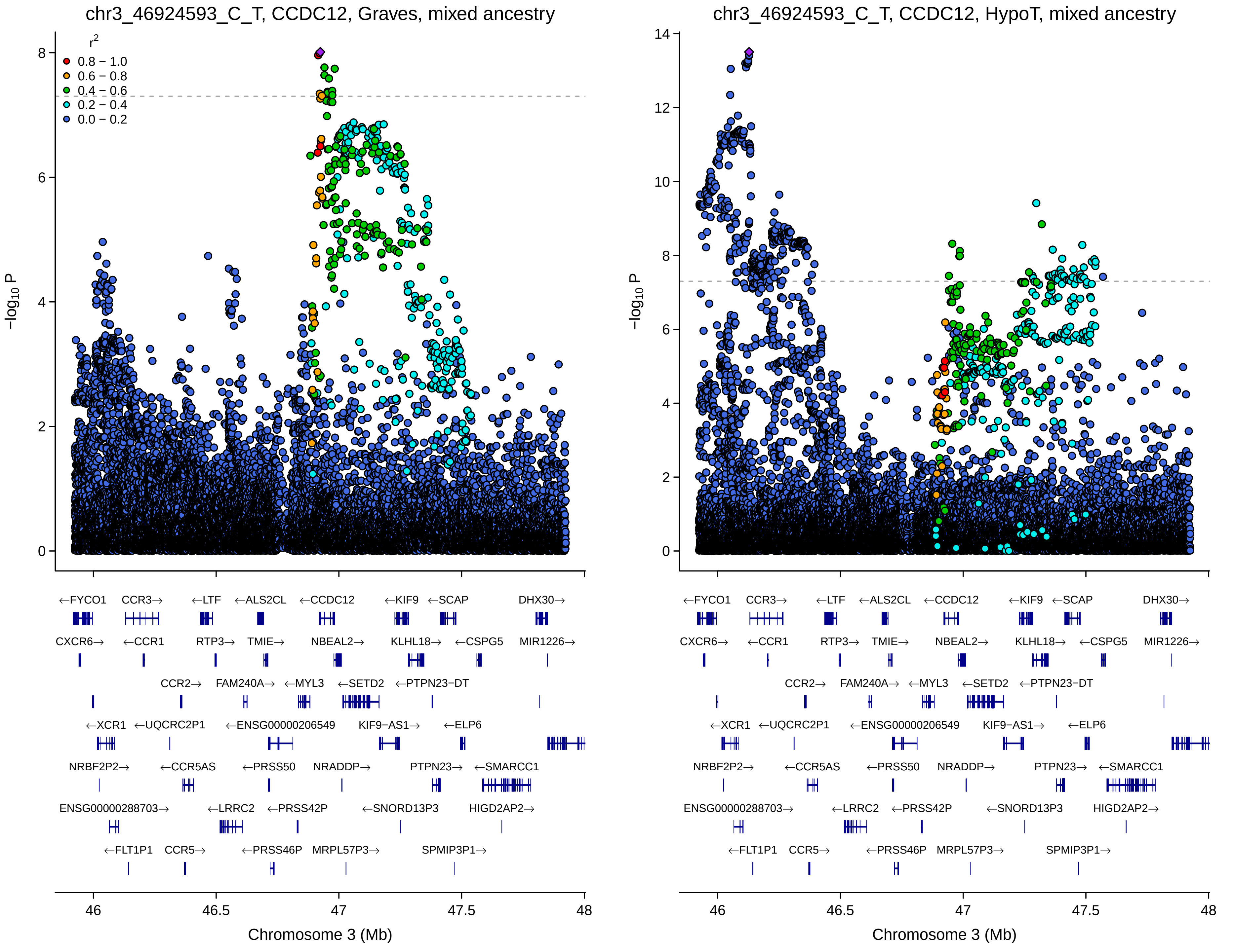

Supplementary Figure 3.2

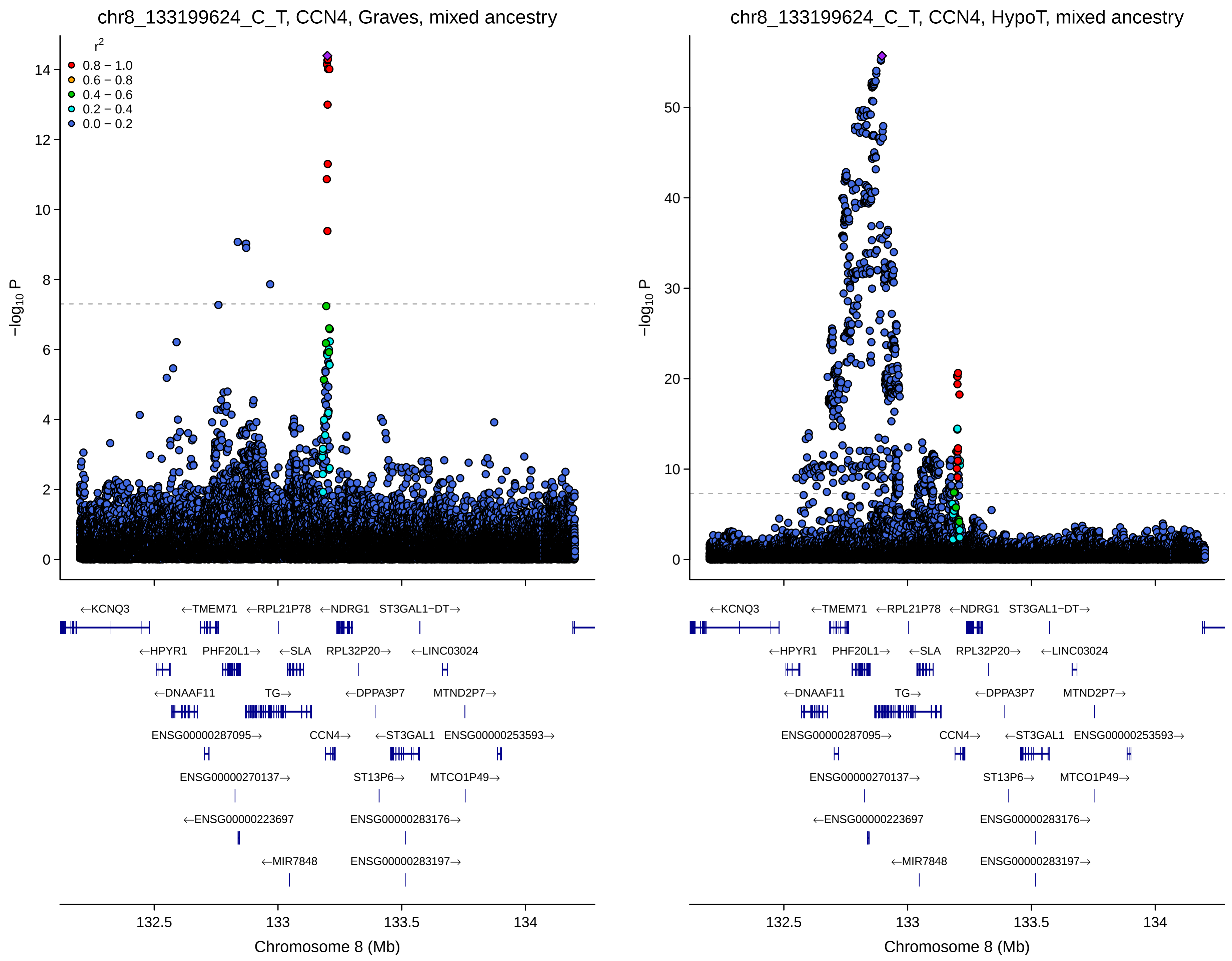

Supplementary Figure 3.2

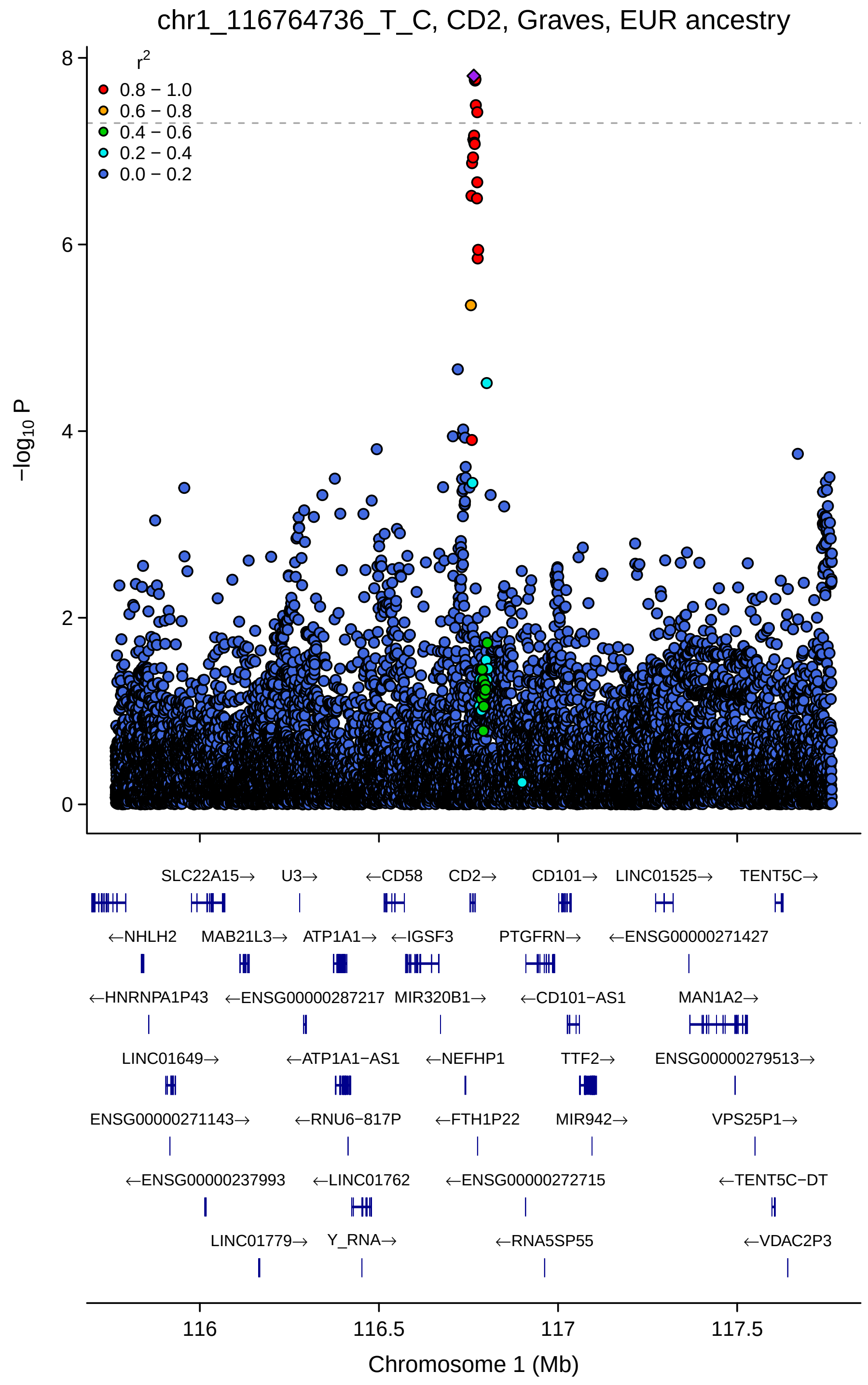

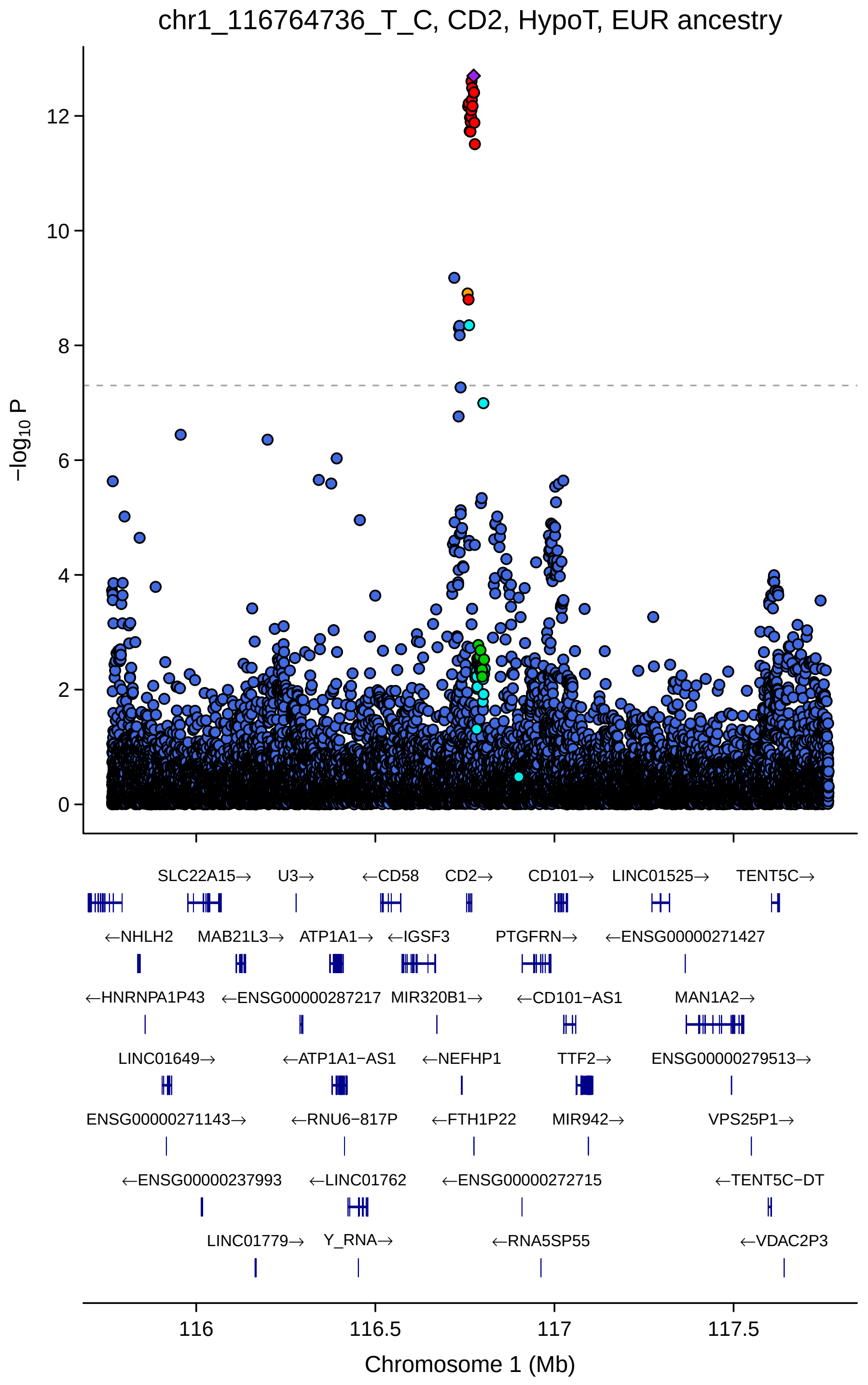

Supplementary Figure 3.2

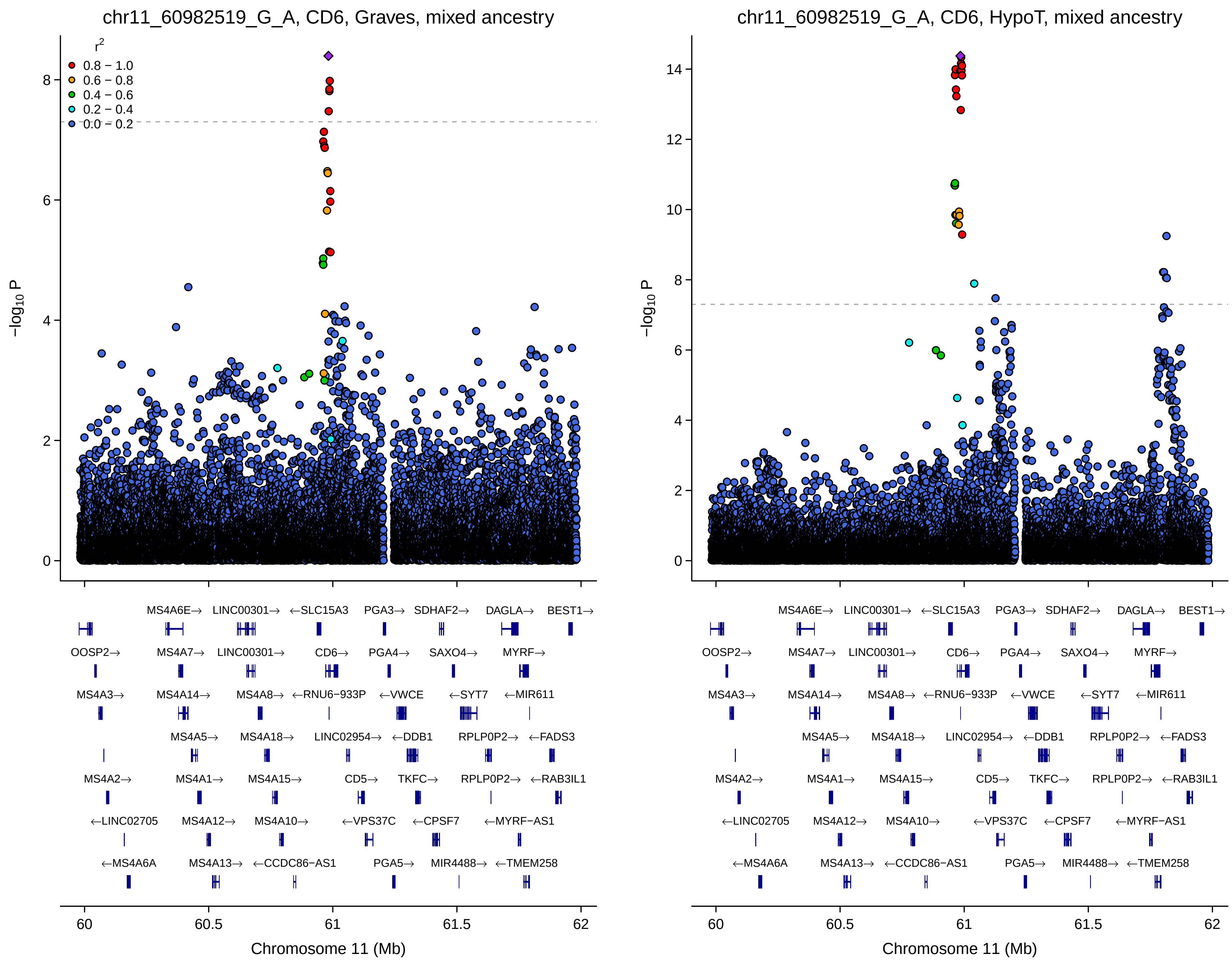

Supplementary Figure 3.2

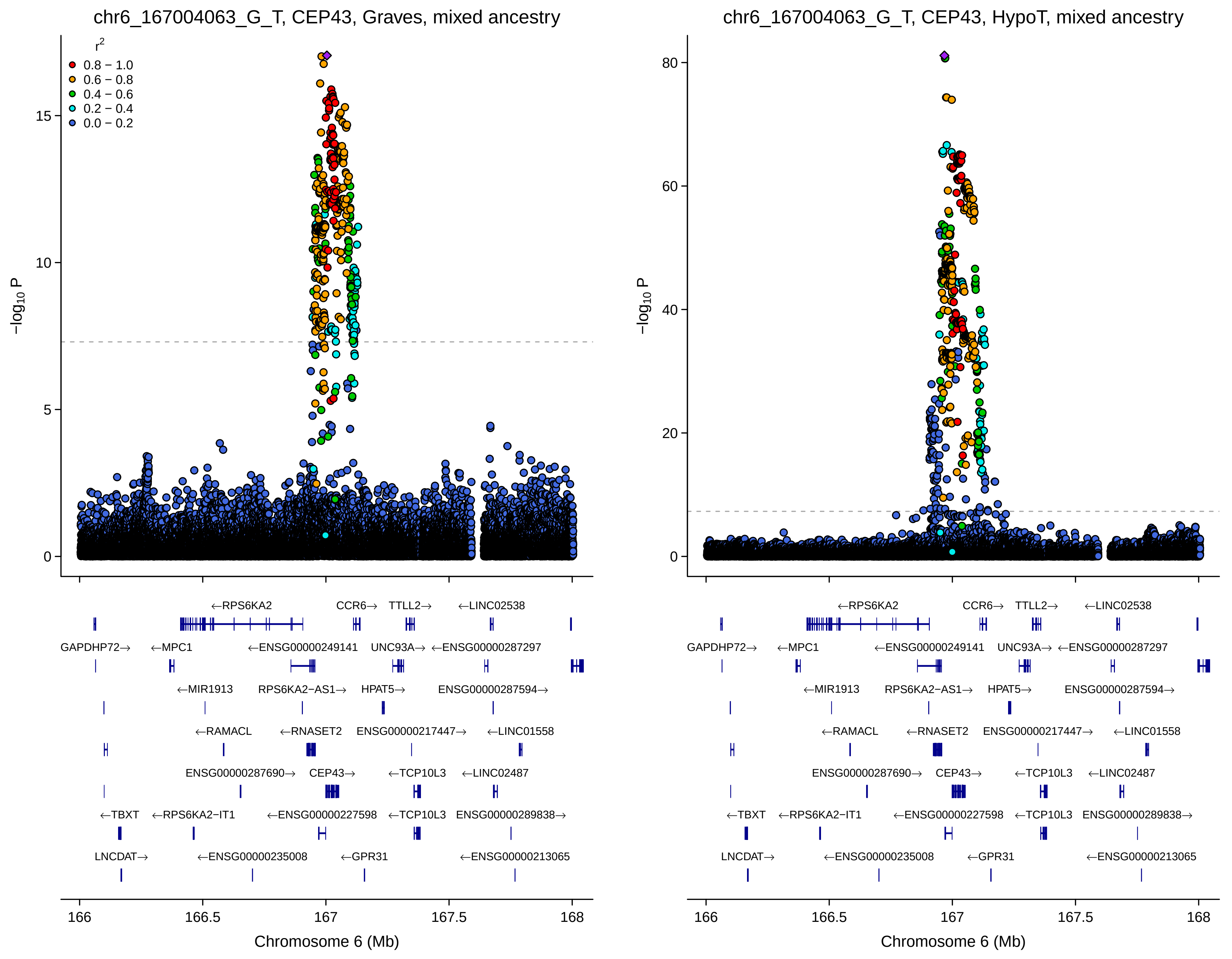

Supplementary Figure 3.2

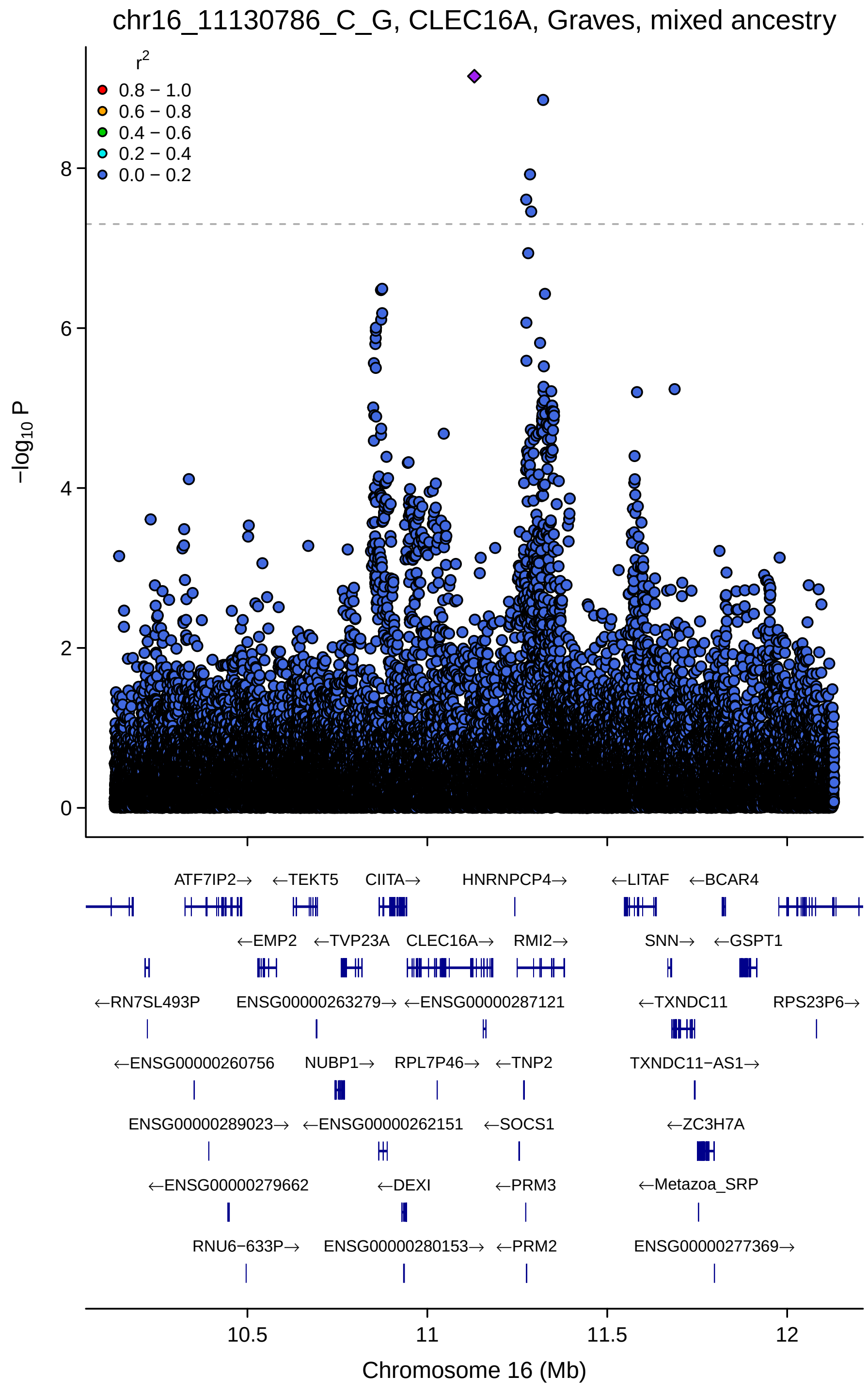

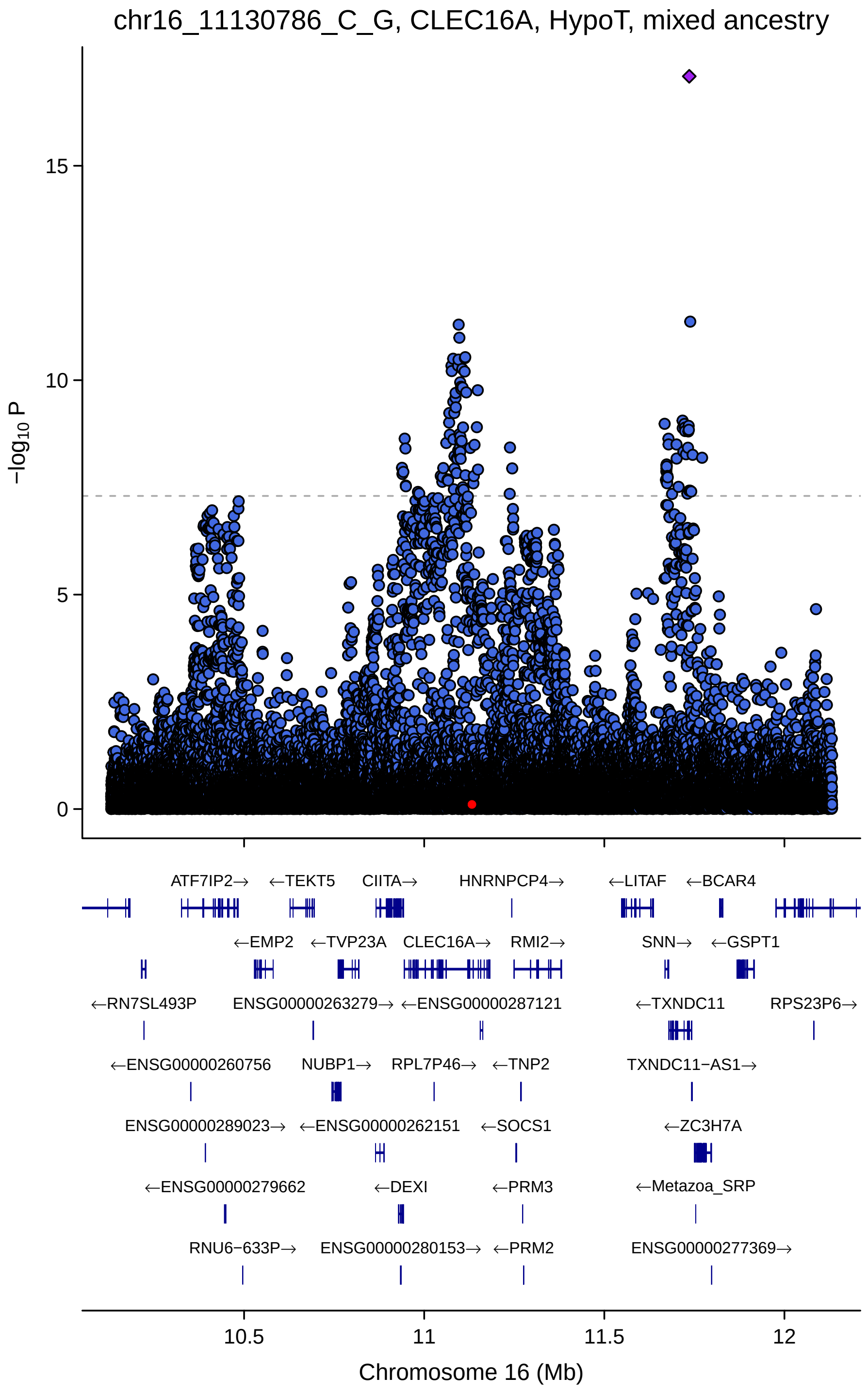

Supplementary Figure 3.2

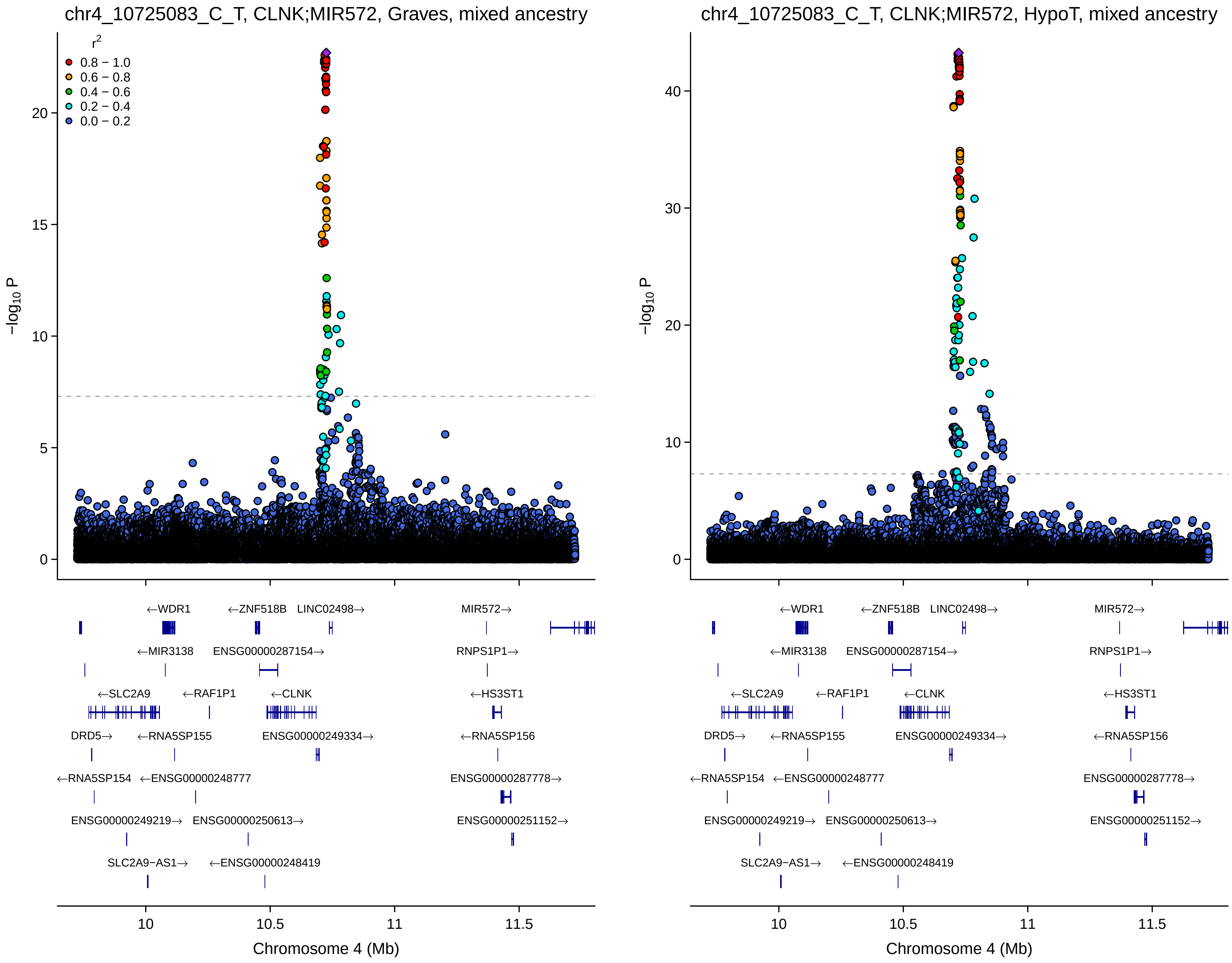

Supplementary Figure 3.2

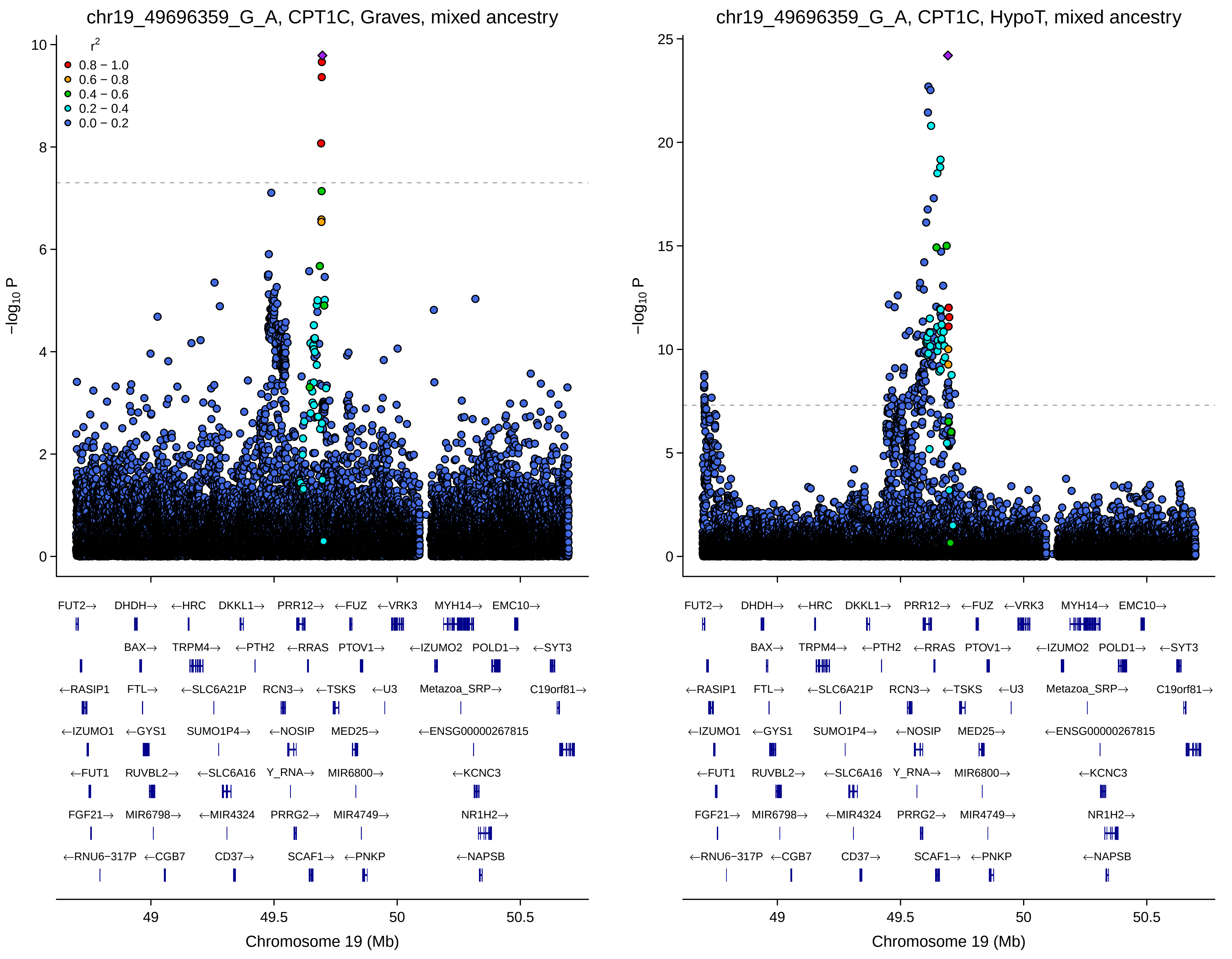

Supplementary Figure 3.2

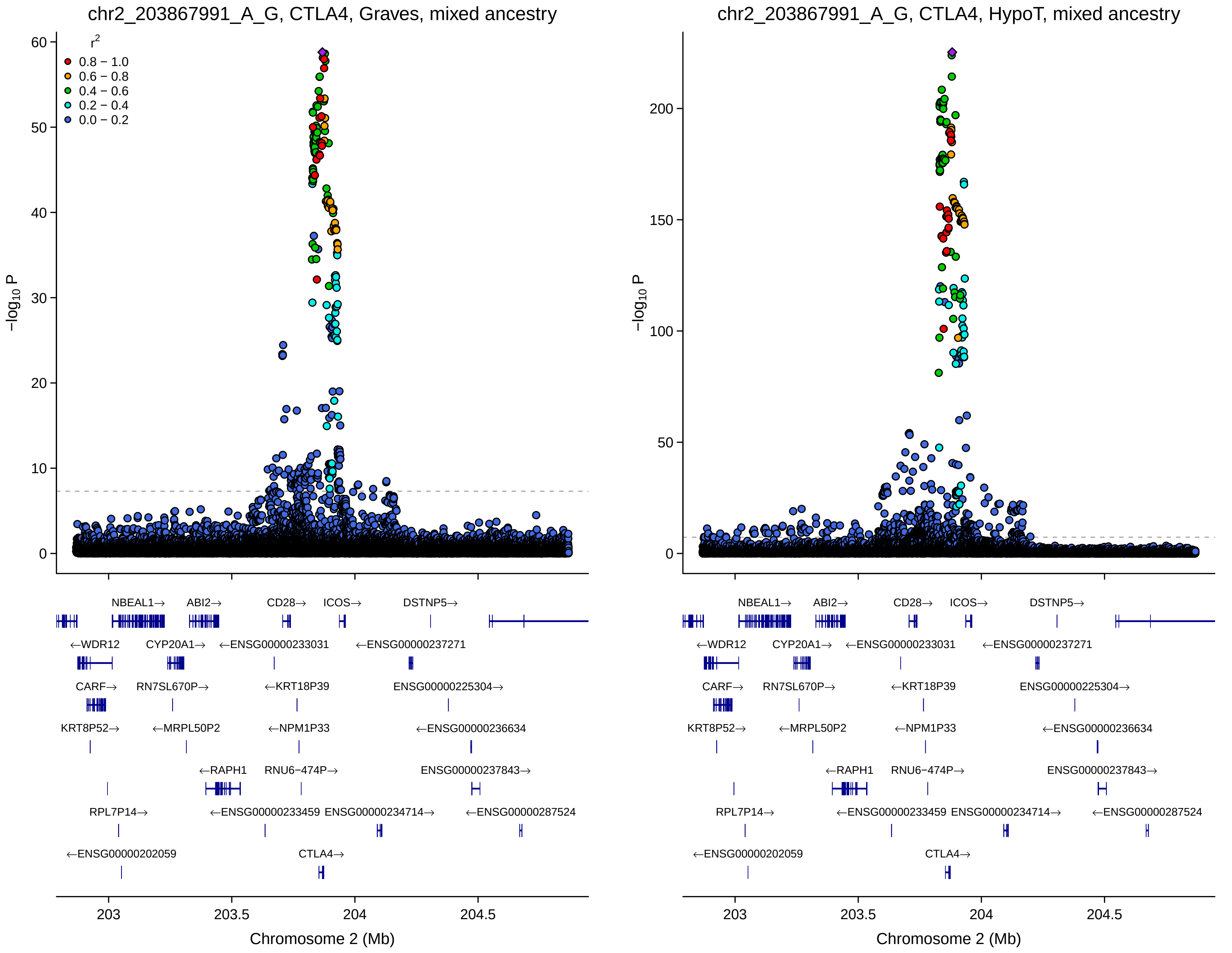

Supplementary Figure 3.2

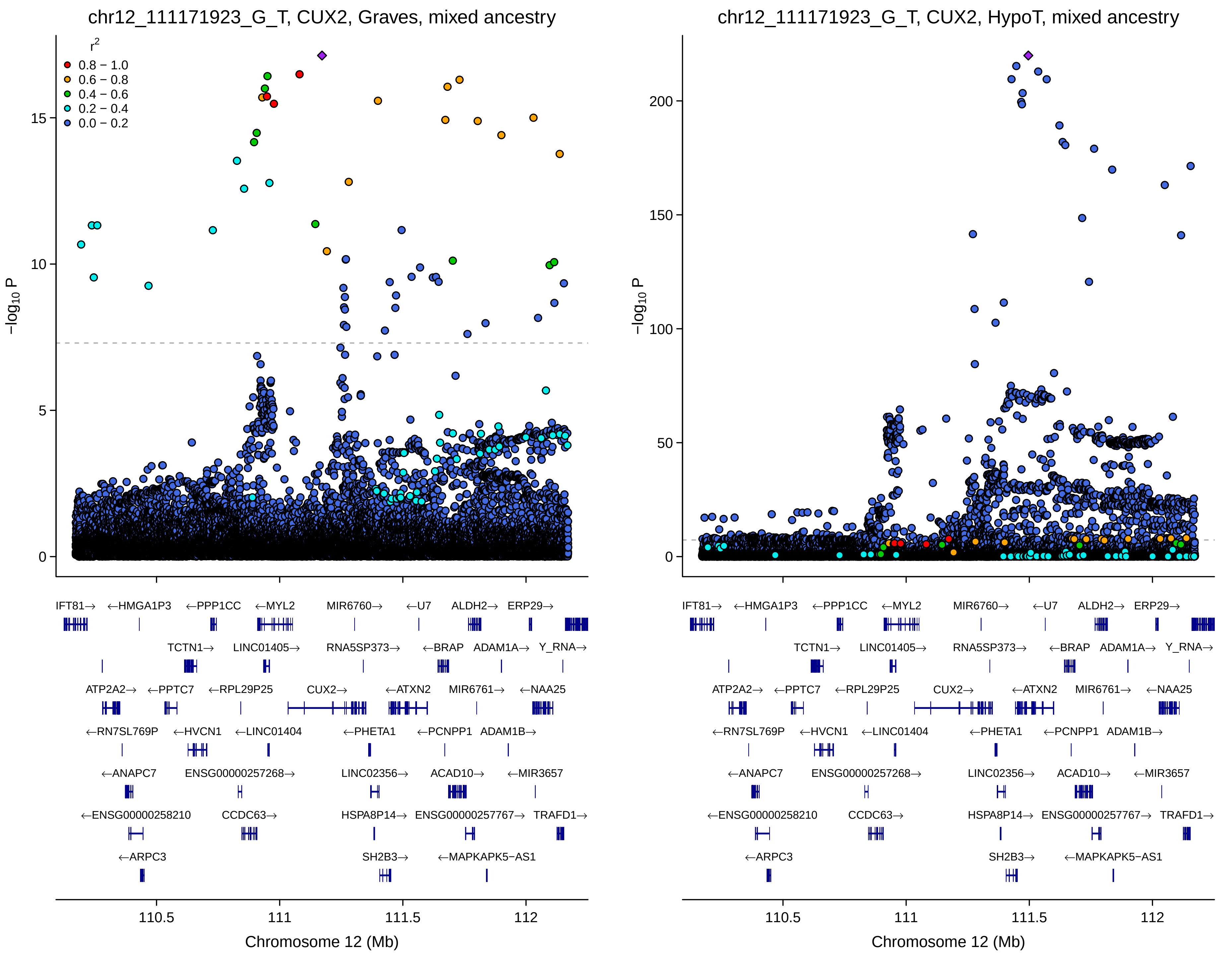

Supplementary Figure 3.2

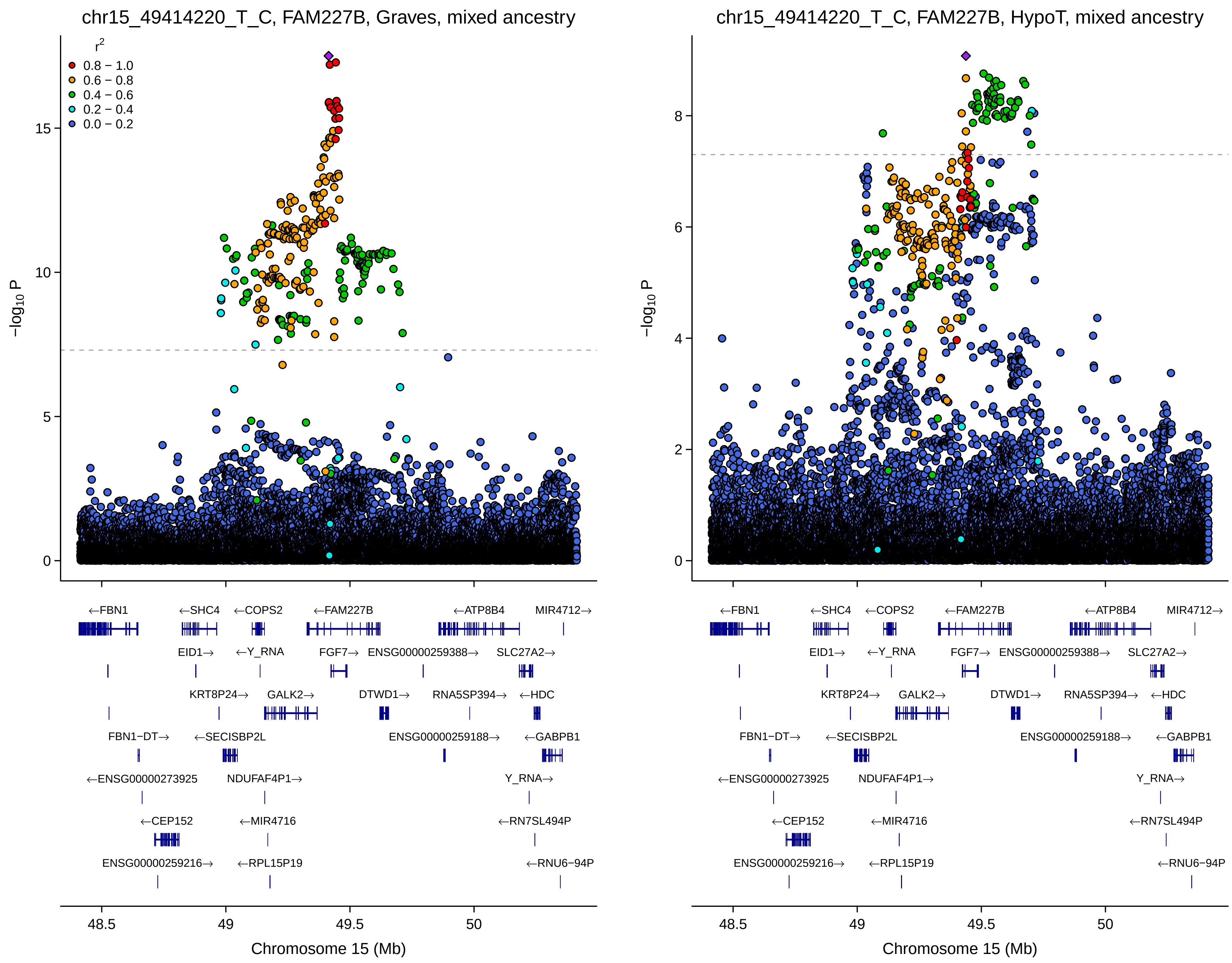

Supplementary Figure 3.2

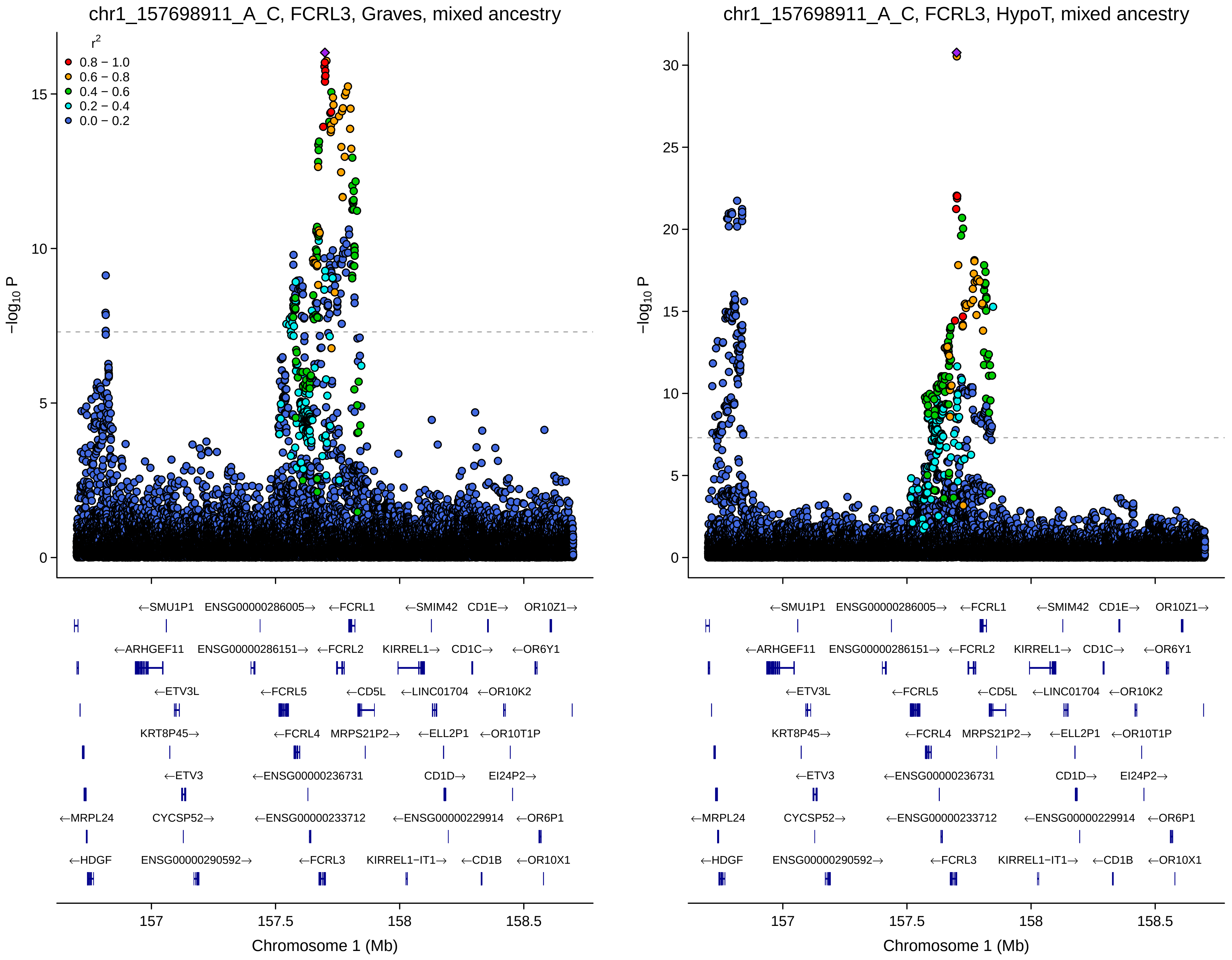

Supplementary Figure 3.2

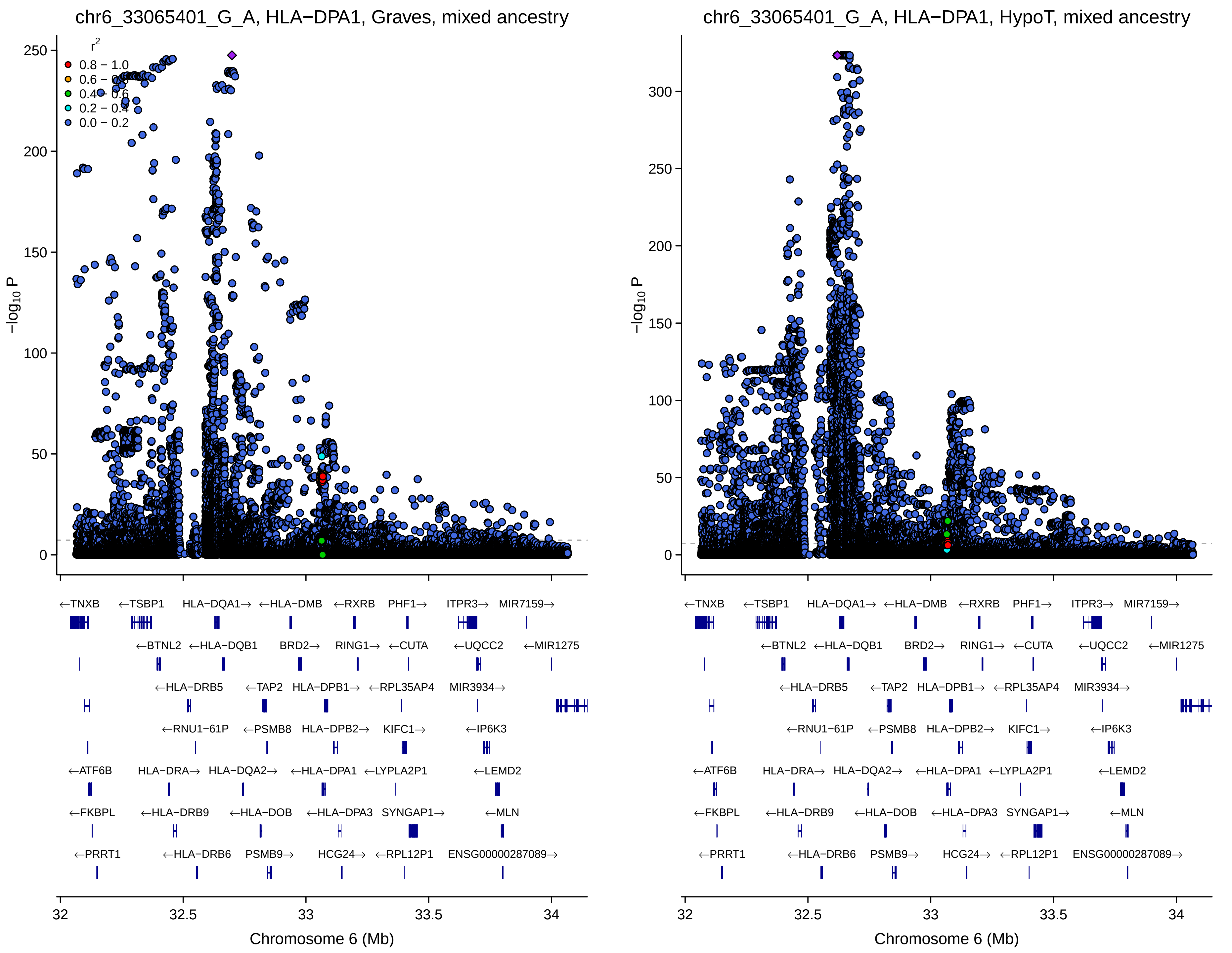

Supplementary Figure 3.2

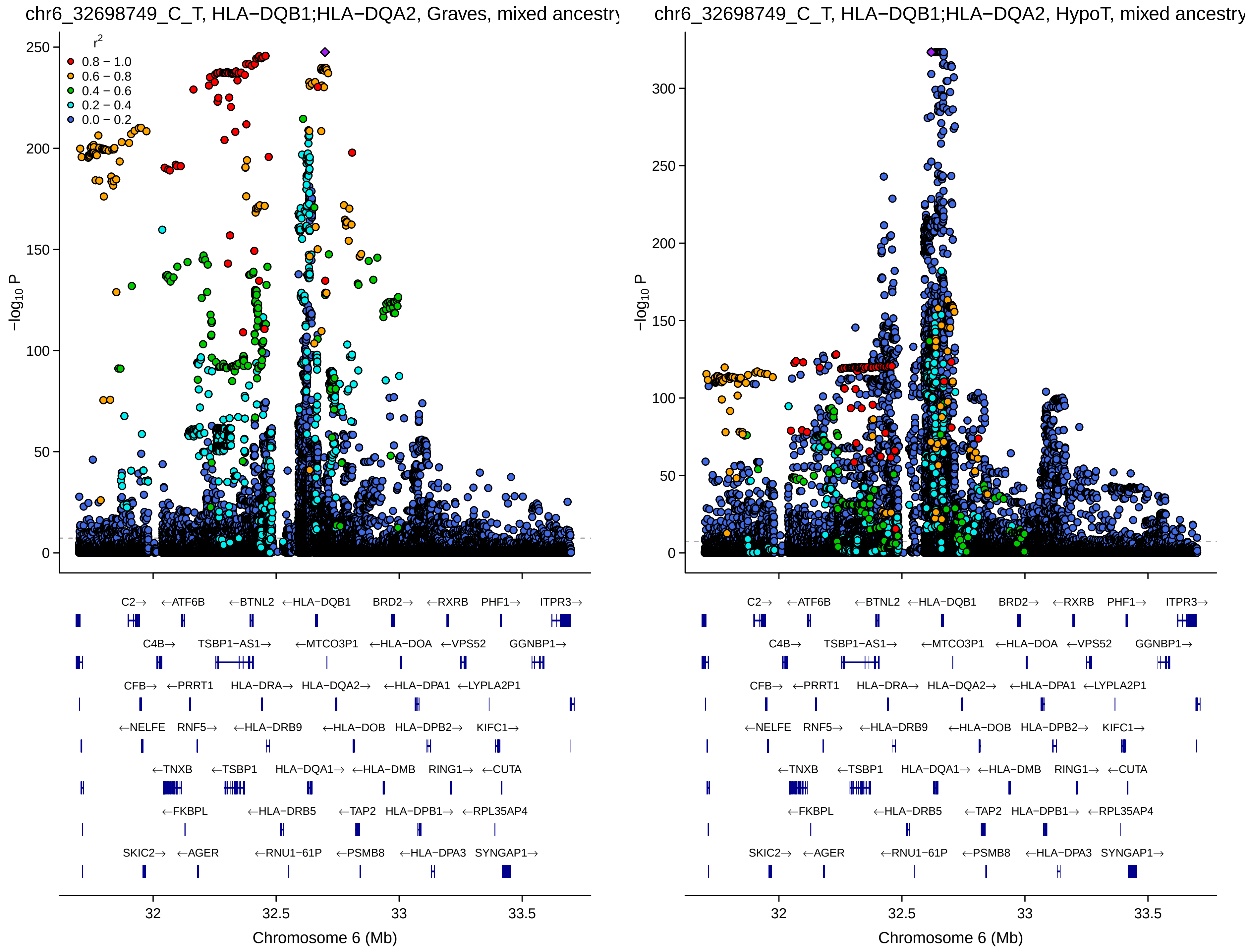

Supplementary Figure 3.2

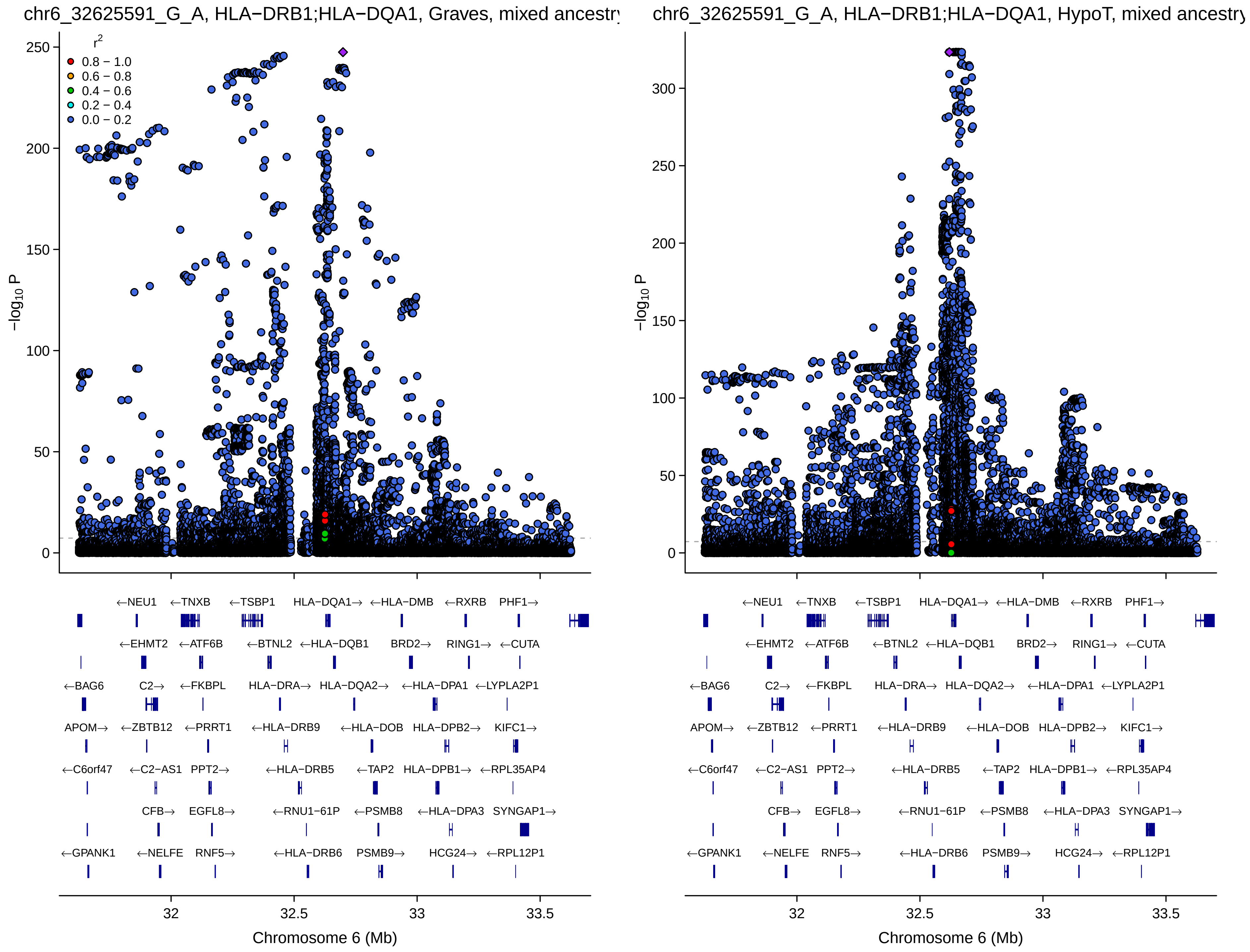

Supplementary Figure 3.2

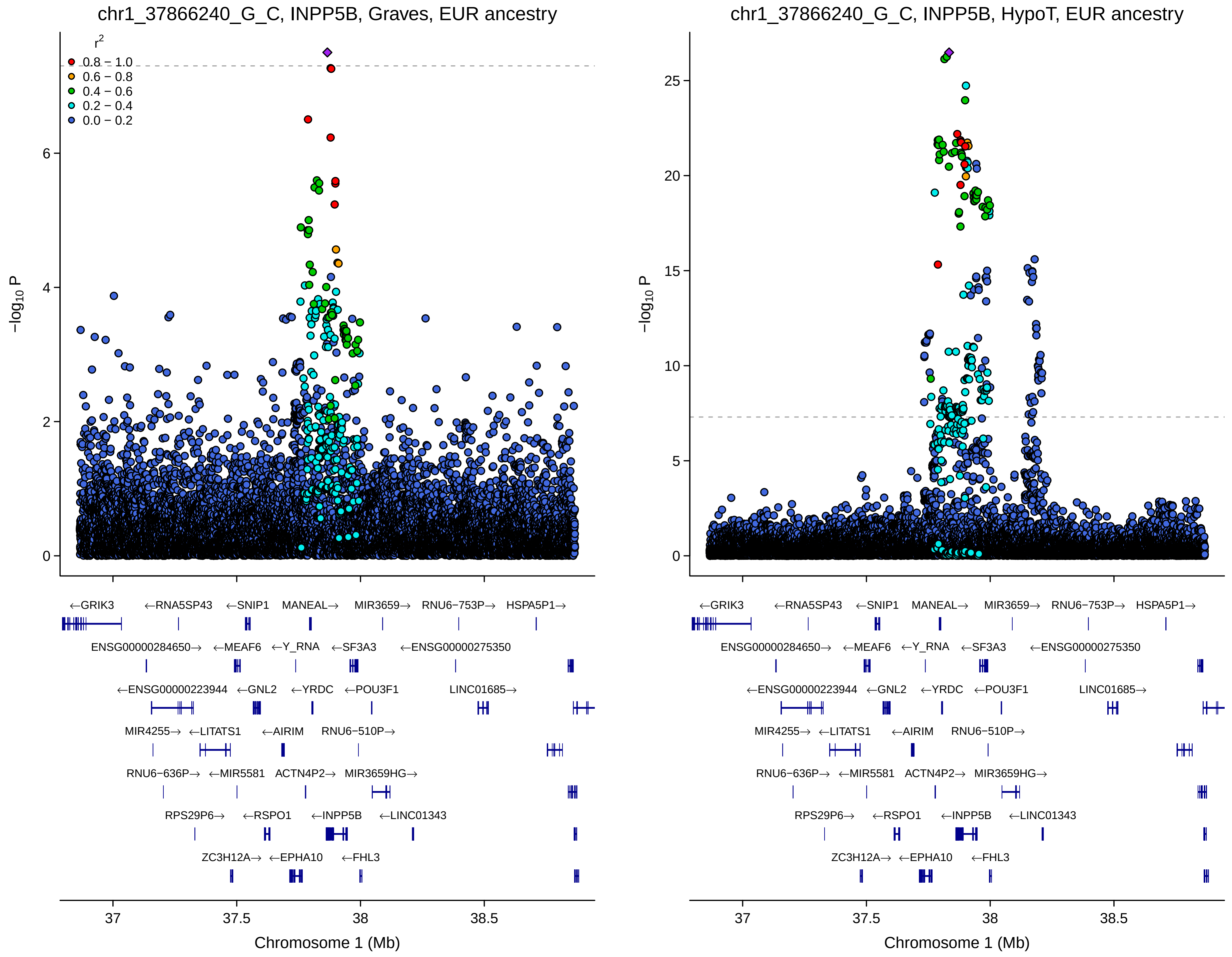

Supplementary Figure 3.2

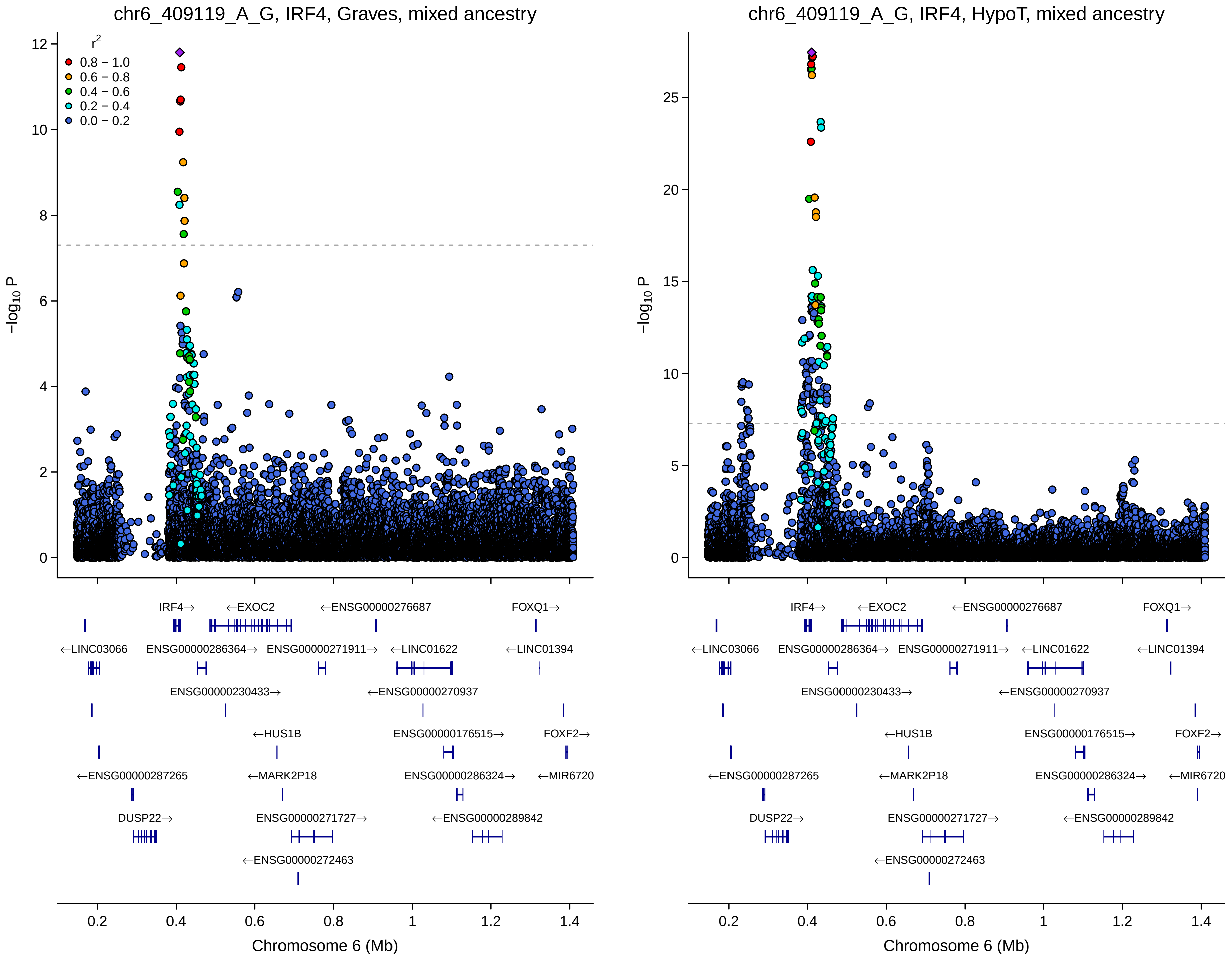

Supplementary Figure 3.2

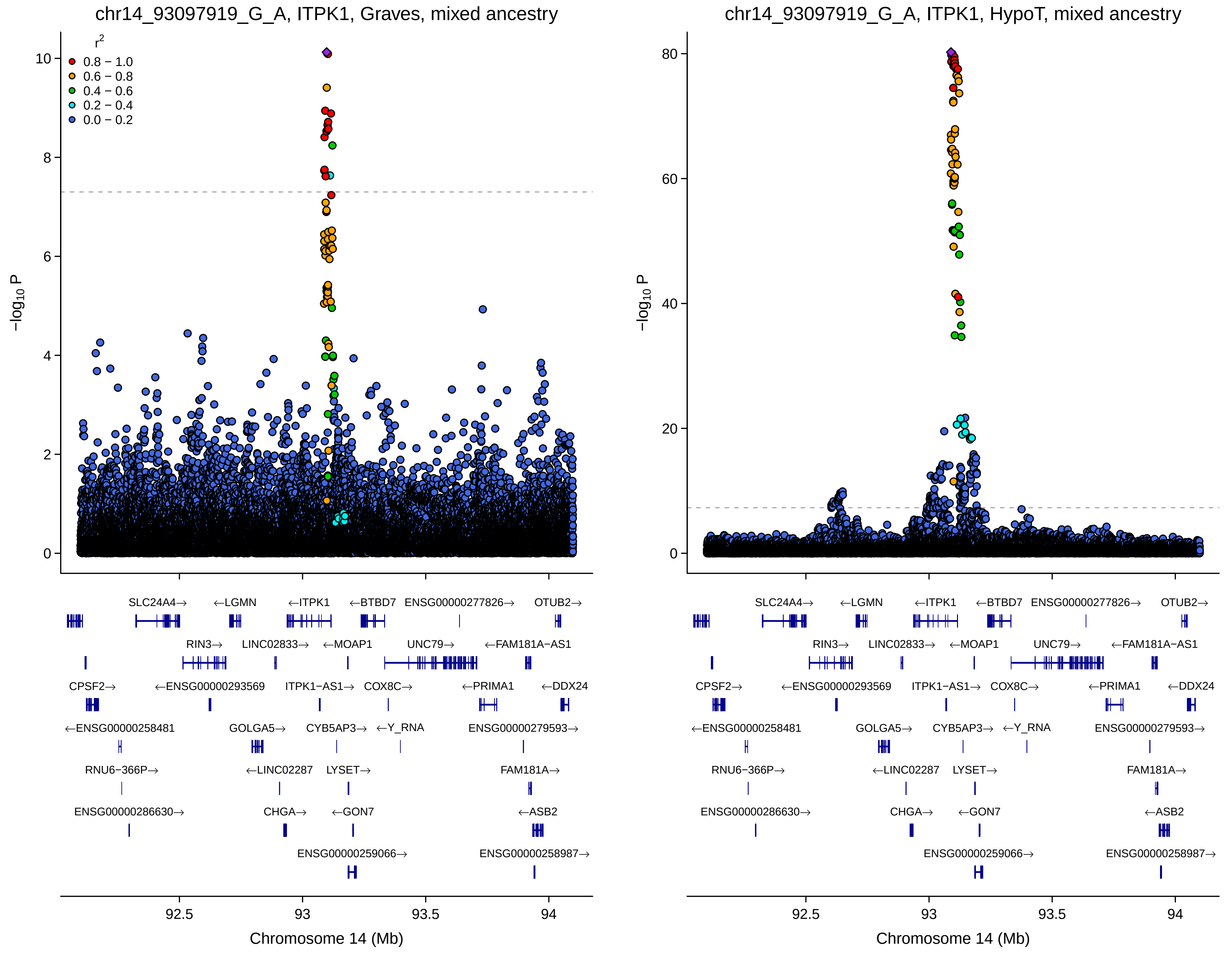

Supplementary Figure 3.2

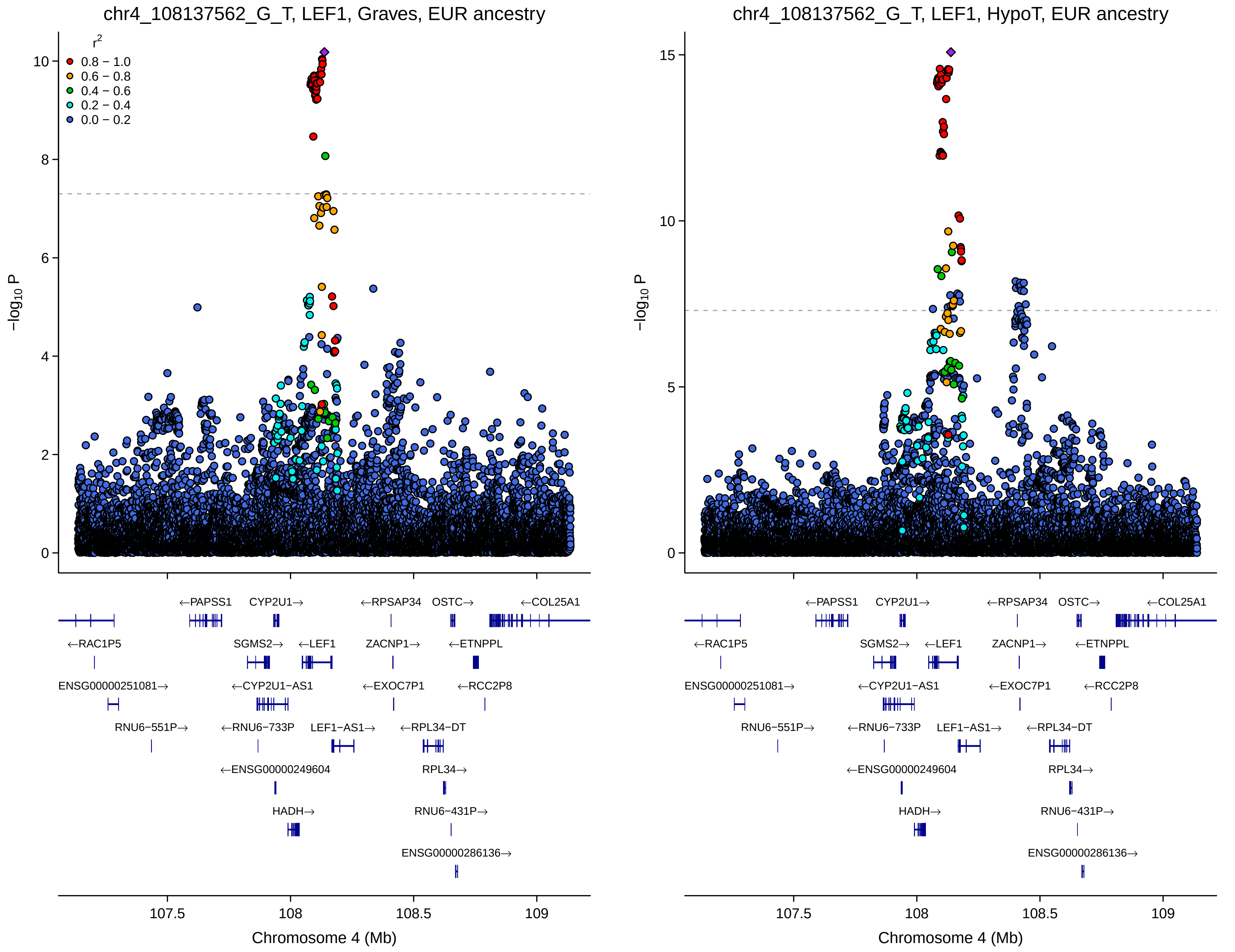

Supplementary Figure 3.2

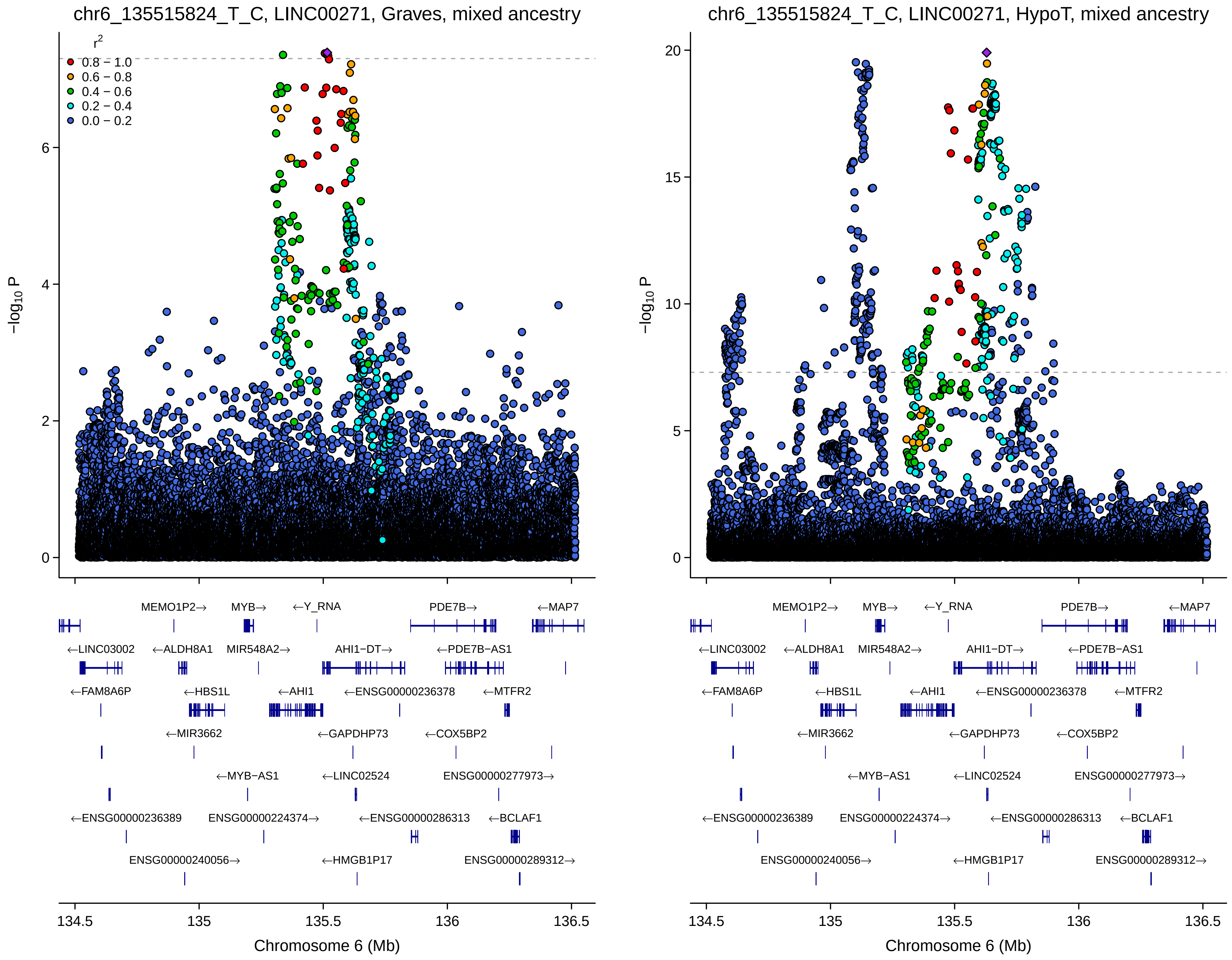

Supplementary Figure 3.2

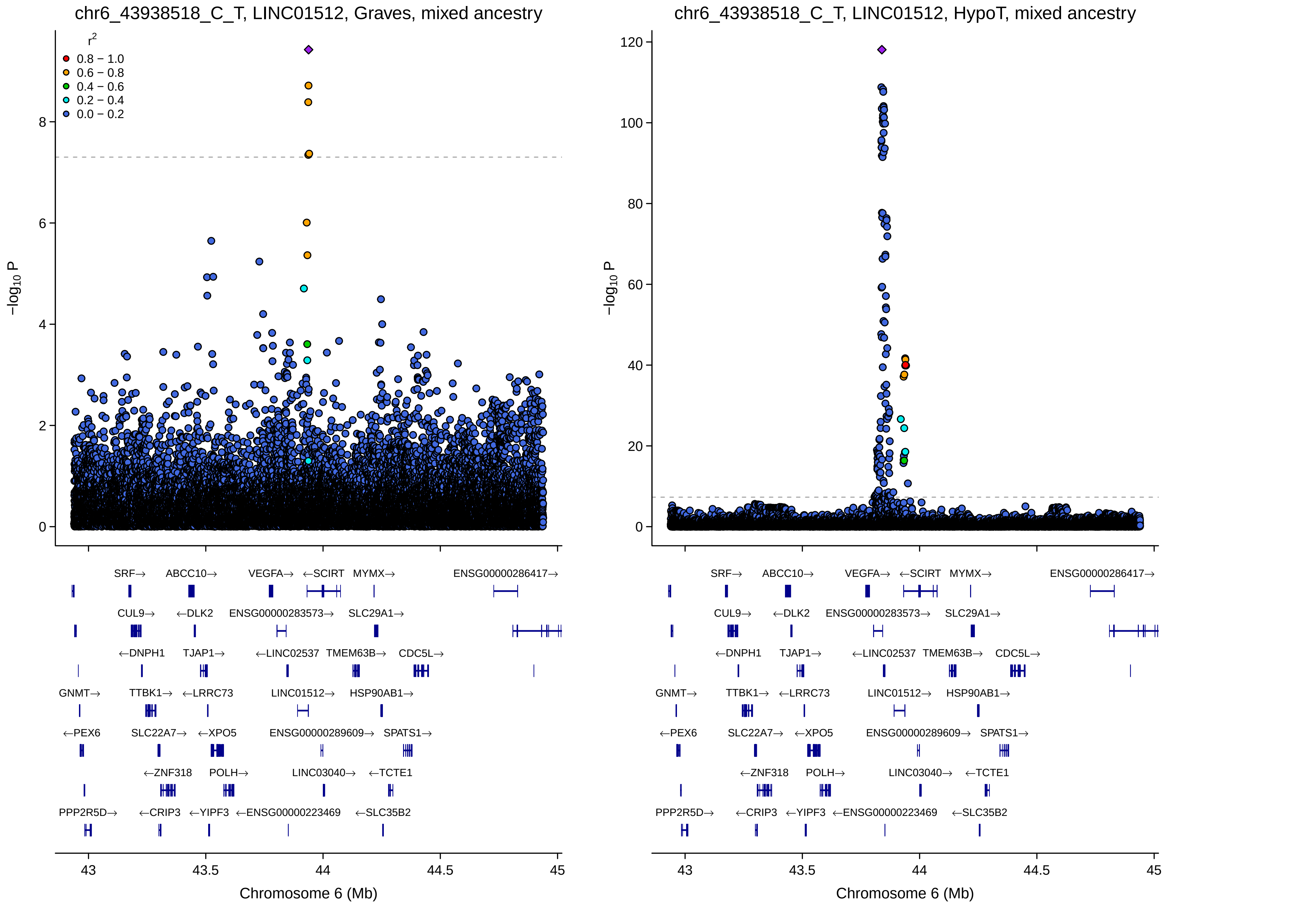

Supplementary Figure 3.2

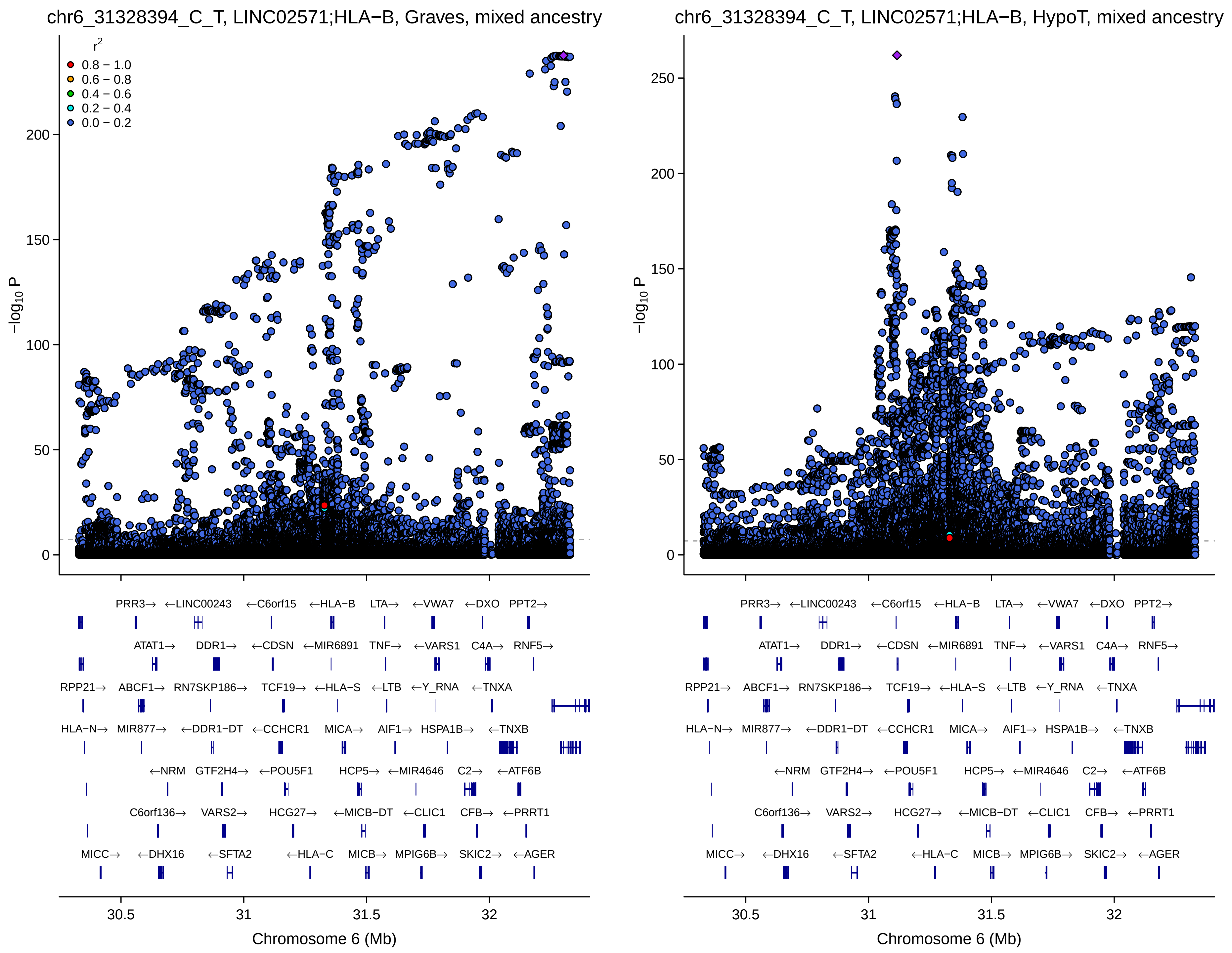

Supplementary Figure 3.2

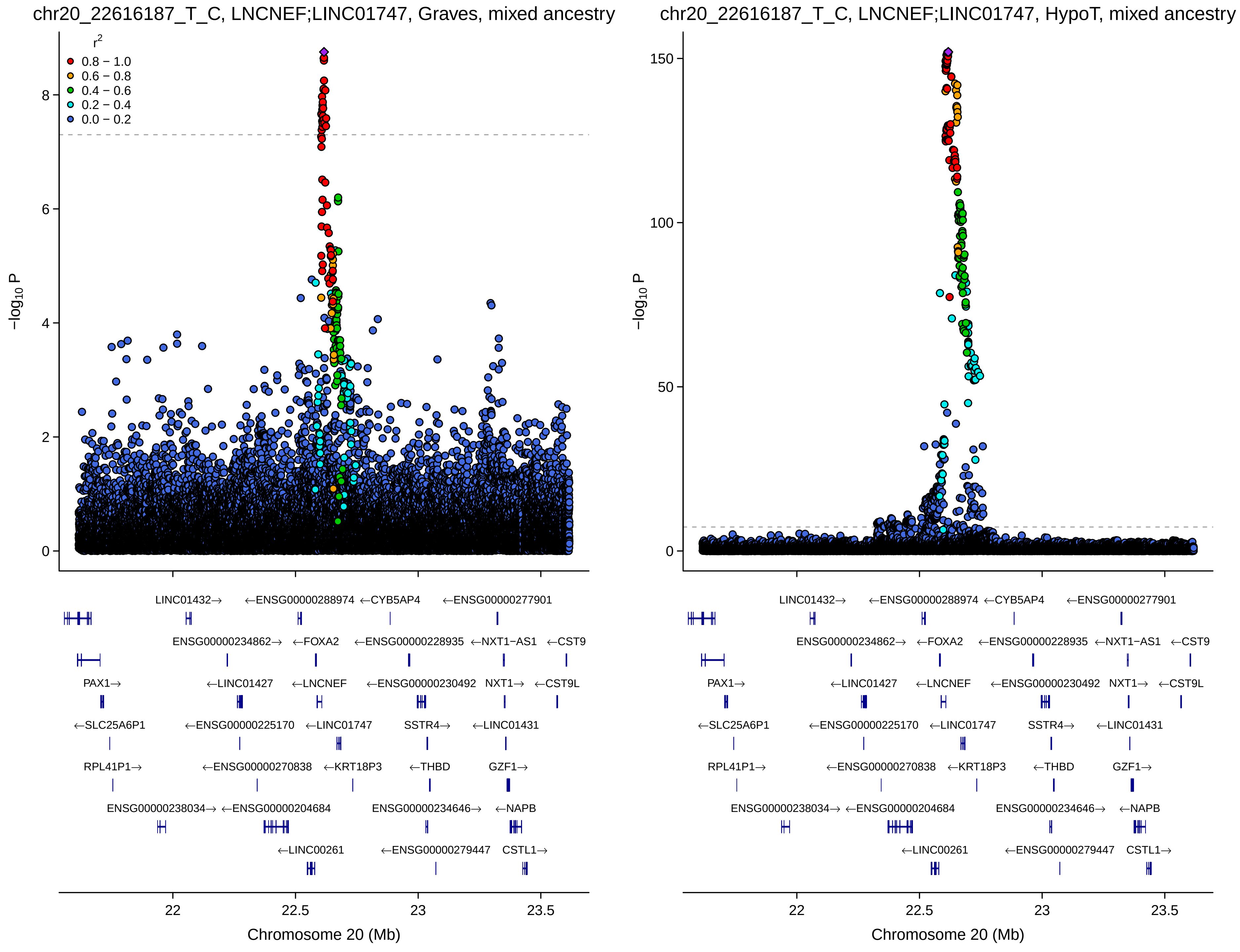

Supplementary Figure 3.2

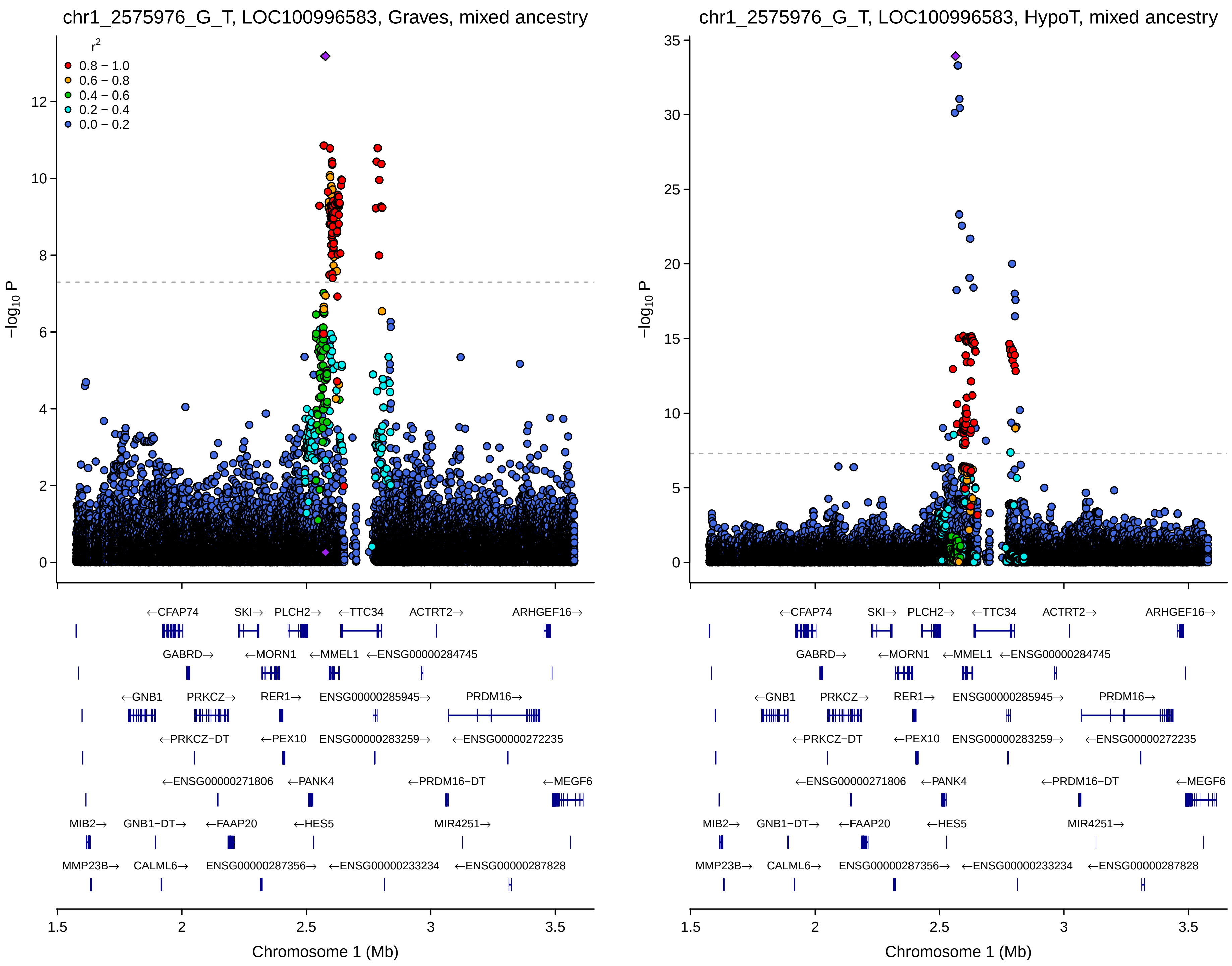

Supplementary Figure 3.2

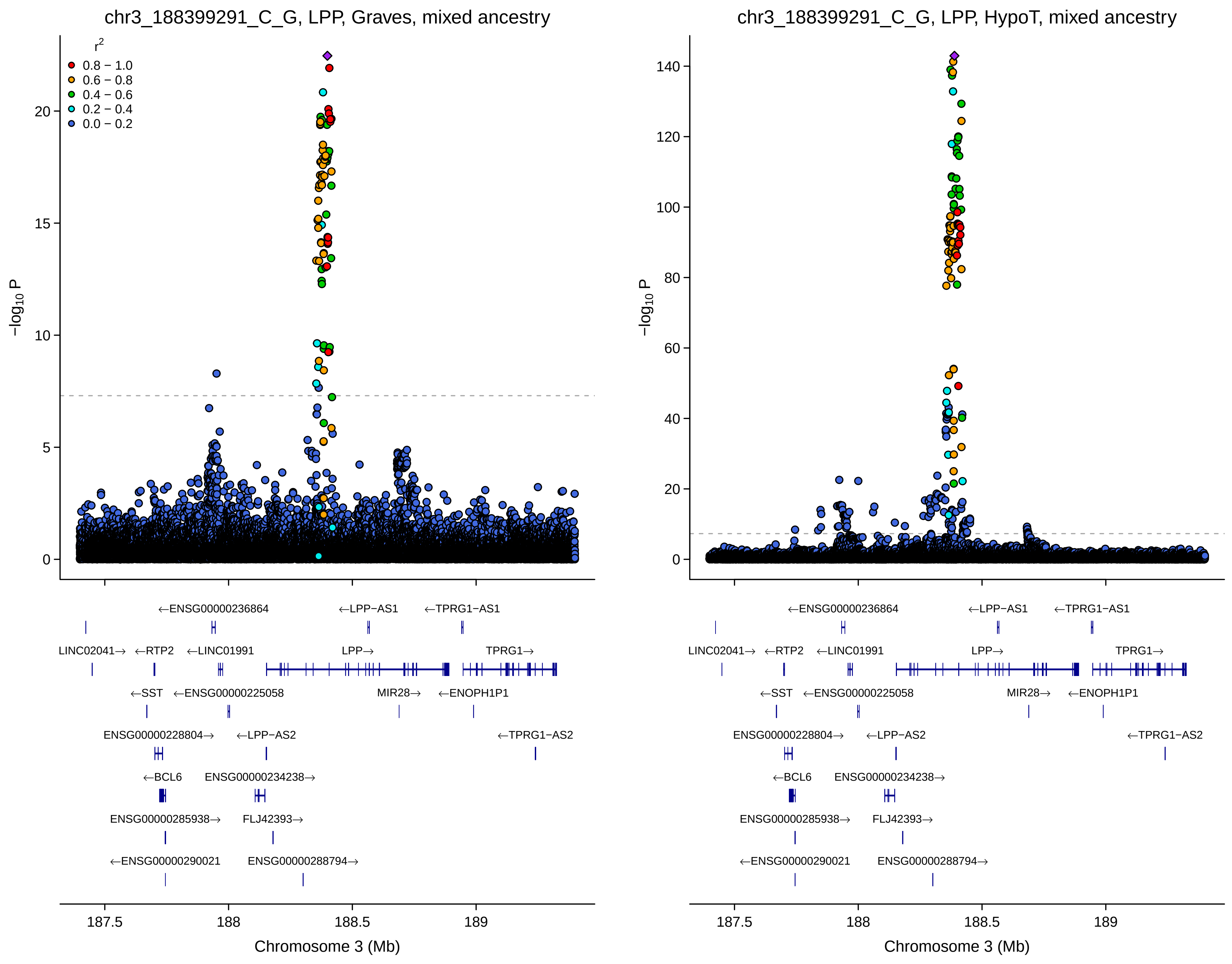

Supplementary Figure 3.2

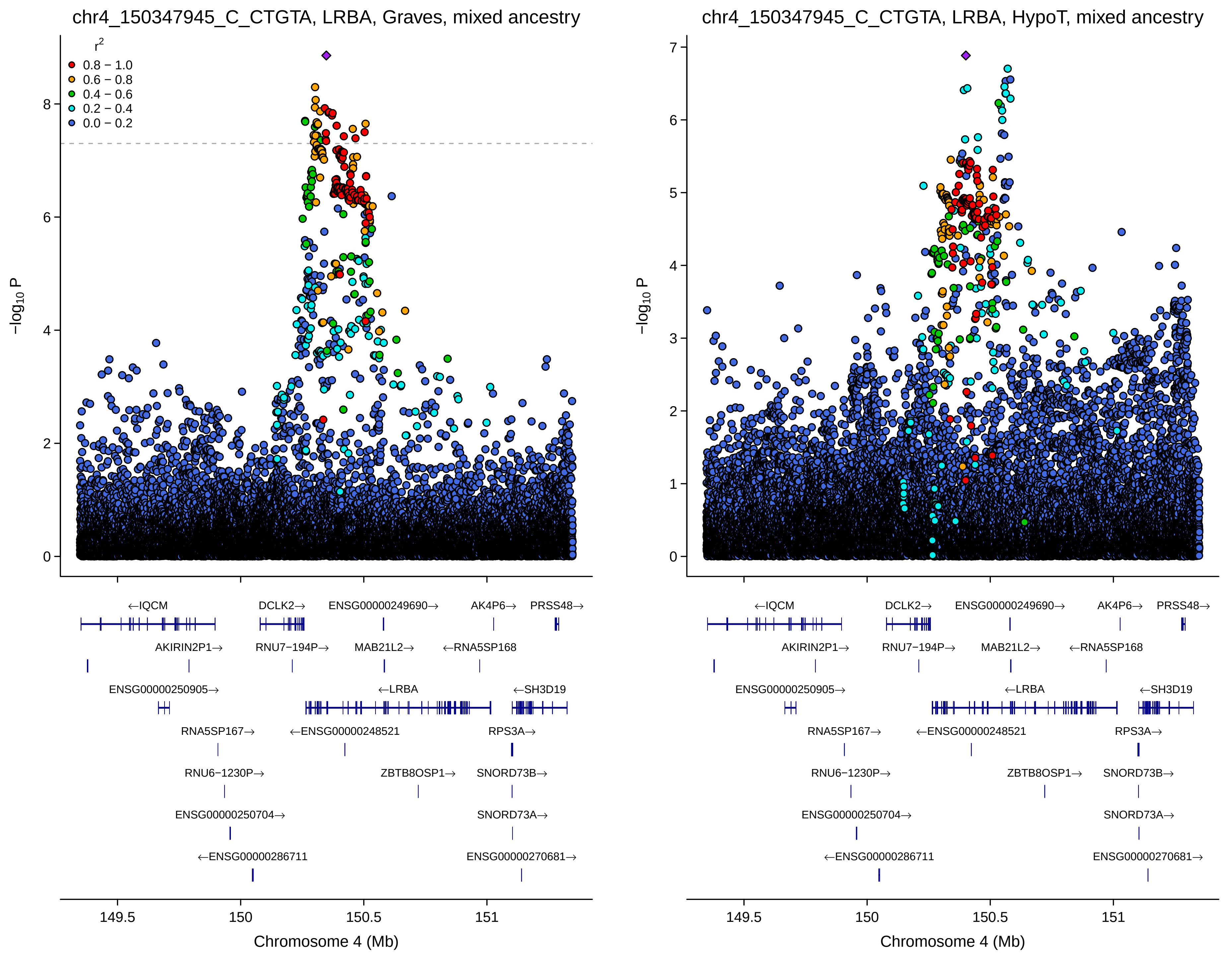

Supplementary Figure 3.2

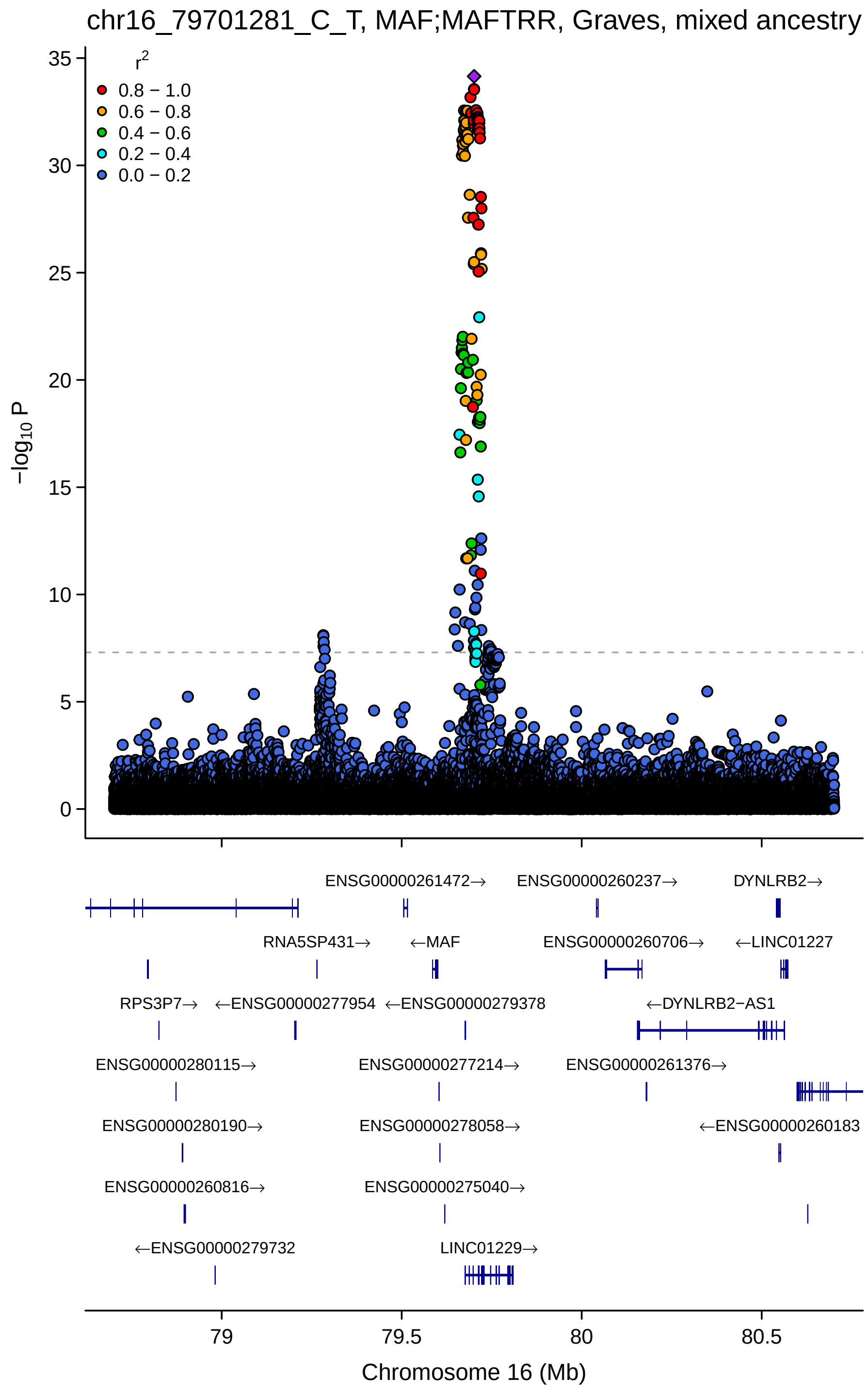

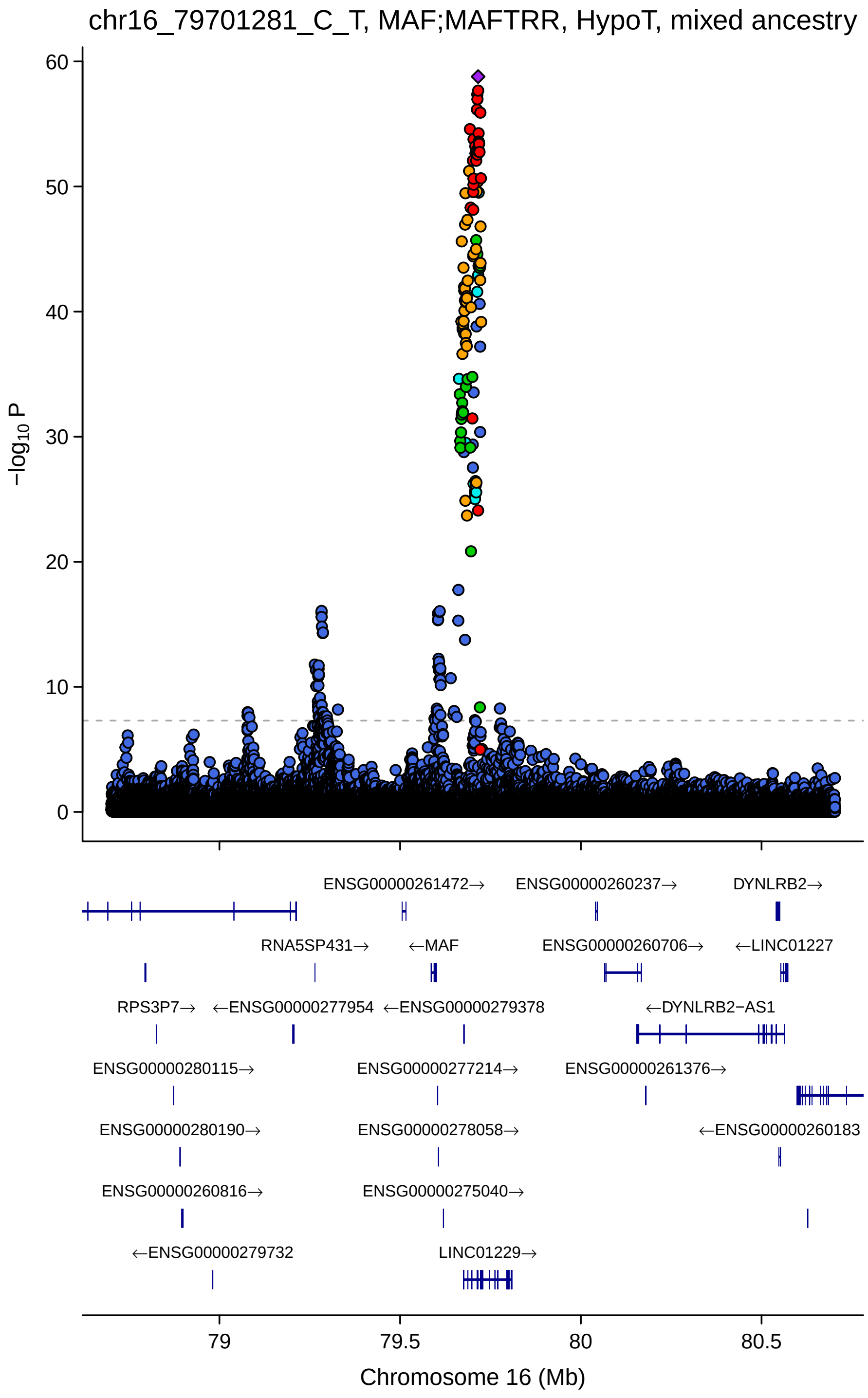

Supplementary Figure 3.2

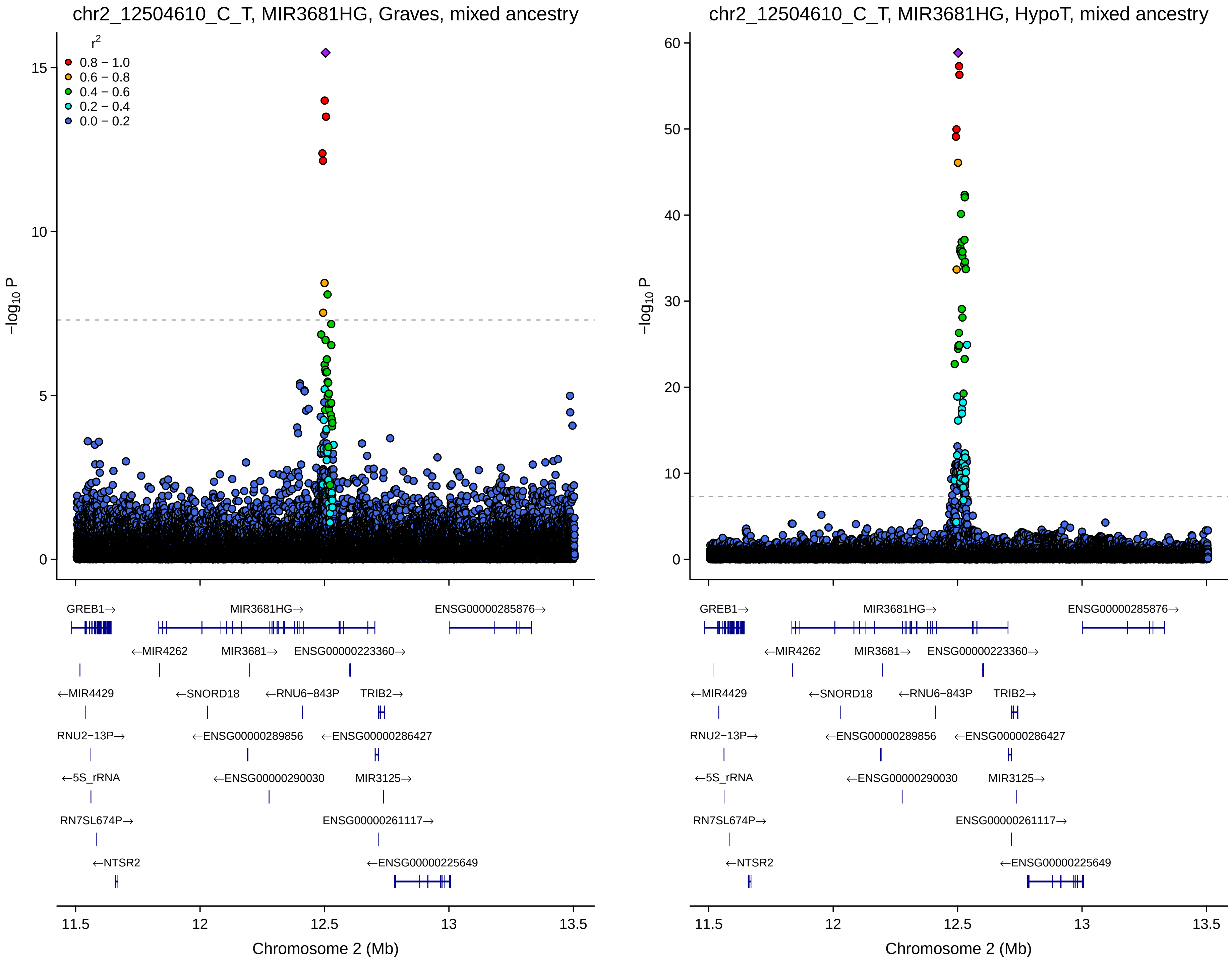

Supplementary Figure 3.2

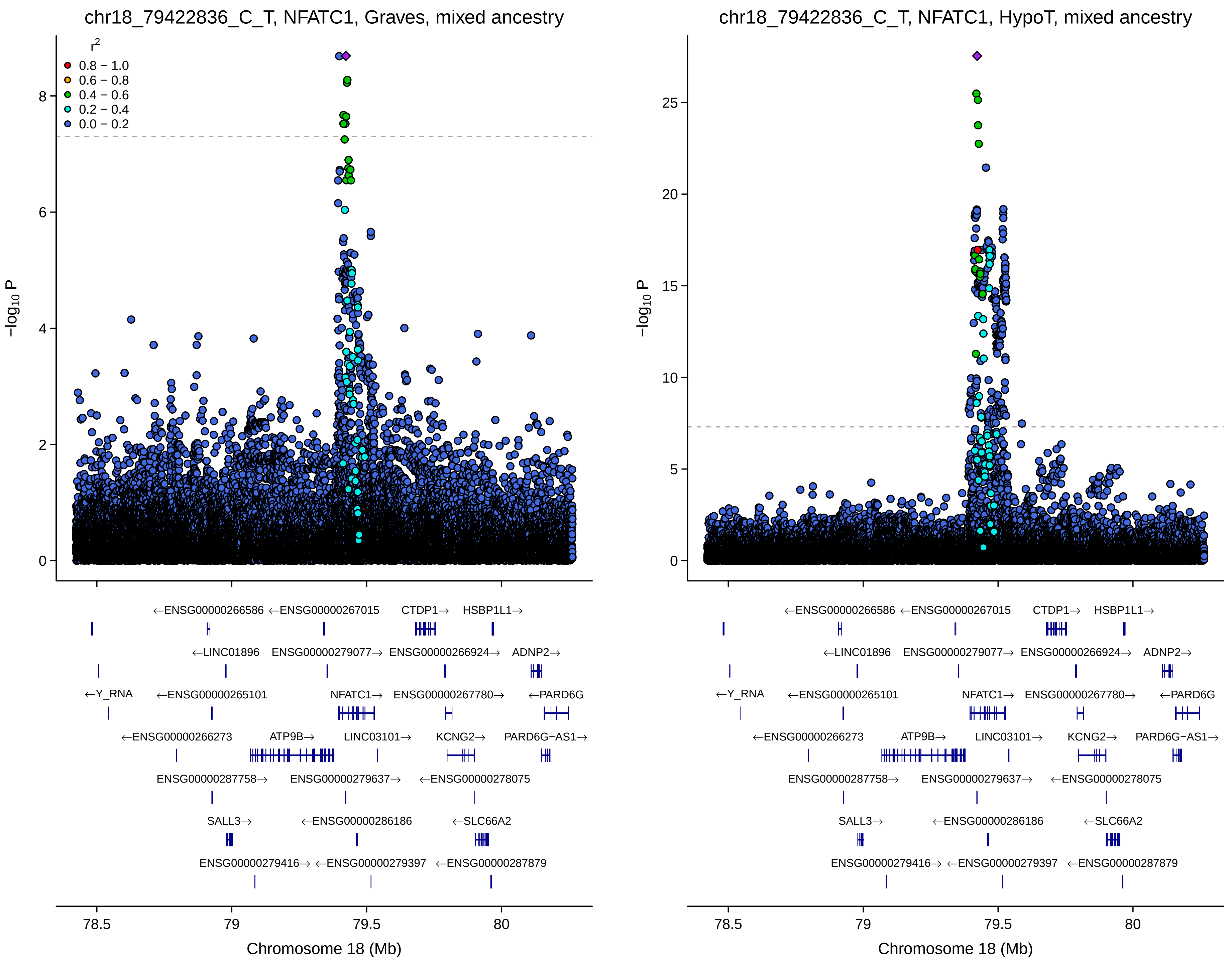

Supplementary Figure 3.2

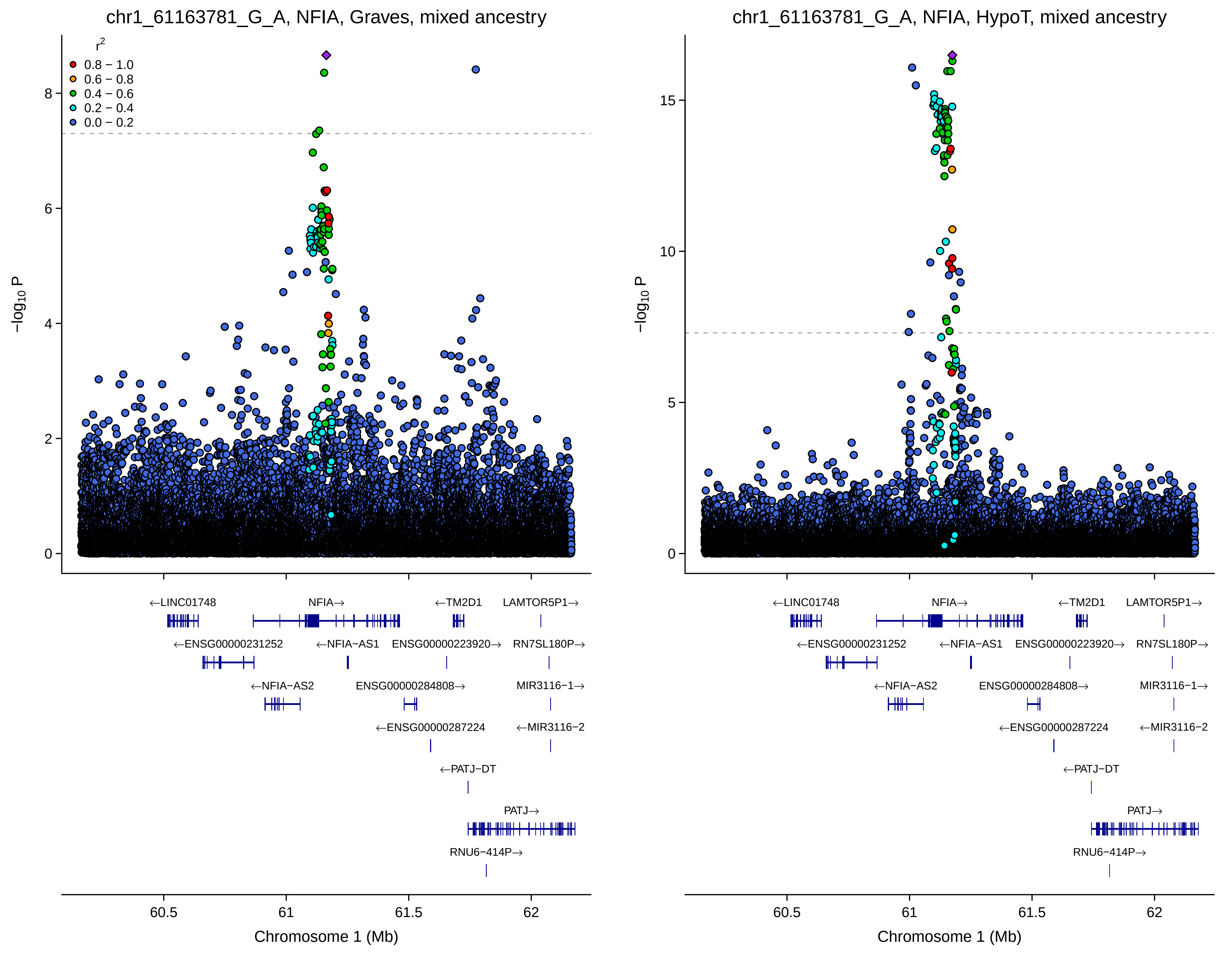

Supplementary Figure 3.2

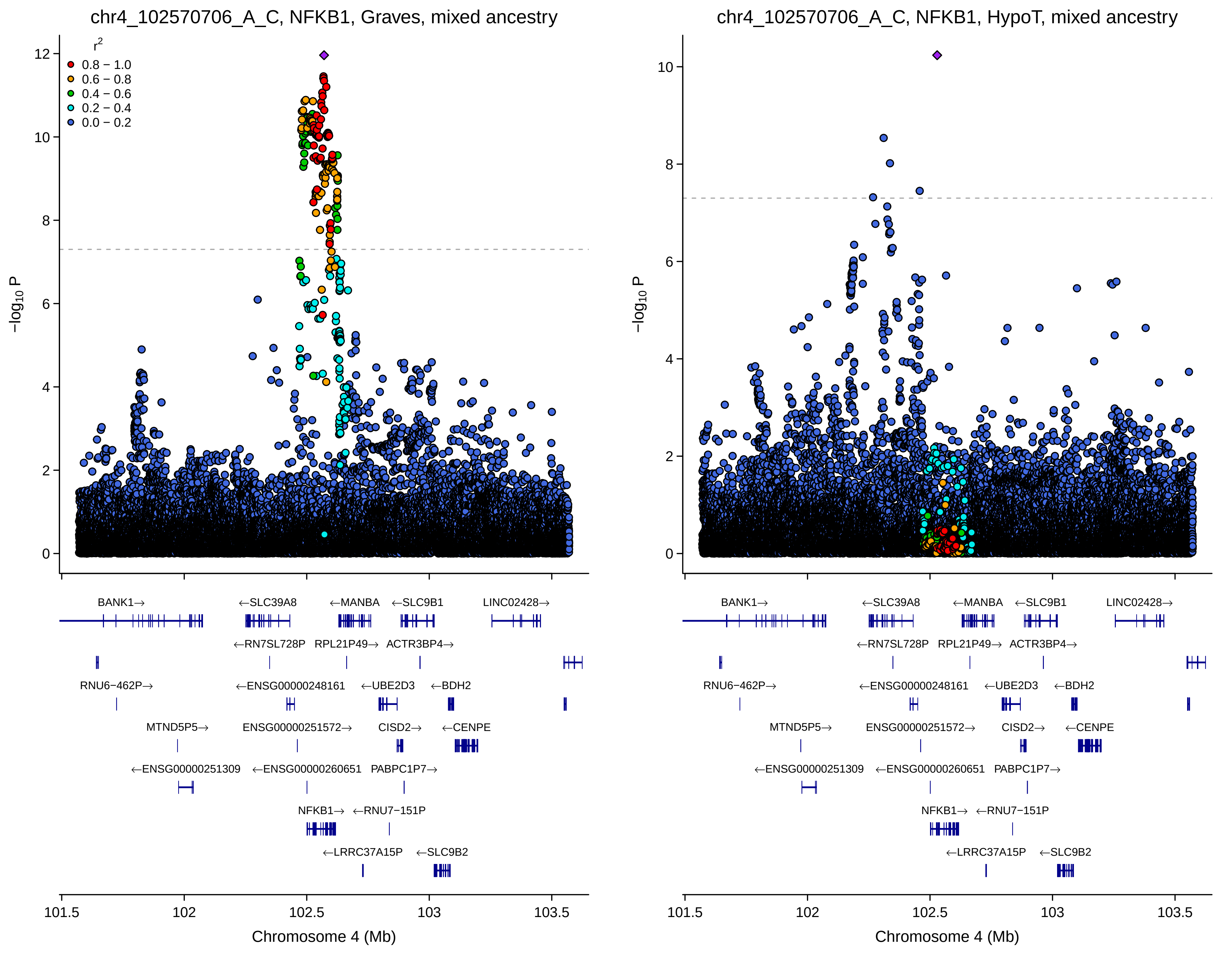

Supplementary Figure 3.2

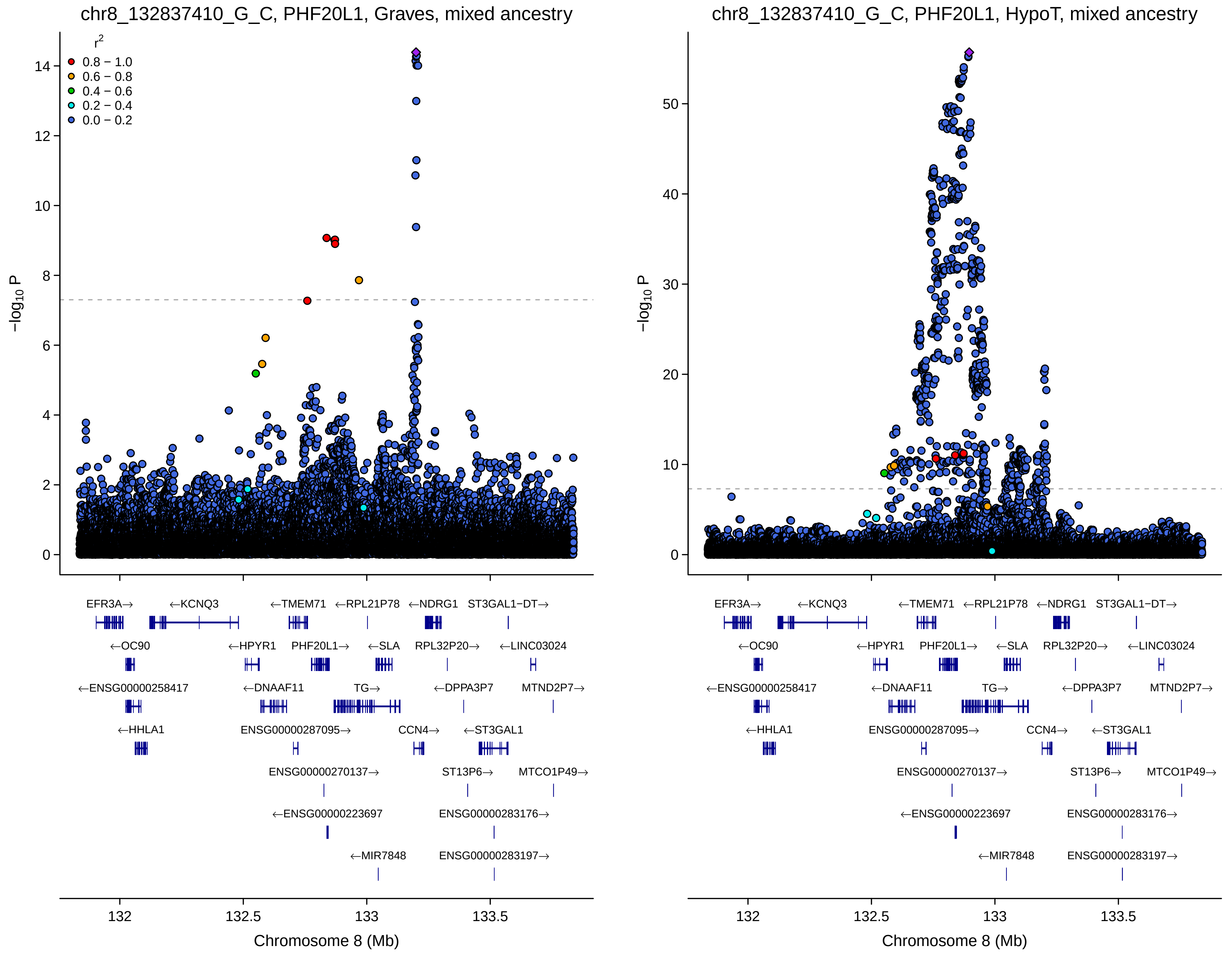

Supplementary Figure 3.2

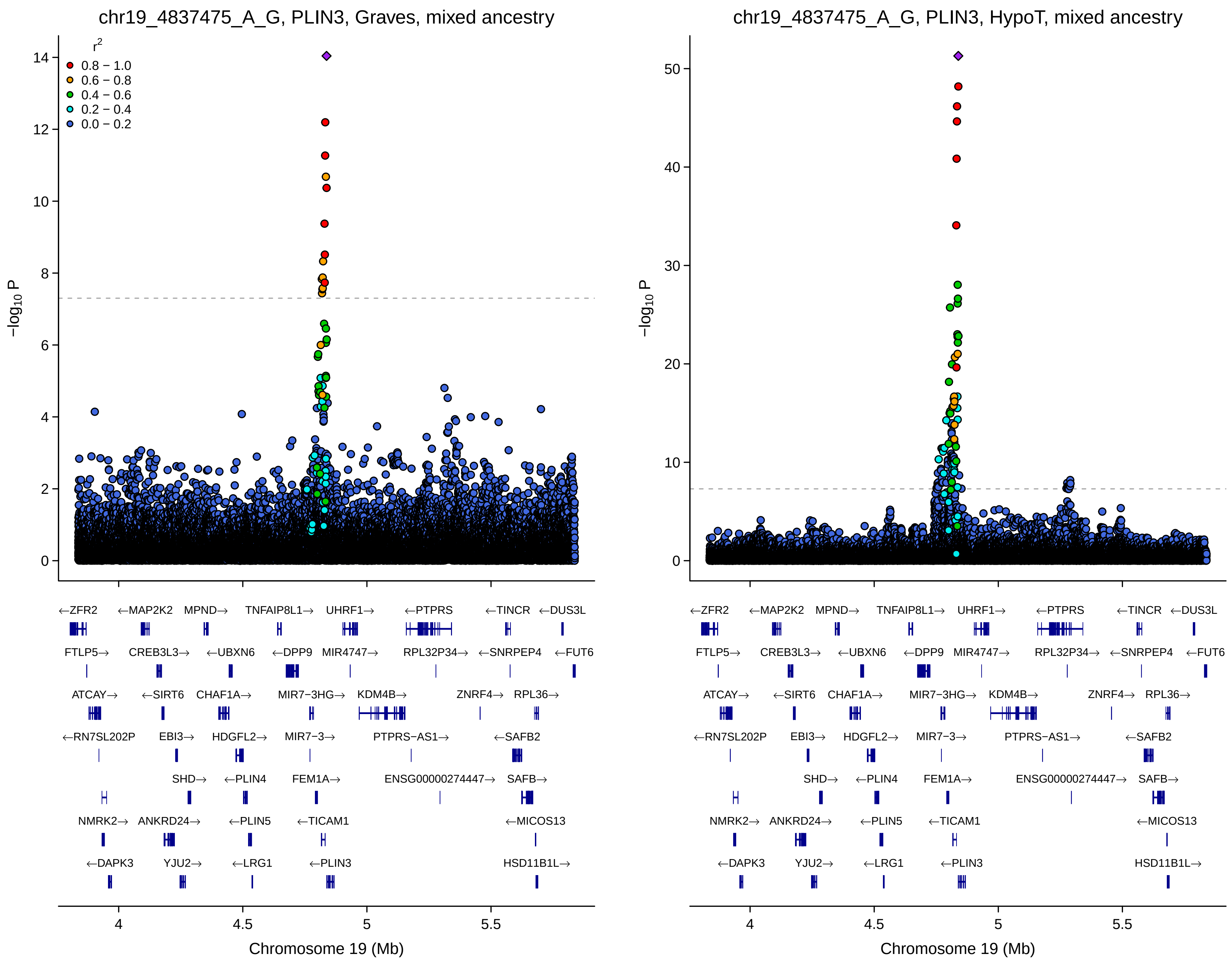

Supplementary Figure 3.2

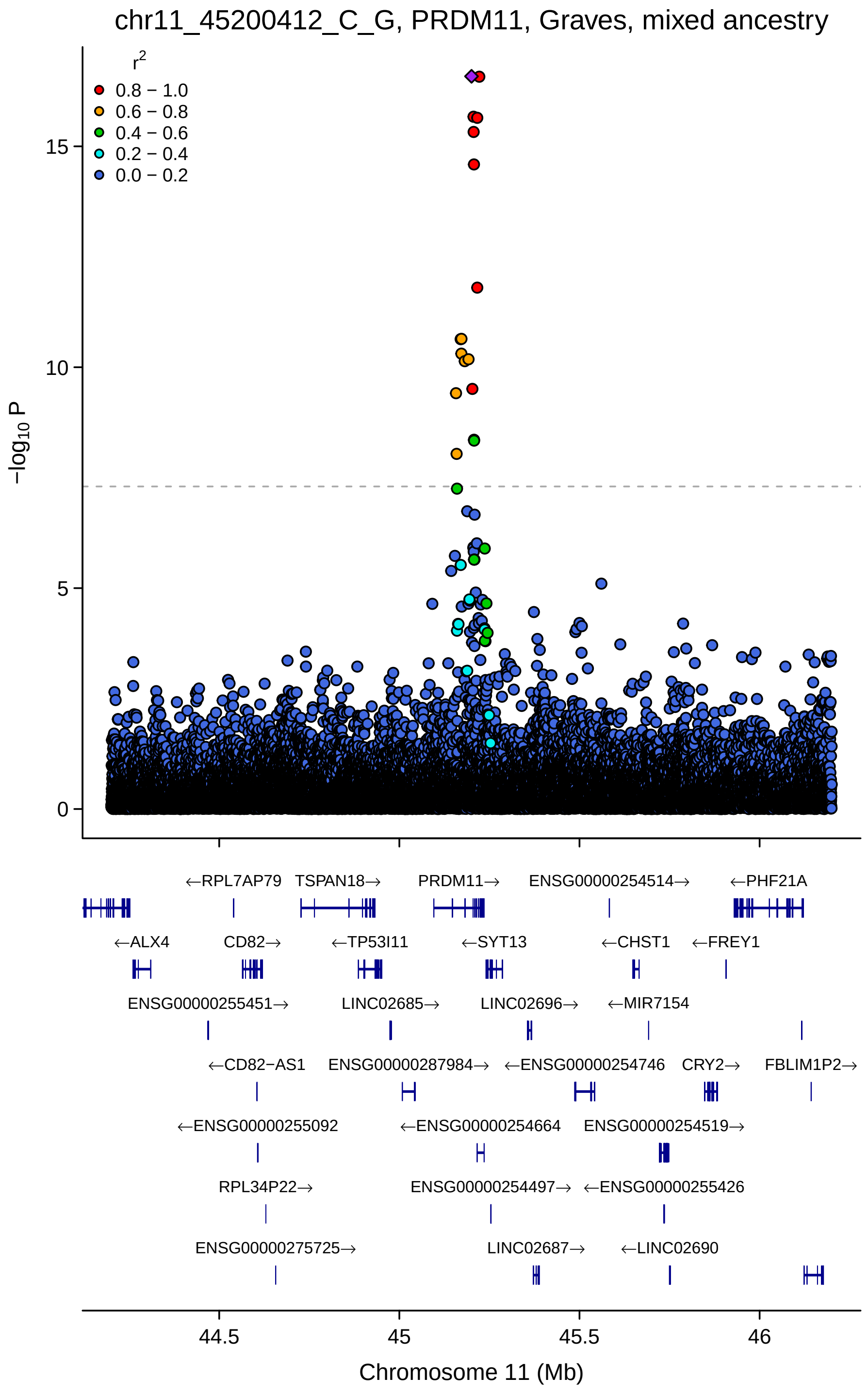

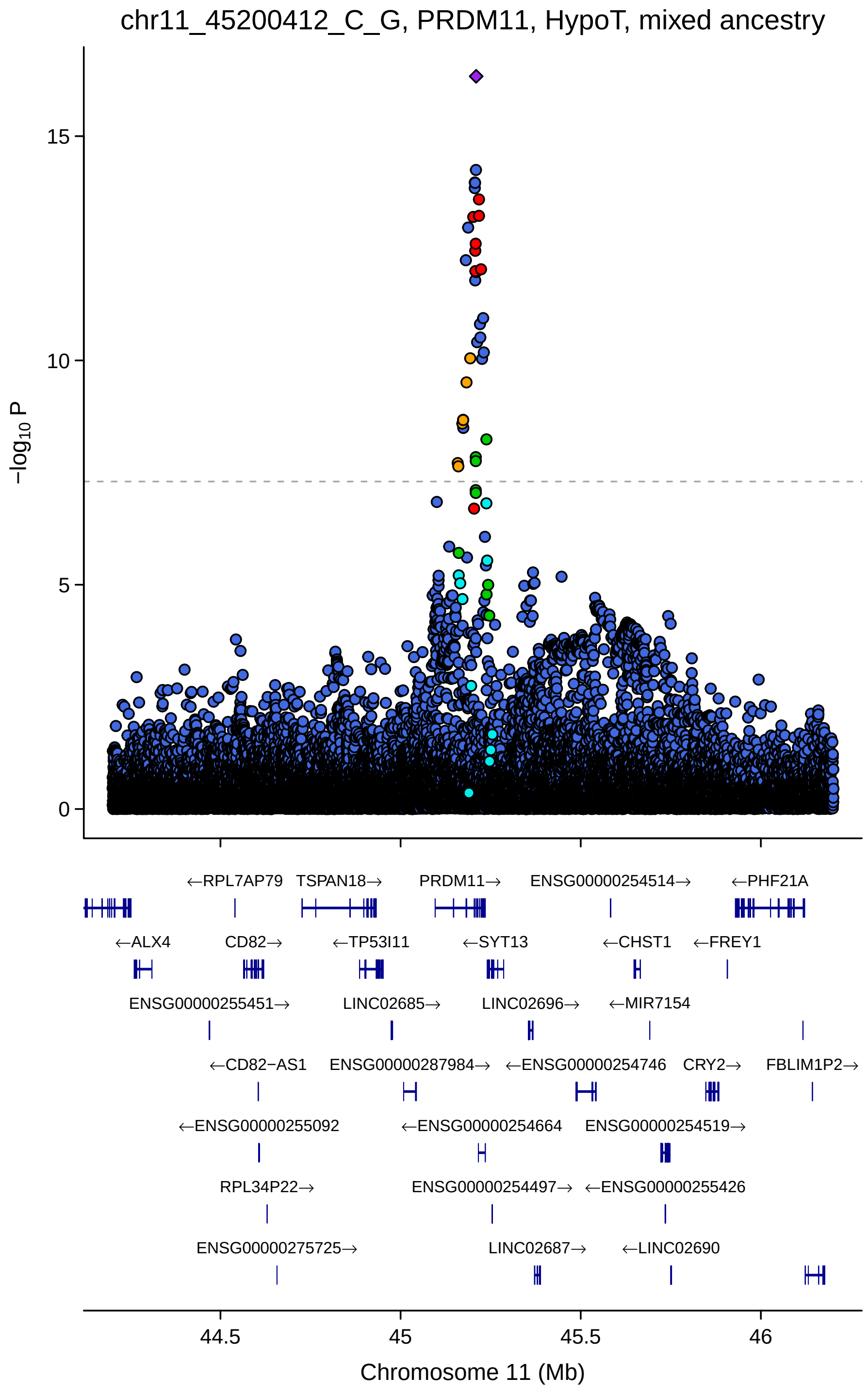

Supplementary Figure 3.2

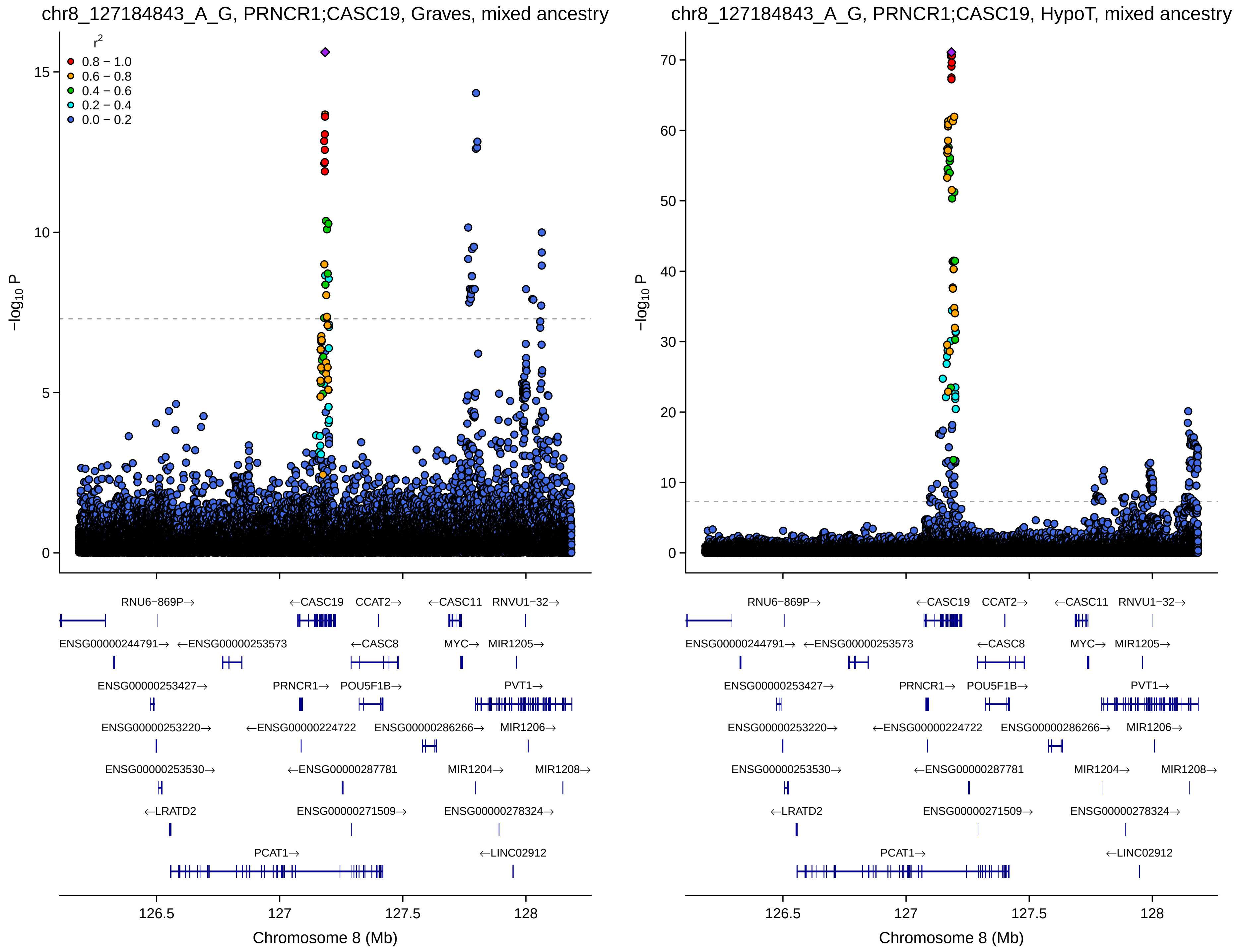

Supplementary Figure 3.2

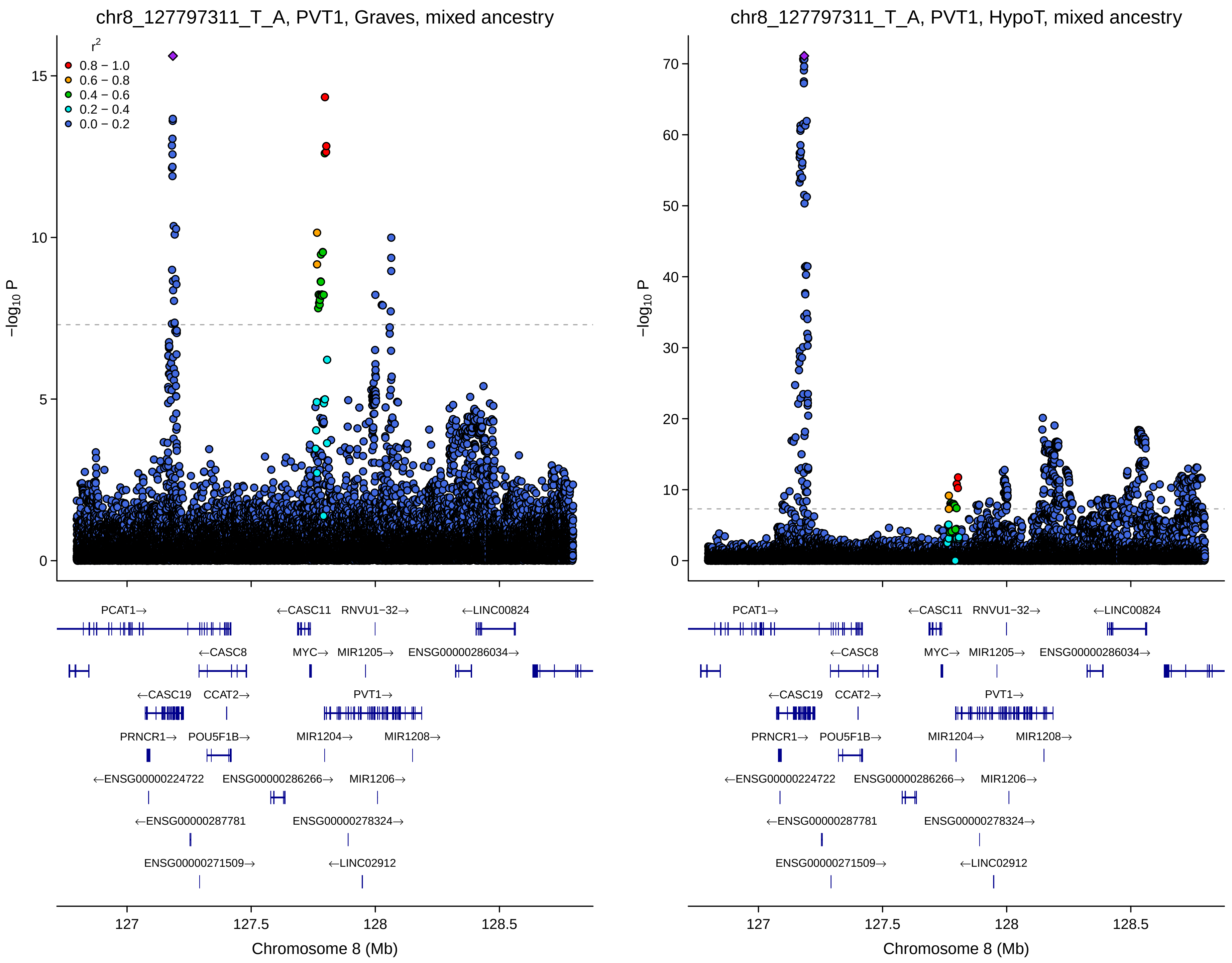

Supplementary Figure 3.2

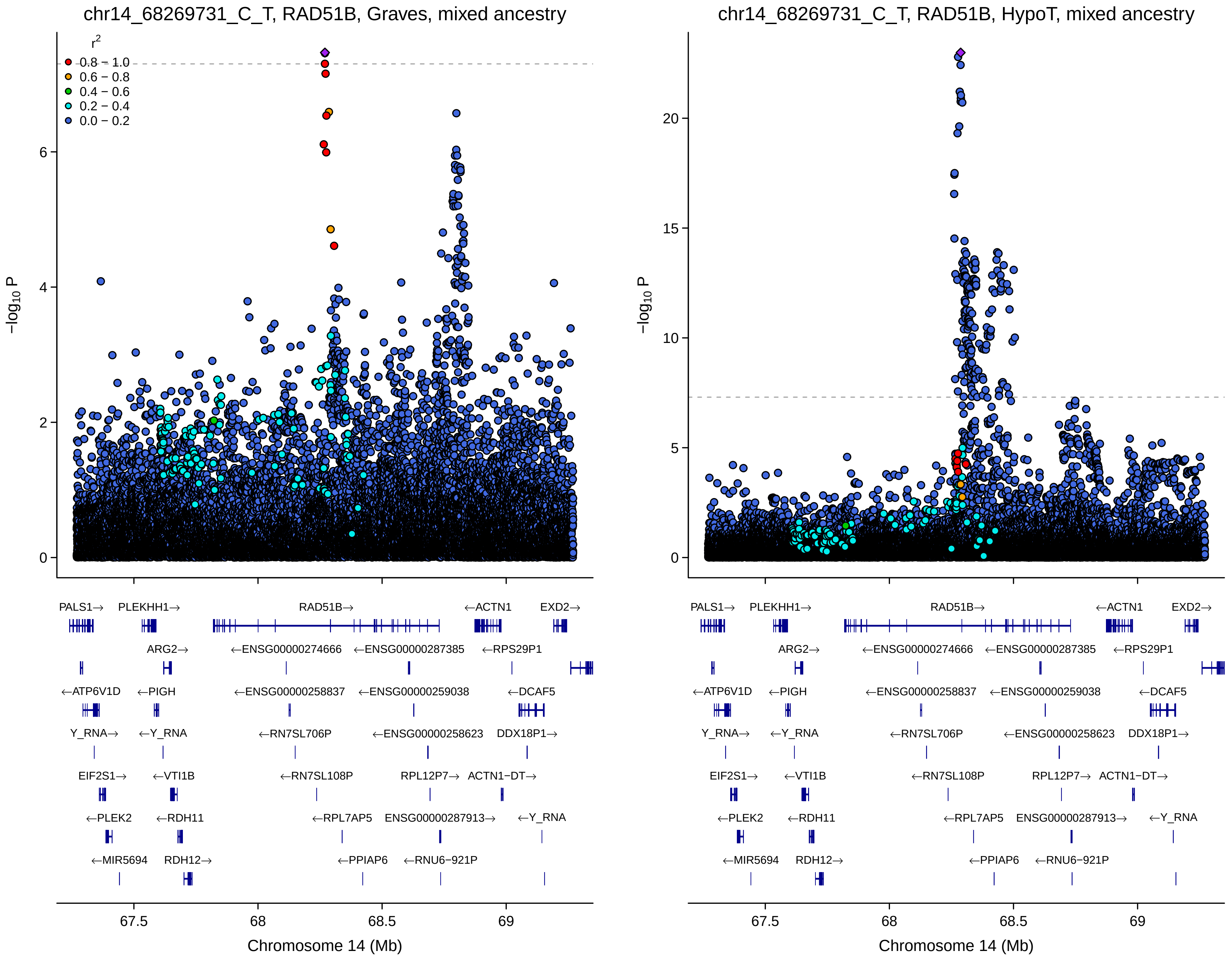

Supplementary Figure 3.2

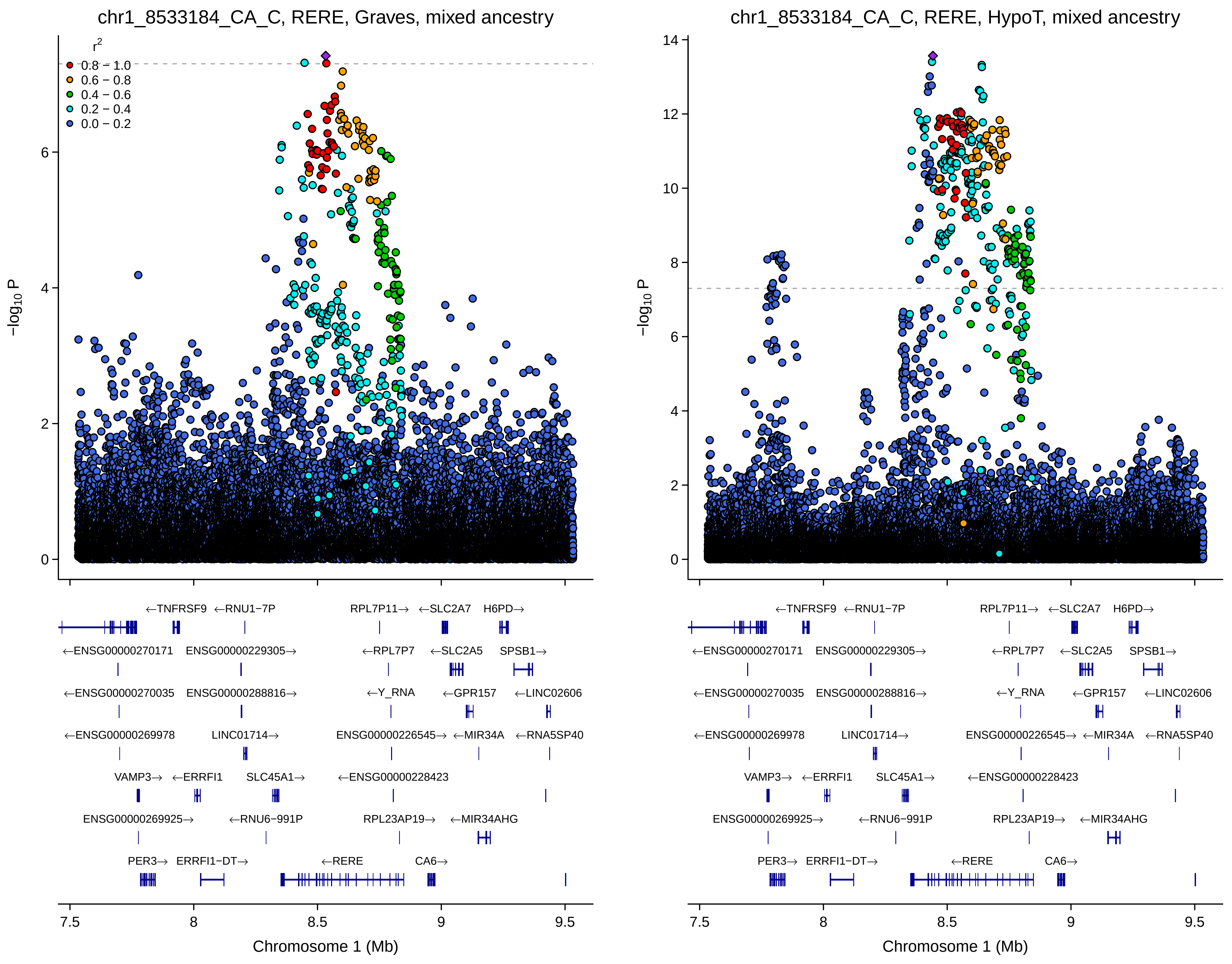

Supplementary Figure 3.2

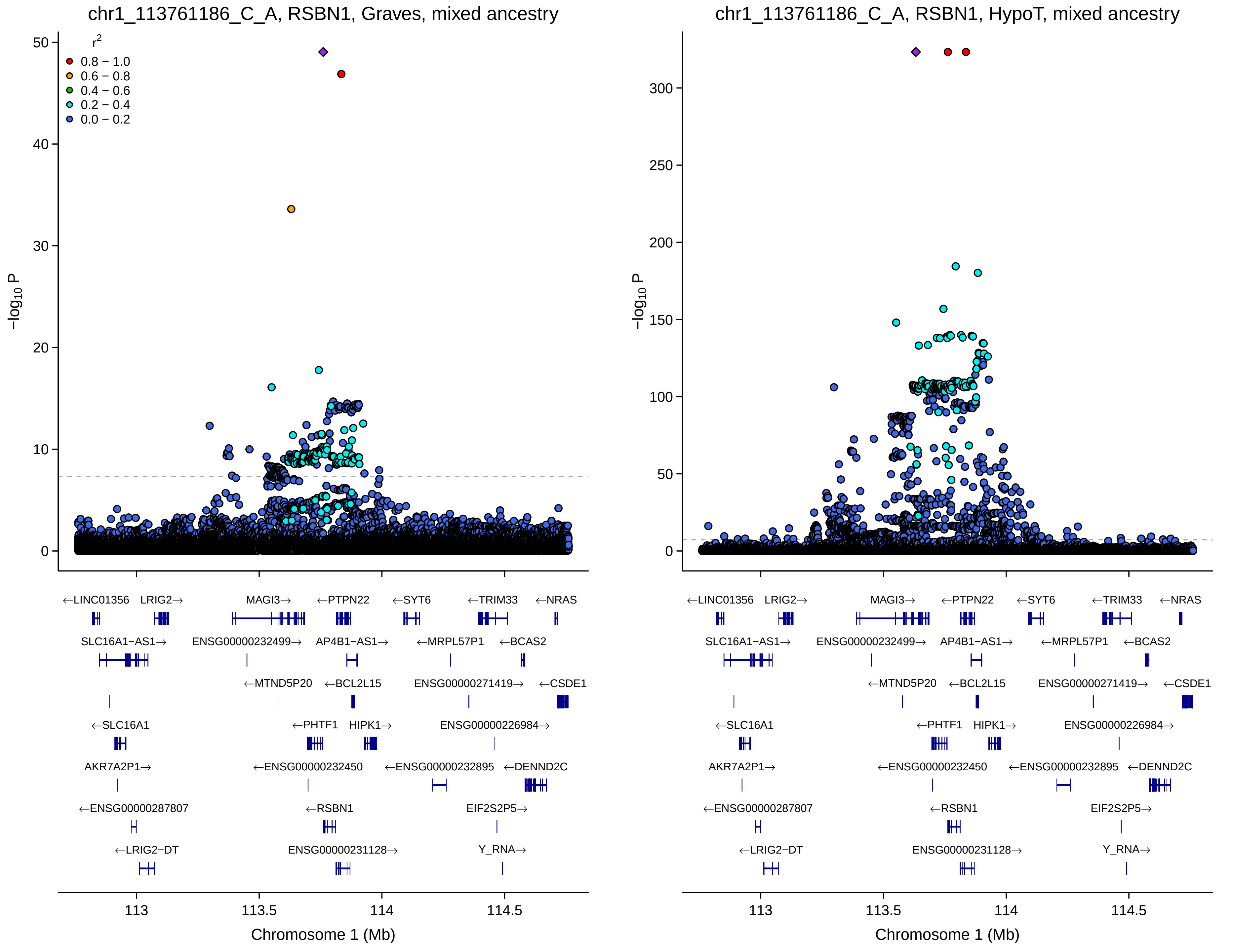

Supplementary Figure 3.2

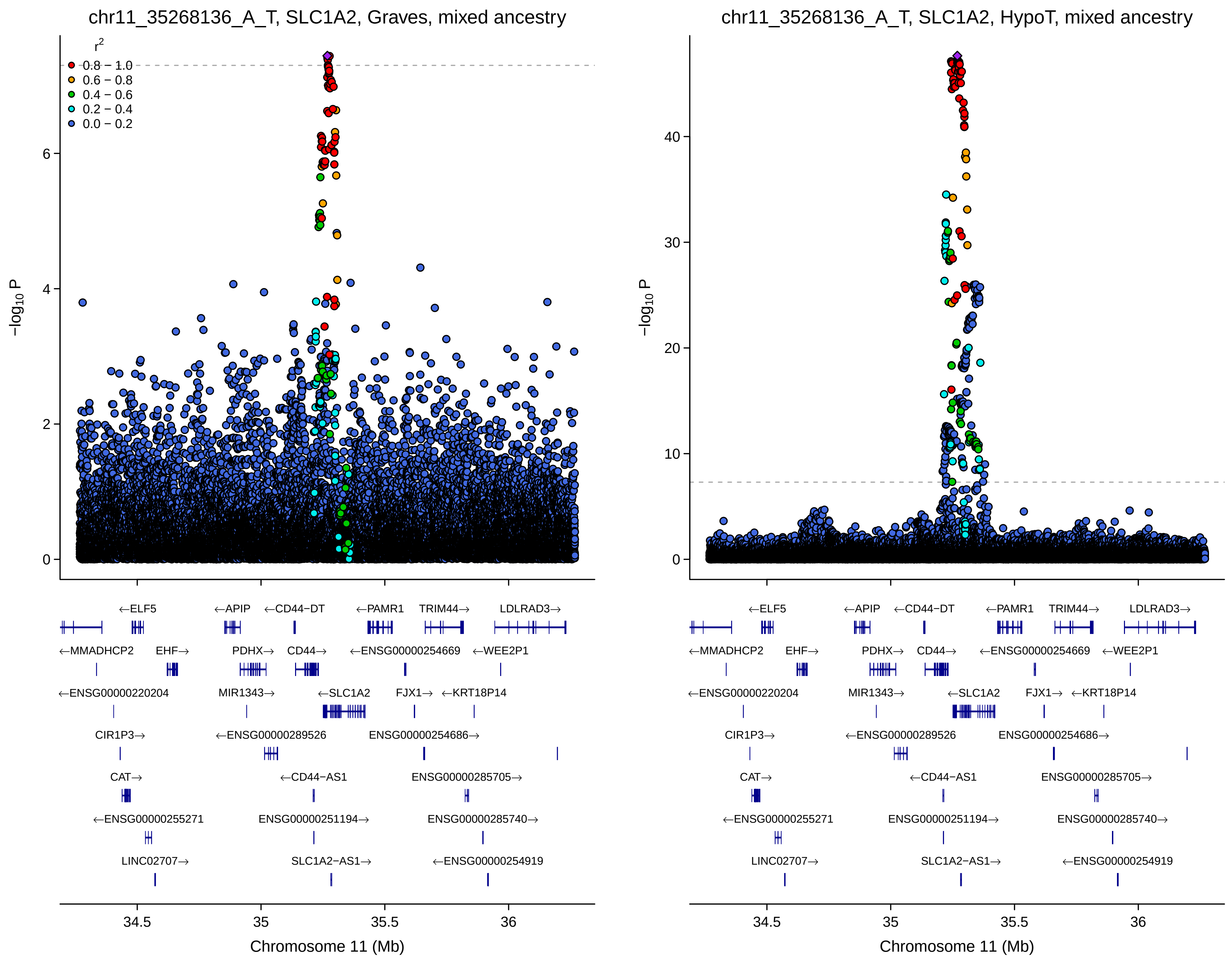

Supplementary Figure 3.2

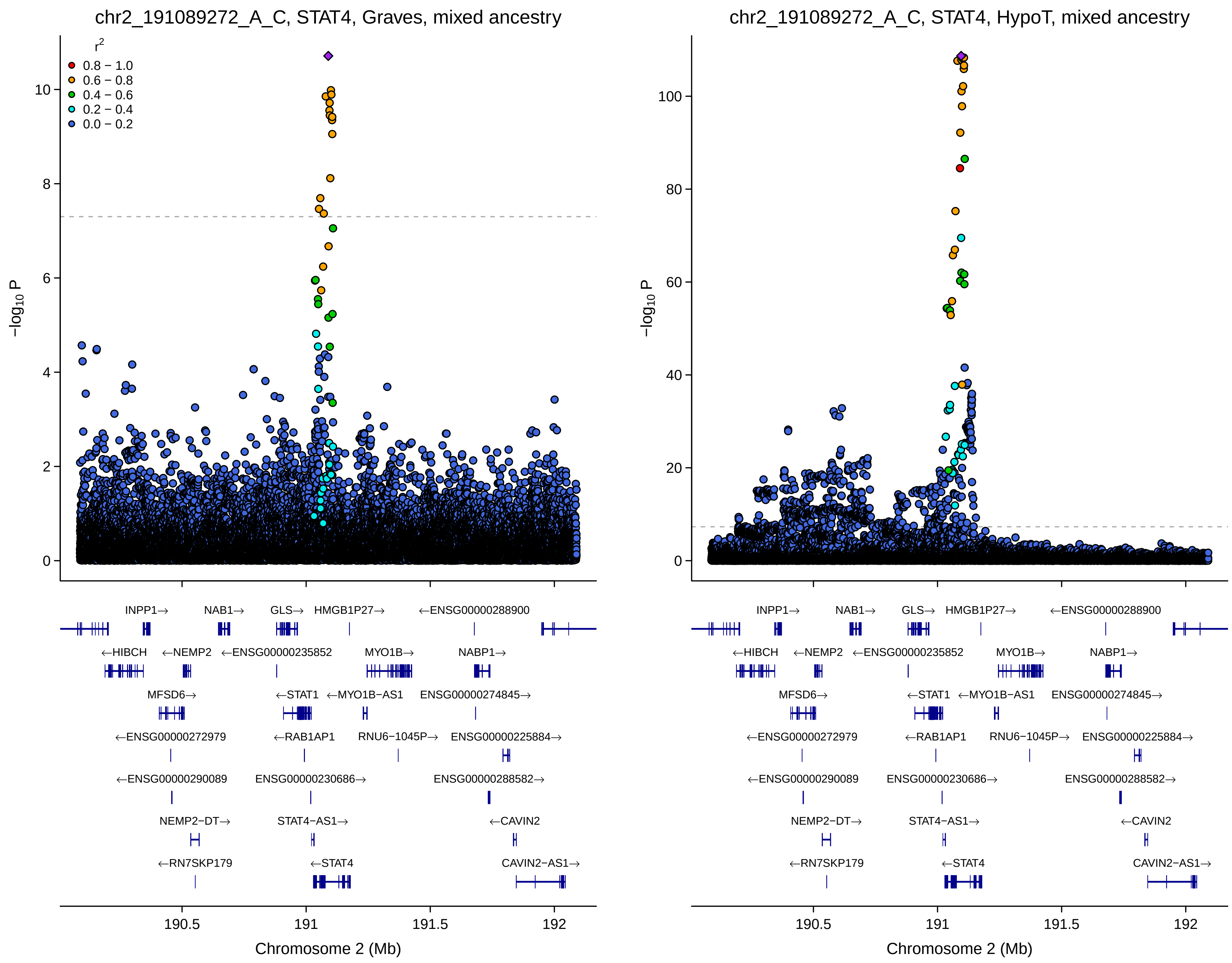

Supplementary Figure 3.2

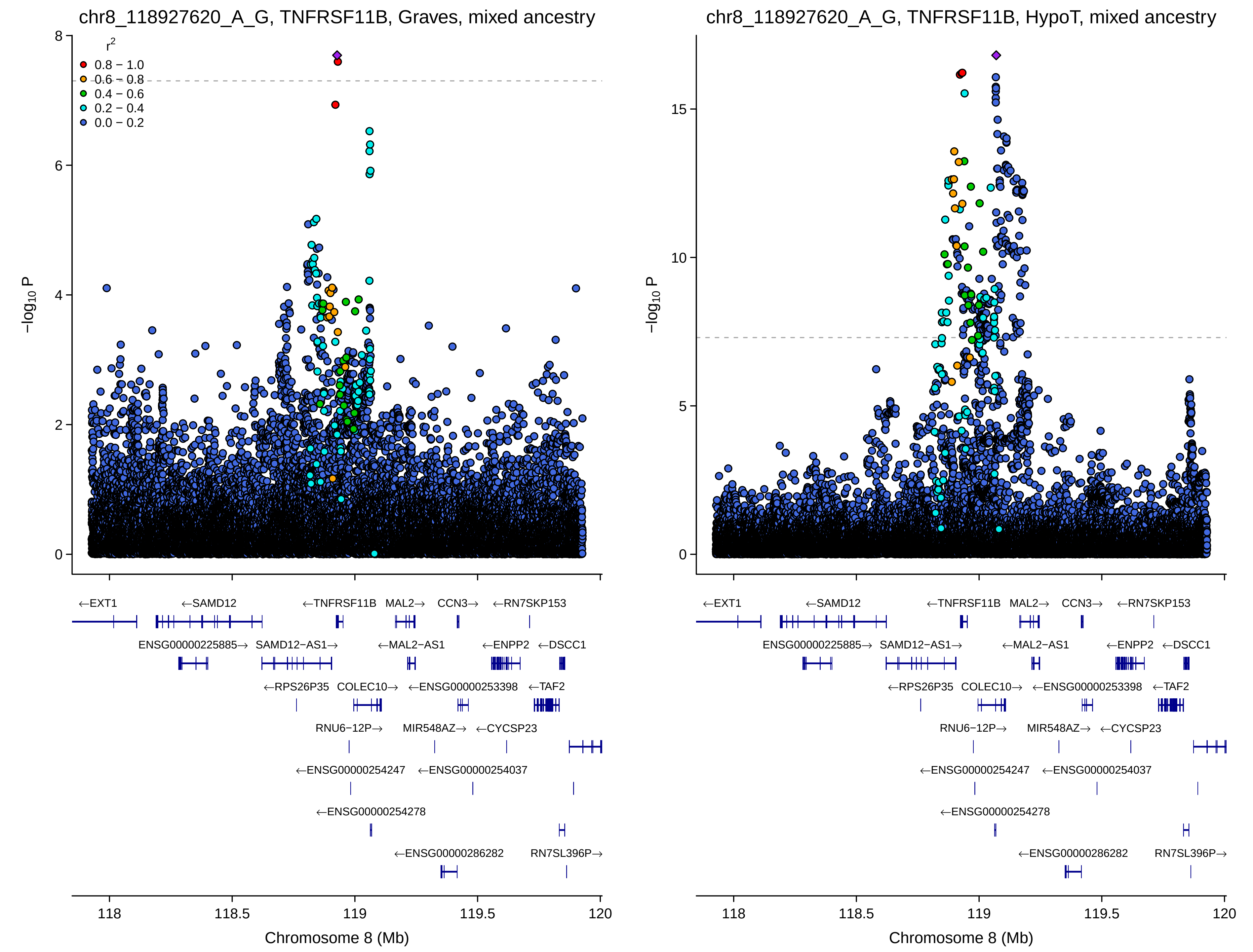

Supplementary Figure 3.2

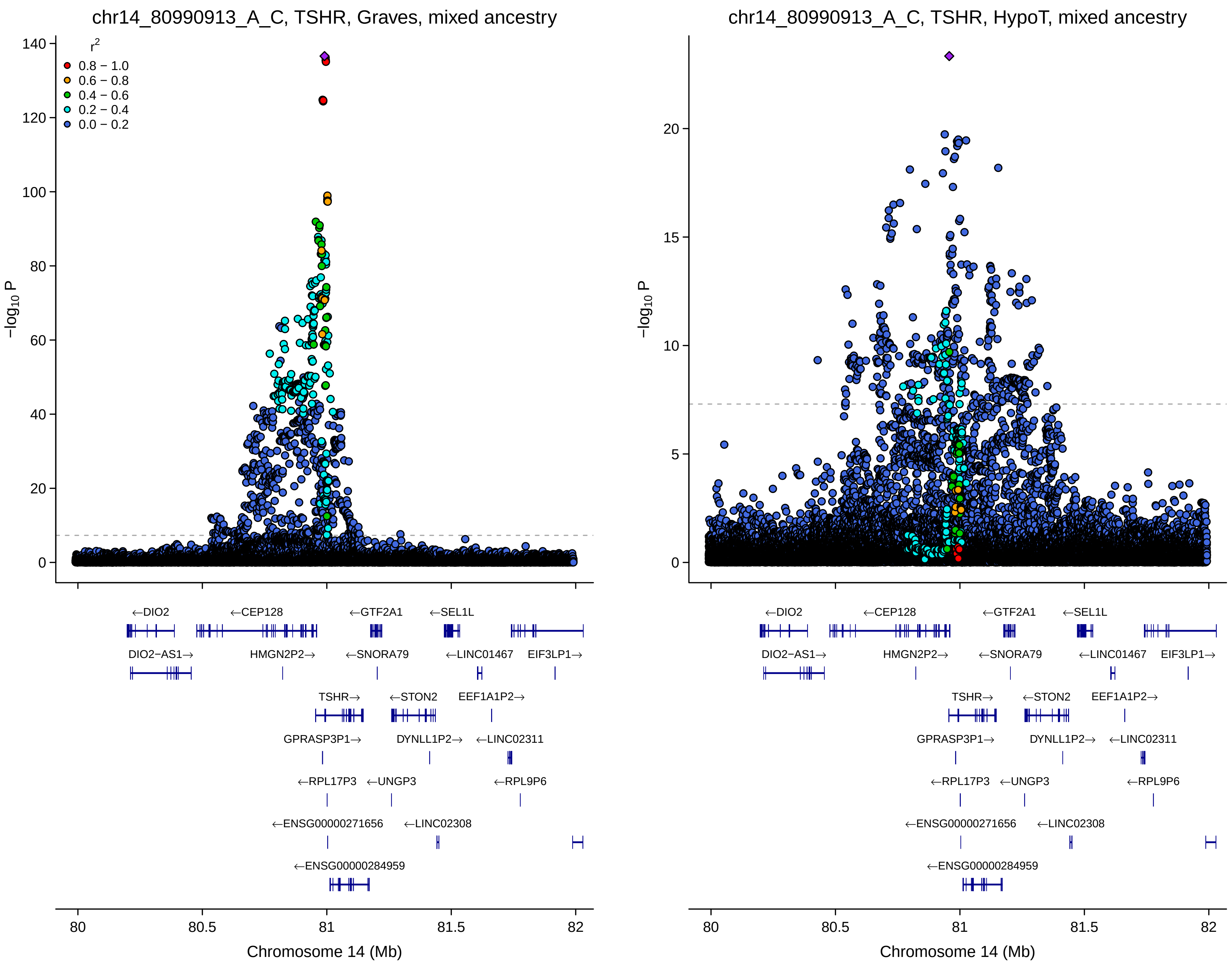

Supplementary Figure 3.2

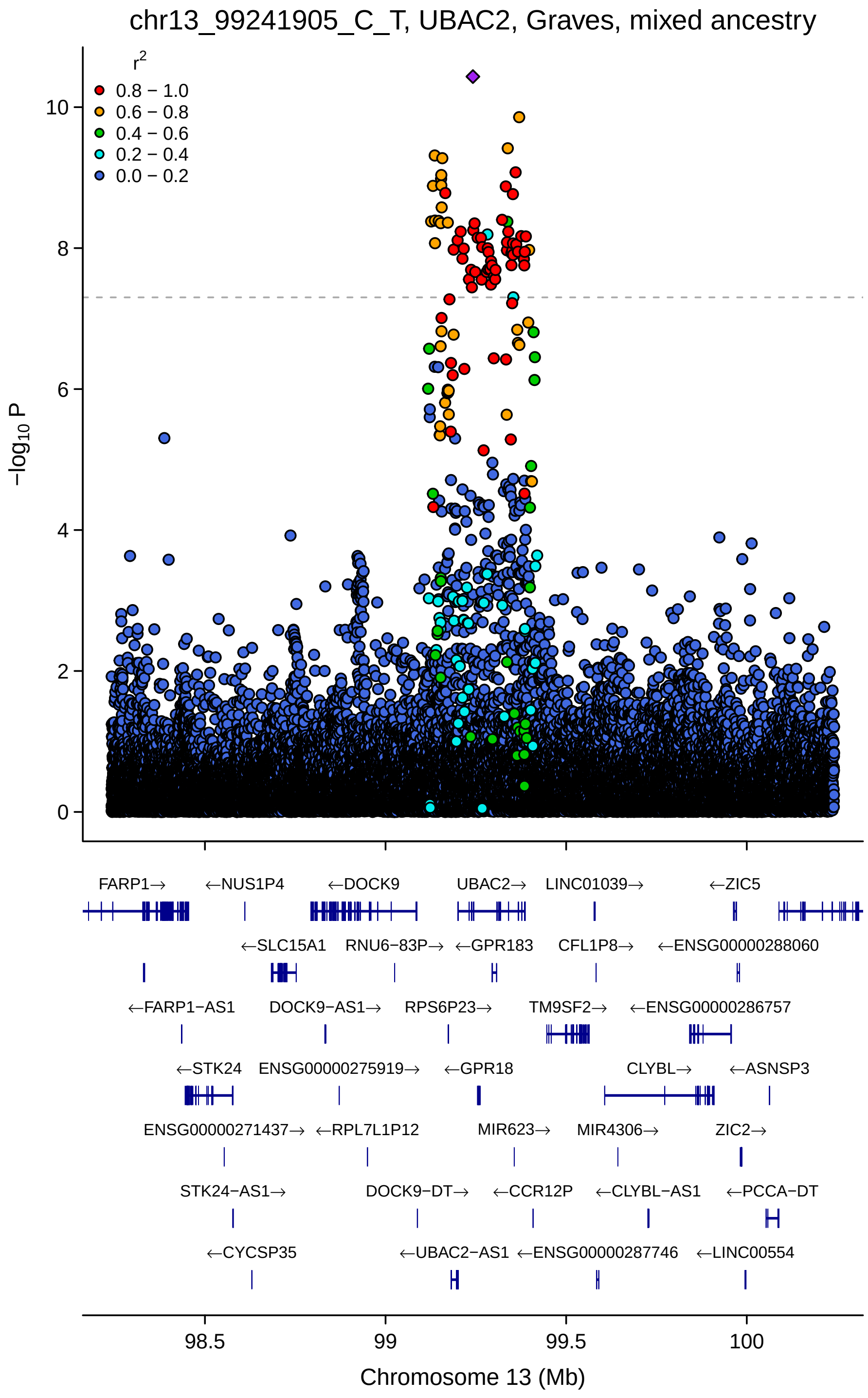

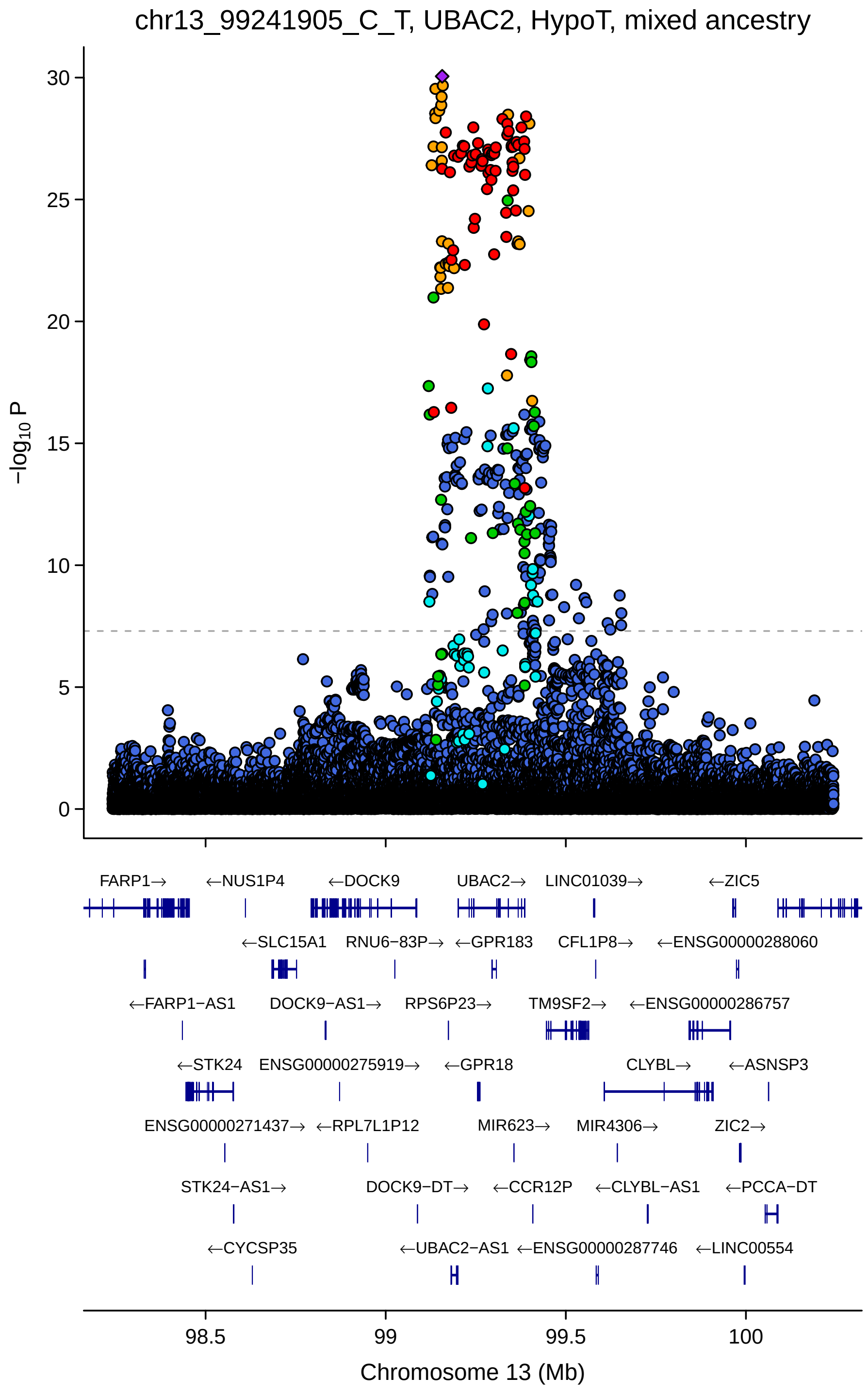

Supplementary Figure 3.2

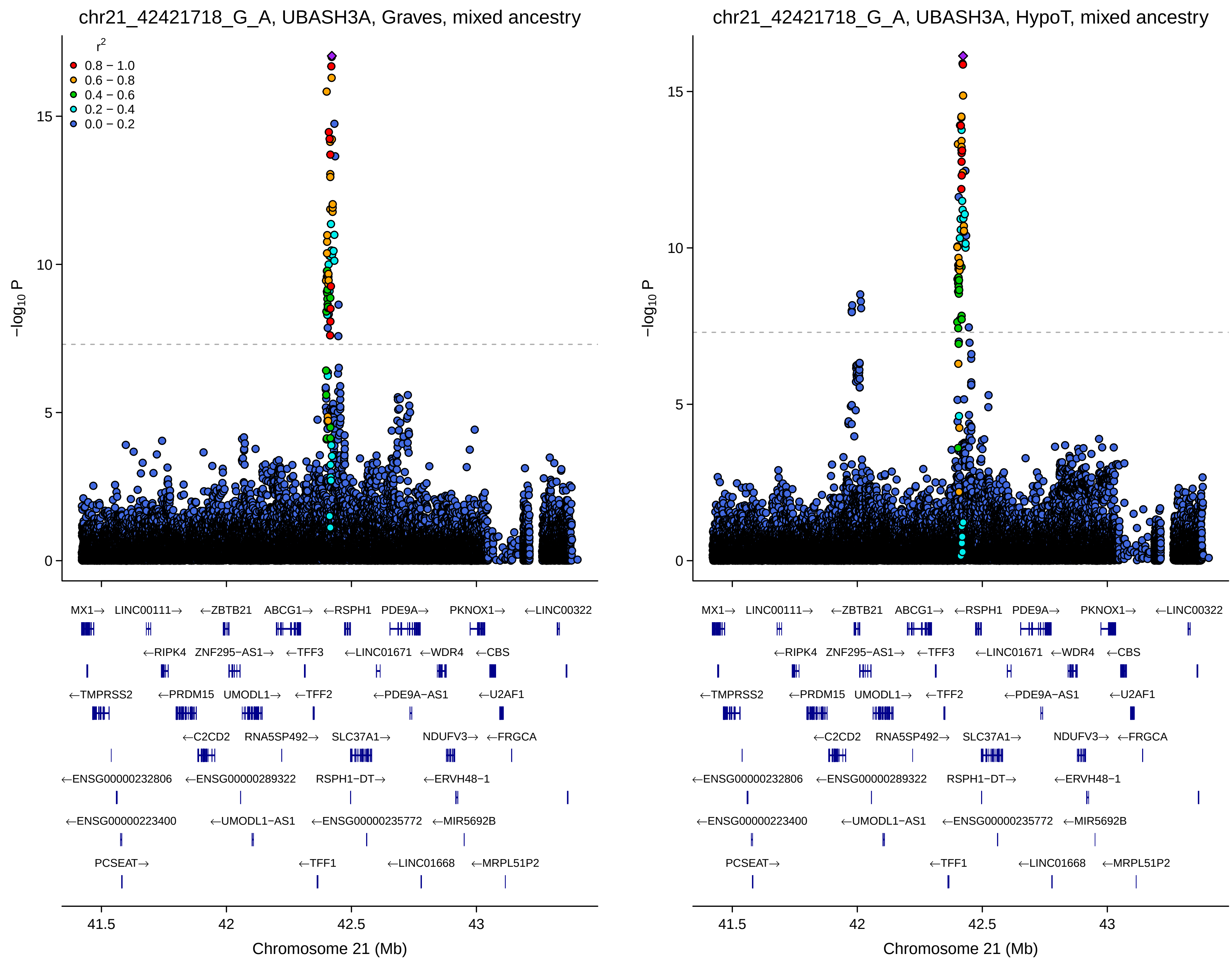

Supplementary Figure 3.2

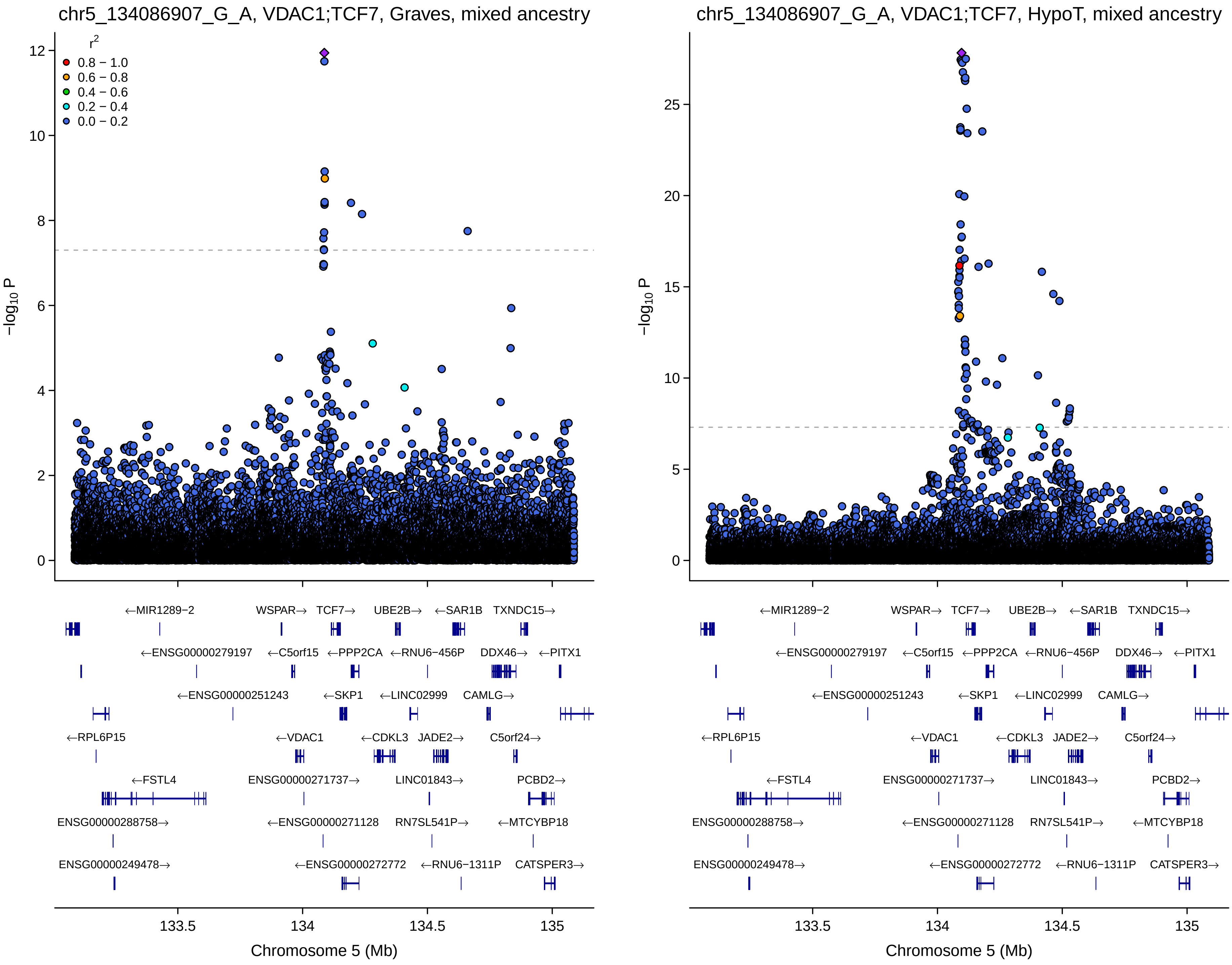

Supplementary Figure 3.2

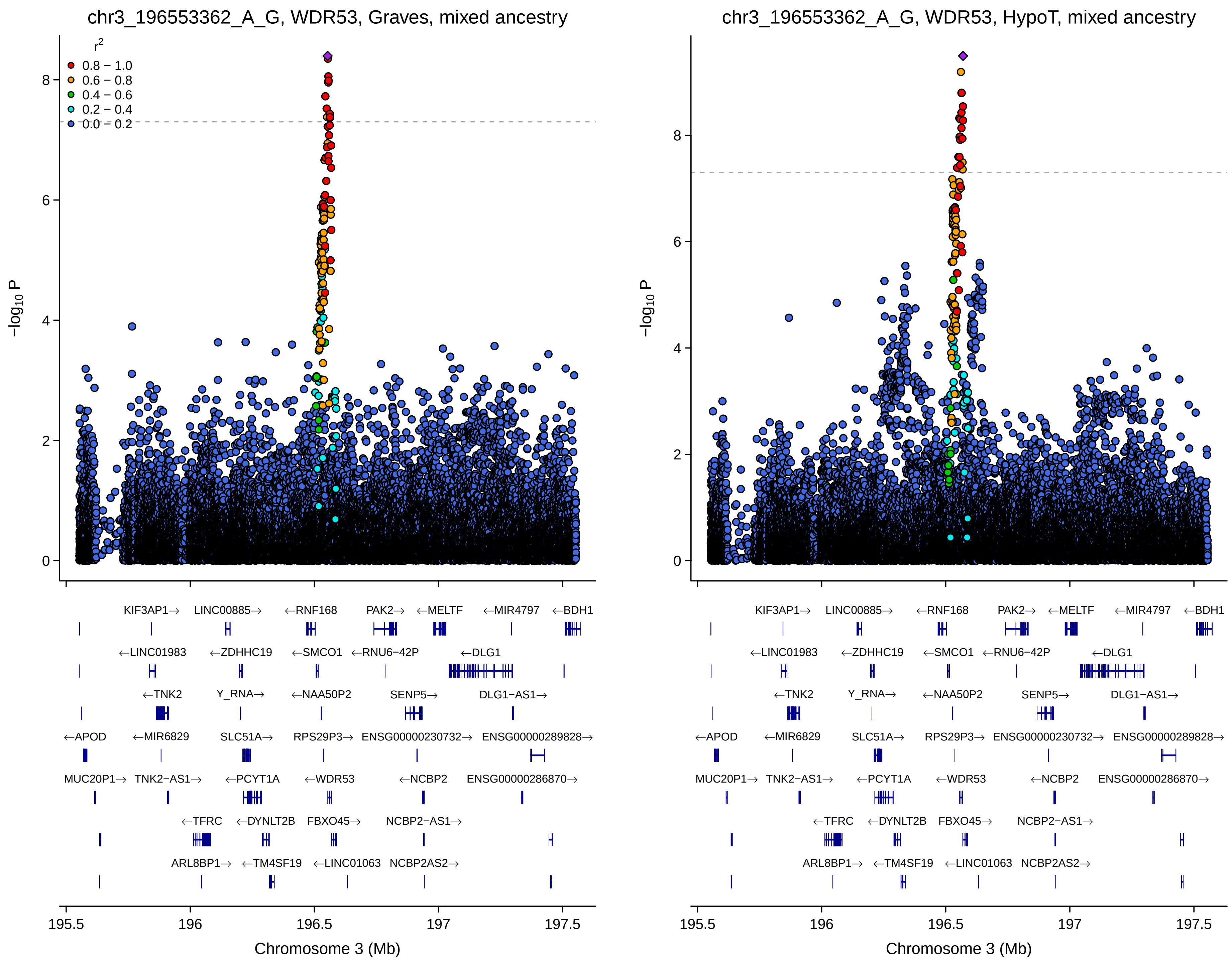

Supplementary Figure 3.2

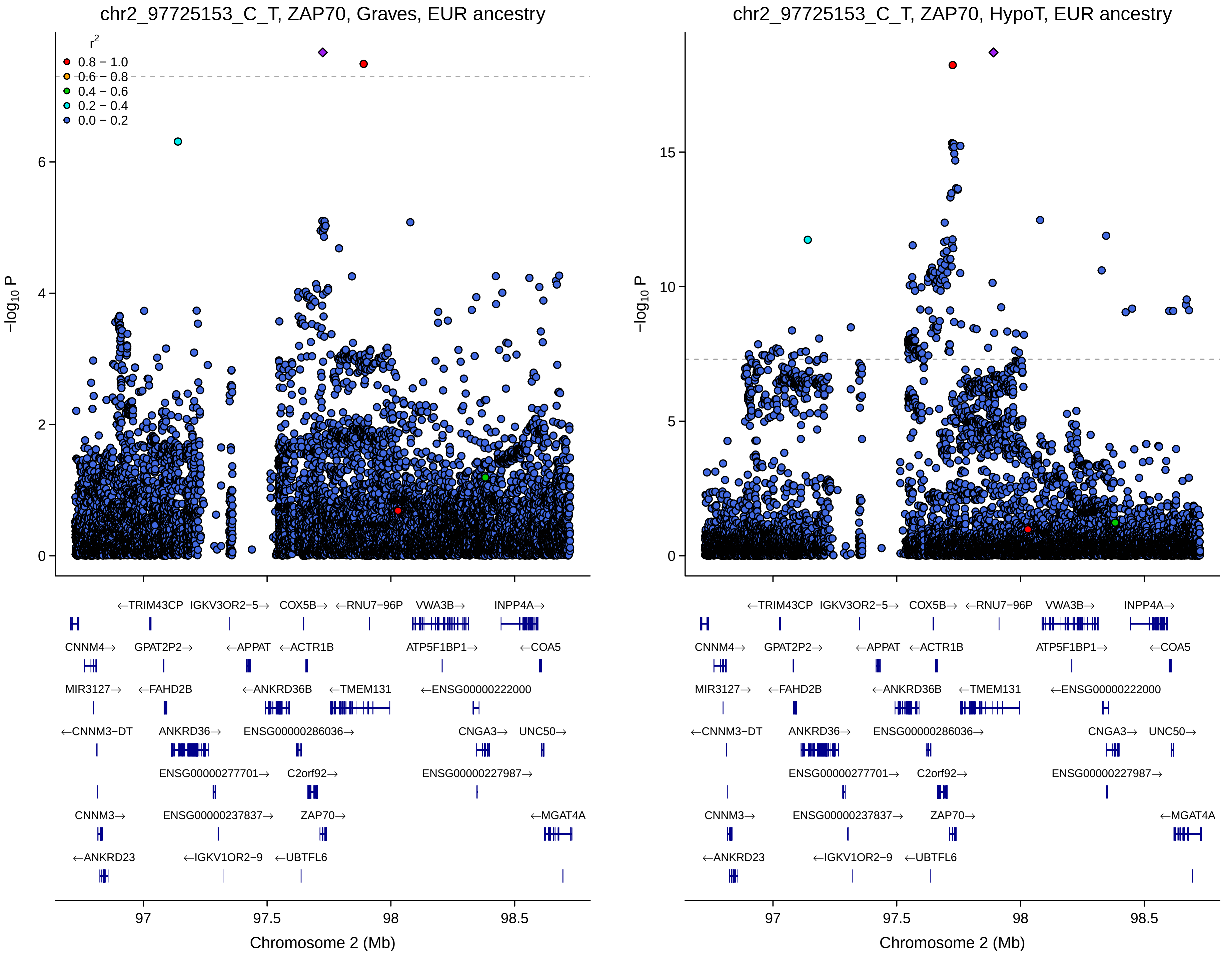

Supplement: Supplementary file 1 — Supplementary Figs. 1–3 and Tables 1–17. [file 41588_2025_2483_MOESM1_ESM.pdf]
