# Supplementary material for: Global multi-ancestry genome-wide analyses identify genes and biological pathways associated with thyroid cancer and benign thyroid diseases
Source: Nat Genet. 2026 Feb 5;58(2):307–16. doi: 10.1038/s41588-025-02483-w (PMC12900643; doi:10.1038/s41588-025-02483-w)
Supplement: Supplementary file 2 — Reporting Summary [file 41588_2025_2483_MOESM2_ESM.pdf]

nature portfolio

Corresponding author(s): Nikita Pozdeyev, Christopher R. Gignoux

Last updated by author(s): Oct 27, 2025

Reporting Summary

Nature Portfolio wishes to improve the reproducibility of the work that we publish. This form provides structure for consistency and transparency in reporting. For further information on Nature Portfolio policies, see our Editorial Policies and the Editorial Policy Checklist.

Statistics

For all statistical analyses, confirm that the following items are present in the figure legend, table legend, main text, or Methods section.

n/a	Confirmed	
☐	☒	The exact sample size (n) for each experimental group/condition, given as a discrete number and unit of measurement
☒	☐	A statement on whether measurements were taken from distinct samples or whether the same sample was measured repeatedly
☐	☒	The statistical test(s) used AND whether they are one- or two-sided<br>*Only common tests should be described solely by name; describe more complex techniques in the Methods section.*
☐	☒	A description of all covariates tested
☐	☒	A description of any assumptions or corrections, such as tests of normality and adjustment for multiple comparisons
☐	☒	A full description of the statistical parameters including central tendency (e.g. means) or other basic estimates (e.g. regression coefficient) AND variation (e.g. standard deviation) or associated estimates of uncertainty (e.g. confidence intervals)
☐	☒	For null hypothesis testing, the test statistic (e.g. F, t, r) with confidence intervals, effect sizes, degrees of freedom and P value noted<br>*Give P values as exact values whenever suitable.*
☒	☐	For Bayesian analysis, information on the choice of priors and Markov chain Monte Carlo settings
☒	☐	For hierarchical and complex designs, identification of the appropriate level for tests and full reporting of outcomes
☐	☒	Estimates of effect sizes (e.g. Cohen's d, Pearson's r), indicating how they were calculated

Our web collection on statistics for biologists contains articles on many of the points above.

Software and code

Policy information about availability of computer code

Data collection	Samples and genetic data were collected by participating biobanks.
Data analysis	Original code is publicly available at GitHub at https://github.com/pozdeyevlab/gwas-analysis and Zenodo https://doi.org/10.5281/zenodo.17468664 74.<br><br>ANNOVAR version date June 7, 2020; https://annovar.openbioinformatics.org/en/latest/<br>Bcftools v1.16 ; https://samtools.github.io/bcftools/bcftools.html<br>LD score regression v1.0.1; https://github.com/bulik/ldsc<br>Covariate adjusted LD score regression, v1.0.0; https://github.com/immunogenomics/cov-ldsc/blob/master/ldsc.py<br>GNOMAD v4.0; https://gnomad.broadinstitute.org/data#v4<br>HAIL version v0.2.91; https://hail.is/docs/0.2/api.html<br>METAL version date May 5, 2020; https://genome.sph.umich.edu/wiki/METAL_Documentation<br>PLINK v1.9; https://www.cog-genomics.org/plink/<br>PLINK2 v2.0; https://www.cog-genomics.org/plink/2.0/<br>PRScs version date May 14, 2024; https://github.com/getian107/PRScs<br>REGENIE v3.2.4; https://rgcgithub.github.io/regenie/<br>S-PrediXcan v0.73; https://github.com/hakyimlab/MetaXcan<br>SAIGE v1.5.0.2; https://saigegit.github.io/SAIGE-doc/<br>Summix2 v2.8.0; https://www.bioconductor.org/packages/release/bioc/vignettes/Summix/inst/doc/Summix.html<br>TWAS/FUSION; http://gusevlab.org/projects/fusion/<br>reactome.db v1.86.2; https://reactome.org/

ReactomePA v 1.46.0 ; https://pubmed.ncbi.nlm.nih.gov/26661513/
clusterProfiler v4.10.1; https://www.cell.com/the-innovation/fulltext/S2666-6758(21)00066-7?_returnURL&
R v4.3.1; https://www.r-project.org/
Python v3.11.10; https://www.python.org/downloads/

For manuscripts utilizing custom algorithms or software that are central to the research but not yet described in published literature, software must be made available to editors and reviewers. We strongly encourage code deposition in a community repository (e.g. GitHub). See the Nature Portfolio guidelines for submitting code & software for further information.

Data

Policy information about availability of data

All manuscripts must include a data availability statement. This statement should provide the following information, where applicable:

- Accession codes, unique identifiers, or web links for publicly available datasets
- A description of any restrictions on data availability
- For clinical datasets or third party data, please ensure that the statement adheres to our policy

The GWAS meta-analysis summary data are available for download from the GWAS Catalog (https://www.ebi.ac.uk/gwas/; accession numbers GCST90627737-GCST90627776). The PRS weights are deposited in the PGS Catalog (https://www.pgscatalog.org/; accession number PGP000748).
The proprietary Avatar data used in this study was generated through private funding by Aster Insights (www.asterinsights.com) in collaboration with the Oncology Research Information Exchange Network (ORIEN®, www.oriencancer.org) and is not open source in public repositories; all inquiries regarding access to the data or collaboration within ORIEN should be submitted to the corresponding author or https://researchdatarequest.orienavatar.com/.
Further information and requests for resources should be directed to and will be fulfilled by the lead contact, Nikita Pozdeyev, email: nikita.pozdeyev@cuanschutz.edu.
Original code is publicly available at GitHub at https://github.com/pozdeyevlab/gwas-analysis and Zenodo https://doi.org/10.5281/zenodo.17468664.

Research involving human participants, their data, or biological material

Policy information about studies with human participants or human data. See also policy information about sex, gender (identity/presentation), and sexual orientation and race, ethnicity and racism.

Reporting on sex and gender	Both sexes were included in the study and sex was used as a covariate in the analyses. Study findings apply to both sexes. Sex was determined from the genotyping data. Gender information was not collected or used in this study.
Reporting on race, ethnicity, or other socially relevant groupings	Genetically inferred ancestry was estimated as described in the Supplementary Table 1 for each participating biobank. Ancestry stratified meta-analyses were performed and results reported. Race or ethnicity was not used as a proxy for genetic ancestry.
Population characteristics	Population characteristics for each genome-wide association study are reported in the Supplementary Table 4.
Recruitment	Information on recruitment for each biobank is reported in the Supplementary Table 1.
Ethics oversight	Colorado Multiple Institutional Review Board for the University of Colorado Denver Anschutz Medical Campus, Aurora, Colorado, USA, waived ethical approval for this work (COMIRB #20-2315). This study is the result of a large collaborative effort among multiple biobanks and programs. Cohort-specific GWAS analyses were performed by local researchers. Data collections for the cohorts were approved by local ethics committees. Biobank participants were not compensated for their involvement in this study.

Note that full information on the approval of the study protocol must also be provided in the manuscript.

Field-specific reporting

Please select the one below that is the best fit for your research. If you are not sure, read the appropriate sections before making your selection.

☒ Life sciences ☐ Behavioural & social sciences ☐ Ecological, evolutionary & environmental sciences

For a reference copy of the document with all sections, see nature.com/documents/nr-reporting-summary-flat.pdf

Life sciences study design

All studies must disclose on these points even when the disclosure is negative.

Sample size	No predetermined sample size was used. All available data from participating biobanks was aggregated to maximize discovery power.
Data exclusions	No data were excluded from the analyses
Replication	Only variants tested by 4 or more biobanks were included in the multi-ancestry meta-analysis to ensure consistency across data sets. Heterogeneity was calculated using Cochran's Q measure. The number of biobanks with significant associations is reported for each variant.

Randomization | There is no allocation to experimental groups in this study. Randomization was not performed.

Blinding | Blinding is not relevant for this study.

Reporting for specific materials, systems and methods

We require information from authors about some types of materials, experimental systems and methods used in many studies. Here, indicate whether each material, system or method listed is relevant to your study. If you are not sure if a list item applies to your research, read the appropriate section before selecting a response.

Materials & experimental systems

n/a	Involved in the study
☒	☐ Antibodies
☒	☐ Eukaryotic cell lines
☒	☐ Palaeontology and archaeology
☒	☐ Animals and other organisms
☒	☐ Clinical data
☒	☐ Dual use research of concern
☒	☐ Plants

Methods

n/a	Involved in the study
☒	☐ ChIP-seq
☒	☐ Flow cytometry
☒	☐ MRI-based neuroimaging

Plants

Seed stocks | *Report on the source of all seed stocks or other plant material used. If applicable, state the seed stock centre and catalogue number. If plant specimens were collected from the field, describe the collection location, date and sampling procedures.*

Novel plant genotypes | *Describe the methods by which all novel plant genotypes were produced. This includes those generated by transgenic approaches, gene editing, chemical/radiation-based mutagenesis and hybridization. For transgenic lines, describe the transformation method, the number of independent lines analyzed and the generation upon which experiments were performed. For gene-edited lines, describe the editor used, the endogenous sequence targeted for editing, the targeting guide RNA sequence (if applicable) and how the editor was applied.*

Authentication | *Describe any authentication procedures for each seed stock used or novel genotype generated. Describe any experiments used to assess the effect of a mutation and, where applicable, how potential secondary effects (e.g. second site T-DNA insertions, mosiacism, off-target gene editing) were examined.*
